# High resolution genome-wide SNP array analyses on matched colorectal-based lung and brain metastases

**DOI:** 10.1007/s00432-026-06427-7

**Published:** 2026-01-30

**Authors:** Vivian-Pascal Brandt, Carolin Sander, Lydia Holland, Ronald Koschny, Wolf C. Müller, Hendrik Bläker, Ulf Nestler, Erdem Güresir, Heidrun Holland

**Affiliations:** 1 Department of Neurosurgery, University Hospital Leipzig, Leipzig, Saxony Germany; 2 Saxonian Incubator for Clinical Translation (SIKT), University of Leipzig, Leipzig, Saxony Germany; 3 Interdisciplinary Endoscopy Center (IEZ), Department of Gastroenterology and Hepatology, University Hospital Heidelberg, Heidelberg, Baden-Wuerttemberg Germany; 4 Paul Flechsig Institute of Neuropathology, University Medicine Leipzig, Leipzig, Saxony Germany; 5 Institute of Pathology, University Hospital Leipzig, Leipzig, Germany

**Keywords:** Colorectal cancer, Lung metastasis, Brain metastasis, SNP array, CNV, Cn-LOH

## Abstract

**Purpose:**

Colorectal-based brain metastasis formation is a rare and late event in colorectal cancer (CRC) patients and is associated with poor survival. Compared with other metastatic sites, the knowledge about copy number variation (CNV) in brain metastases is still very limited. To get more information about CNVs, we applied SNP array to analyze chromosomal regions with a higher density of SNP markers.

**Methods:**

Genome-wide high resolution single nucleotide polymorphism (SNP) array (CytoScan™ HD) analyses were carried out in matched colorectal-based lung and brain metastases of two patients.

**Results:**

Brain metastases harbored more CNVs (77 CNVs) than pulmonary metastases (24 CNVs). Not previously described specific CNVs were: gain of 1p36.33-p36.32, 4p16.3-p16.1, 6q27, 12q24.33, 16p13.3, as well as 16p12.1-p11.2 in lung metastases and gain of 1p36.33-p36.21, 5q11.1-q13.2, 21q22.2-q22.3, 22q11.21-q12.2, as well as 22q12.3-q13.33 in brain metastases.

Furthermore, we found 20 copy-neutral loss of heterozygosity (cn-LOH) regions exclusively in brain metastases, of which 11 cn-LOH regions have not been previously described.

**Conclusion:**

Brain metastases of CRC showed more cn-LOH regions than lung metastases. Potentially affected genes within these regions could influence signaling pathways (e.g., PI3K/AKT signaling) as well as transcriptional processes. Perspectively, increased awareness of specific genetic characteristics can potentially increase the chance of early diagnosis of brain metastases, which could contribute to improved treatment options.

**Supplementary Information:**

The online version contains supplementary material available at 10.1007/s00432-026-06427-7.

## Introduction

Colorectal cancer (CRC) is the fourth most common malignant tumor in humans and the third leading cancer-related mortality worldwide (Siegel et al. 2020). Currently, the standard therapy of CRC consists of surgical resection with neoadjuvant or adjuvant chemo- or radiotherapy according to the tumor stage (Vatandoust et al. 2015; Argilés et al. 2020). CRC mortality is mainly based on metastases formation as ~ 25% of CRC patients initial present with metastases, 50% of the CRC patients will develop metastases during the later course (van Cutsem and Oliveira 2009; Vatandoust et al. 2015). Pulmonary and hepatic metastases are common metastatic sites of CRC (van Cutsem et al. 2014). However, the development of brain metastases (BM) in CRC patients is very rare with an incidence of 0.6%—3.2%. These patients show a median overall survival of only ~ 5 months, which is worse compared to patients with breast cancer or lung cancer-based BM (Bartelt et al. 2004; Christensen et al. 2016).

Three carcinogenesis pathways can be distinguished in CRC: chromosomal instability (CIN), microsatellite instability (MSI) as well as the CpG island methylator phenotype (CIMP) (Pino and Chung 2010; Jasmine et al. 2012; Mauri et al. 2019). CRC predominantly develops through chromosomal instabilities like chromosomal rearrangements as well as copy number variations (Jasmine et al. 2012). Frequently detected chromosomal aberrations (> 20% of CRC patients) are gains of chromosome 7, 8q, 12, 13q, and 20q as well as the losses of chromosome 1p, 8p, 17p, 18q, 19p, and 22 (Joosse et al. 2018). Chromosomal aberrations emerge early in tumor development and the accumulation of these genetic alterations is important for tumor evolution. Additionally, the timeline of occurrence of specific genetic events is crucial, potentially bearing influence on metastases formation (Golas et al. 2022; Nguyen et al. 2022).

In primary CRC as well as CRC-based lung metastases, a plethora of genetic aberrations has already been identified. In 2011, Danner et al. (2011) carried out genetic analysis on 30 CRC patients and their corresponding 35 CRC-based lung metastases using comparative genomic hybridization assay (CGH). Generally, the mean average number of chromosomal alterations in lung metastases was higher compared to the primary CRC. These analyses revealed frequently detected gains of the chromosomal regions 7p/q, 8q, and 20q as well as frequently detected losses of the chromosomal regions 4p/q, 8p, 11q, 14q, 15q,18p/q, and 20p/q, for CRC-based lung metastases. Most frequently detected aberrations for both primary CRC and lung metastases were losses at chromosome 4, 8p, 11q, 14q, 15q, 17p, 18p/q, and 21q as well as gains at chromosome 6p, 7, 8q, 9q, 12p, 13q, and 20q. The loss of 5q were significantly more often detected in pulmonary metastases vs primary CRC (Danner et al. 2011).

Gutenberg et al. performed similar comparative genome-wide analyses on primary CRC vs CRC-based brain metastases. Using comparative genomic hybridization assay (CGH), the authors found significantly more aberrations in brain metastases vs primary CRC. The gains of 8q, 12p, 12q, and 20 p as well as the loss of 5q were detected only in brain metastases (Gutenberg et al. 2010). However, copy-neutral loss of heterozygosity (cn-LOH) cannot be detected by CGH and has not been sufficiently investigated in lung and brain metastases of CRC. Therefore, we had used high resolution SNP array on matched metachronous primary CRC, liver metastases and brain metastases in previous analyses revealing a higher number of cn-LOH aberrations in brain metastases compared to primary CRC and liver metastases. Possibly, the cn-LOH aberrations may be important for the CRC progression into the cerebral seeding phenotype (Brandt et al. 2023).

The aim of this study was to determine whether the more frequent occurrence of cn-LOH aberrations in brain metastases is also revealed for the metastases path from primary tumor to brain via lung. These investigations could contribute to an enlarged knowledge about the increased number of cn-LOH aberrations in brain metastases. Therefore, we investigated the genetic profile of matched and metachronous CRC-based pulmonary metastases vs brain metastases of two patients. These investigations were performed applying the more sensitive and high resolution CytoScan™ HD to analyze chromosomal regions with higher marker density. To the best of our knowledge, these are the first genetic analyses on matched and metachronous pulmonary and brain metastases using SNP array by applying the genome-wide high resolution CytoScan HD.

## Material and methods

### Patient material

**Fig. 1:**
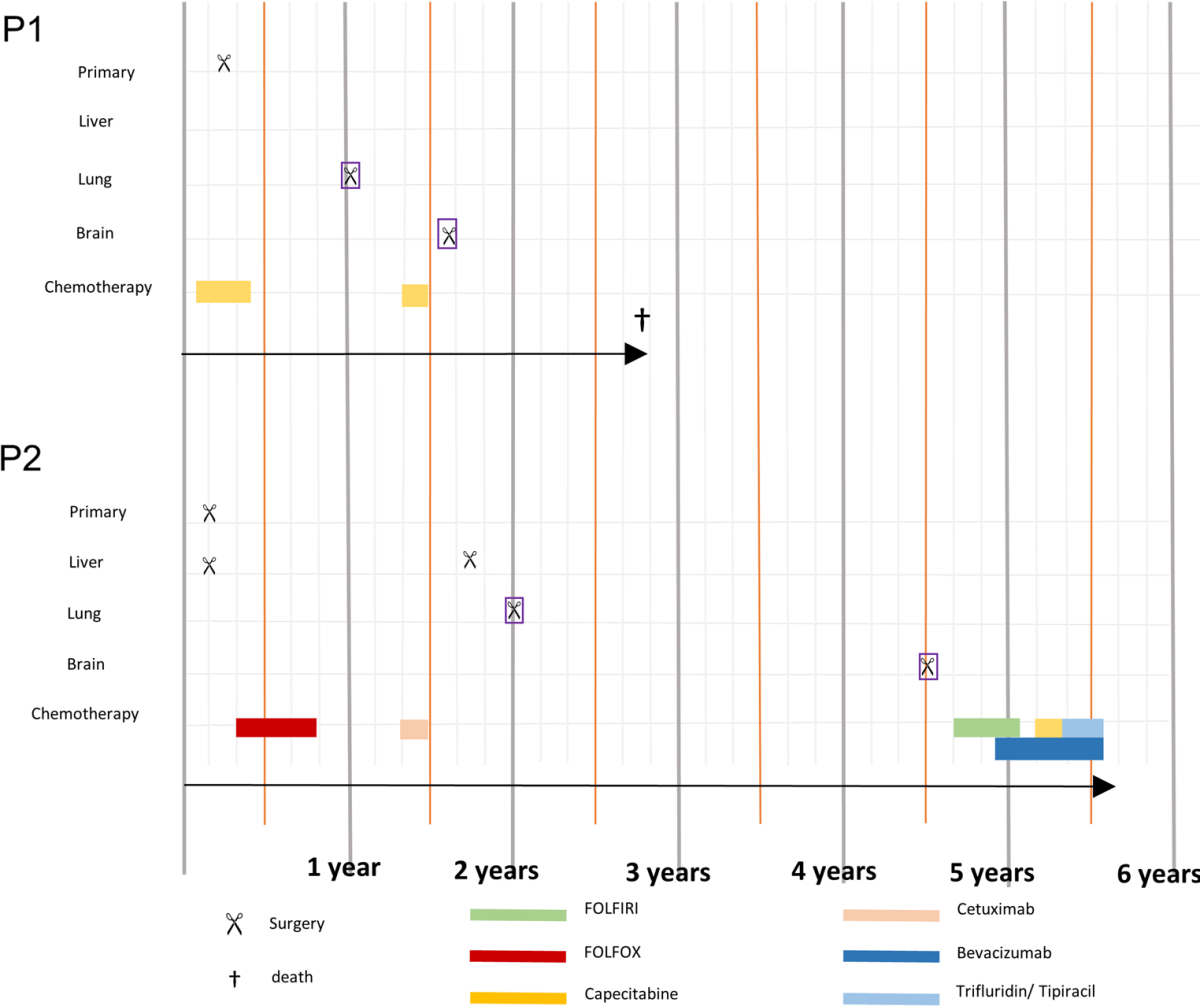
Timeline of tumor progression and therapy in both patients (P1-P2). Sampling dates are violet framed. The sample material of the brain metastasis from patient 1 was collected from the first of two brain surgeries

The ethics vote for this study was obtained from the ethics committee of the University of Leipzig (AZ.: 005/17-ek). This study was carried out in accordance with the Declaration of Helsinki (as revised in Fortaleza, Brazil, in 2013). The consent of the patients was given after prior clarification, taking into the account the anonymity of the data. Two CRC patients with metachronous lung metastases which were operated on metachronous brain metastases were included (see Fig. 1).

Primary CRCs were localized in the rectum. The investigated metastases were diagnosed at an age of 50.5 years (for lung metastases) as well as at an age of 52 years (for brain metastases). One patient is still alive. The patient characteristics are summarized in Table 1.

**Table 1:** Overview of the clinical characteristics for each patient including tumor progression and treatment

Characteristics	Patient 1 HK	Patient 2 NA
Gender	m	f
Age at first diagnosis [years]	52	46
Localization CRC	Rectum	Rectum
Initial TNM Stage	ypT3pN2a (4/18) M0 L1 V0 Pn0	ycT3 pN1 M0
Initial UICC Stage	III C	III B
Grading Latest documented M Stage	G2 brain, vertebra, lung	G2 brain, liver, lung, adrenal
CRC Subtype	KRAS mutated (pG12.D), Her2 amplification, NRAS-Wildtype (BM)	KRAS Wildtype, NRAS- Wildtype, MSS, BRAF-Wildtype
Chemotherapy	Neoadjuvant / adjuvant with Capecitabin	adjuvant with FOLFOX, Cetuximab, FOLFIRI, Bevacizumab, Trifluridin/ Tipiracil
Radiation	Yes Rectum: neoadjuvant 50.4 Gy Brain: + Vertebra: 36 Gy	Yes Rectum: neoadjuvant 54.4 Gy Brain: + +
Interval between resection of colorectal cancer and lung metastasis [months]	9	21
Localization of initial brain metastasis	left temporal, left frontal, right cerebellar	right parietotemporooccipital
Volume brain metastasis [cm^3^]	12.9	26.3
Interval between diagnosis of colorectal cancer and brain metastasis [months]	17	52
Total Overall Survival [months]	33	68 (still alive)
Overall Survival after brain surgery [months]	17	13 (still alive)

+ adjuvant radiation of brain metastases: radiation of metastatic bed right cerebellar (36 Gy), brain metastases left temporal (15 Gy), left frontal (20 Gy), followed by resection and radiation of whole brain (30 Gy)

+  + adjuvant radiation of brain metastases: radiation of metastatic bed with 42 Gy, subsequently followed by cerebellar stereo tactical radiation (22.5 Gy), stereotactic radiation of a right parietooccipital metastasis (25 Gy). Third brain radiation with stereotactic radiation of right precentral, right cerebellar and right postcentral metastases (total dose: 28.6 Gy)

#### Isolation and conservation of tumor tissue

Fresh non-necrotic tissue specimens were removed by surgery fixed in formalin and embedded in paraffin.

#### DNA isolation and molecular karyotyping using SNP array

Histopathological investigations on the collected tissue samples were performed before genetic analyses. All samples were subjected to analyses of genome-wide copy number variation (CNV) and copy number neutral losses of heterozygosity (cn-LOH) chromosomal regions using SNP array (CytoScan™ HD Array Kit, ThermoFisher Scientific, Dreieich, Hesse, Germany). Therefore, paraffin was dissolved in Xylene and lysed under denaturing conditions using Proteinase K. Genomic DNA of lung metastases as well as brain metastases were extracted according to the manufacturers’ protocols (QIAamp® DNA FFPE Tissue Handbook, QIAGEN, Hilden, North Rhine-Westphalia, Germany). Residual contaminants were removed using a column and pure genomic DNA was obtained. The eluted DNA was applied for CytoScan™ HD Array according to the manufacturers’ protocol (User Guide CytoScan™ Assay, Publication Number MAN0027808, Revision 8 by ThermoFisher Scientifc, Affymetrix, USA, Waltham). Subsequently, the obtained raw data were evaluated using the software Chromosome Analysis Suite, Version 4.5.0.34 (ThermoFisher Scientific Inc., USA, Waltham) according to the User Guide for Chromosome Analysis Suite (ChAS v4.5), Publication Number MAN0027798, Revision 19, available by ThermoFisher Scientific (ThermoFisher Scientific Inc., USA, Waltham). As reference model, CytoScanHD_Array.na36.r4.FFPE.REF_MODEL (as copy number reference and somatic mutations reference) was used. For evaluation of the data, pre-processing including Dual Quantile Normalization was performed, followed by Array Data QC Metrics and TuScan Algorithm (see User Guide for Chromosome Analysis Software v4.5, Appendix F). Copy Number Variations ≥ 3 Mb as well as Copy Neutral Loss of Heterozygosity ≥ 5 Mb were defined as reliable genetic alterations (Beroukhim et al. 2006; Žilina et al. 2015). The detailed workflow is available in the appendix of our previously published analyses (Brandt et al. 2023).

## Results

Altogether, we identified 24 aberrations (all of them were gains) in lung metastases and 97 aberrations (72 gains, 5 losses, 20 cn-LOHs) in brain metastases. The number of aberrations for each sample are listed in Table 2. Detailed information on each detected CNV is available in Table 5 in the appendix.

**Table 2:** Overview of the detected CNVs and cn-LOHs for lung and brain metastases, respectively (CNV ≥ 3 Mb, cn-LOH ≥ 5 Mb)

Patient ID	1	2
Lung metastases	CNV: 20 Gains: 20 Losses: 0 cn-LOH: 0	CNV: 4 Gains: 4 Losses: 0 cn-LOH: 0
Brain metastases	CNV: 25 Gains: 22 Losses: 3 cn-LOH: 7	CNV: 52 Gains: 50 Losses: 2 cn-LOH: 13

### Lung metastases

**Fig. 2:**
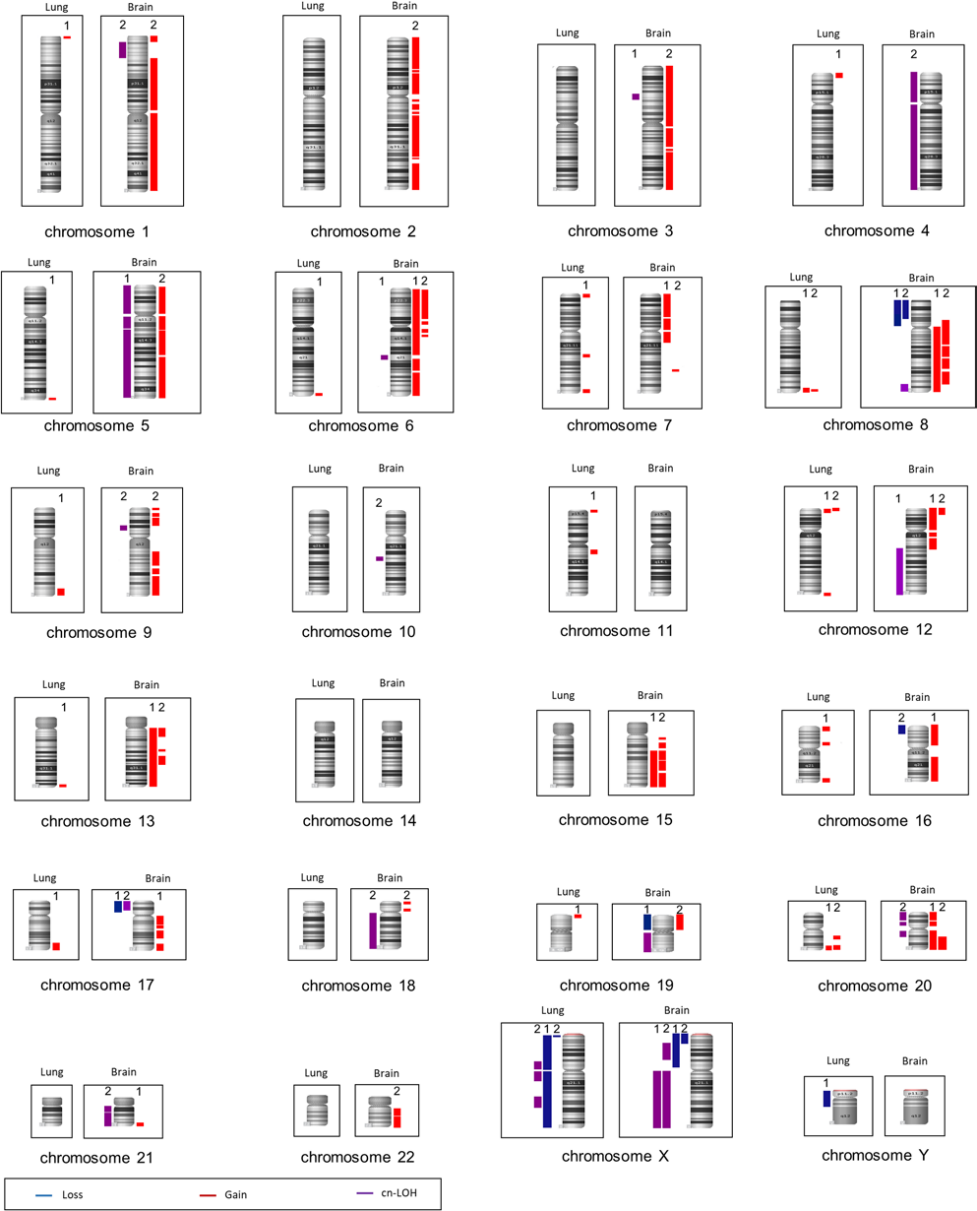
Frequencies of chromosomal aberrations (red: gain, blue: loss, violet: cn-LOH) in both patients. The chromosomes (from left to right) show detected aberrations in CRC-based lung metastases (left frame) and brain metastases (right frame). The numbers represent whether the aberrations were detected in patient 1 or patient 2

Gains of the chromosomal regions 8q24.3, 12p13.33-p13.32 and 20q13.33 were detected in lung metastases of both patients. Interestingly, gains of chromosomal regions were predominantly detected on the distal chromosomal regions (20 out of 24) (see Fig. 2). Not previously described chromosomal aberrations for CRC-based pulmonary metastases are: gain of 1p36.33-p36.32, 4p16.3-p16.1, 6q27, 12q24.33, 16p13.3, and 16p12.1-p11.2.

### Brain metastases

In the brain metastases, gains of chromosomal regions 6p25.3-p12.3, 6p12.2-p12.1, 6q12-q13, 6q13-q14.3, 8p11.21-q13.3, 8q13.3-q21.3, 8q22.1-q23.1, 8q23.3-q24.22, 12p13.33-p13.21, 13q12.11-q13.1, 13q14.3-q21.1, 13q21.31-q22.3, 15q21.1-q21.3, 15q22.2-q24.2, 15q24.3-q26.3, 20q12-q13.33 as well as loss of chromosomal region 8p23.3-p12 were detected in both patients. To the best of our knowledge, gains of the chromosomal regions 1p36.33-p36.21, 5q11.1-q13.2, 21q22.2-q22.3, 22q11.21-q12.2, as well as 22q12.3-q13.33 have not been previously described. Additionally, 20 cn-LOH aberrations (patient 1: 7 cn-LOH; patient 2: 13 cn-LOH) were identified. Interestingly, all cn-LOH aberrations were exclusively detected in brain metastases. The following cn-LOH regions have not been previously described: 1p36.21-p34.3, 4p16.3-p11, 4q12-q35.2, 5p15.33-p11, 6q16.3-q21, 8q24.22-q24.3, 10q22.1-q22.3, 20p13-p12.1, 20p12.1-q11.21, 20q11.21-q12 as well as 21q11.2-q21.2. Detailed information on the detected cn-LOH aberrations is listed in Table 3.

**Table 3:** Detailed overview of all detected cn-LOH regions ≥ 5 Mb

Chromosomal region	Aberration	Size (kb)	Start (kb)	End (kb)	Patient ID	previously described
1p36.21-p34.3	cn-LOH	23,024	15,372,831	38,396,459	2	No
3p21.31-p21.1	cn-LOH	6,017	47,612,885	53,630,208	1	Yes*
4p16.3-p11	cn-LOH	49,018	69,019	49,087,164	2	No
4q12-q35.2	cn-LOH	138,161	51,833,594	189,994,495	2	No
5p15.33-p11	cn-LOH	46,246	137,401	46,383,233	1	No
5q11.1-q13.2	cn-LOH	19,244	50,265,025	59,508,917	1	Yes*
5q13.2-q35.3	cn-LOH	109,874	71,384,705	181,258,604	1	Yes*
6q16.3-q21	cn-LOH	7,496	104,700,606	112,196,566	1	No
8q24.22-q24.3	cn-LOH	11,916	133,151,196	145,067,348	2	No
9p21.1-p13.2	cn-LOH	7,267	30,097,790	37,364,678	2	Yes*
10q22.1-q22.3	cn-LOH	6,123	72,092,798	78,215,437	2	No
12q14.2-q24.33	cn-LOH	70,192	63,009,396	133,201,580	1	Yes*
17p13.3-p11.2	cn-LOH	17,945	170,188	18,115,042	2	Yes*
18q11.1-q23	cn-LOH	59,276	20,980,393	80,256,699	2	Yes*
19q11-q13.43	cn-LOH	30,786	27,782,420	58,568,566	1	Yes*
20p13-p12.1	cn-LOH	14,451	94,613	14,546,046	2	No
20p12.1-q11.21	cn-LOH	7,859	15,510,115	23,369,112	2	No
20q11.21-q12	cn-LOH	10,836	31,394,130	42,230,410	2	No
21q11.2-q21.2	cn-LOH	10,189	13,757,057	23,946,545	2	No
21q21.2-q22.3	cn-LOH	21,618	25,040,343	46,657,900	2	Yes*

*cn-LOH* copy neutral loss of heterozygosity

^*^aberrant chromosomal region previously described inBrandt et al. (2023)

According to the NCG7.0 Network of Cancer Genes & Healthy Drivers, a total of 730 different cancer genes are located within the detected cn-LOH regions (Dressler et al. 2022). All identified cancer genes are listed in Table 6 of the appendix. Performing pathway analyses using Reactome database, these cancer genes could possible significantly affect signaling pathways (e.g., PI3K / AKT signaling) as well as transcriptional processes. Detailed information on the most relevant pathways is given in Table 4.

**Table 4:** The most significantly affected pathways according to Reactome database, based on the p-values. Pathways that have already been described as potentially affected on the detected cn-LOH aberrations in CRC – liver metastases – brain metastases are marked in bold

Pathway name	*p*-value
Nuclear Receptor transcription pathway	4.02*10^–8^
Netrin-1 signaling	2.37*10^–6^
**Generic Transcription Pathway**	5.97*10^–6^
**RNA Polymerase II Transcription**	1.12*10^–5^
PI3K/AKT Signaling in Cancer	1.22*10^–5^
Diseases of signal transduction by growth factor receptors and second messengers	2.66*10^–5^
**Gene expression (Transcription)**	4.37*10^–5^
**Transcriptional regulation by RUNX3**	6.01*10^–5^
Intracellular Signaling by second messengers	8.03*10^–5^
PIP3 activates AKT signaling	9.83*10^–5^
Developmental Biology	9.83*10^–5^
Signaling by NOTCH1	1.16*10^–4^
DCC mediated attractive signaling	1.22*10^–4^
**Signaling by Receptor Tyrosine Kinases**	1.49*10^–4^
Constitutive Signaling by Aberrant PI3K in Cancer	4.69*10^–4^

### Comparison of lung and brain metastases

Our genetic analyses revealed a heterogeneous genetic profile of lung and brain metastases. More chromosomal aberrations were detected in brain metastases (97 aberrations including 72 gains, 5 losses and 20 cn-LOH aberrations) than in lung metastases (24 aberrations, all of them gains, Table 2). The following chromosomal aberrations were identified in lung metastases as well as in brain metastases (of patient 1 or 2, respectively): gains of 1p36.33-p36.32, 5q35.3, 6q27, 7p22.3-p22.1, 8q24.3, 9q33.3-q34.3, 13q34, 16p13.3, 16q23.3-q24.3, 17q25.1-q25.3 as well as 19p13.3. Interestingly, gain of 12p13.33-p13.32 and 20q13.33 were identified for both lung and brain metastases of both patients. A more general overview of all affected chromosomal arms is given in our Venn diagram (see Fig. 5 in the supplemental part).

**Fig. 3:**
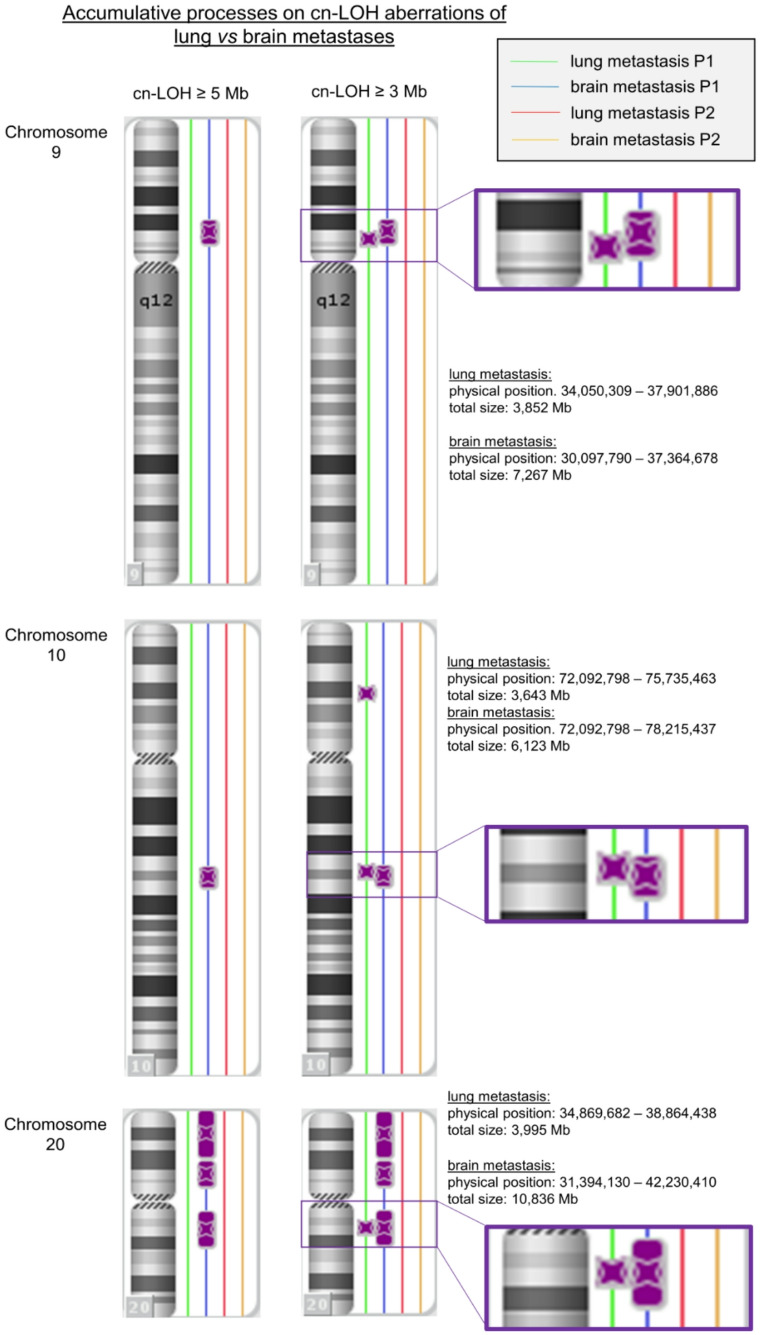
Accumulative processes on cn-LOH regions in lung metastases vs brain metastases. For the chromosomes 9, 10, and 20, smaller cn-LOHs in lung metastases (≥ 3 Mb) *vs* cn-LOHs in brain metastases (≥ 5 Mb) were detected at the same chromosomal regions

For the identification of possible accumulative processes on cn-LOH aberrant regions, a reduced cut off level of ≥ 3 Mb was set to detect smaller chromosomal aberrations too. Therefore, accumulative processes were revealed for the chromosomal regions 9p [lung metastases: 9p13.3-p13.2 (3,852 Mb); brain metastases: 9p21.1-p13.1 (7,267 Mb)], 10q [lung metastases: 10q22.1-q22.2 (3,643 Mb); brain metastases: 10q22.1-q22.3 (6,123 Mb)], and 20q [lung metastases: 20q11.22-q11.23 (3,995 Mb); brain metastases: 20q11.21-q12 (10,836 Mb)]. An overview is shown in Fig. 3.

**Fig. 4:**
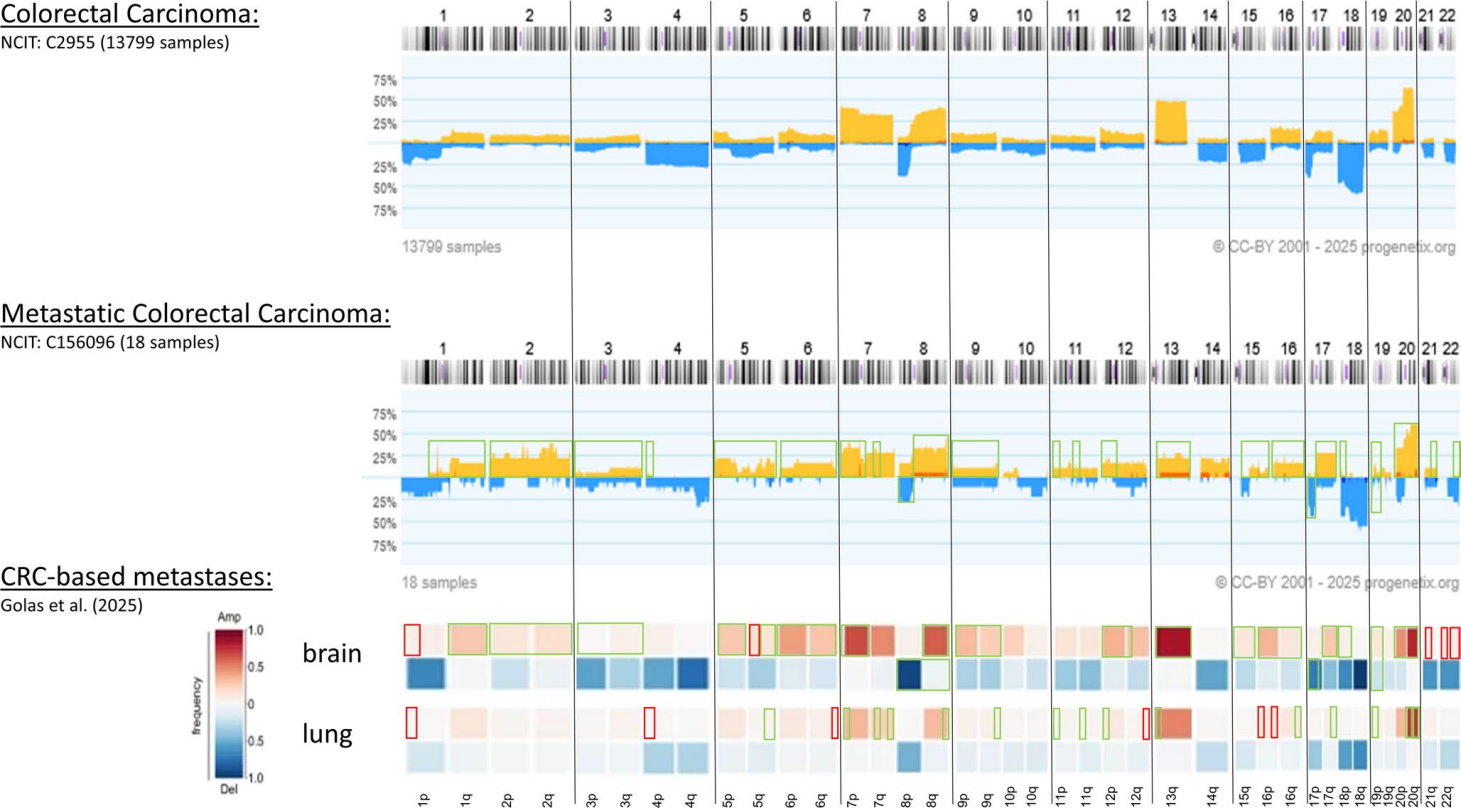
Comprehensive comparison of the results obtained database and recently published data for each chromosome. The Progenetix database was used for Colorectal Carcinoma (top line) as well as for Metastatic Colorectal Carcinoma (middle line). In the bottom line, results of genetic analyses of lung and brain metastases of Golas et al. (2025) are shown. Green framed boxes: the aberration could also be identified in our analyses. Red framed boxes: aberrations were not previously described

Furthermore, we performed an extensive comparison of the results obtained from the genome-wide investigations on CRC-based lung and brain metastases vs previously described aberrations for metastases of CRC using the Progenetix database as well as the recently published results from Golas et al. (2025). This comparison revealed a certain comparability of the detected aberrations and seems to be specific for the metastatic site, respectively (see Fig. 4).

Considering the results from our previously performed study on primary CRC – liver metastases – brain metastases, the following chromosomal aberrations were identified for all four unmatched tumor samples (detected at least one time for primary CRC and each metastasis): gain of 5q35.3, 7p22.2-p22.1, 7q21.3-q22.1, 7q36.2-q36.3, 8q24.3, 12p13.31, 12q24.33, 13q34, 16p13.3, 16p12.1, 16q23.3-q24.1, and 20q13.2-q13.33 (see Fig. 6 in the appendix). Gain of 20q13.33 was detected in each tumor sample of all 6 patients.

## Discussion

The occurrence of brain metastases is a rare event in CRC patients and is associated with an unfavorable prognosis. In the present study, we highlighted the comparative genetic analyses of CRC-based pulmonary and brain metastases using CytoScan™ HD array for the analyses with a higher marker density.

We identified previously described chromosomal aberrations for CRC-based pulmonary as well as brain metastases. The gain of chromosomal region 20q13.33 could be of special interest, because it was detected in each tumor/metastases sample of primary CRC, liver metastases and brain metastases (Brandt et al. 2023). Golas et al. (2025) revealed gains of 20q in their analyses too. These aberrations represent an early event in the cytogenetic tumor evolution of CRC, independent of the location of later occurring metastases. The authors point out, that the timing and chronology of the occurrence of aberrations in primary tumor may also have an influence on metastases formation (Golas et al. 2025). Using array-based Comparative Genomic Hybridization (aCGH) and quantitative Polymerase chain reaction (qPCR), a study on 571 CRC patients have revealed gain of 20q13.33 as the main chromosomal abnormality in CRC and colon polyps (Bui et al. 2020). Within this region, the tumor suppressor gene *CDH4* is located, which encodes for a cell surface glycoprotein (as member of the cadherin family) involved in cell adhesion. Miotto et al. identified an aberrant gene promotor methylation of this tumor suppressor in 78% of colorectal carcinomas followed by an loss of gene expression (Miotto et al. 2004).

Furthermore, we identified gain of 9q33.3-q34.3 for the lung and brain metastases. Danner et al. (2011) had found gains on 9q for pulmonary metastases too. Interestingly, these gains of 9q occurred with lower frequencies in lung metastases vs primary CRC (Danner et al. 2011). Recently, Kelly et al. (2024) published their chromosomal microarray results on primary and metastatic tumor specimens of stage IV lung adenocarcinoma. The authors identified the gain of 9q22.32-q34.11 solely in metastatic specimen and they suggest a possible impact on tumor progression and metastases formation (Kelly et al. 2024). The chromosomal region 1p36.33 with detected gains for lung as well as brain metastases could be of special interest. Aberrations on chromosome 1p36 are described for intracranial tumors, e.g., glioblastoma, neuroblastoma or oligodendroglioma (Korshunov et al. 2007; Yao et al. 2020; Wang et al. 2023). According to the NCG7.0 Network of Cancer Genes & Healthy Drivers, the cancer gene PRKCZ is localized within this aberrant region (Dressler et al. 2022). This protein kinase C is involved in the TGF-beta signaling pathway (Milacic et al. 2024). Jacob et al. performed gene expression analyses and revealed a dysregulation of the TGF-beta pathway for CRC-based brain metastases formation. Their results suggest that TGF beta signaling could reduce brain metastases formation of CRC patients. Still, the influence of TGF beta signaling on brain metastases formation is not completely understood (Jacob et al. 2024). In further analyses, it is useful to understand more deeply the impact of this signaling pathway for the cerebral metastases’ formation.

We found gain of chromosomal region 8p11.21-q24.3 in brain metastases. This aberration could already be identified both in our previously performed study on primary CRC and matched liver and brain metastases (detected for primary CRC and both metachronous metastases) as well as by Gutenberg et al. (2010). The gain of chromosome 8q occurred significantly more frequently in brain metastases than in the primary CRC tissue. Within these aberrant regions, oncogenes like *MYC* and *RAB2* are located (Masramon et al. 1998; Agrawal et al. 2002; Gutenberg et al. 2010).

In lung metastases, gain of chromosomal region 16p13.3 has not been previously described. Within this chromosomal region, the tumor suppressor gene *TSC2* is located. This gene is frequently mutated in non-small cell lung cancer (NSCLC). It potentially affects the immune checkpoint blockade in NSCLC (Huang et al. 2022). Interestingly, mutations of *TSC2* gene are described with an increased risk for the formation of brain metastases of papillary thyroid cancer (PTC) (Luo et al. 2021).

These genome-wide analyses identify chromosomal imbalances that may be significant for brain metastases formation. Genetic predisposition may also contribute to these brain metastases. Mei et al. (2025) provides interesting insights with analyses on breast cancer and these investigations suggests that a missense germline variant is associated with the initiation of lung metastases as well as with the proliferation of the metastatic cells (Mei et al. 2025).

High resolution genome-wide SNP array enables the detection of CNVs as well as cn-LOH aberrations. In our samples, cn-LOH aberrations were detected in brain metastases exclusively. We could confirm some cn-LOH aberrations from our previously published study, e.g., 17p13.3-p11.2. Interestingly, the tumor suppressor gene TP53 is located within this region (Brandt et al. 2023). Recurrent cn-LOH aberrations in brain metastases at this chromosomal region suggest an influence on tumor genesis and CRC-based brain metastases formation.

Similarly, a cn-LOH aberration for brain metastases could also be identified for the chromosomal region 19q11-q13.43 (patient 1). This aberration was already detected for brain and liver metastases in our previously published study (Brandt et al. 2023). The CEACAM5 gene (located 19q13.2) belongs to the carcinoembryonic antigen (CEA) family and has been described as a prognostic factor of CRC (Sheng et al. 2025). A higher CEA level is associated with an increased risk for the CRC-based brain metastases development (Xiaohong Yang et al. 2017; Mo et al. 2020; Müller et al. 2021). The identification of prognostic factors may contribute to more specific screenings when the primary tumor appears. This could support an improved assessment of the tumor course and enables possibly the initiation of a patient-specific therapy.

Furthermore, we detected previously not described cn-LOH aberrations, e.g., almost whole chromosome 4 (4p16.3-p11 and 4q12-q35.2). According to Kobayashi et al., loss of chromosome 4q is associated with poor survival in stage III colorectal cancer (Kobayashi et al. 2021). In context of the tumorigenesis, the homologous reduplication (loss of chromosomal sequences followed by reduplication) have been discussed as somatic repair mechanism (Teh et al. 2005; Holland et al. 2007).

Usually, according to literature, cn-LOH aberrations ≥ 5 Mb are considered as reliable alterations. In order to get a deeper insight into tumor genetic events, we have decreased the cut-off level to 3 Mb and identified smaller cn-LOH aberrations for the pulmonary metastases too (chromosomal regions 9p, 10q, and 20q). Recently published results suggest that smaller chromosomal cn-LOH aberrations may also be significant. Therefore, Loh et al. analyzed the genotyping data of blood-derived DNA of the hematopoietic cells and identified cn-LOH aberrations (in a scale of kilobase). These hematopoietic cells with cn-LOHs tended to have a clonal advantages *vs* the homologous counterparts, based on analyses of 29 blood count traits. These results suggest that small cn-LOH aberrations may have a significance for clonally expanding cells and stem cells. The acquisition of cn-LOH mutations results in inherited homozygous alleles. As previously described, these homologous counterparts (cn-LOH segments) could promote the hematopoietic cell expansion, resulting in an increased polygenic drive for blood-cell proliferation (Loh et al. 2020). Therefore, also the initially smaller cn-LOH regions in lung metastases may influence the progression into the cerebellar seeding phenotype.

It has been described that lung metastases are associated with an increased risk for brain metastases formation. The incidence of brain metastases is higher in CRC patients with preceding lung metastases (6.2–22.6%) than with liver metastases (1.3–5%) (Christensen et al. 2016). However, together with the presented results, it remains to identify the potential significance of accumulative processes at chromosomal regions 9p, 10q, and 20q for the tumor progression of colorectal-based lung and brain metastases.

Another very promising approach is provided by the chromosomal region 13q34 (gain detected for brain metastases). Within this region the IRS2 gene is located. Greenberg et al. (2025) recently published their results of the investigations on the CRC-based brain metastases formation and showed that changes in the IRS 2 gene affect the β-catenin pathway. These changes could support brain metastases. Such insights allow the development of further therapeutic approaches contributing to an improved treatment of CRC-based brain metastases. The authors demonstrated, that a therapy applying NT219 (inhibitor of the IRS2 gene product) suppress the brain metastases formation of CRC patients (Greenberg et al. 2025). Nevertheless, further analyses are necessary to enlarge the knowledge of brain metastases formation for further improvement of the therapeutic responsiveness of brain metastases despite the difficult accessibility (blood brain barrier).

Here, we analyzed the genetic profile of CRC-based lung metastases and brain metastases of two patients. Due to the small number of samples, statistical statements are limited. Nevertheless, we decided to apply the CytoScan™ HD array with a better resolution and SNP marker density compared to the OncoScan array knowing about the challenge performing this array on DNA of paraffin-embedded tissue. This array provides more precise results due to the higher density of SNP markers in the chromosomal regions. Our results need to be confirmed in follow-up studies in different CRC patient cohorts (e.g., patients with rectal versus colon cancer, colon cancer patients with and without liver metastases, synchronous versus metachronous brain metastases) – each with a higher number of patients.

## Conclusions

Applying genome-wide high resolution SNP array on colorectal-based lung metastases and brain metastases, we confirmed previously described chromosomal aberrations. The gain of 20q seems to be a repeatedly occurring CNV in the process of colorectal cancer metastasis. Furthermore, we found not previously described chromosomal aberrations (e.g., gain of 1p36.33-p36.32). Cn-LOH regions ≥ 5 Mb were identified exclusively in brain metastases. Applying a reduced cut off level of ≥ 3 Mb, some small cn-LOH regions were found on lung metastases too. These results indicate that cn-LOH regions, considered as accumulative processes, could be a relevant event in the development of colorectal-based metastases.

## Supplementary Information

Below is the link to the electronic supplementary material.

Supplementary Material 1

Supplementary Material 2

Supplementary Material 3

## Data Availability

The datasets generated and/ or analyzed during the current study are available from the corresponding author on reasonable request.

## Abbreviations

CRC: Colorectal carcinoma
BM: Brain metastases
CIMP: CpG island methylator phenotype
cn-LOH: Copy Neutral Loss of Heterozygosity
CGH: Comparative Genomic Hybridization
CNV: Copy Number Variation
