# Supplementary material for: High resolution genome-wide SNP array analyses on matched colorectal-based lung and brain metastases
Source: J Cancer Res Clin Oncol. 2026 Jan 30;152(2):47. doi: 10.1007/s00432-026-06427-7 (PMC12858677; doi:10.1007/s00432-026-06427-7)
Supplement: Supplementary file 1 — Supplementary Material 1 [file 432_2026_6427_MOESM1_ESM.docx]

**Chromosomal aberrant regions - details**

Table with all detected chromosomal aberrant regions, including the potentially affected genes and ISCN-based microarray nomenclature.

| **ID** | **Type** | **Chr.** | **Cytoband**  **Start** | **Cytoband**  **End** | **Size (kbp)** | **Genes** | **Microarray Nomenclature** |
| --- | --- | --- | --- | --- | --- | --- | --- |
| 1  Lung | Gain | 1 | p36.33 | p36.32 | 3,083 | LINC02593, LOC107985728, SAMD11, NOC2L, KLHL17, PLEKHN1, PERM1, HES4, ISG15, AGRN, LOC100288175, LOC105378948, RNF223, C1orf159, LINC01342, MIR200B, MIR200A, MIR429, TTLL10-AS1, TTLL10, TNFRSF18, TNFRSF4, SDF4, B3GALT6, C1QTNF12, UBE2J2, SCNN1D, ACAP3, MIR6726, SNORD167, PUSL1, INTS11, MIR6727, CPTP, TAS1R3, DVL1, MIR6808, MXRA8, AURKAIP1, CCNL2, MRPL20-AS1, MRPL20, MRPL20-DT, ANKRD65, TMEM88B, LINC01770, VWA1, ATAD3C, ATAD3B, ATAD3A, TMEM240, SSU72, FNDC10, LOC105378586, MIB2, MMP23B, CDK11B, SLC35E2B, MMP23A, CDK11A, SLC35E2A, NADK, GNB1, GNB1-DT, CALML6, TMEM52, CFAP74, GABRD, PRKCZ-DT, PRKCZ, PRKCZ-AS1, FAAP20, LOC112268219, SKI, MORN1, LOC100129534, RER1, PEX10, PLCH2, PANK4, HES5, TNFRSF14-AS1, TNFRSF14, LOC100996583, PRXL2B, MMEL1, MMEL1-AS1, TTC34, ACTRT2, PRDM16-DT, PRDM16, MIR4251, ARHGEF16, MEGF6, MIR551A, TPRG1L, WRAP73, TP73, TP73-AS1, CCDC27, SMIM1, LRRC47, CEP104, DFFB, C1orf174, LINC01134, LINC01346, LINC01345 | arr[GRCh38] 1p36.33p36.32(914,087_3,996,949)x3 |
| 1  Lung | Gain | 4 | p16.3 | p16.1 | 8,184 | ZNF721, PIGG, TMEM271, LOC105374338, PDE6B, PDE6B-AS1, ATP5ME, MYL5, SLC49A3, PCGF3, PCGF3-AS1, CPLX1, GAK, TMEM175, DGKQ, SLC26A1, IDUA, FGFRL1, RNF212, LOC105374344, TMED11P, SPON2, LOC100130872, CTBP1-AS, CTBP1, CTBP1-DT, MAEA, UVSSA, NKX1-1, FAM53A, SLBP, TMEM129, TACC3, FGFR3, LETM1, NSD2, SCARNA22, NELFA, MIR943, C4orf48, NAT8L, POLN, HAUS3, MXD4, MIR4800, ZFYVE28, CFAP99, RNF4, FAM193A, TNIP2, SH3BP2, ADD1, MFSD10, NOP14-AS1, NOP14, GRK4, HTT-AS, HTT, MSANTD1, RGS12, HGFAC, DOK7, LRPAP1, LINC00955, LINC02171, ADRA2C, FAM86EP, OTOP1, TMEM128, LYAR, ZBTB49, NSG1, STX18, STX18-IT1, STX18-AS1, SNORD162, LOC101928279, LINC01396, MSX1, LOC101928306, CYTL1, STK32B, LINC01587, EVC2, EVC, CRMP1, MIR378D1, C4orf50, JAKMIP1, LOC128125818, JAKMIP1-DT, WFS1, PPP2R2C, MAN2B2, MRFAP1, LINC02482, LOC93622, LINC02481, S100P, MRFAP1L1, BLOC1S4, KIAA0232, TBC1D14, LOC100129931, CCDC96, TADA2B, GRPEL1, LINC02447, SORCS2, MIR4798, PSAPL1, MIR4274, AFAP1-AS1, AFAP1, LOC389199, ABLIM2, MIR95, SH3TC1, HTRA3, LINC02517, ACOX3, TRMT44, GPR78, CPZ | arr[GRCh38] 4p16.3p16.1(480,646_8,664,923)x3 |
| 1  Lung | Gain | 5 | q35.3 | q35.3 | 3,004 | PHYKPL, COL23A1, CLK4, MSANTD5, ZNF354A, AACSP1, ZNF354B, ZFP2, ZNF454, GRM6, ZNF879, ZNF354C, ADAMTS2, LOC100289470, RUFY1, RUFY1-AS1, HNRNPH1, C5orf60, LOC100502572, LOC105377763, CBY3, CANX, MAML1, LTC4S, MGAT4B, MIR1229, SQSTM1, MRNIP, MRNIP-DT, TBC1D9B, RNF130, MIR340, RASGEF1C, MAPK9, GFPT2, CNOT6, SCGB3A1, FLT4, LINC02222, OR2Y1, MGAT1, HEIH, LINC00847, ZFP62, BTNL8, BTNL3, BTNL9, MIR8089, OR2V1, OR2V2, LINC01962, TRIM7-AS2, TRIM7, MIR4638, TRIM41 | arr[GRCh38] 5q35.3(178,223,694_181,228,055)x3 |
| 1  Lung | Gain | 6 | q27 | q27 | 4,192 | PRR18, SFT2D1, LOC100289495, MPC1, RPS6KA2, RPS6KA2-IT1, MIR1913, RAMACL, RPS6KA2-AS1, RNASET2, MIR3939, CEP43, CCR6, GPR31, LOC105378123, HPAT5, UNC93A, TTLL2, TCP10L3, LOC105378127, LINC02538, LINC02487, LINC01558, LOC441179, AFDN-DT, AFDN, HGC6.3, KIF25-AS1, KIF25, FRMD1, LOC105378137, LOC101929420, DACT2, SMOC2, LOC105378146, LOC101929460, LOC102724357, LINC01615, LINC02544, THBS2-AS1, THBS2, WDR27, C6orf120, PHF10, DYNLT2, ERMARD, LINC00242, LINC00574, LOC100131532, LOC102724511, LOC154449, LOC285804, LINC01624, DLL1, FAM120B, MIR4644 | arr[GRCh38] 6q27(166,292,436_170,484,133)x3 |
| 1  Lung | Gain | 7 | p22.3 | p22.1 | 6,670 | LINC03015, LINC03014, LOC105375115, FAM20C, FOXL3, FOXL3-OT1, LOC442497, LOC116435278, LOC112267991, PDGFA, PDGFA-DT, PRKAR1B, PRKAR1B-AS2, PRKAR1B-AS1, DNAAF5, SUN1, GET4, ADAP1, COX19, C7orf50, CYP2W1, MIR339, GPR146, GPER1, ZFAND2A, ZFAND2A-DT, UNCX, MICALL2, INTS1, MAFK, LOC100128653, TMEM184A, PSMG3, PSMG3-AS1, TFAMP1, LOC105375303, ELFN1, ELFN1-AS1, MAD1L1, MIR4655, SNORA114, MRM2, NUDT1, SNX8, MIR6836, EIF3B, CHST12, GRIFIN, LFNG, MIR4648, BRAT1, IQCE, TTYH3, AMZ1, GNA12, CARD11, LOC100129603, SDK1, LOC105375131, FOXK1, AP5Z1, MIR4656, RADIL, SNORD165, PAPOLB, MMD2, RNF216P1, RBAK, RBAK-RBAKDN, RBAKDN, ZNF890P, WIPI2, SLC29A4, TNRC18, LINC02983, FBXL18, MIR589, LOC221946, ACTB, FSCN1, RNF216, RNF216-IT1, MIR6874, ZNF815P, OCM, CCZ1, RSPH10B, PMS2, AIMP2, SNORA80D, EIF2AK1, ANKRD61, USP42, CYTH3, FAM220A, SAGSIN1, RAC1, DAGLB, KDELR2, GRID2IP, ZDHHC4, INTS15, ZNF853, ZNF316, ZNF12 | arr[GRCh38] 7p22.3p22.1(43,361_6,713,032)x3 |
| 1  Lung | Gain | 7 | q21.3 | q22.1 | 4,565 | LMTK2, BHLHA15, TECPR1, BRI3, BAIAP2L1, NPTX2, TMEM130, TRRAP, MIR3609, SCARNA28, LOC101927550, SMURF1, KPNA7, MYH16, ARPC1A, ARPC1B, PDAP1, BUD31, MIR12119, PTCD1, ATP5MF-PTCD1, CPSF4, ATP5MF, ZNF789, ZNF394, ZKSCAN5, FAM200A, ZNF655, TMEM225B, ZSCAN25, CYP3A5, CYP3A7-CYP3A51P, CYP3A7, CYP3A4, CYP3A43, OR2AE1, TRIM4, GJC3, AZGP1, AZGP1P1, LOC105375423, ZKSCAN1, ZSCAN21, ZNF3, COPS6, MCM7, MIR25, MIR93, MIR106B, AP4M1, TAF6, CNPY4, MBLAC1, LAMTOR4, TRAPPC14, MIR4658, GAL3ST4, GPC2, STAG3, CASTOR3P, PVRIG, SPDYE3, PMS2P1, STAG3L5P-PVRIG2P-PILRB, STAG3L5P, PVRIG2P, MIR6840, PILRB, PILRA, ZCWPW1, MEPCE, PPP1R35, SPACDR, TSC22D4-C7ORF61, TSC22D4, NYAP1, AGFG2, SAP25, LRCH4, FBXO24, PCOLCE-AS1, PCOLCE, MOSPD3, TFR2, ACTL6B, LOC105375429, GNB2, GIGYF1, POP7, EPO, ZAN, EPHB4, SLC12A9, SLC12A9-AS1, TRIP6, MIR6875, SRRT, UFSP1, ACHE, MUC3A, MUC12, MUC12-AS1, MUC17, TRIM56, SERPINE1, AP1S1, MIR4653, VGF, NAT16, MOGAT3, PLOD3, ZNHIT1, CLDN15, FIS1, LNCPRESS1, EMSLR, IFT22, COL26A1, LINC01007, MYL10, CUX1, SH2B2, MIR4285, SPDYE6, LOC100630923, LOC100289561, PRKRIP1, MIR548O, ORAI2, ALKBH4, LRWD1, MIR5090, MIR4467, POLR2J, RASA4B, UPK3BL2, POLR2J3-UPK3BL2, SPDYE2, POLR2J3, RASA4, UPK3BL1, POLR2J2-UPK3BL1, SPDYE2B, POLR2J2 | arr[GRCh38] 7q21.3q22.1(98,112,079_102,676,650)x3 |
| 1  Lung | Gain | 7 | q36.2 | q36.3 | 5,267 | DPP6, LOC101929998, PAXIP1-AS2, PAXIP1, PAXIP1-DT, HTR5A-AS1, HTR5A, INSIG1-DT, INSIG1, BLACE, LINC03010, EN2, CNPY1, RBM33-DT, RBM33, SHH, LOC389602, LOC285889, RNF32-DT, LINC00244, RNF32, LMBR1, NOM1, MNX1, MNX1-AS2, MNX1-AS1, UBE3C, DNAJB6, LOC101927914, PTPRN2, MIR153-2, PTPRN2-AS1, MIR595, LINC01022, MIR5707, NCAPG2, ESYT2, DYNC2I1, LINC00689, VIPR2 | arr[GRCh38] 7q36.2q36.3(153,882,042_159,148,958)x3 |
| 1  Lung | Gain | 8 | q24.23 | q24.3 | 6,755 | FAM135B, COL22A1, KCNK9, TRAPPC9, C8orf17, PEG13, CHRAC1, AGO2, ERICD, PTK2, MIR151A, DENND3-AS1, DENND3, SLC45A4, SLC45A4-AS1, LINC01300, GPR20, PTP4A3, MROH5, C8orf90, MIR1302-7, MIR4472-1, LINC00051, TSNARE1, ADGRB1, ARC, LOC101928087, JRK, PSCA, LY6K, LNCOC1, THEM6, SLURP1, LYPD2, SLURP2, LYNX1-SLURP2, LYNX1, LY6D, GML, CYP11B1, CYP11B2, LY6E-DT, CDC42P3, LY6E, LINC02904, LY6S, LY6L, LY6H, GPIHBP1, ZFP41, GLI4, MINCR, ZNF696, TOP1MT, RHPN1-AS1, RHPN1, MAFA-AS1, MAFA, ZC3H3, SNORD149, GSDMD, LOC100310756, MROH6, NAPRT, EEF1D, TIGD5, PYCR3, GFUS, ZNF623, ZNF707, LINC02878, CCDC166, LOC101928160, MAPK15, FAM83H, MIR4664, IQANK1, LOC105375800, SCRIB, MIR937, PUF60, NRBP2, MIR6845, EPPK1, PLEC, MIR661, PARP10, GRINA, SPATC1, SMPD5, OPLAH, MIR6846, EXOSC4, MIR6847, GPAA1, CYC1, SHARPIN, MAF1, WDR97, HGH1, MROH1, BOP1, MIR7112, SCX, HSF1, DGAT1, MIR6848, SCRT1, TMEM249, FBXL6, SLC52A2, LOC101928902, ADCK5, CPSF1, MIR939, MIR1234, MIR6849, SLC39A4, VPS28, TONSL, TONSL-AS1, MIR6893, ZFTRAF1, TMEM276-ZFTRAF1, MIR10400, TMEM276, KIFC2, FOXH1, PPP1R16A, GPT, MFSD3, RECQL4, LRRC14, LRRC24, C8orf82, ARHGAP39, ZNF251, ZNF34, RPL8, MIR6850, ZNF517, LOC100130027, ZNF7, COMMD5, ZNF250, ZNF16, ZNF252P, TMED10P1, ZNF252P-AS1, C8orf33 | arr[GRCh38] 8q24.23q24.3(138,315,463_145,070,385)x3 |
| 1  Lung | Gain | 9 | q33.3 | q34.3 | 12,482 | MAPKAP1, PBX3-DT, PBX3, LOC101929116, MVB12B, NRON, LMX1B-DT, LMX1B, ZBTB43, ZBTB34, RALGPS1, ANGPTL2, GARNL3, SLC2A8, ZNF79, RPL12, SNORA65, LRSAM1, NIBAN2, STXBP1, MIR3911, CFAP157, PTRH1, TTC16, TOR2A, SH2D3C, MIR3960, MIR2861, CDK9, FPGS, ENG, LOC102723566, AK1, ST6GALNAC4-ST6GALNAC6-AK1, MIR4672, ST6GALNAC6, ST6GALNAC4, PIP5KL1, DPM2, EEIG1, NAIF1, SLC25A25, SLC25A25-AS1, PTGES2, PTGES2-AS1, LCN2, BBLN, CIZ1, DNM1, MIR199B, MIR3154, GOLGA2, SWI5, TRUB2, COQ4, SLC27A4, URM1, MIR219A2, MIR219B, CERCAM, ODF2, ODF2-AS1, GLE1, SPTAN1, DYNC2I2, SET, PKN3, ZDHHC12, ZDHHC12-DT, ZER1, TBC1D13, ENDOG, SPOUT1, KYAT1-SPOUT1, KYAT1, LRRC8A, PHYHD1, DOLK, NUP188, SH3GLB2, MIGA2, DOLPP1, CRAT, PTPA, IER5L, LINC02975, LINC02913, LINC01503, LINC00963, NTMT1, C9orf50, ASB6, PRRX2, PRRX2-AS1, PTGES, TOR1B, TOR1A, C9orf78, USP20, MIR6855, FNBP1, GPR107, GPRACR, NCS1, MIR12126, HMCN2, ASS1, LOC100272217, FUBP3, MIR6856, PRDM12, EXOSC2, ABL1, LOC128092248, QRFP, FIBCD1, LAMC3, AIF1L, NUP214, FAM78A, PLPP7, PRRC2B, SNORD62A, SNORD62B, POMT1, UCK1, PRRT1B, RAPGEF1, MED27, NTNG2, SETX, TTF1, CFAP77, BARHL1, DDX31, GTF3C4, AK8, SPACA9, TSC1, GFI1B, MIR548AW, SNORD141A, LOC105376306, GTF3C5, MIR6877, CEL, CELP, RALGDS, GBGT1, OBP2B, ABO, SURF6, MED22, RPL7A, SNORD24, SNORD36B, SNORD36A, SNORD36C, SURF1, SURF2, SURF4, STKLD1, REXO4, ADAMTS13, CACFD1, SLC2A6, MYMK, ADAMTSL2, FAM163B, DBH, DBH-AS1, SARDH, VAV2, BRD3OS, BRD3, LOC100130548, WDR5, RNU6ATAC, LINC02247, RXRA, MIR4669, COL5A1, COL5A1-AS1, LOC101448202, MIR3689C, MIR3689A, MIR3689D1, MIR3689B, MIR3689D2, MIR3689E, MIR3689F, FCN2, FCN1, OLFM1, LOC401557, LINC02907, PPP1R26-AS1, PPP1R26, PIERCE1, MRPS2, LOC101928525, LCN1, OBP2A, PAEP, LINC01502, LOC102723971, GLT6D1, LCN9, SOHLH1, KCNT1, CAMSAP1, UBAC1, NACC2, TMEM250, LOC107987142, LHX3, QSOX2, CCDC187, DKFZP434A062, GPSM1, DNLZ, CARD9, SNAPC4, ENTR1, PMPCA, INPP5E, SEC16A, C9orf163, NOTCH1, MIR4673, MIR4674, NALT1, LINC01451, HSPC324, EGFL7, MIR126, AGPAT2, DIPK1B, SNHG7, SNORA17B, SNORA17A, LCN10, LCN6, LOC100128593, MIR6722, LCN8, LCN15, TMEM141, CCDC183, CCDC183-AS1, RABL6, MIR4292, AJM1, PHPT1, MAMDC4, EDF1, TRAF2, MIR4479, FBXW5, C8G, LCN12, LINC02692, PTGDS, LCNL1, PAXX, CLIC3, ABCA2, LINC02908, FUT7, NPDC1, ENTPD2, SAPCD2, UAP1L1, MAN1B1-DT, MAN1B1, DPP7, GRIN1, LRRC26, MIR3621, TMEM210, ANAPC2, SSNA1, TPRN, TMEM203, NDOR1, LOC122513141, RNF208, CYSRT1, RNF224, SLC34A3, TUBB4B, FAM166A, STPG3-AS1, STPG3, NELFB, TOR4A, NRARP, EXD3, NOXA1, ENTPD8, NSMF, MIR7114, PNPLA7, MRPL41, DPH7, ZMYND19, ARRDC1, ARRDC1-AS1, EHMT1, LOC651337, MIR602, LOC100133077, CACNA1B, CACNA1B-AS1, LOC101928786 | arr[GRCh38] 9q33.3q34.3(125,608,768_138,090,324)x3 |
| 1  Lung | Gain | 11 | p15.5 | p15.4 | 3,357 | B4GALNT4, PKP3, SIGIRR, ANO9, PTDSS2, RNH1, HRAS, LRRC56, LMNTD2, LMNTD2-AS1, RASSF7, MIR210HG, MIR210, LOC143666, PHRF1, IRF7, CDHR5, SCT, DRD4, DEAF1, TMEM80, EPS8L2, TALDO1, GATD1, GATD1-DT, CEND1, SLC25A22, PIDD1, RPLP2, SNORA52, PNPLA2, CRACR2B, CD151, POLR2L, TSPAN4, CHID1, AP2A2, MUC6, LINC02688, MUC2, MUC5AC, MUC5B, MUC5B-AS1, MIR6744, TOLLIP, TOLLIP-DT, BRSK2, MOB2, DUSP8, KRTAP5-AS1, KRTAP5-1, KRTAP5-2, KRTAP5-3, KRTAP5-4, KRTAP5-5, FAM99A, FAM99B, KRTAP5-6, IFITM10, CTSD, PRADX, SYT8, TNNI2, LSP1, MIR4298, MIR7847, PRR33, LINC01150, TNNT3, MRPL23, SNORD131, MRPL23-AS1, LINC01219, H19, MIR675, IGF2, INS-IGF2, MIR483, IGF2-AS, INS, TH, MIR4686, ASCL2, C11orf21, TSPAN32, CD81-AS1, CD81, TSSC4, TRPM5, KCNQ1, KCNQ1OT1, KCNQ1-AS1, KCNQ1DN, CDKN1C, SLC22A18AS, SLC22A18, PHLDA2, NAP1L4, SNORA54, CARS1, CARS1-AS1, OSBPL5, MRGPRG, MRGPRG-AS1, MRGPRE, ZNF195, TSSC2, OR7E12P, LOC105376526, LOC101927708, XNDC1CP, TRPC2, ART5, ART1, CHRNA10, NUP98 | arr[GRCh38] 11p15.5p15.4(372,356_3,728,953)x3 |
| 1  Lung | Gain | 11 | q13.1 | q13.3 | 5,640 | MARK2, RCOR2, NAA40, COX8A, OTUB1, MACROD1, FLRT1, STIP1, FERMT3, TRPT1, NUDT22, DNAJC4, VEGFB, FKBP2, LOC114841035, PPP1R14B, PPP1R14B-AS1, PLCB3, BAD, GPR137, KCNK4, KCNK4-CATSPERZ, CATSPERZ, ESRRA, TRMT112, PRDX5, CCDC88B, MIR7155, RPS6KA4, MIR1237, LINC02724, SLC22A11, SLC22A12, NRXN2, RASGRP2, PYGM, SF1, MAP4K2, MEN1, CDC42BPG, EHD1, MIR10392, MIR194-2HG, MIR192, MIR194-2, ATG2A, MIR6750, MIR6749, PPP2R5B, GPHA2, MAJIN, BATF2, ARL2, ARL2-SNX15, MIR6879, SNX15, SAC3D1, NAALADL1, CDCA5, ZFPL1, TMEM262, VPS51, TM7SF2, ZNHIT2, FAU, MRPL49, SYVN1, MIR6751, SPDYC, CAPN1-AS1, CAPN1, SLC22A20P, POLA2, CDC42EP2, DPF2, TIGD3, SLC25A45, FRMD8, NEAT1, MIR612, FAUP4, MALAT1, TALAM1, MASCRNA, SCYL1, LTBP3, ZNRD2-DT, ZNRD2, FAM89B, EHBP1L1, KCNK7, MAP3K11, PCNX3, MIR4690, SIPA1, MIR4489, RELA, RELA-DT, KAT5, RNASEH2C, AP5B1, OVOL1, OVOL1-AS1, SNX32, CFL1, MUS81, EFEMP2, CTSW, FIBP, CCDC85B, FOSL1, C11orf68, DRAP1, TSGA10IP, SART1, EIF1AD, BANF1, CST6, CATSPER1, GAL3ST3, SF3B2, SNORD13F, PACS1, KLC2, RAB1B, CNIH2, YIF1A, TMEM151A, CD248, RIN1, BRMS1, B4GAT1, B4GAT1-DT, SLC29A2, NPAS4, MRPL11, PELI3, DPP3-DT, DPP3, BBS1, ZDHHC24, ACTN3, CTSF, CCDC87, CCS, RBM14, RBM14-RBM4, RBM4, RBM4B, SPTBN2, C11orf80, RCE1, PC, LRFN4, MIR3163, C11orf86, SYT12, MIR6860, RHOD, LOC107984341, KDM2A, GRK2, ANKRD13D, SSH3, LOC100130987, POLD4, CLCF1, RAD9A, PPP1CA, TBC1D10C, CARNS1, RPS6KB2, PTPRCAP, CORO1B, GPR152, CABP4, TMEM134, AIP, MIR6752, PITPNM1, CDK2AP2, CABP2, GSTP1, NDUFV1-DT, NDUFV1, DOC2GP, NUDT8, TBX10, ACY3, ALDH3B2, FAM86C2P, LINC02754, UNC93B1, ALDH3B1, NDUFS8, MIR7113, MIR4691, TCIRG1, MIR6753, CHKA, CHKA-DT, KMT5B, C11orf24, LRP5, PPP6R3, GAL, TESMIN, CPT1A, MRPL21, IGHMBP2, MRGPRD, MRGPRF, MRGPRF-AS1, TPCN2, MIR3164, LOC338694, SMIM38, MYEOV, LINC02953, LINC02747, LINC01488 | arr[GRCh38] 11q13.1q13.3(63,858,305_69,498,371)x3 |
| 1  Lung | Gain | 12 | p13.33 | p13.31 | 5,510 | CACNA2D4, LRTM2, LINC00940, DCP1B, CACNA1C-IT2, CACNA1C, CACNA1C-AS4, CACNA1C-IT3, CACNA1C-AS2, CACNA1C-AS1, ITFG2-AS1, FKBP4, ITFG2, NRIP2, TEX52, FOXM1, RHNO1, TULP3, TEAD4, TSPAN9, LOC100128253, LINC02417, PRMT8, THCAT155, CRACR2A, PARP11, PARP11-AS1, CCND2-AS1, CCND2, TIGAR, FGF23, FGF6, C12orf4, RAD51AP1, DYRK4, AKAP3, NDUFA9, GAU1, GALNT8, KCNA6, KCNA1, KCNA5, LINC02443, NTF3, ANO2, VWF, SNORA120, CD9, PLEKHG6, TNFRSF1A, SCNN1A, LTBR, CD27-AS1, CD27, TAPBPL, VAMP1, MRPL51, NCAPD2, SCARNA10, GAPDH, IFFO1, NOP2, CHD4, SCARNA11, LPAR5, ACRBP, ING4, ZNF384, PIANP, COPS7A, MLF2, PTMS, LAG3, CD4, GPR162, P3H3, GNB3, CDCA3, USP5, TPI1, SPSB2, LOC105369632, RPL13P5, DSTNP2, LRRC23, ENO2, ATN1, C12orf57, RNU7-1, PTPN6, MIR200CHG, MIR200C, MIR141, PHB2, SCARNA12, EMG1, LPCAT3, C1S, C1R, C1RL, C1RL-AS1, RBP5, CLSTN3, PEX5 | arr[GRCh38] 12p13.33p13.31(1,792,380_7,302,228)x3 |
| 1  Lung | Gain | 12 | q24.33 | q24.33 | 3,125 | LINC02418, LINC02419, FZD10-AS1, FZD10, PIWIL1, RIMBP2, STX2, RAN, ADGRD1, ADGRD1-AS1, LINC01257, LINC02415, LINC02370, LINC02414, SFSWAP, MMP17, ULK1, PUS1, EP400, SNORA49, EP400P1, DDX51, NOC4L, LINC02361, GALNT9, GALNT9-AS1, LOC101928416, FBRSL1, MIR6763, LRCOL1, P2RX2, POLE, PXMP2, PGAM5, ANKLE2, GOLGA3, CHFR, CHFR-DT, ZNF605, ZNF26, ZNF84-DT, ZNF84, ZNF140, ZNF891, ZNF10 | arr[GRCh38] 12q24.33(130,008,303_133,133,229)x3 |
| 1  Lung | Gain | 13 | q34 | q34 | 4,202 | LINC03032, COL4A1, COL4A2, MIR8073, COL4A2-AS2, COL4A2-AS1, RAB20, NAXD-AS1, NAXD, CARS2, ING1, LINC00567, LOC105370362, PRECSIT, ANKRD10, LINC00431, LINC00368, ARHGEF7-AS2, ARHGEF7, ARHGEF7-AS1, LOC101060553, TEX29, LINC02337, LINC00354, SOX1-OT, SOX1, LOC100506016, LINC01070, LOC101928730, LINC01043, LINC01044, SPACA7, TUBGCP3, ATP11AUN, ATP11A, ATP11A-AS1, MCF2L-AS1, MCF2L, F7, F10, F10-AS1, PROZ, PCID2, CUL4A, MIR8075, LAMP1, GRTP1, GRTP1-AS1, ADPRHL1, DCUN1D2, TMCO3, TFDP1, ATP4B, GRK1, LINC00552, TMEM255B, GAS6-AS1, GAS6, GAS6-DT, LINC00454, LINC00452, SWINGN, C13orf46, RASA3, RASA3-IT1, CFAP97D2, CDC16, MIR548AR, MIR4502, UPF3A | arr[GRCh38] 13q34(110,097,247_114,299,021)x3 |
| 1  Lung | Gain | 16 | p13.3 | p13.3 | 5,096 | POLR3K, SNRNP25, RHBDF1, MPG, NPRL3, HBZ, HBM, HBA2, HBA1, HBQ1, LUC7L, FAM234A, RGS11, ARHGDIG, PDIA2, AXIN1, MRPL28, PGAP6, LOC100134368, NME4, DECR2, RAB11FIP3, LINC00235, CAPN15, MIR5587, MIR3176, PRR35, NHLRC4, PIGQ, RAB40C, WFIKKN1, METTL26, LOC100287175, MCRIP2, LOC105371038, WDR90, RHOT2, RHBDL1, STUB1-DT, STUB1, JMJD8, WDR24, FBXL16, METRN, ANTKMT, CCDC78, HAGHL, CIAO3, MSLN, MIR662, RPUSD1, CHTF18, GNG13, LMF1, LMF1-AS1, CEROX1, SOX8, SSTR5-AS1, SSTR5, C1QTNF8, CACNA1H, TPSG1, TPSB2, TPSAB1, TPSD1, UBE2I, BAIAP3, TSR3, GNPTG, UNKL, UQCC4, PERCC1, CCDC154, CLCN7, PTX4, TELO2, IFT140, TMEM204, LOC105371046, CRAMP1, JPT2, MAPK8IP3, MAPK8IP3-AS1, MIR3177, NME3, MRPS34, EME2, SPSB3, NUBP2, IGFALS, HAGH, FAHD1, MEIOB, LINC00254, LINC02124, HS3ST6, MSRB1, RPL3L, NDUFB10, RPS2, SNORA10, SNORA64, SNHG9, SNORA78, RNF151, TBL3, NOXO1, GFER, SYNGR3, ZNF598, NPW, NHERF2, NTHL1, TSC2, PKD1, MIR1225, PKD1-AS1, MIR6511B1, MIR4516, MIR3180-5, RAB26, SNHG19, SNORD60, TRAF7, CASKIN1, MLST8, BRICD5, PGP, E4F1, DNASE1L2, ECI1, RNPS1, MIR3677HG, MIR3677, MIR940, MIR4717, ABCA3, ABCA17P, CCNF, MIR6767, TEDC2, MIR6768, TEDC2-AS1, NTN3, TBC1D24, ATP6V0C, AMDHD2, CEMP1, MIR3178, PDPK1, LOC652276, FLJ42627, ERVK13-1, KCTD5, PRSS27, SRRM2-AS1, SRRM2, ELOB, PRSS33, SNORA3C, PRSS41, PRSS21, ZG16B, PRSS30P, PRSS22, FLYWCH2, FLYWCH1, KREMEN2, PAQR4, PKMYT1, GREP1, CLDN9, CLDN6, TNFRSF12A, HCFC1R1, THOC6, BICDL2, LOC100128770, MMP25, MMP25-AS1, IL32, ZSCAN10, ZNF205-AS1, ZNF205, ZNF213-AS1, ZNF213, CASP16P, OR1F1, OR1F2P, ZNF200, MEFV, LINC00921, ZNF263, TIGD7, ZNF75A, OR2C1, ZSCAN32, ZNF174, ZNF597, NAA60, MIR6126, C16orf90, CLUAP1, NLRC3, SLX4, DNASE1, TRAP1, CREBBP, LINC02861, ADCY9, SRL, LINC01569, TFAP4, GLIS2, GLIS2-AS1, PAM16, CORO7-PAM16, CORO7, VASN, DNAJA3, NMRAL1, HMOX2, CDIP1, C16orf96, UBALD1, MGRN1, MIR6769A, NUDT16L1, ANKS3, DNAAF8, ZNF500, SEPTIN12, SMIM22, ROGDI, GLYR1, UBN1, PPL, SEC14L5, NAGPA, NAGPA-AS1, C16orf89, ALG1, EEF2KMT | arr[GRCh38] 16p13.3(35,881_5,131,586)x3 |
| 1  Lung | Gain | 16 | p12.1 | p11.2 | 4,761 | C16orf82, LINC02129, KDM8, NSMCE1, NSMCE1-DT, IL4R, IL21R, IL21R-AS1, GTF3C1, KATNIP, LOC100128079, GSG1L, XPO6, SBK1, NPIPB6, EIF3CL, MIR6862-1, NPIPB7, CLN3, APOBR, IL27, NUPR1, SGF29, SULT1A2, SULT1A1, NPIPB8, EIF3C, MIR6862-2, NPIPB9, ATXN2L, TUFM, MIR4721, SH2B1, ATP2A1, ATP2A1-AS1, RABEP2, CD19, NFATC2IP, MIR4517, SPNS1, LAT, RRN3P2, SNX29P2, NPIPB11, SMG1P6, BOLA2-SMG1P6, LOC606724, BOLA2, SLX1B, SLX1B-SULT1A4, SULT1A4, LOC388242, NPIPB12, SMG1P2, MIR3680-2, SPN, QPRT, C16orf54, ZG16, KIF22, MAZ, PRRT2, PAGR1, MVP, CDIPT, CDIPTOSP, SEZ6L2, ASPHD1, KCTD13, TMEM219, TAOK2, HIRIP3, INO80E, DOC2A, C16orf92, TLCD3B, LOC112694756, ALDOA, PPP4C, TBX6, YPEL3, YPEL3-DT, GDPD3, MAPK3, CORO1A, BOLA2B, SLX1A, SLX1A-SULT1A3, SULT1A3, LOC613038, NPIPB13, SMG1P5, CD2BP2, CD2BP2-DT, TBC1D10B, MYL11, SEPTIN1, ZNF48, ZNF771, SNORA80C, DCTPP1, SEPHS2, ITGAL, MIR4518, ZNF768, ZNF747, ZNF747-DT, ZNF764, ZNF688, ZNF785, ZNF689, PRR14, FBRS, LOC730183, SRCAP, SNORA30, TMEM265, PHKG2, CFAP119, RNF40, ZNF629, BCL7C, MIR4519, MIR762HG, MIR762, CTF1, FBXL19-AS1, FBXL19, ORAI3, SETD1A, HSD3B7, STX1B, STX4, ZNF668, ZNF646, PRSS53, VKORC1, BCKDK, KAT8, PRSS8, PRSS36, FUS, PYCARD, PYCARD-AS1, TRIM72, PYDC1, ITGAM, ITGAX, ITGAD, COX6A2, ZNF843, ARMC5, TGFB1I1, SLC5A2, RUSF1, AHSP, LINC02190 | arr[GRCh38] 16p12.1p11.2(26,799,961_31,561,136)x3 |
| 1  Lung | Gain | 16 | q23.3 | q24.3 | 6,139 | MLYCD, OSGIN1, NECAB2, SLC38A8, MBTPS1, HSDL1, DNAAF1, TAF1C, ADAD2, LOC654780, KCNG4, WFDC1, ATP2C2, ATP2C2-AS1, MEAK7, COTL1, KLHL36, USP10, CRISPLD2, ZDHHC7, KIAA0513, CIBAR2, LINC02139, MIR12128, LINC00311, MIR5093, GSE1, GINS2, C16orf74, MIR1910, EMC8, LOC101928557, COX4I1, IRF8, MIR6774, LINC01082, LINC01081, LINC02135, LINC00917, FENDRR, FOXF1, MTHFSD, FLJ30679, FOXC2-AS1, FOXC2, FOXL1, LINC02189, LINC02188, LINC02181, LOC101928708, LOC101928682, C16orf95, C16orf95-DT, FBXO31, MAP1LC3B, ZCCHC14, ZCCHC14-DT, JPH3, KLHDC4, LOC102724467, SLC7A5, MIR6775, MIR11401, CA5A, BANP, LOC400553, LINC02182, LOC107984862, ZNF469, ZFPM1, MIR5189, ZFPM1-AS1, ZC3H18-AS1, ZC3H18, IL17C, CYBA, MVD, SNAI3-AS1, SNAI3, RNF166, CTU2, PIEZO1, MIR4722, LOC100289580, LOC339059, CDT1, APRT, GALNS, TRAPPC2L, PABPN1L, CBFA2T3, LOC101927793, LOC100129697, ACSF3, LINC00304, LINC02138, CDH15, SLC22A31, ZNF778, ANKRD11, LOC105371414, LOC128462377, LOC100287036, LOC101927817, SPG7, RPL13, SNORD68, CPNE7, DPEP1, CHMP1A, SPATA33, LINC02166, CDK10, SPATA2L, VPS9D1, VPS9D1-AS1, ZNF276, FANCA, SPIRE2, TCF25, MC1R, TUBB3, DEF8, SNORA119, CENPBD1P, AFG3L1P, DBNDD1, GAS8, GAS8-AS1 | arr[GRCh38] 16q23.3q24.3(83,892,763_90,032,002)x3 |
| 1  Lung | Gain | 17 | q24.3 | q25.3 | 10,694 | LINC00673, LINC00511, SLC39A11, SSTR2, COG1, FAM104A, C17orf80, CPSF4L, CDC42EP4, SDK2, LOC101928251, LOC100134391, LINC00469, LINC02092, LINC02074, RPL38, MGC16275, TTYH2, DNAI2, KIF19, BTBD17, GPR142, GPRC5C, CD300A, CD300LB, CD300C, CD300H, CD300LD, CD300LD-AS1, LOC101928343, CD300E, RAB37, CD300LF, MIR3615, NHERF1, NAT9, TMEM104, GRIN2C, FDXR, FADS6, USH1G, OTOP2, OTOP3, HID1, HID1-AS1, CDR2L, MRPL58, KCTD2, ATP5PD, SLC16A5, ARMC7, NT5C, JPT1, SUMO2, NUP85, GGA3, MRPS7, MIF4GD, MIF4GD-DT, SLC25A19, GRB2, MIR3678, TMEM94, MIR6785, CASKIN2, TSEN54, LLGL2, MYO15B, RECQL5, SMIM5, SMIM6, SAP30BP, ITGB4, GALK1, H3-3B, MIR4738, UNK, UNC13D, WBP2, TRIM47, TRIM65, MRPL38, FBF1, ACOX1, TEN1-CDK3, TEN1, CDK3, EVPL, SRP68, GALR2, ZACN, EXOC7, MIR6868, FOXJ1, RNF157-AS1, RNF157, UBALD2, QRICH2, PRPSAP1, SPHK1, UBE2O, AANAT, RHBDF2, CYGB, PRCD, SNHG16, SNORD1C, SNORD1B, SNORD1A, ST6GALNAC2, LOC122455342, ST6GALNAC1, LOC105274304, MXRA7, JMJD6, METTL23, SRSF2, MIR636, MFSD11, LINC02080, LINC00868, MGAT5B, LOC105371899, SNHG20, SEC14L1, SCARNA16, MIR6516, SEPTIN9-DT, SEPTIN9, MIR4316, LOC400622, LOC100507351, LINC01987, LINC01973, TNRC6C, TMC6, TMC8, C17orf99, SYNGR2, TK1, AFMID, BIRC5, TMEM235, LOC105371910, LINC01993, SOCS3, SOCS3-DT, PGS1, DNAH17, DNAH17-AS1, SCAT1, CYTH1, USP36, TIMP2, CEP295NL, LGALS3BP, CANT1, C1QTNF1-AS1, C1QTNF1, ENGASE, RBFOX3, MIR4739, LINC02078, ENPP7, CBX2, CBX8, LINC01977, CBX4, LINC01979, LINC01978, TBC1D16, CCDC40, MIR1268B, GAA, EIF4A3, CARD14, SGSH, SLC26A11, RNF213, RNF213-AS1, ENDOV, MIR4730, NPTX1, RPTOR, LOC101928855, LOC400627, CHMP6, BAIAP2-DT, BAIAP2, AATK, MIR657, MIR3065, MIR338, MIR1250, PVALEF, CEP131, TEPSIN, LOC105371925, NDUFAF8, SLC38A10, LINC00482, TMEM105, LINC03048, BAHCC1, MIR4740, MIR3186, LINC01971, ACTG1, FSCN2, FAAP100, NPLOC4, TSPAN10, PDE6G, OXLD1, CCDC137, ARL16, HGS, MIR6786, MRPL12, SLC25A10, GCGR, MCRIP1, PPP1R27, P4HB, ARHGDIA, ALYREF, ANAPC11, PCYT2, NPB, SIRT7, MAFG, MILIP, PYCR1, MYADML2, NOTUM, ASPSCR1, CENPX, LRRC45, RAC3, DCXR, DCXR-DT, RFNG, GPS1, DUS1L, FASN, SNORD134, CCDC57, SLC16A3, MIR6787, CSNK1D, LINC01970, CD7, SECTM1, TEX19, UTS2R, OGFOD3, HEXD, HEXD-IT1, CYBC1, NARF-AS2, NARF, FOXK2, WDR45B, RAB40B, MIR4525, LOC101929552, FN3KRP, FN3K, TBCD, ZNF750, B3GNTL1, METRNL | arr[GRCh38] 17q24.3q25.3(72,389,792_83,083,947)x3 |
| 1  Lung | Gain | 19 | p13.3 | p13.3 | 4,845 | TPGS1, CDC34, GZMM, BSG-AS1, BSG, HCN2, POLRMT, FGF22, RNF126, FSTL3, PRSS57, PALM, MISP, PTBP1, MIR4745, PLPPR3, MIR3187, AZU1, PRTN3, ELANE, CFD, MED16, RNU6-9, R3HDM4, KISS1R, ARID3A, WDR18, GRIN3B, TMEM259, RNU6-2, CNN2, ABCA7, ARHGAP45, POLR2E, GPX4, SBNO2, STK11, CBARP, ATP5F1D, MIDN, CIRBP-AS1, CIRBP, FAM174C, EFNA2, PWWP3A, NDUFS7, GAMT, DAZAP1, RPS15, APC2, C19orf25, PCSK4, REEP6, ADAMTSL5, PLK5, MEX3D, MBD3, UQCR11, TCF3, ONECUT3, ATP8B3, REXO1, MIR1909, LOC100288123, KLF16, ABHD17A, ADAT3, SCAMP4, CSNK1G2, CSNK1G2-AS1, BTBD2, MKNK2, MOB3A, IZUMO4, AP3D1, DOT1L, PLEKHJ1, MIR1227, MIR6789, SF3A2, AMH, MIR4321, JSRP1, OAZ1, PEAK3, LINGO3, LSM7, SPPL2B, TMPRSS9, TIMM13, LMNB2, MIR7108, LINC01775, GADD45B, GNG7, MIR7850, DIRAS1, SLC39A3, SGTA, THOP1, ZNF554, ZNF555, ZNF556, ZNF57, ZNF77, TLE6, TLE2, TLE5, GNA11, GNA15, GNA15-DT, S1PR4, NCLN, CELF5, NFIC, SMIM24, SMIM44, DOHH, FZR1, TEKTIP1, MFSD12, HMG20B, GIPC3, TBXA2R, CACTIN-AS1, CACTIN, PIP5K1C, TJP3, APBA3, MRPL54, RAX2, MATK, ZFR2, ATCAY, NMRK2, DAPK3, MIR637, EEF2, SNORD37, PIAS4, ZBTB7A, MAP2K2, CREB3L3, SIRT6, ANKRD24, EBI3, YJU2, SHD, TMIGD2, FSD1, STAP2, MPND, SH3GL1, CHAF1A, UBXN6, MIR4746, HDGFL2, PLIN4, PLIN5, LRG1, SEMA6B, TNFAIP8L1, MYDGF, DPP9, DPP9-AS1, MIR7-3HG, MIR7-3, FEM1A, TICAM1, PLIN3, ARRDC5, UHRF1, MIR4747, KDM4B, PTPRS | arr[GRCh38] 19p13.3(507,877_5,352,713)x3 |
| 1  Lung | Gain | 20 | q13.33 | q13.33 | 3,382 | LINC01718, CDH4, LOC100128310, MIR1257, TAF4, MIR3195, LSM14B, PSMA7, SS18L1, MTG2, HRH3, LOC105369209, OSBPL2, ADRM1, LAMA5, MIR4758, LAMA5-AS1, RPS21, CABLES2, RBBP8NL, GATA5, CRMA, MIR1-1HG, MIR1-1, MIR133A2, LINC02970, SLCO4A1, SLCO4A1-AS2, SLCO4A1-AS1, NTSR1, LINC00659, MRGBP, OGFR-AS1, OGFR, COL9A3, TCFL5, DIDO1, SNORA117, GID8, SLC17A9, BHLHE23, LINC01749, LINC00029, LINC01056, HAR1B, HAR1A, MIR124-3, YTHDF1, BIRC7, MIR3196, NKAIN4, FLJ16779, ARFGAP1, MIR4326, COL20A1, CHRNA4, LOC100130587, KCNQ2, EEF1A2, PPDPF, PTK6, SRMS, FNDC11, HELZ2, GMEB2, MHENCR, STMN3, RTEL1, RTEL1-TNFRSF6B, TNFRSF6B, ARFRP1, ZGPAT, LIME1, SLC2A4RG, ZBTB46, ZBTB46-AS1, LOC112268269, ABHD16B, TPD52L2, DNAJC5, MIR941-1, MIR941-2, MIR941-3, MIR941-4, MIR941-5, UCKL1, MIR1914, MIR647, UCKL1-AS1, ZNF512B, SAMD10, PRPF6, C20orf204, SOX18, TCEA2, RGS19, MIR6813, OPRL1, LKAAEAR1, NPBWR2, MYT1 | arr[GRCh38] 20q13.33(60,862,643_64,245,018)x3 |
| 1  Lung | Loss | X | p22.33 | p11.1 | 55,761 | CD99, XG, GYG2, ARSD, ARSD-AS1, ARSL, ARSH, ARSF, LINC01546, MXRA5, SNORA48B, PRKX, PRKX-AS1, LOC389906, FAM239A, FAM239B, LOC101928201, NLGN4X, LOC105373156, MIR4770, VCX3A, PUDP, STS, MIR4767, VCX, PNPLA4, MIR651, VCX2, VCX3B, ANOS1, FAM9A, FAM9B, TBL1X, GPR143, SHROOM2, CLDN34, WWC3, CLCN4, MID1, HCCS, ARHGAP6, AMELX, MIR548AX, MSL3, FRMPD4, FRMPD4-AS1, PRPS2, TLR7, TLR8-AS1, TLR8, TMSB4X, FAM9C, LOC105373133, LINC02154, GS1-600G8.3, ATXN3L, LINC01203, EGFL6, MIR6086, LOC107985657, TCEANC, RAB9A, TRAPPC2, OFD1, GPM6B, GEMIN8, UBE2E4P, GLRA2, FANCB, MOSPD2, ASB9, ASB11, PIGA, PIR-FIGF, VEGFD, PIR, BMX, ACE2, ACE2-DT, CLTRN, CA5BP1, CA5BP1-CA5B, CA5B, INE2, ZRSR2, AP1S2, GRPR, MAGEB17, CTPS2, MIR548AM, S100G, SYAP1, TXLNG, RBBP7, REPS2, NHS, MIR4768, LOC101928389, NHS-AS1, SCML1, RAI2, LINC01456, BEND2, SCML2, CDKL5, RS1, PPEF1, PPEF1-AS1, PHKA2-AS1, PHKA2, ADGRG2, PDHA1, MAP3K15, SH3KBP1, BCLAF3, LOC729609, MAP7D2, MIR23C, EIF1AX, SCARNA9L, EIF1AX-AS1, RPS6KA3, CNKSR2, KLHL34, SMPX, MBTPS2, YY2, SMS, PHEX, PHEX-AS1, PTCHD1-AS, CBLL2, DDX53, PTCHD1, PRDX4, ACOT9, SAT1-DT, SAT1, LOC127933115, APOO, CXorf58, KLHL15, EIF2S3, ZFX-AS1, ZFX, SUPT20HL2, SUPT20HL1, PDK3, PCYT1B, PCYT1B-AS1, POLA1, SCARNA23, ARX, MAGEB18, MAGEB6B, MAGEB6, MAGEB5, VENTXP1, PPP4R3C, DCAF8L2, MAGEB10, DCAF8L1, MIR6134, IL1RAPL1, MIR4666B, MAGEB2, MAGEB3, MAGEB4, MAGEB1, NR0B1, TASL, GK, GK-AS1, TAB3, TAB3-AS1, FTHL17, DMD, MIR3915, MIR548F5, FAM47A, TMEM47, FAM47B, MAGEB16, CFAP47, LOC101928627, FAM47C, FTHL18P, PRRG1, LANCL3, XK, CYBB, DYNLT3, H2AP, H2AL3, SYTL5, MIR548AJ2, SRPX, RPGR, OTC, TSPAN7, MID1IP1-AS1, MID1IP1, LINC01281, LINC01282, MIR3937, MIR1587, BCOR, LOC107985687, LINC03099, ATP6AP2, MPC1L, CXorf38, MED14, MED14OS, TNIP2P1, USP9X, LINC02601, DDX3X, NYX, CASK, GPR34, GPR82, PPP1R2C, PINCR, MAOA, MAOB, NDP, NDP-AS1, EFHC2, FUNDC1, DUSP21, KDM6A, DIPK2B, LINC01204, MFFP3, MIR222HG, MIR221, MIR222, LINC02595, LINC01186, KRBOX4, ZNF674, ZNF674-AS1, CHST7, SLC9A7, RP2, LINC01545, JADE3, RGN, NDUFB11, RBM10, UBA1, INE1, CDK16, USP11, ZNF157, SNORA11C, ZNF41, LINC01560, ARAF, SYN1, TIMP1, MIR4769, CFP, ELK1, UXT, UXT-AS1, CXXC1P1, ZNF81, ZNF182, SPACA5, ZNF630-AS1, ZNF630, SSX6P, SPACA5B, SSX5, SSX1, SSX9P, SSX3, SSX4, SSX4B, SLC38A5, FTSJ1, PORCN-DT, PORCN, EBP, TBC1D25, RBM3, WDR13, WAS, SUV39H1, GLOD5, GATA1, HDAC6, ERAS, PCSK1N, TIMM17B, PQBP1, SLC35A2, PIM2, OTUD5, KCND1, GRIPAP1, TFE3, CCDC120, PRAF2, WDR45, GPKOW, MAGIX, PLP2, PRICKLE3, SYP, SYP-AS1, CACNA1F, CCDC22, FOXP3, FLICR, PPP1R3F, GAGE10, GAGE12J, GAGE13, GAGE12B, GAGE12C, GAGE12D, GAGE12E, GAGE12F, GAGE12G, GAGE12H, GAGE2A, GAGE1, PAGE1, PAGE4, USP27X-DT, USP27X, CLCN5, MIR532, MIR188, MIR500A, MIR362, MIR501, MIR500B, MIR660, MIR502, AKAP4, CCNB3, DGKK, SHROOM4, BMP15, LINC01284, NUDT10, EZHIP, NUDT11, LINC01496, CENPVL3, CENPVL2, CENPVL1, GSPT2, MAGED1, MAGED4B, SNORA11E, MAGED4, SNORA11D, MIR8088, XAGE2, XAGE1A, XAGE1B, SSX8P, SSX7, SSX2, SSX2B, SPANXN5, XAGE5, XAGE3, FAM156B, FAM156A, GPR173, TSPYL2, KANTR, KDM5C, MIR6895, MIR6894, IQSEC2, SMC1A, MIR6857, RIBC1, HSD17B10, HUWE1, MIR98, MIRLET7F2, PHF8, FAM120C, WNK3, TSR2, FGD1, GNL3L, ITIH6, MAGED2, SNORA11, TRO, SNORA11G, PFKFB1, APEX2, ALAS2, PAGE2B, PAGE2, FAM104B, SNORA109, PAGE5, PAGE3, LOC100421746, MIR4536-1, MIR4536-2, MAGEH1, USP51, FOXR2, RRAGB, KLF8, UBQLN2, NBDY, UQCRBP1, SPIN3, SPIN2B, SPIN2A, FAAH2, ZXDB, NLRP2B, ZXDA | arr[GRCh38] Xp22.33p11.1(2,740,114_58,500,731)x1 |
| 1  Lung | Loss | X | q11.1 | q28 | 93,250 | SPIN4-AS1, SPIN4, LINC01278, ARHGEF9, ARHGEF9-IT1, LOC128031833, MIR1468, AMER1, ASB12, MTMR8, PFN5P, ZC4H2, ZC3H12B, LAS1L, FRMD8P1, MSN, MIR223HG, MIR223, VSIG4, HEPH, EDA2R, AR, OPHN1, YIPF6, STARD8, EFNB1, PJA1, LINC00269, NALF2, EDA, MIR676, AWAT2, OTUD6A, IGBP1, DGAT2L6, AWAT1, P2RY4, ARR3, RAB41, PDZD11, KIF4A, GDPD2, LOC105373244, DLG3, DLG3-AS1, TEX11, SNORD3E, SLC7A3, SNX12, FOXO4, CXorf65, IL2RG, MED12, NLGN3, GJB1, ZMYM3, NONO, ITGB1BP2, TAF1, INGX, OGT, GCNA, CXCR3, LOC100129291, LOC101059915, LINC00891, CXorf49, CXorf49B, LOC100132741, NHSL2, RPS26P11, RTL5, PIN4, ERCC6L, RPS4X, CITED1, HDAC8, PHKA1, PHKA1-AS1, DMRTC1B, FAM226B, FAM236B, FAM236D, DMRTC1, FAM236C, FAM236A, FAM226A, PABPC1L2B-AS1, PABPC1L2B, PABPC1L2A, NAP1L6P, NAP1L2, CDX4, MAP2K4P1, CHIC1, TSIX, XIST, JPX, FTX, MIR421, MIR374B, MIR374C, MIR545, MIR374A, ZCCHC13, SLC16A2, RLIM, NEXMIF, ABCB7, UPRT, ZDHHC15, TTC3P1, MAGEE2, PBDC1, MAGEE1, MIR325HG, MIR384, MIR325, FGF16, ATRX, MAGT1, COX7B, ATP7A, PGAM4, PGK1, TAF9B, CYSLTR1, RTL3, LPAR4, MIR4328, P2RY10, GPR174, ITM2A, TBX22, CHMP1B2P, TENT5D, BRWD3, HMGN5, SH3BGRL, EIF3JP1, POU3F4, CYLC1, RPS6KA6, MIR548I4, HDX, UBE2DNL, APOOL, SATL1, LOC101928128, ZNF711, POF1B, MIR1321, CHM, MIR361, DACH2, KLHL4, CPXCR1, TGIF2LX, PABPC5-AS1, PABPC5, PCDH11X, NAP1L3, FAM133A, MIR548M, BRDTP1, DIAPH2, RPA4, DIAPH2-AS1, PCDH19, TNMD, TSPAN6, SRPX2, SYTL4, CSTF2, NOX1, XKRX, ARL13A, TRMT2B, TMEM35A, CENPI, DRP2, TAF7L, TIMM8A, BTK, RPL36A, RPL36A-HNRNPH2, GLA, HNRNPH2, ARMCX4, ARMCX1, ARMCX6, ARMCX3, ARMCX2, NXF5, ZMAT1, TCEAL2, TCEAL6, BEX5, TCP11X1, NXF2, NXF2B, TCP11X2, TMSB15A, NXF4, ARMCX5, ARMCX5-GPRASP2, GPRASP1, GPRASP2, GPRASP3, LINC00630, RAB40AL, BEX1, NXF3, BEX4, TCEAL8, TCEAL5, BEX2, TCEAL7, TCEAL9, BEX3, RAB40A, LINC02589, TCEAL4, TCEAL3, TCEAL1, MORF4L2, MORF4L2-AS1, GLRA4, TMEM31, RAB9B, PLP1, TMSB15B-AS1, TMSB15B, H2BW4P, H2BW1, H2BW2, H2BW3P, TMSB15C, SLC25A53, ZCCHC18, LOC286437, FAM199X, ESX1, IL1RAPL2, TEX13A, NRK, SERPINA7, PWWP3B, RADX, MIR548AN, RNF128, TBC1D8B, RIPPLY1, CLDN2, MORC4, RBM41, NUP62CL, DNAAF6, FRMPD3, FRMPD3-AS1, PRPS1, TSC22D3, NCBP2L, MID2, LOC101928335, LOC112267910, TEX13B, VSIG1, PSMD10, ATG4A, COL4A6, COL4A5, IRS4, IRS4-AS1, GUCY2F, NXT2, KCNE5, ACSL4, LOC105373311, TMEM164, MIR652, MIR3978, AMMECR1, SNORD96B, GNG5B, RTL9, TDGF1P3, CHRDL1, PAK3, CAPN6, DCX, SERTM2, ALG13, TRPC5, TRPC5OS, RTL4, LHFPL1, AMOT, MIR4329, LOC101928437, XACT, HTR2C, SNORA35, MIR764, MIR1912, MIR1264, MIR1298, MIR1911, MIR448, IL13RA2, LRCH2, SNORA35B, RBMXL3, LUZP4, PLS3-AS1, PLS3, DANT2, DANT1, AGTR2, SLC6A14, CT83, LOC100126447, KLHL13, WDR44, MIR1277, DOCK11, IL13RA1, ZCCHC12, LINC01285, LONRF3, CT47C1, KIAA1210, PGRMC1, AKAP17BP, LINC03098, SLC25A43, SLC25A5-AS1, SLC25A5, STEEP1, UBE2A, NKRF, SEPTIN6, MIR766, SOWAHD, RPL39, SNORA69, UPF3B, RNF113A, NDUFA1, AKAP14, NKAP, RHOXF1P1, RHOXF1-AS1, RHOXF2B, RHOXF1, LINC01402, RHOXF2, NKAPP1, ZBTB33, TMEM255A, ATP1B4, LAMP2, CUL4B, MCTS1, C1GALT1C1, CT47B1, CT47A12, CT47A11, CT47A10, CT47A9, CT47A8, CT47A7, CT47A6, CT47A5, CT47A4, CT47A3, CT47A2, CT47A1, GLUD2, MIR3672, GRIA3, THOC2, XIAP, STAG2-AS1, STAG2, TEX13D, SH2D1A, TENM1, TEX13C, LOC101928495, DCAF12L2, DCAF12L1, PRR32, ACTRT1, SMARCA1, OCRL, APLN, XPNPEP2, SASH3, ZDHHC9, UTP14A, BCORL1, ELF4, AIFM1, RAB33A, ZNF280C, SLC25A14, GPR119, RBMX2, DENND10P1, ENOX2, ENOX2-AS1, LINC01201, ARHGAP36, IGSF1, OR13H1, FIRRE, STK26, FRMD7, RAP2C, RAP2C-AS1, MBNL3, HS6ST2, HS6ST2-AS1, USP26, TFDP3, GPC4, GPC3, GPC3-AS1, MIR363, MIR92A2, MIR19B2, MIR20B, MIR18B, MIR106A, CCDC160, PHF6, HPRT1, MIR450B, MIR450A1, MIR450A2, MIR542, MIR503HG, MIR503, MIR424, LINC00629, PLAC1, PABIR2, PABIR3, MOSPD1, LINC02243, SMIM10, RTL8B, RTL8C, RTL8A, SMIM10L2B, ETDB, SMIM10L2B-AS1, CT55, ZNF75D, ETDA, ETDC, ZNF449, LOC100506790, SMIM10L2A, INTS6L-AS1, INTS6L, SAGE2P, CT45A1, CT45A3, CT45A5, CT45A6, CT45A2, CT45A7, CT45A8, CT45A9, CT45A10, SAGE1, MMGT1, SLC9A6, FHL1, MAP7D3, ADGRG4, BRS3, HTATSF1, VGLL1, MIR934, LINC00892, CD40LG, ARHGEF6, RBMX, SNORD61, GPR101, ZIC3, FGF13, MIR504, FGF13-AS1, SRD5A1P1, F9, MCF2, ATP11C, MIR505, CXorf66, LOC728660, HAPSTR2, SOX3, LINC00632, MIR320D2, SPANXB1, LDOC1, SPANXC, SPANXA2-OT1, SPANXA1, SPANXA2, LOC645188, SPANXD, MAGEC3, MAGEC1, MAGEC2, SPANXN4, SPANXN3, SLITRK4, SPANXN2, UBE2NL, SPANXN1, SLITRK2, MIR892C, MIR890, MIR888, MIR892A, MIR892B, MIR891B, MIR891A, CXorf51B, CXorf51A, MIR513C, MIR513B, MIR513A1, MIR513A2, MIR506, MIR507, MIR508, MIR514B, MIR509-2, MIR509-3, MIR509-1, MIR510, MIR514A1, MIR514A2, MIR514A3, FMR1-AS1, FMR1, FMR1NB, AFF2, LOC122319696, IDS, EOLA1-DT, EOLA1, HSFX3, MAGEA9B, HSFX2, TMEM185A, MAGEA11, HSFX1, MAGEA9, LINC00850, MAGEA8-AS1, MAGEA8, HSFX4, EOLA2, EOLA2-DT, MIR2114, MAMLD1, MTM1, MTMR1, CD99L2, HMGB3, MIR4330, GPR50-AS1, GPR50, VMA21, LOC105377213, PASD1, PRRG3, FATE1, CNGA2, MAGEA4-AS1, MAGEA4, GABRE, MIR224, MIR452, MAGEA5P, LOC100533997, MAGEA10, GABRA3, MIR105-1, MIR767, MIR105-2, GABRQ, MAGEA3, CSAG2, MAGEA2B, CSAG1, MAGEA12, CSAG4, MAGEA2, CSAG3, MAGEA6, CETN2, NSDHL, ZNF185, PNMA5, PNMA3, PNMA6A, MAGEA1, PWWP4, PNMA6F, ZNF275, MIR12129, LOC105373378, PNMA6E, ZFP92, TREX2, HAUS7, ECMXP, BGN, ATP2B3, CCNQ, LOC105373383, DUSP9, PNCK, SLC6A8, BCAP31, ABCD1, PLXNB3, SRPK3, IDH3G, SSR4, PDZD4, L1CAM, L1CAM-AS1, AVPR2, ARHGAP4, NAA10, RENBP, HCFC1, HCFC1-AS1, TMEM187, MIR3202-1, MIR3202-2, IRAK1, MIR718, MECP2, OPN1LW, OPN1MW, OPN1MW2, OPN1MW3, TEX28, TKTL1, FLNA, EMD, RPL10, SNORA70, DNASE1L1, TAFAZZIN, ATP6AP1-DT, ATP6AP1, GDI1, FAM50A, MIR6858, PLXNA3, LAGE3, UBL4A, SLC10A3, FAM3A, G6PD, IKBKG, FAM223A, CTAG1A, CTAG1B, FAM223B, CTAG2, GAB3, DKC1, SNORA36A, MIR664B, SNORA56, MPP1, SMIM9, F8, H2AB1, F8A1, MIR1184-1, FUNDC2, CMC4, MTCP1, BRCC3, VBP1, RAB39B, CLIC2, LOC101927830, H2AB2, F8A2, MIR1184-2, F8A3, MIR1184-3, H2AB3, TMLHE-AS1, TMLHE, SPRY3, VAMP7, IL9R | arr[GRCh38] Xq11.1q28(62,754,016_156,004,066)x1 |
| 1  Lung | Loss | Y | p11.2 | q12 | 23,854 | SRY, RPS4Y1, ZFY, ZFY-AS1, LINC00278, TGIF2LY, MIR9985, PCDH11Y, TTTY23B, TSPY2, FAM197Y9, LINC00280, TTTY1B, TTTY2B, TTTY21B, TTTY7, TTTY8B, AMELY, TBL1Y, PRKY, TTTY16, TTTY12, LINC00279, TTTY18, TTTY19, TTTY11, RBMY1A3P, TTTY20, TSPY4, FAM197Y8, TSPY8, FAM197Y7, FAM197Y6, TSPY3, FAM197Y5, TSPY1, FAM197Y4, TSPY9, FAM197Y3, FAM197Y2, TSPY10, RBMY3AP, TTTY8, TTTY7B, TTTY21, TTTY2, TTTY1, TTTY22, TTTY23, GYG2P1, TTTY15, USP9Y, DDX3Y, UTY, MIR12120, TMSB4Y, VCY, VCY1B, NLGN4Y, NLGN4Y-AS1, FAM41AY1, FAM224B, XKRY, CDY2B, CDY2A, XKRYP7, FAM224A, FAM41AY2, HSFY1, TTTY9B, TTTY9A, HSFY2, TTTY14, BCORP1, TXLNGY, KDM5D, TTTY10, EIF1AY, RPS4Y2, PRORY, RBMY2EP, RBMY1B, RBMY1A1, TTTY13, RBMY1D, RBMY1E, PRY2, TTTY6B, RBMY1F, TTTY5, RBMY2FP, LOC100652931, RBMY1J, LOC101929148, TTTY6, PRY, TTTY17A, TTTY4, BPY2, DAZ1, DAZ2, TTTY3B, CDY1B, GOLGA2P2Y, TTTY17B, TTTY4B, BPY2B, DAZ3, DAZ4, BPY2C, TTTY4C, TTTY17C, SEPTIN14P23, GOLGA2P3Y, CSPG4P1Y, CDY1, TTTY3 | arr[GRCh38] Yp11.2q12(2,782,100_26,636,252)x1 |
| 1  Brain | cn-LOH | 3 | p21.31 | p21.1 | 6,017 | SMARCC1, SNORD146, DHX30, MIR1226, MAP4, CDC25A, MIR4443, CAMP, ZNF589, FCF1P2, NME6, SPINK8, MIR2115, FBXW12, PLXNB1, CCDC51, TMA7, ATRIP, ATRIP-TREX1, TREX1, SHISA5, PFKFB4, MIR6823, UCN2, COL7A1, MIR711, UQCRC1, SNORA94, TMEM89, SLC26A6, MIR6824, CELSR3, MIR4793, LINC02585, NCKIPSD, IP6K2, PRKAR2A, PRKAR2A-AS1, SLC25A20, ARIH2OS, ARIH2, P4HTM, WDR6, DALRD3, MIR425, NDUFAF3, MIR191, IMPDH2, QRICH1, QARS1, MIR6890, USP19, LAMB2, LAMB2P1, CCDC71, KLHDC8B, C3orf84, IHO1, C3orf62, MIR4271, USP4, GPX1, RHOA, TCTA, AMT, NICN1, DAG1, BSN-DT, BSN, APEH, MST1, RNF123, AMIGO3, GMPPB, IP6K1, CDHR4, INKA1, UBA7, MIR5193, TRAIP, CAMKV, MST1R, MON1A, RBM6, RBM5, RBM5-AS1, SEMA3F-AS1, SEMA3F, GNAT1, SLC38A3, GNAI2, MIR5787, SEMA3B-AS1, SEMA3B, MIR6872, LSMEM2, IFRD2, HYAL3, NAA80, HYAL1, HYAL2, TUSC2, RASSF1, RASSF1-AS1, ZMYND10, NPRL2, CYB561D2, LOC127898564, TMEM115, CACNA2D2, LOC101928965, C3orf18, HEMK1, CISH, MAPKAPK3, LINC02019, DOCK3, MIR4787, MANF, RBM15B, DCAF1, RAD54L2, TEX264, GRM2, IQCF6, IQCF4P, IQCF3, IQCF2, IQCF5-AS1, IQCF5, IQCF1, RRP9, PARP3, GPR62, PCBP4, ABHD14B, ABHD14A, ABHD14A-ACY1, ACY1, RPL29, DUSP7, LINC00696, POC1A, ALAS1, TLR9, TWF2, TWF2-DT, PPM1M, WDR82, MIRLET7G, GLYCTK, GLYCTK-AS1, MIR135A1, DNAH1, BAP1, PHF7, SEMA3G, TNNC1, NISCH, STAB1, NT5DC2, UQCC5, PBRM1, GNL3, SNORD136, SNORD19, SNORD19B, SNORD19C, SNORD69, GLT8D1, SPCS1, NEK4, ITIH1, ITIH3, ITIH4, ITIH4-AS1, MUSTN1, STIMATE-MUSTN1, STIMATE, MIR8064, SFMBT1, RFT1, PRKCD, TKT, DCP1A, SNORD38C, CACNA1D | arr[GRCh38] 3p21.31p21.1(47,612,885_53,630,208)x2 hmz |
| 1  Brain | cn-LOH | 5 | p15.33 | p11 | 46,246 | PLEKHG4B, LRRC14B, CCDC127, SDHA, PDCD6-DT, PDCD6, PDCD6-AHRR, AHRR, EXOC3-AS1, EXOC3, SLC9A3, SLC9A3-OT1, SLC9A3-AS1, MIR4456, CEP72-DT, CEP72, TPPP, ZDHHC11B, ZDHHC11, BRD9, TRIP13, LINC02982, NKD2, SLC12A7, MIR4635, TERLR1, SLC6A19, SLC6A18, TERT, MIR4457, CLPTM1L, LINC01511, SLC6A3, LPCAT1, MIR6075, SDHAP3, PDCD6P1, MIR4277, MRPL36, NDUFS6, LINC02116, IRX4, IRX4-AS1, CTD-2194D22.4, LOC105374618, LOC100506858, LSINCT5, IRX2, IRX2-DT, LOC105374620, LINC01377, LINC01019, LINC02162, LINC01017, IRX1, LINC02063, LINC02114, LINC01020, LINC02121, ADAMTS16-DT, ADAMTS16, ICE1, LINC02145, MED10, UBE2QL1, LINC01018, NSUN2, SRD5A1, LINC02102, TENT4A, LINC02236, MIR4278, LOC442132, LINC02123, LINC02142, ADCY2, CFAP90, FASTKD3, MTRR, LINC02226, MIR4458HG, MIR4458, LINC02199, SEMA5A, MIR4636, SEMA5A-AS1, SNHG18, SNORD123, TAS2R1, LINC02112, LINC02221, ATPSCKMT, CCT5, CMBL, MARCHF6-DT, MARCHF6, MIR10397, ROPN1L-AS1, ROPN1L, MIR6131, LINC02212, LINC02213, ANKRD33B, DAP, CTNND2, LINC01194, LINC02220, DNAH5, TRIO, SNORD170, OTULINL, SNORD141B, OTULIN-DT, OTULIN, ANKH, LOC100130744, MIR4637, LINC02149, FBXL7, CTD-2350J17.1, MIR887, MARCHF11, MARCHF11-DT, LINC02150, ZNF622, RETREG1, RETREG1-AS1, MYO10, BASP1-AS1, MIR10522, BASP1, LINC02111, LINC02217, LINC02218, H3Y2, TAF11L2, TAF11L3, TAF11L4, TAF11L5, TAF11L6, TAF11L7, TAF11L8, TAF11L9, TAF11L10, TAF11L11, TAF11L12, TAF11L13, TAF11L14, H3Y1, LINC02223, CDH18, CDH18-AS1, LINC02241, LINC02146, GUSBP1, CDH12, SNORA105A, PMCHL1, PRDM9, LINC02899, CDH10, LINC02239, LINC02228, LINC02211, CDH9, PURPL, LINC02103, LSP1P3, LOC101929645, LINC02109, LINC02064, LOC105374704, CDH6, DROSHA, C5orf22, PDZD2, MIR4279, GOLPH3, MTMR12, ZFR, MIR579, SUB1, NPR3, LINC02120, LINC02160, TARS1, ADAMTS12, RXFP3, SLC45A2, AMACR, C1QTNF3-AMACR, C1QTNF3, LOC646652, RAI14-DT, RAI14, TTC23L-AS1, TTC23L, RAD1, BRIX1, DNAJC21, AGXT2, PRLR, SPEF2, IL7R, CAPSL, CAPSL-DT, UGT3A1, UGT3A2, LMBRD2, MIR580, SKP2, NADK2, NADK2-AS1, RANBP3L, SLC1A3, NIPBL-DT, NIPBL, CPLANE1, CPLANE1-AS1, NUP155, WDR70, GDNF, GDNF-AS1, LINC02110, LINC02107, LINC02119, EGFLAM, EGFLAM-AS4, EGFLAM-AS2, EGFLAM-AS1, LIFR, LIFR-AS1, MIR3650, OSMR-DT, LINC01265, OSMR, RICTOR, FYB1, C9, DAB2, LINC02104, LINC00603, LINC00604, PTGER4, TTC33, PRKAA1, RPL37, SNORD72, CARD6, C7, MROH2B, C6, PLCXD3, OXCT1, OXCT1-AS1, RIMOC1, FBXO4, LINC02996, GHR, CCDC152, SELENOP, FLJ32255, ANXA2R-OT1, ANXA2R, ANXA2R-AS1, LOC100132356, LOC100506639, ZNF131, NIM1K, HMGCS1, CCL28, TMEM267, C5orf34, PAIP1, NNT-AS1, NNT, FGF10, FGF10-AS1, LINC02224, MRPS30-DT, MRPS30, HCN1 | arr[GRCh38] 5p15.33p11(137,401_46,383,233)x2 hmz |
| 1  Brain | cn-LOH | 5 | q11.1 | q13.2 | 19,244 | EMB, PARP8, LINC02106, ISL1-DT, ISL1, LINC02118, PELO, ITGA1, ITGA2, MOCS2, MOCS2-DT, FST, NDUFS4, LINC02105, ARL15, MIR581, LINC01033, HSPB3, SNX18, LINC02998, ESM1, LOC102467081, GZMK, GZMA, CDC20B, GPX8, MIR449A, MIR449B, MIR449C, MCIDAS, CCNO, DHX29, MTREX, PLPP1, MIR5687, RNF138P1, SLC38A9, DDX4, IL31RA, IL6ST, IL6ST-DT, ANKRD55, LINC01948, C5orf67, LOC105378979, MAP3K1, SETD9, MIER3, GPBP1, ACTBL2, LNCBRM, LOC101928505, LINC02225, LINC02101, PLK2, GAPT, MIR548AE2, LINC02108, RAB3C, PDE4D, MIR582, PART1, DEPDC1B, ELOVL7, ERCC8, ERCC8-AS1, NDUFAF2, SMIM15, SMIM15-AS1, LINC02057, ZSWIM6, C5orf64, LOC101928651, C5orf64-AS1, KIF2A, DIMT1, IPO11, IPO11-LRRC70, LRRC70, HTR1A, RNF180, RGS7BP, SHISAL2B, SREK1IP1, CWC27, ADAMTS6, CENPK, PPWD1, TRIM23, SHLD3, TRAPPC13, SGTB, NLN, ERBIN, LOC100303749, SREK1, LINC02065, LINC02229, MAST4, MAST4-AS1, CD180, LINC02242, LINC02997, LINC02219, PIK3R1, LINC02198, SLC30A5, SNORA50D, CCNB1, CENPH, MRPS36, CDK7, CCDC125, AK6, TAF9, RAD17, MARVELD2, LOC101928924, OCLN | arr[GRCh38] 5q11.1q13.2(50,265,025_69,508,917)x2 hmz |
| 1  Brain | cn-LOH | 5 | q13.2 | q35.3 | 109,874 | LINC02197, PMCHL2, BDP1, MCCC2, CARTPT, MAP1B, MIR4803, MRPS27, PTCD2, ZNF366, LOC102503427, LINC02056, TNPO1, MIR4804, FCHO2, TMEM171, LOC105379030, TMEM174, LOC340090, LINC02230, FOXD1, LINC01386, BTF3, ANKRA2, UTP15, ARHGEF28, LINC02122, LINC01335, LINC01333, LINC01331, ENC1, HEXB, GFM2, NSA2, FAM169A, FAM169A-AS1, GCNT4, ANKRD31, HMGCR, CERT1, POLK, ANKDD1B, POC5, LOC441087, SV2C-AS1, SV2C, IQGAP2, LOC101929109, F2RL2, NCRUPAR, F2R, F2RL1, S100Z, CRHBP, AGGF1, ZBED3, SNORA47, ZBED3-AS1, PDE8B, WDR41, OTP, TBCA, LOC101929154, AP3B1, SCAMP1-AS1, SCAMP1, LHFPL2, ARSB, DMGDH, BHMT2, BHMT, JMY, HOMER1, TENT2, CMYA5, LINC01455, MTX3, THBS4, THBS4-AS1, SERINC5, LOC644936, SPZ1, CRSP8P, ZFYVE16, FAM151B-DT, FAM151B, ANKRD34B, LINC01337, DHFR, MSH3, RASGRF2-AS1, RASGRF2, CKMT2, CKMT2-AS1, ZCCHC9, ACOT12, SSBP2, ATG10, RPS23, ATP6AP1L, MIR3977, LINC01338, TMEM167A, SCARNA18, XRCC4, VCAN, VCAN-AS1, HAPLN1, EDIL3, EDIL3-DT, NBPF22P, COX7C, SNORD138, LINC02059, MIR4280, LOC101929380, LINC01949, RASA1, CCNH, LOC644285, LINC02144, LINC02488, TMEM161B, TMEM161B-DT, LINC02060, LINC00461, MIR9-2, MEF2C-AS2, MEF2C, MEF2C-AS1, MIR3660, LINC01339, CETN3, LOC731157, MBLAC2, POLR3G, LYSMD3, ADGRV1, LUCAT1, ARRDC3, ARRDC3-AS1, NR2F1-AS1, NR2F1, FAM172A, MIR2277, POU5F2, KIAA0825, SLF1, MCTP1, FAM81B, SKIC3, ARSK, GPR150, RFESD, SPATA9, RHOBTB3, GLRX, LOC102724720, LINC01554, ELL2, LOC101929710, MIR583, PCSK1, CAST, ERAP1, ERAP2, LNPEP, LIX1, RIOK2, LINC01340, LINC02234, LINC01846, RGMB, RGMB-AS1, CHD1, CHD1-DT, LOC100289230, LINC02113, GUSBP19, FAM174A-DT, FAM174A, ST8SIA4, MIR548P, SLCO4C1, SLCO6A1, LINC00492, LINC00491, PAM, GIN1, PPIP5K2, MACIR, LINC02115, NUDT12, RAB9BP1, LINC01950, EFNA5, FBXL17, LINC01023, FER, LOC285638, PJA2, MAN2A1, LINC01848, TMEM232, MIR548F3, SLC25A46, TSLP, WDR36, CAMK4, STARD4, STARD4-AS1, NREP, NREP-AS1, EPB41L4A, EPB41L4A-AS1, SNORA13, LOC101927023, EPB41L4A-DT, LINC02200, LOC102467216, APC, SRP19, REEP5, DCP2, MCC, TSSK1B, YTHDC2, KCNN2, LOC101927078, LINC01957, TRIM36, PGGT1B, CCDC112, FEM1C, TICAM2, TMED7-TICAM2, TICAM2-AS1, TMED7, LOC102467217, CDO1, ATG12, AP3S1, LINCADL, LVRN, ARL14EPL, COMMD10, MIR12130, LOC101927190, SEMA6A, SEMA6A-AS1, SEMA6A-AS2, LINC02214, LINC00992, LINC02147, LINC02148, LINC02208, LINC02216, LINC02215, DTWD2, MIR1244-2, DMXL1-DT, DMXL1, MIR5706, TNFAIP8, HSD17B4, FAM170A, PRR16, LOC102467226, FTMT, SRFBP1, LOX, ZNF474, LOC100505841, SNCAIP, MGC32805, LOC101927357, LINC02201, SNX2, SNX24, PPIC, PRDM6-AS1, PRDM6, CEP120, CSNK1G3, LINC01170, ZNF608, LOC101927421, LINC02240, LINC02039, LOC101927488, GRAMD2B, ALDH7A1, PHAX, TEX43, LMNB1-DT, LMNB1, MARCHF3, C5orf63, MEGF10, PRRC1, CTXN3, CCDC192, SLC12A2-DT, SLC12A2, FBN2, SLC27A6, ISOC1, MIR4633, MIR4460, ADAMTS19-AS1, ADAMTS19, MINAR2, CHSY3, HINT1, LYRM7, CDC42SE2, RAPGEF6, FNIP1, MEIKIN, ACSL6, ACSL6-AS1, IL3, CSF2, P4HA2-AS1, P4HA2, MIR6830, PDLIM4, SLC22A4, MIR3936HG, MIR3936, SLC22A5, IRF1-AS1, IRF1, IL5, RAD50, TH2LCRR, IL13, IL4, LOC105379176, KIF3A, CCNI2, SEPTIN8, SOWAHA, SHROOM1, GDF9, UQCRQ, LEAP2, AFF4, ZCCHC10, HSPA4, FSTL4, MIR1289-2, WSPAR, C5orf15, LOC105379183, VDAC1, TCF7, SKP1, PPP2CA, MIR3661, CDKL3, UBE2B, CDKN2AIPNL, LINC02999, LINC01843, JADE2, SAR1B, SEC24A, CAMLG, DDX46, C5orf24, TXNDC15, PCBD2, CATSPER3, PITX1, PITX1-AS1, EPIST, LINC02900, MACROH2A1, DCANP1, TIFAB, NEUROG1, CXCL14, SLC25A48, SLC25A48-AS1, MIR5692C1, LOC107986453, IL9, FBXL21P, LECT2, TGFBI, VTRNA2-1, SMAD5-AS1, SMAD5, SMIM32, TRPC7, TRPC7-AS1, TRPC7-AS2, SPOCK1, LOC105379192, KLHL3, MIR874, HNRNPA0, NPY6R, MYOT, PKD2L2, FAM13B, FAM13B-AS1, WNT8A, NME5, BRD8, KIF20A, CDC23, GFRA3, CDC25C, LOC100128966, FAM53C, KDM3B, REEP2, EGR1, ETF1, HSPA9, SNORD63B, SNORD63, CTNNA1-AS1, CTNNA1, LRRTM2, SIL1, SNHG4, MATR3, SNORA74D, SNORA74A, PAIP2, SLC23A1, MZB1, PROB1, SPATA24, DNAJC18, ECSCR, SMIM33, STING1, UBE2D2, CXXC5, CXXC5-AS1, PSD2-AS1, PSD2, NRG2, MALINC1, PURA, IGIP, LOC101929719, CYSTM1, PFDN1, HBEGF, SLC4A9, ANKHD1, ANKHD1-EIF4EBP3, EIF4EBP3, SRA1, APBB3, MIR6831, SLC35A4, CD14, TMCO6, NDUFA2, IK, MIR3655, WDR55, DND1, HARS1, HARS2, ZMAT2, VTRNA1-1, VTRNA1-2, VTRNA1-3, PCDHA1, PCDHA2, PCDHA3, PCDHA4, PCDHA5, PCDHA6, PCDHA7, PCDHA8, PCDHA9, PCDHA10, PCDHA11, LOC112267934, PCDHA12, PCDHA13, PCDHAC1, PCDHAC2, PCDHB1-AS1, PCDHB1, PCDHB2, PCDHB3, PCDHB4, PCDHB5, PCDHB6, PCDHB17P, PCDHB7, PCDHB8, PCDHB16, PCDHB9, PCDHB10, PCDHB11, PCDHB12, PCDHB13, PCDHB14, PCDHB18P, PCDHB19P, PCDHB15, SLC25A2, TAF7, PCDHGA1, PCDHGA2, PCDHGA3, PCDHGB1, PCDHGA4, PCDHGB2, PCDHGA5, PCDHGB3, PCDHGA6, PCDHGA7, PCDHGB4, PCDHGA8, PCDHGB5, PCDHGA9, PCDHGB6, PCDHGA10, PCDHGB7, PCDHGA11, PCDHGB8P, PCDHGA12, PCDHGC3, PCDHGC4, PCDHGC5, DIAPH1, DIAPH1-AS1, HDAC3, RELL2, FCHSD1, ARAP3, PCDH1, LOC729080, DELE1, PCDH12, RNF14, GNPDA1, NDFIP1, SPRY4, SPRY4-IT1, SPRY4-AS1, FGF1, LINC01844, ARHGAP26, ARHGAP26-AS1, ARHGAP26-IT1, NR3C1, MIR5197, HMHB1, YIPF5, KCTD16, PRELID2, GRXCR2, SH3RF2, PLAC8L1, LARS1, RBM27, LOC127814297, POU4F3, TCERG1, GPR151, PPP2R2B, PPP2R2B-IT1, STK32A-AS1, STK32A, DPYSL3, JAKMIP2-AS1, JAKMIP2, SPINK1, SCGB3A2, C5orf46, SPINK5, SPINK14, SPINK6, MARCOL, FBXO38-DT, SPINK13, SPINK7, SPINK9, FBXO38, HTR4, ADRB2, SH3TC2, MIR584, SH3TC2-DT, ABLIM3, AFAP1L1, GRPEL2, GRPEL2-AS1, PCYOX1L, IL17B, CARMN, MIR143, MIR145, CSNK1A1, ARHGEF37, PPARGC1B, MIR378A, PDE6A, MFFP2, SLC26A2, TIGD6, HMGXB3, CSF1R, PDGFRB, CDX1, SLC6A7, CAMK2A, ARSI, TCOF1, CD74, RPS14, NDST1-AS1, NDST1, SYNPO, MYOZ3, RBM22, DCTN4, SMIM3, IRGM, ZNF300, ZNF300P1, GPX3, TNIP1, ANXA6, CCDC69, LOC105378230, GM2A, SLC36A3, SLC36A2, SLC36A1, FAT2, MIR6499, SPARC, CLMAT3, ATOX1, LOC100652758, G3BP1, GLRA1, LINC01933, NMUR2, LINC01470, GRIA1, LINC01861, FAM114A2, MFAP3, GALNT10, MIR1294, SAP30L-AS1, SAP30L, HAND1, MIR3141, LARP1, MIR1303, FAXDC2, MIR378H, CNOT8, GEMIN5, MRPL22, KIF4B, SGCD, PPP1R2B, TIMD4, HAVCR1, HAVCR2, MED7, GARIN3, ITK, CYFIP2, FNDC9, NIPAL4-DT, NIPAL4, ADAM19, SOX30, C5orf52, THG1L, LSM11, CLINT1, LINC02227, EBF1, LINC02202, RNF145, LINC01932, UBLCP1, IL12B, LOC285626, LINC01845, LINC01847, ADRA1B, TTC1, PWWP2A, FABP6, CCNJL, C1QTNF2, FAM200C, SLU7, PTTG1, MIR3142HG, MIR3142, MIR146A, ATP10B, LINC02159, GABRB2, GABRA6, GABRA1, LINC01202, GABRG2, CCNG1, NUDCD2, HMMR, HMMR-AS1, MAT2B, LINC02143, LOC102546299, LINC01938, LINC01947, TENM2, LOC101927908, MIR12125, TENM2-AS1, WWC1, RARS1, FBLL1, PANK3, MIR103A1, MIR103B1, SLIT3, SLIT3-AS2, MIR218-2, SLIT3-AS1, MIR585, SPDL1, DOCK2, INSYN2B, MIR378E, FOXI1, LINC01187, C5orf58, LCP2, LOC100128059, LINC01366, KCNIP1, KCNMB1, KCNIP1-OT1, KCNIP1-AS1, GABRP, RANBP17, TLX3, SNORA70J, MIR3912, NPM1, FGF18, SMIM23, FBXW11, STK10, EFCAB9, UBTD2, LOC100288254, SH3PXD2B, LINC01944, NEURL1B, MIR5003, LOC101928093, DUSP1, ERGIC1, MIR10523, RPL26L1-AS1, RPL26L1, ATP6V0E1, SNORA74B, CREBRF, BNIP1, NKX2-5, MIR12118, STC2, MIR8056, LINC02995, BOD1, LINC01942, LINC01484, LINC01485, CPEB4, C5orf47, NSG2, LINC01411, MSX2, MIR4634, LINC01951, DRD1, SFXN1, HRH2, CPLX2, THOC3, THOC3-AS1, LOC100996385, FAM153B, LOC643201, SIMC1, KIAA1191, ARL10, MIR1271, NOP16, HIGD2A, CLTB, FAF2, RNF44, CDHR2, GPRIN1, SNCB, MIR4281, EIF4E1B, TSPAN17, LINC01574, UNC5A, HK3, UIMC1, ZNF346, FGFR4, NSD1, RAB24, PRELID1, MXD3, LMAN2, RGS14, SLC34A1, PFN3, F12, GRK6, PRR7-AS1, PRR7, DBN1, PDLIM7, DOK3, DDX41, FAM193B, TMED9, B4GALT7, LOC202181, FAM153A, LOC105377752, LOC728554, LOC100128340, PROP1, FAM153CP, N4BP3, RMND5B, NHP2, GMCL2, HNRNPAB, PHYKPL, COL23A1, CLK4, MSANTD5, ZNF354A, AACSP1, ZNF354B, ZFP2, ZNF454, GRM6, ZNF879, ZNF354C, ADAMTS2, LOC100289470, RUFY1, RUFY1-AS1, HNRNPH1, C5orf60, LOC100502572, LOC105377763, CBY3, CANX, MAML1, LTC4S, MGAT4B, MIR1229, SQSTM1, MRNIP, MRNIP-DT, TBC1D9B, RNF130, MIR340, RASGEF1C, MAPK9, GFPT2, CNOT6, SCGB3A1, FLT4, LINC02222, OR2Y1, MGAT1, HEIH, LINC00847, ZFP62, BTNL8, BTNL3, BTNL9, MIR8089, OR2V1, OR2V2, LINC01962, TRIM7-AS2, TRIM7, MIR4638, TRIM41, RACK1, SNORD96A, SNORD95, CTC-338M12.4, TRIM52 | arr[GRCh38] 5q13.2q35.3(71,384,705_181,258,604)x2 hmz |
| 1  Brain | Gain | 6 | p25.3 | q16.3 | 102,736 | FOXF2, MIR6720, FOXCUT, FOXC1, GMDS, GMDS-DT, LINC01600, LINC02521, MYLK4, WRNIP1, SERPINB1, MIR4645, SERPINB9P1, SERPINB9-AS1, SERPINB9, SERPINB6, LINC01011, NQO2, HTATSF1P2, LOC101927759, RIPK1, BPHL, TUBB2A, LINC02525, TUBB2B, LOC100422781, PSMG4, SLC22A23, LOC643327, PXDC1, FAM50B, PRPF4B, FAM217A, TEX56P, ECI2, ECI2-DT, LOC102724096, LINC02533, KU-MEL-3, CDYL, CDYL-AS1, RPP40, LYRM4-AS1, LYRM4, PPP1R3G, MIR3691, FARS2, FARS2-AS1, LOC101927950, NRN1, F13A1, MIR7853, MIR5683, LY86-AS1, LY86, RREB1, SSR1, CAGE1, RIOK1, DSP-AS1, DSP, SNRNP48, BMP6, BLOC1S5-TXNDC5, TXNDC5, PIP5K1P1, BLOC1S5, EEF1E1-BLOC1S5, EEF1E1, SCARNA27, SLC35B3, LOC100506207, HULC, OFCC1, TFAP2A, TFAP2A-AS2, TFAP2A-AS1, LINC00518, MIR5689HG, MIR5689, LINC02522, GCNT2, C6orf52, PAK1IP1, TMEM14C, TMEM14B, MAK, GCM2, SYCP2L, LOC101928191, ELOVL2, ELOVL2-AS1, SMIM13, ERVFRD-1, NEDD9, TMEM170B, ADTRP, LOC101928253, HIVEP1, EDN1, LINC02530, PHACTR1, TBC1D7-LOC100130357, LOC100130357, TBC1D7, GFOD1, GFOD1-AS1, SIRT5, NOL7, RANBP9, MCUR1, RNF182, CD83, LINC01108, JARID2, JARID2-AS1, DTNBP1, LINC02543, MYLIP, MIR4639, GMPR, ATXN1, LOC127903862, ATXN1-AS1, STMND1, RBM24, CAP2, LOC101928491, FAM8A1, NUP153, NUP153-AS1, KIF13A, NHLRC1, TPMT, KDM1B, DEK, RNF144B, MIR548A1HG, MIR548A1, LOC101928519, LOC105374960, LNC-LBCS, ID4, MBOAT1, E2F3, E2F3-IT1, CDKAL1, LINC00581, SOX4, CASC15, NBAT1, PRL, HDGFL1, LINC03005, NRSN1, DCDC2, KAAG1, MRS2, GPLD1, ALDH5A1, KIAA0319, TDP2, ACOT13, C6orf62, LINC02828, GMNN, ARMH2, RIPOR2, CMAHP, LOC101928663, CARMIL1, SCGN, H2AC1, H2BC1, H2AC2P, SLC17A4, SLC17A1, SLC17A3, SLC17A2, TRIM38, H1-1, H3C1, H4C1, H4C2, H3C2, H2AC4, H2BC3, H3C3, H1-2, HFE-AS1, HFE, H4C3, H1-6, H2BC4, H2AC6, H1-4, H2BC5, H2BC6, H4C4, H3C4, H2AC7, H2BC7, H4C5, H2BC8, H2AC8, H3C6, H1-3, H4C6, H4C7, H3C7, H2BC9, H3C8, H2BC10, H4C8, BTN3A2, BTN2A2, BTN3A1, BTN2A3P, BTN3A3, BTN2A1, LOC285819, BTN1A1, HCG11, HMGN4, LOC105374988, ABT1, ZNF322, GUSBP2, LINC00240, LARRPM, H2BC11, H2AC11, H2BC12, H4C9, H2AC12, MIR3143, PRSS16, POM121L2, VN1R10P, ZNF204P, ZNF391, ZNF184, LINC01012, LOC100131289, H2BC13, H2AC13, H3C10, H2AC14, H2BC14, H4C11, H4C12, H2AC15, H2BC15, H2AC16, H1-5, H3C11, H4C13, H3C12, H2AC17, H2BC17, OR2B2, OR2B6, OR2B8P, ZNF165, ZSCAN12P1, ZSCAN16-AS1, ZSCAN16, ZKSCAN8, ZKSCAN8P1, TOB2P1, ZSCAN9, ZKSCAN4, NKAPL, ZSCAN26, PGBD1, ZSCAN31, ZKSCAN3, ZSCAN12, ZSCAN23, GPX6, GPX5, SCAND3, LINC00533, LINC01623, HCG14, TRIM27, LINC01556, HCG15, ZNF311, OR2W1-AS1, OR2W1, OR2B3, OR2J1, OR2J3, OR2J2, LINC03003, OR14J1, OR5V1, OR12D3, OR12D2, OR12D1, OR11A1, OR10C1, OR2H1, MAS1L, LINC02829, LINC01015, OR2I1P, UBD, SNORD32B, OR2H2, GABBR1, MOG, ZFP57, HLA-F, HLA-F-AS1, IFITM4P, HCG4, HLA-V, HLA-G, HCP5B, HLA-H, HCG4B, HLA-A, HCG9, POLR1HASP, HLA-J, POLR1H, PPP1R11, RNF39, TRIM31, TRIM31-AS1, TRIM40, TRIM10, TRIM15, TRIM26, HCG17, HLA-L, HCG18, TRIM39, TRIM39-RPP21, RPP21, HLA-E, LINC02569, GNL1, PRR3, ABCF1, MIR877, PPP1R10, MRPS18B, ATAT1, C6orf136, DHX16, PPP1R18, NRM, MDC1, MDC1-AS1, TUBB, FLOT1, IER3-AS1, IER3, HCG20, LINC00243, LINC02570, DDR1, MIR4640, GTF2H4, VARS2, SFTA2, MUCL3, HCG21, MUC21, MUC22, HCG22, C6orf15, PSORS1C1, CDSN, PSORS1C2, CCHCR1, TCF19, POU5F1, PSORS1C3, HCG27, HLA-C, LINC02571, HLA-B, MIR6891, MICA-AS1, MICA, LINC01149, HCP5, HCG26, MICB-DT, MICB, MCCD1, ATP6V1G2-DDX39B, DDX39B, SNORD117, SNORD84, DDX39B-AS1, ATP6V1G2, NFKBIL1, LOC100287329, LTA, TNF, LTB, LST1, NCR3, AIF1, PRRC2A, SNORA38, MIR6832, BAG6, APOM, C6orf47, GPANK1, CSNK2B, LY6G5B, LY6G5C, ABHD16A, MIR4646, LY6G6F, LY6G6F-LY6G6D, LY6G6E, LY6G6D, LY6G6C, MPIG6B, DDAH2, CLIC1, MSH5-SAPCD1, MSH5, SAPCD1, SAPCD1-AS1, VWA7, VARS1, LSM2, HSPA1L, HSPA1A, HSPA1B, SNHG32, SNORD48, SNORD52, NEU1, SLC44A4, EHMT2-AS1, EHMT2, C2, ZBTB12, C2-AS1, CFB, NELFE, MIR1236, SKIC2, DXO, STK19, C4A, CYP21A1P, TNXA, C4B, CYP21A2, TNXB, ATF6B, FKBPL, PRRT1, LOC100507547, PPT2, PPT2-EGFL8, EGFL8, AGPAT1, MIR6721, RNF5, MIR6833, AGER, PBX2, GPSM3, NOTCH4, TSBP1-AS1, TSBP1, HCG23, BTNL2, HLA-DRA, HLA-DRB5, HLA-DRB6, HLA-DRB1, HLA-DQA1, HLA-DQB1, HLA-DQB1-AS1, HLA-DQA2, MIR3135B, HLA-DQB2, HLA-DOB, TAP2, PSMB8, PSMB8-AS1, TAP1, PSMB9, LOC100294145, HLA-DMB, HLA-DMA, BRD2, HLA-DOA, HLA-DPA1, HLA-DPB1, HLA-DPB2, HCG24, COL11A2, RXRB, SLC39A7, HSD17B8, MIR219A1, RING1, HCG25, VPS52, RPS18, B3GALT4, WDR46, MIR6873, PFDN6, MIR6834, RGL2, TAPBP, ZBTB22, DAXX, SMIM40, KIFC1, PHF1, CUTA, SYNGAP1, SYNGAP1-AS1, MIR5004, ZBTB9, BAK1, GGNBP1, LINC00336, ITPR3, ITPR3-AS1, UQCC2, MIR3934, IP6K3, LEMD2, MLN, LINC01016, MIR7159, MIR1275, GRM4, HMGA1, MIR6835, SMIM29, NUDT3, RPS10-NUDT3, RPS10, PACSIN1, SPDEF, ILRUN, ILRUN-AS1, SNRPC, BLTP3A, TAF11, ANKS1A, TCP11, SCUBE3, ZNF76, DEF6, PPARD, FANCE, RPL10A, MIR7111, TEAD3, TULP1, FKBP5, MIR5690, LOC285847, ARMC12, CLPSL2, CLPSL1, CLPS, LHFPL5, SRPK1, SLC26A8, MAPK14, MAPK13, BRPF3, PNPLA1, BNIP5, ETV7, PXT1, KCTD20, STK38, SRSF3, MIR3925, PANDAR, CDKN1A, DINOL, RAB44, CPNE5, PPIL1, C6orf89, PI16, MTCH1, FGD2, PIM1, TMEM217, TMEM217B, TBC1D22B, RNF8, LOC107986531, CMTR1, CCDC167, LINC02520, MIR4462, MDGA1, ZFAND3, BTBD9, BTBD9-AS1, GLO1, DNAH8, DNAH8-AS1, GLP1R, MIR9983, SAYSD1, KCNK5, KCNK17, KCNK16, KIF6, DAAM2, DAAM2-AS1, MOCS1, LINC00951, TDRG1, LRFN2, LOC105379699, LOC101929555, UNC5CL, TSPO2, APOBEC2, OARD1, NFYA, ADCY10P1, TREML1, TREM2, TREML2, TREML3P, TREML4, TREML5P, TREM1, NCR2, LINC01276, FOXP4-AS1, FOXP4, MIR4641, MDFI, TFEB, MIR10398, PGC, FRS3, PRICKLE4, TOMM6, USP49, MED20, BYSL, CCND3, TAF8, C6orf132, GUCA1ANB, GUCA1ANB-GUCA1A, GUCA1A, GUCA1B, MRPS10, TRERF1, LOC107986596, UBR2, PRPH2, ATP6V0CP3, TBCC, BICRAL, LOC401261, RPL7L1, C6orf226, PTCRA, CNPY3, CNPY3-GNMT, GNMT, PEX6, PPP2R5D, MEA1, KLHDC3, RRP36, CUL7, MRPL2, KLC4, PTK7, SRF, CUL9, DNPH1, TTBK1, SLC22A7, CRIP3, ZNF318, ABCC10, MIR6780B, DLK2, TJAP1, LRRC73, YIPF3, POLR1C, XPO5, POLH, GTPBP2, MAD2L1BP, RSPH9, MRPS18A, VEGFA, LINC02537, LINC01512, SCIRT, LINC03040, MRPL14, TMEM63B, CAPN11, MYMX, SLC29A1, HSP90AB1, SLC35B2, MIR4647, NFKBIE, TMEM151B, TCTE1, AARS2, SPATS1, CDC5L, MIR4642, LOC105375075, SUPT3H, MIR586, RUNX2, CLIC5, ENPP4, ENPP5, RCAN2, LOC101926915, RCAN2-DT, CYP39A1, SLC25A27, TDRD6-AS1, TDRD6, PLA2G7, ANKRD66, MEP1A, ADGRF5, ADGRF5-AS1, ADGRF1, TNFRSF21, CD2AP, ADGRF2, ADGRF4, OPN5, PTCHD4, MMUT, CENPQ, GLYATL3, C6orf141, RHAG, CRISP2, CRISP3, PGK2, LOC101927020, LOC101927048, CRISP1, DEFB133, DEFB114, DEFB113, DEFB110, DEFB112, TFAP2D, TFAP2B, PKHD1, LOC101927082, MIR206, LINCMD1, MIR133B, IL17A, IL17F, MCM3, PAQR8, EFHC1, TRAM2, TRAM2-AS1, LOC730101, TMEM14A, GSTA7P, GSTA2, GSTA1, GSTA5, GSTA3, GSTA4, RN7SK, CILK1, LOC128092246, FBXO9, LOC128031835, GCM1, ELOVL5, MIR5685, RPS16P5, GCLC-AS1, GCLC, KILH, LINC01564, KLHL31, LRRC1, LOC101927189, MLIP-IT1, MLIP, MLIP-AS1, TINAG, FAM83B, HCRTR2, GFRAL, HMGCLL1, BMP5, COL21A1, DST, DST-AS1, BEND6, KIAA1586, ZNF451, ZNF451-AS1, BAG2, RAB23, LINC03001, PRIM2, MIR548U, GUSBP4, LINC00680-GUSBP4, LINC00680, KHDRBS2, FKBP1C, LGSN, PTP4A1, LOC128125822, PHF3, EYS, SCAT8, ZC3H11C, SLC25A51P1, LOC102723883, LINC02549, ADGRB3-DT, ADGRB3, LOC127898563, LMBRD1, COL19A1, COL9A1, LINC01610, FAM135A-AS1, FAM135A, SDHAF4, SMAP1, B3GAT2, OGFRL1, MIR30C2, MIR30A, LINC00472, LINC01626, RIMS1, KCNQ5, KCNQ5-IT1, MIR4282, KCNQ5-AS1, KHDC1L, LOC122539213, KHDC1, LOC122539212, C6orf147, DPPA5, KHDC3L, OOEP, OOEP-AS1, DDX43, CGAS, MTO1, EEF1A1, SLC17A5, CD109-AS1, CD109, LOC101928516, COL12A1, SNORD156, COX7A2, TMEM30A, TMEM30A-DT, FILIP1, LOC101928540, MIR4463, SENP6, MYO6, IMPG1, LINC02540, HTR1B, MEI4, MIR10524, IRAK1BP1, PHIP, HMGN3, HMGN3-AS1, LCAL1, LCA5, SH3BGRL2, LINC01621, ELOVL4, TTK, BCKDHB, TENT5A, LINC01526, LINC02542, IBTK, TPBG, UBE3D, DOP1A, PGM3, RWDD2A, ME1, PRSS35, SNAP91, LOC105377879, RIPPLY2, RIPPLY2-CYB5R4, CYB5R4, MRAP2, CEP162, LINC01611, TBX18-AS1, TBX18, LINC02535, NT5E, SNX14, SYNCRIP, SNHG5, SNORD50A, SNORD50B, HTR1E, CGA, ZNF292, GJB7, SMIM8, C6orf163, LINC01590, CFAP206, SLC35A1, RARS2, ORC3, AKIRIN2, LOC101928911, SPACA1, CNR1, LOC101928936, RNGTT, PNRC1, SRSF12, PM20D2, GABRR1, GABRR2, UBE2J1, RRAGD, ANKRD6, LYRM2, MDN1-AS1, MDN1, CASP8AP2, GJA10, BACH2, MIR4464, MAP3K7, MIR4643, CASC6, EPHA7, TSG1, MANEA-DT, MANEA, FUT9, UFL1, FHL5, GPR63, NDUFAF4, KLHL32, MMS22L, LOC101927314, MIR2113, PNKY, POU3F2, FBXL4, MIR548AI, FAXC, COQ3, PNISR, PNISR-AS1, USP45, TSTD3, CCNC, PRDM13, MCHR2, MCHR2-AS1, SIM1, ASCC3, GRIK2 | arr[GRCh38] 6p25.3q16.3(1,350,885_104,087,099)x3~4 |
| 1  Brain | cn-LOH | 6 | q16.3 | q21 | 7,496 | HACE1, LIN28B-AS1, LIN28B, BVES, BVES-AS1, POPDC3, PREP, PRDM1, ATG5, CRYBG1, LOC105377924, RTN4IP1, QRSL1, LINC02526, LINC02532, MIR587, CD24, MTRES1, BEND3, PDSS2, SOBP, SCML4, SEC63, OSTM1, OSTM1-AS1, NR2E1, SNX3, AFG1L, FOXO3, LINC00222, ARMC2, ARMC2-AS1, SESN1, CEP57L1, CCDC162P, CD164, PPIL6, SMPD2, MICAL1, ZBTB24, AK9, FIG4, GPR6, WASF1, CDC40, METTL24, DDO, SLC22A16, CDK19, AMD1, SNORA40C, GTF3C6, RPF2, GSTM2P1, SLC16A10, SNORD166, MFSD4B, REV3L, TRAF3IP2-AS1, TRAF3IP2, FYN, LOC102724646, LINC02527, CCN6, TUBE1, FAM229B, LAMA4 | arr[GRCh38] 6q16.3q21(104,700,606_112,196,566)x2 hmz |
| 1  Brain | Gain | 6 | q21 | q23.1 | 17,918 | LINC02518, LINC02541, MARCKS, MROCKI, LINC02880, HDAC2, HDAC2-AS2, HS3ST5, LNCPOIR, LINC02534, FRK, TPI1P3, NT5DC1, COL10A1, TSPYL4, DSE, TSPYL1, LOC100287467, CALHM6-AS1, CALHM6, TRAPPC3L, CALHM5, CALHM4, RWDD1, RSPH4A, ZUP1, KPNA5, FAM162B, GPRC6A, RFX6, VGLL2, ROS1, DCBLD1, GOPC, LOC101927919, NUS1, SLC35F1, LOC105377967, CEP85L, BRD7P3, PLN, SELENOKP3, MCM9, ASF1A, FAM184A, MIR548B, MAN1A1, LOC285762, LOC105377975, MIR3144, TBC1D32, GJA1, HSF2, SERINC1, PKIB, FABP7, SMPDL3A, CLVS2, TRDN, TRDN-AS1, NKAIN2, RNF217-AS1, RNF217, TPD52L1, LOC100126584, HDDC2, HEY2-AS1, LINC02523, HEY2, NCOA7, NCOA7-AS1, HINT3, TRMT11, CENPW, MIR588, RSPO3, RNF146, ECHDC1, KIAA0408, SOGA3-KIAA0408, SOGA3, C6orf58, THEMIS, PTPRK, PTPRK-AS1, LAMA2, ARHGAP18, TMEM244, L3MBTL3, SAMD3, TMEM200A | arr[GRCh38] 6q21q23.1(112,548,611_130,466,978)x3~4 |
| 1  Brain | Gain | 6 | q23.2 | q27 | 37,026 | TARID, LINC01312, TCF21, TBPL1, LOC128092253, SLC2A12, HMGA1P7, SGK1, CT69, LINC01010, LINC03002, ALDH8A1, HBS1L, MIR3662, MYB, MIR548A2, AHI1, AHI1-DT, LINC02524, PDE7B, PDE7B-AS1, MTFR2, BCLAF1, MAP7, MAP3K5, MAP3K5-AS1, MAP3K5-AS2, PEX7, SLC35D3, NHEG1, IL20RA, IL22RA2, IFNGR1, OLIG3, LINC03004, LINC02539, WAKMAR2, TNFAIP3, SIMALR, LINC02865, PERP, ARFGEF3, PBOV1, SMIM28, HEBP2, NHSL1, MIR3145, NHSL1-AS1, CCDC28A-AS1, CCDC28A, ECT2L, REPS1, ABRACL, HECA, TXLNB, CITED2, LINC01625, FILNC1, LINC02941, LOC103352541, MIR3668, MIR4465, NMBR, GJE1, VTA1, ADGRG6, LOC153910, HIVEP2, LINC01277, AIG1, ADAT2, PEX3, FUCA2, PHACTR2, PHACTR2-AS1, LTV1, ZC2HC1B, PLAGL1, HYMAI, SF3B5, STX11, UTRN, SNORA98, EPM2A, EPM2A-DT, FBXO30, SHPRH, GRM1, RAB32, ADGB-DT, ADGB, KATNBL1P6, STXBP5-AS1, LUADT1, STXBP5, SAMD5, SASH1, UST, UST-AS1, UST-AS2, TAB2, TAB2-AS1, SUMO4, ZC3H12D, PPIL4, GINM1, RPS18P9, KATNA1, LATS1, LOC645967, NUP43, PCMT1, LRP11, RAET1E-LRP11, RAET1E-AS1, RAET1E, RAET1G, LOC105378052, ULBP2, ULBP1, RAET1K, RAET1L, ULBP3, PPP1R14C, IYD, PLEKHG1, MTHFD1L, MIR12131, LOC102723831, AKAP12, ZBTB2, RMND1, ARMT1, CCDC170, ESR1, SYNE1, SYNE1-AS1, MYCT1, VIP, LINC02840, FBXO5, MTRF1L, RGS17, OPRM1, IPCEF1, CNKSR3, SCAF8, MIR1273C, TIAM2, TFB1M, CLDN20, NOX3, LOC105378068, MIR1202, SNORD28B, ARID1B, LOC115308161, MIR4466, TMEM242, ZDHHC14, MIR3692, SNX9, SNX9-AS1, SYNJ2, SYNJ2-IT1, SERAC1, GTF2H5, TULP4, SNORA116, TMEM181, MIR7161, DYNLT1, SYTL3, MIR3918, EZR, EZR-AS1, OSTCP1, LINC02901, RSPH3, TAGAP-AS1, TAGAP, FNDC1-AS1, FNDC1, FNDC1-IT1, LINC02529, SOD2, WTAP, SOD2-OT1, ACAT2, TCP1, SNORA20, SNORA29, MRPL18, PNLDC1, MAS1, IGF2R, AIRN, CHP1P2, SLC22A1, SLC22A2, SLC22A3, LPAL2, LPA, PLG, MAP3K4-AS1, MAP3K4, AGPAT4, AGPAT4-IT1, PRKN, LOC105378098, PACRG, PACRG-AS2, PACRG-AS3, PACRG-AS1, DKFZp451B082, CAHM, QKI, LOC102724152, MEAT6, C6orf118, PDE10A, LINC00473, LINC00602, LOC729681, TBXT, LNCDAT, LOC101929297, PRR18, SFT2D1, LOC100289495, MPC1, RPS6KA2, RPS6KA2-IT1, MIR1913, RAMACL, RPS6KA2-AS1, RNASET2, MIR3939, CEP43, CCR6, GPR31, LOC105378123, HPAT5, UNC93A, TTLL2, TCP10L3, LOC105378127, LINC02538, LINC02487, LINC01558, LOC441179, AFDN-DT, AFDN, HGC6.3, KIF25-AS1, KIF25, FRMD1, LOC105378137, LOC101929420, DACT2, SMOC2, LOC105378146, LOC101929460, LOC102724357, LINC01615, LINC02544, THBS2-AS1, THBS2, WDR27, C6orf120, PHF10, DYNLT2, ERMARD, LINC00242, LINC00574, LOC100131532, LOC102724511, LOC154449, LOC285804, LINC01624, DLL1, FAM120B, MIR4644, PSMB1, TBP, PDCD2 | arr[GRCh38] 6q23.2q27(133,584,207_170,610,394)x3~4 |
| 1  Brain | Gain | 7 | p22.3 | p14.1 | 38,567 | LINC03015, LINC03014, LOC105375115, FAM20C, FOXL3, FOXL3-OT1, LOC442497, LOC116435278, LOC112267991, PDGFA, PDGFA-DT, PRKAR1B, PRKAR1B-AS2, PRKAR1B-AS1, DNAAF5, SUN1, GET4, ADAP1, COX19, C7orf50, CYP2W1, MIR339, GPR146, GPER1, ZFAND2A, ZFAND2A-DT, UNCX, MICALL2, INTS1, MAFK, LOC100128653, TMEM184A, PSMG3, PSMG3-AS1, TFAMP1, LOC105375303, ELFN1, ELFN1-AS1, MAD1L1, MIR4655, SNORA114, MRM2, NUDT1, SNX8, MIR6836, EIF3B, CHST12, GRIFIN, LFNG, MIR4648, BRAT1, IQCE, TTYH3, AMZ1, GNA12, CARD11, LOC100129603, SDK1, LOC105375131, FOXK1, AP5Z1, MIR4656, RADIL, SNORD165, PAPOLB, MMD2, RNF216P1, RBAK, RBAK-RBAKDN, RBAKDN, ZNF890P, WIPI2, SLC29A4, TNRC18, LINC02983, FBXL18, MIR589, LOC221946, ACTB, FSCN1, RNF216, RNF216-IT1, MIR6874, ZNF815P, OCM, CCZ1, RSPH10B, PMS2, AIMP2, SNORA80D, EIF2AK1, ANKRD61, USP42, CYTH3, FAM220A, SAGSIN1, RAC1, DAGLB, KDELR2, GRID2IP, ZDHHC4, INTS15, ZNF853, ZNF316, ZNF12, SPDYE20P, PMS2CL, RSPH10B2, CCZ1B, MIR3683, LOC100131257, C1GALT1, LINC03016, COL28A1, MIOS-DT, MIOS, RPA3, UMAD1, GLCCI1-DT, GLCCI1, ICA1, ICA1-AS1, NXPH1, PER3P1, MGC4859, LOC100131472, NDUFA4, PHF14, THSD7A, TMEM106B, VWDE, LOC102725191, SCIN, ARL4A, ETV1, DGKB, AGMO, MEOX2, LOC105375166, LINC02587, CRPPA, CRPPA-AS1, SOSTDC1, LRRC72, ANKMY2, BZW2, TSPAN13, AGR2, AGR3, AHR, LINC02888, LINC02889, SNX13, PRPS1L1, HDAC9, MIR1302-6, TWIST1, FERD3L, POLR1F, MIR3146, TMEM196, LOC101927668, MACC1, MACC1-AS1, GIRGL, LOC101927769, ITGB8-AS1, ITGB8, ABCB5, SP8, RPL23P8, LINC01162, SP4, MIR1183, DNAH11, CDCA7L, RAPGEF5, STEAP1B, STEAP1B-AS1, LOC401312, IL6-AS1, IL6, TOMM7, SNHG26, SNORD93, HYCC1, KLHL7-DT, KLHL7, NUP42, GPNMB, MALSU1, IGF2BP3, SNORD65C, RPS2P32, TRA2A, CLK2P1, CCDC126, FAM221A, STK31, NPY, PALS2, GSDME, OSBPL3, CYCS, C7orf31, NPVF, LINC03007, MIR148A, NFE2L3, HNRNPA2B1, CBX3, SNX10, SNX10-AS1, LINC02981, KIAA0087, LINC03095, LINC02860, SKAP2, HOXA1, HOTAIRM1, HOXA2, HOXA3, HOXA-AS2, HOXA4, HOXA-AS3, HOXA5, HOXA6, HOXA7, HOXA9, HOXA10-HOXA9, HOXA10-AS, MIR196B, HOXA10, HOXA11, HOXA11-AS, HOXA13, HOTTIP, EVX1-AS, EVX1, HIBADH, TSL, TAX1BP1, JAZF1, JAZF1-AS1, CREB5, TRIL, CPVL-AS2, CPVL, CHN2, CHN2-AS1, PRR15-DT, PRR15, LOC646762, MIR550A3, ZNRF2P2, DPY19L2P3, WIPF3, SCRN1, FKBP14, PLEKHA8, MTURN, LOC105375218, ZNRF2, MIR550A1, MIR550B1, DKFZP586I1420, LINC01176, NOD1, GGCT, GARS1-DT, GARS1, CRHR2, INMT, INMT-MINDY4, MINDY4, AQP1, GHRHR, ADCYAP1R1, NEUROD6, ITPRID1, PPP1R17, PDE1C, LOC100130673, LSM5, AVL9, DPY19L1P1, ZNRF2P1, MIR550A2, MIR550B2, LINC00997, DPY19L1P2, KBTBD2, RP9P, FKBP9, NT5C3A, RP9, BBS9, FLJ20712, BMPER, NPSR1-AS1, NPSR1, DPY19L1, MIR548N, DPY19L2P1, TBX20, LOC401324, HERPUD2, HERPUD2-AS1, LINC03013, SEPTIN7-DT, SEPTIN7, LOC101928618, EEPD1, MATCAP2, ANLN, AOAH, AOAH-IT1, ELMO1, MIR1200, ELMO1-AS1, GPR141, NME8, SFRP4, EPDR1, STARD3NL, TARP, TRG-AS1, AMPH | arr[GRCh38] 7p22.3p14.1(43,361_38,610,703)x3~4 |
| 1  Brain | Gain | 7 | p14.1 | p11.2 | 16,952 | LINC01449, INHBA, INHBA-AS1, GLI3, LINC01448, C7orf25, PSMA2, MRPL32, HECW1, HECW1-IT1, MIR3943, LUARIS, STK17A, COA1, BLVRA, MRPS24, URGCP-MRPS24, URGCP, UBE2D4, POLR2J4, SPDYE1, RASA4CP, LINC00957, DBNL, MIR6837, PGAM2, POLM, MIR6838, AEBP1, MIR4649, POLD2, MYL7, GCK, YKT6, CAMK2B, NUDCD3, NPC1L1, DDX56, TMED4, OGDH, ZMIZ2, PPIA, H2AZ2, PURB, MIR4657, MYO1G, SNHG15, SNORA9, CCM2, NACAD, TBRG4, SNORA5A, SNORA5C, SNORA5B, RAMP3, ADCY1, SEPTIN7P2, LOC730234, CCDC201, IGFBP1, IGFBP3, LOC730338, TNS3, SNORD151, LINC01447, LINC02902, LINC00525, PKD1L1, PKD1L1-AS1, HUS1, SUN3, C7orf57, UPP1, ABCA13, LINC02838, CDC14C, VWC2, ZPBP, SPATA48, IKZF1, FIGNL1, DDC, DDC-AS1, GRB10, COBL, LOC107986794, POM121L12, LINC01446, LINC01445, VSTM2A, VSTM2A-OT1, SEC61G, SEC61G-DT, EGFR, EGFR-AS1, ELDR, LANCL2, VOPP1, FKBP9P1, SEPTIN14, ZNF713, MRPS17, NIPSNAP2, PSPH, CCT6A, SNORA22B, SNORA15, SUMF2, PHKG1, CHCHD2, NUPR2, LOC650226, LOC100240728, DKFZp434L192, LOC101928401, LOC401357, LOC100130849, MIR4283-1, ZNF479, GUSBP10, LOC105375297, MIR3147HG, MIR3147, ZNF716 | arr[GRCh38] 7p14.1p11.2(41,013,703_57,966,167)x3~4 |
| 1  Brain | Gain | 7 | q11.21 | q21.11 | 15,386 | ZNF733P, LOC102724738, LOC100287704, LOC100287834, MIR4283-2, ZNF722, LINC01005, ZNF727, ZNF735, ZNF679, ZNF736, YWHAEP1, ZNF680, LOC100128885, LOC641746, ZNF107, MIR6839, ZNF138, ZNF273, ZNF117, ERV3-1-ZNF117, ERV3-1, CCT6P3, SNORA22C, SNORA15B-1, INTS4P1, ZNF92, LOC101929322, LINC03006, INTS4P2, CCT6P1, SNORA22, SNORA15B-2, VKORC1L1, GUSB, ASL, CRCP, TPST1, LINC00174, LINC03011, RABGEF1P1, KCTD7, LOC100996437, RABGEF1, GTF2IRD1P1, GTF2IP23, LINC02604, TMEM248, SBDS, TYW1, MIR4650-1, PMS2P4, SPDYE21, STAG3L4, LINC01372, LOC102723427, CT66, AUTS2, GALNT17, MIR3914-1, MIR3914-2, CALN1, TYW1B, MIR4650-2, SBDSP1, SPDYE7P, POM121, NSUN5P2, TRIM74, LOC100101148, STAG3L3, PMS2P7, SPDYE8, SPDYE11, SPDYE9, SPDYE10, GTF2IP4, NCF1B, NSUN5, TRIM50, FKBP6, FZD9, BAZ1B, BCL7B, TBL2, MLXIPL, VPS37D, DNAJC30, BUD23, STX1A, MIR4284, ABHD11-AS1, ABHD11, CLDN3, CLDN4, METTL27, TMEM270, ELN, ELN-AS1, LIMK1, EIF4H, MIR590, LAT2, RFC2, CLIP2, GTF2IRD1, MIR10525, GTF2I, GTF2I-AS1, NCF1, GTF2IRD2, STAG3L2, PMS2P5, SPDYE12, CASTOR2, RCC1L, GTF2IRD2B, NCF1C, GTF2IP1, SPDYE14, SPDYE13, SPDYE15, PMS2P2, STAG3L1, LOC541473, TRIM73, NSUN5P1, POM121C, SPDYE5, PMS2P3, HIP1, CCL26, CCL24, RHBDD2, POR, MIR4651, SNORA14A, TMEM120A, STYXL1, MDH2, GTF2IP7, SRRM3, HSPB1, YWHAG, SSC4D, ZP3, DTX2, FDPSP2, UPK3B, SPDYE16, LINC03009, POMZP3, DTX2P1-UPK3BP1-PMS2P11, SPDYE17, PMS2P9, SPDYE18, FAM185BP, CCDC146, FGL2, GSAP, LOC101927243, PTPN12, APTR, RSBN1L, TMEM60, PHTF2, DDX3ILA1, MAGI2, RPL13AP17 | arr[GRCh38] 7q11.21q21.11(62,977,012_78,362,941)x3~4 |
| 1  Brain | Loss | 8 | p23.3 | p11.21 | 41,947 | ERICH1, DLGAP2, LOC401442, LOC105377777, LOC286083, DLGAP2-AS1, CLN8-AS1, CLN8, MIR3674, MIR596, ARHGEF10, KBTBD11-AS1, KBTBD11-OT1, KBTBD11, MYOM2, MIR7160, LINC03021, LOC105377785, CSMD1, MCPH1-DT, MCPH1, ANGPT2, MCPH1-AS1, MIR8055, AGPAT5, MIR4659A, MIR4659B, XKR5, GS1-24F4.2, DEFB1, DEFA6, DEFA4, DEFA8P, DEFA9P, DEFA10P, DEFA1, DEFT1P, DEFA1B, DEFT1P2, DEFA3, DEFA11P, DEFA5, LOC101928095, LINC00965, FAM66B, DEFB109B, USP17L1, USP17L4, ZNF705G, DEFB4B, DEFB103B, SPAG11B, DEFB104B, DEFB106B, DEFB105B, DEFB107B, PRR23D1, FAM90A7, FAM90A22, FAM90A23, FAM90A14, FAM90A18, FAM90A16, FAM90A8, FAM90A17, FAM90A19, FAM90A9, FAM90A10, PRR23D2, DEFB107A, DEFB105A, DEFB106A, DEFB104A, SPAG11A, DEFB103A, DEFB4A, ZNF705B, FAM66E, USP17L8, USP17L3, MIR548I3, FAM85B, FAM86B3P, PRAG1, CLDN23, MFHAS1, ERI1, MIR4660, SNORD3I, PPP1R3B, PPP1R3B-DT, LOC101929128, LOC157273, TNKS, MIR597, MIR124-1HG, MIR124-1, MSRA, LINC03022, PRSS55, RP1L1, MIR4286, C8orf74, SOX7, LOC102723313, PINX1, MIR1322, PINX1-DT, XKR6, MIR598, LOC101929269, LINC00529, MTMR9, SLC35G5, TDH, TDH-AS1, FAM167A-AS1, FAM167A, BLK, LINC00208, GATA4, SNORA99, LINC02905, NEIL2, FDFT1, CTSB, DEFB136, DEFB135, DEFB134, DEFB130B, ZNF705D, FAM66D, LOC392196, USP17L7, USP17L2, FAM90A2P, FAM86B1, FAM85A, DEFB130A, FAM66A, LOC649352, DEFB109A, FAM90A25P, FAM86B2, FAM86B2-DT, LOC729732, MIR5692A2, LONRF1, MIR3926-1, MIR3926-2, LINC03019, LINC00681, TRMT9B, DLC1, C8orf48, LOC102725080, SGCZ, MIR383, TUSC3, MSR1, FGF20, MICU3, ZDHHC2, CNOT7, VPS37A, MTMR7, SLC7A2, PDGFRL, MTUS1, MIR548V, FGL1, PCM1, ASAH1, ASAH1-AS1, NAT1, NAT2, PSD3, LOC100128993, SH2D4A, CSGALNACT1, INTS10, LPL, SLC18A1, ATP6V1B2, LZTS1, LZTS1-AS1, SNORD3F, LINC03023, LINC02153, LINC03093, GFRA2, DOK2, XPO7, NPM2, FGF17, DMTN, FHIP2B, NUDT18, HR, HRURF, REEP4, LGI3, SFTPC, BMP1, PHYHIP, MIR320A, POLR3D, PIWIL2-DT, PIWIL2, SLC39A14, PPP3CC, SORBS3, PDLIM2, C8orf58, LOC107986876, CCAR2, BIN3, BIN3-IT1, EGR3, PEBP4, LOC101929237, RHOBTB2, TNFRSF10B, LOC286059, LOC254896, TNFRSF10C, TNFRSF10D, TNFRSF10A, TNFRSF10A-DT, CHMP7, R3HCC1, LOXL2, LOXL2-AS1, ENTPD4, SLC25A37, NKX3-1, NKX2-6, STC1, ADAM28, ADAM7-AS1, ADAMDEC1, ADAM7, ADAM7-AS2, NEFM, NEFL, MIR6841, DOCK5, MIR6876, LOC105379331, GNRH1, KCTD9, CDCA2, EBF2, PPP2R2A, SDAD1P1, BNIP3L, PNMA2, DPYSL2, ADRA1A, MIR548H4, STMN4, TRIM35, PTK2B, MIR6842, CHRNA2, EPHX2, CLU, MIR6843, SCARA3, MIR3622B, MIR3622A, CCDC25, ESCO2, PBK, SCARA5, MIR4287, NUGGC, ELP3, PNOC, ZNF395, FBXO16, FZD3, MIR4288, EXTL3-AS1, EXTL3, INTS9, HMBOX1, KIF13B, DUSP4, LINC00589, LINC02099, LOC101929470, LINC02209, MIR3148, SARAF, LEPROTL1, MBOAT4, DCTN6, RBPMS-AS1, RBPMS, GTF2E2, SMIM18, GSR, UBXN8, PPP2CB, TEX15, PURG, WRN, NRG1, NRG1-IT1, NRG1-IT3, LOC128092250, FUT10, MAK16, TTI2, SNORD13, RNF122, DUSP26, LINC01288, UNC5D, LOC101929550, KCNU1, LINC01605, ZNF703, LOC101929622, LOC102723701, ERLIN2, LOC728024, PLPBP, ADGRA2, BRF2, RAB11FIP1, GOT1L1, ADRB3, EIF4EBP1, ASH2L, STAR, LSM1, BAG4, DDHD2, PLPP5, NSD3, LETM2, FGFR1, LINC03042, RNF5P1, TACC1, PLEKHA2, HTRA4, TM2D2, ADAM9, SNORD38D, ADAM32, ADAM5, ADAM3A, LOC100130964, ADAM18, ADAM2, IDO1, IDO2, TCIM, SIRLNT, ZMAT4, SFRP1, MIR548AO, SNORD65B, GOLGA7, GINS4, GPAT4-AS1, GPAT4, NKX6-3, ANK1, MIR486-1, MIR486-2, KAT6A, KAT6A-AS1, AP3M2, PLAT, IKBKB-DT, IKBKB, POLB, DKK4, VDAC3, SLC20A2 | arr[GRCh38] 8p23.3p11.21(591,636_42,538,556)x1 |
| 1  Brain | Gain | 8 | p11.21 | q24.3 | 102,532 | SLC20A2, SMIM19, CHRNB3, CHRNA6, THAP1, RNF170, MIR4469, HOOK3, FNTA, POMK, HGSNAT, POTEA, ASNSP1, LINC00293, LOC100287846, SPIDR, CEBPD, PRKDC, MCM4, UBE2V2, LINC02947, LOC101929268, LINC03054, CLXN, SNAI2, PPDPFL, LOC100507464, SNTG1, PXDNL, PCMTD1, ST18, LOC101929341, ALKAL1, RB1CC1, NPBWR1, OPRK1, LINC02984, ATP6V1H, RGS20, TCEA1, LYPLA1, MRPL15, RNU105C, SOX17, RP1, XKR4, SBF1P1, XKR4-AS1, TMEM68, TGS1, LYN, SNORA1B, RPS20, SNORD54, CERNA3, MOS, PLAG1, CHCHD7, SDR16C5, SDR16C6P, PENK, PENK-AS1, LINC00968, BPNT2, LINC01606, LOC286177, LINC00588, LOC101929488, LINC03018, LINC01602, FAM110B, LOC101929528, UBXN2B, CYP7A1, SDCBP, NSMAF, TOX, TOX-DT, CA8, LINC01301, RAB2A, CHD7, LOC105375938, LOC100130298, CLVS1, ASPH, MIR4470, LINC02155, NKAIN3, GGH, TTPA, YTHDF3-DT, YTHDF3, LOC102724612, LINC01289, LINC01414, MIR124-2HG, MIR124-2, BHLHE22-AS1, BHLHE22, CYP7B1, LINC00251, LINC01299, ARMC1, MTFR1, PDE7A, DNAJC5B, TRIM55, CRH, LINC00967, RRS1-DT, RRS1, ADHFE1, VXN, MYBL1, VCPIP1, C8orf44, C8orf44-SGK3, SGK3, PTTG3P, MCMDC2, SNHG6, SNORD87, TCF24, PPP1R42, COPS5, CSPP1, ARFGEF1, ARFGEF1-DT, CPA6, PREX2, C8orf34-AS1, C8orf34, LINC01592, LINC01603, SULF1, SLCO5A1, PRDM14, NCOA2, LINC03020, TRAM1, LACTB2-AS1, LACTB2, XKR9, EYA1, MSC, MSC-AS1, TRPA1, LOC392232, KCNB2, LOC101926908, TERF1, SBSPON, C8orf89, RPL7, RDH10, RDH10-AS1, STAU2-AS1, STAU2, UBE2W, LINC01617, ELOC, TMEM70, LY96, JPH1, GDAP1, MIR5681A, MIR5681B, MIR2052HG, LINC03071, MIR2052, PI15, CRISPLD1, CASC9, HNF4G, LINC01109, LINC01111, ZFHX4-AS1, ZFHX4, MIR3149, PEX2, LOC102724874, PKIA, PKIA-AS1, ZC2HC1A, C4orf46P3, IL7, LINC02605, MIR12123, STMN2, HEY1, LINC01607, LOC101927040, MRPS28, TPD52-MRPS28, TPD52, MIR5708, ZBTB10, ZNF704, PAG1, FABP5, PMP2, FABP9, FABP4, FABP12, IMPA1P1, IMPA1, SLC10A5, ZFAND1, CHMP4C, SNX16, LINC02235, LINC02839, LOC101927141, LINC01419, RALYL, LRRCC1, E2F5-DT, E2F5, RBIS, CA13, CA1, CA3, CA3-AS1, CA2, REXO1L2P, PSKH2, ATP6V0D2, SLC7A13, WWP1, RMDN1, CPNE3, CNGB3, CNBD1, DCAF4L2, MMP16, RIPK2-DT, RIPK2, OSGIN2, NBN, DECR1, CALB1, LINC00534, LINC01030, TMEM64, NECAB1, C8orf88, PIP4P2, OTUD6B-AS1, OTUD6B, LRRC69, MIR4661, SLC26A7, RUNX1T1, LOC102724710, FLJ46284, TRIQK, MIR8084, LINC02906, CIBAR1-DT, CIBAR1, RBM12B, RBM12B-AS1, RBM12B-DT, TMEM67, MIR378D2HG, MIR378D2, PDP1, CDH17, GEM, RAD54B, FSBP, VIRMA, VIRMA-DT, LINC02894, ESRP1, DPY19L4, INTS8, CCNE2, NDUFAF6, TP53INP1, MIR3150BHG, MIR3150B, MIR3150A, PLEKHF2, LINC01298, CFAP418, CFAP418-AS1, SRSF3P2, GDF6, UQCRB, UQCRB-AS1, MTERF3, PTDSS1, LOC102724804, SDC2, CPQ, LOC101927066, TSPYL5, SNORD3H, MTDH, LAPTM4B, MATN2, RPL30, SNORA72, ERICH5, RIDA, POP1, NIPAL2, MIR9903, KCNS2, STK3, OSR2, VPS13B, MIR599, MIR875, COX6C, RGS22, SNORD77B, FBXO43, POLR2K, SPAG1, RNF19A, MIR4471, ANKRD46, SNX31, PABPC1, MIR7705, YWHAZ, FLJ42969, ZNNT1, ZNF706, LINC02844, NACA4P, GRHL2, NCALD, LOC104054148, MIR5680, RRM2B, UBR5-DT, UBR5, LOC105375683, ODF1, KLF10, LOC101927245, GASAL1, AZIN1, MAILR, ATP6V1C1, LINC01181, BAALC-AS2, BAALC, MIR3151, BAALC-AS1, LOC105369147, FZD6, SNORD173, CTHRC1, SLC25A32, DCAF13, LOC105375690, RIMS2, DCSTAMP, DPYS, MIR548A3, LRP12, ZFPM2, ZFPM2-AS1, OXR1, ABRA, ANGPT1, RSPO2, EIF3E, EMC2, TMEM74, TRHR, NUDCD1, ENY2, PKHD1L1, EBAG9, SYBU, SYBU-AS1, KCNV1, LINC01608, LINC01609, LINC02237, CSMD3, MIR2053, TRPS1, LINC00536, EIF3H, LOC105375713, UTP23, RAD21, RAD21-AS1, MIR3610, AARD, SLC30A8, MED30, EXT1, SNORD168, SAMD12, SAMD12-AS1, TNFRSF11B, COLEC10, LOC101927513, MAL2, MAL2-AS1, MIR548AZ, CCN3, ENPP2, TAF2, DSCC1, DEPTOR, COL14A1, MRPL13, MTBP, SNTB1, LOC101927543, HAS2, HAS2-AS1, LINC02855, SMILR, LINC01151, ZHX2, DERL1, TBC1D31, FAM83A, FAM83A-AS1, MIR4663, C8orf76, ZHX1-C8orf76, ZHX1, ATAD2, MIR548AA1, MIR548D1, NTAQ1, FBXO32, KLHL38, ANXA13, FAM91A1, FER1L6, FER1L6-AS1, FER1L6-AS2, LOC101927588, TMEM65, TRMT12, RNF139-DT, RNF139, TATDN1, MIR6844, NDUFB9, MTSS1, MIR4662B, MIR4662A, LINC00964, ZNF572, SQLE-DT, SQLE, WASHC5, WASHC5-AS1, NSMCE2, TRIB1, LINC00861, LOC101927657, LRATD2, PCAT1, PCAT2, PRNCR1, CASC19, CCAT1, CASC21, CASC8, CCAT2, POU5F1B, CASC11, MYC, PVT1, MIR1204, LINC02912, MIR1205, MIR1206, MIR1207, MIR1208, LINC00824, LINC00976, LINC00977, CCDC26, MIR3686, GSDMC, CYRIB, MIR5194, ASAP1, ASAP1-IT2, ASAP1-IT1, ADCY8, EFR3A, OC90, HHLA1, KCNQ3, HPYR1, DNAAF11, TMEM71, PHF20L1, TG, SLA, MIR7848, PTCSC1, CCN4, NDRG1, ST3GAL1, LINC03024, LOC101927798, LOC101927822, ZFAT, ZFAT-AS1, MIR30B, MIR30D, NCRNA00250, LOC101927845, LINC01591, KHDRBS3, LINC02055, LOC101927915, LOC401478, FAM135B, COL22A1, KCNK9, TRAPPC9, C8orf17, PEG13, CHRAC1, AGO2, ERICD, PTK2, MIR151A, DENND3-AS1, DENND3, SLC45A4, SLC45A4-AS1, LINC01300, GPR20, PTP4A3, MROH5, C8orf90, MIR1302-7, MIR4472-1, LINC00051, TSNARE1, ADGRB1, ARC, LOC101928087, JRK, PSCA, LY6K, LNCOC1, THEM6, SLURP1, LYPD2, SLURP2, LYNX1-SLURP2, LYNX1, LY6D, GML, CYP11B1, CYP11B2, LY6E-DT, CDC42P3, LY6E, LINC02904, LY6S, LY6L, LY6H, GPIHBP1, ZFP41, GLI4, MINCR, ZNF696, TOP1MT, RHPN1-AS1, RHPN1, MAFA-AS1, MAFA, ZC3H3, SNORD149, GSDMD, LOC100310756, MROH6, NAPRT, EEF1D, TIGD5, PYCR3, GFUS, ZNF623, ZNF707, LINC02878, CCDC166, LOC101928160, MAPK15, FAM83H, MIR4664, IQANK1, LOC105375800, SCRIB, MIR937, PUF60, NRBP2, MIR6845, EPPK1, PLEC, MIR661, PARP10, GRINA, SPATC1, SMPD5, OPLAH, MIR6846, EXOSC4, MIR6847, GPAA1, CYC1, SHARPIN, MAF1, WDR97, HGH1, MROH1, BOP1, MIR7112, SCX, HSF1, DGAT1, MIR6848, SCRT1, TMEM249, FBXL6, SLC52A2, LOC101928902, ADCK5, CPSF1, MIR939, MIR1234, MIR6849, SLC39A4, VPS28, TONSL, TONSL-AS1, MIR6893, ZFTRAF1, TMEM276-ZFTRAF1, MIR10400, TMEM276, KIFC2, FOXH1, PPP1R16A, GPT, MFSD3, RECQL4, LRRC14, LRRC24, C8orf82, ARHGAP39, ZNF251, ZNF34, RPL8, MIR6850, ZNF517, LOC100130027, ZNF7, COMMD5, ZNF250, ZNF16, ZNF252P, TMED10P1, ZNF252P-AS1, C8orf33 | arr[GRCh38] 8p11.21q24.3(42,538,718_145,070,385)x4 |
| 1  Brain | Gain | 12 | p13.33 | p11.1 | 34,480 | IQSEC3, IQSEC3-AS2, LOC574538, IQSEC3-AS1, SLC6A12, SLC6A12-AS1, SLC6A13, LOC102723544, KDM5A, CCDC77, B4GALNT3, NINJ2, LOC105369595, NINJ2-AS1, WNK1, RAD52, ERC1, LINC00942, LOC107984507, FBXL14, WNT5B, MIR3649, ADIPOR2, CACNA2D4, LRTM2, LINC00940, DCP1B, CACNA1C-IT2, CACNA1C, CACNA1C-AS4, CACNA1C-IT3, CACNA1C-AS2, CACNA1C-AS1, ITFG2-AS1, FKBP4, ITFG2, NRIP2, TEX52, FOXM1, RHNO1, TULP3, TEAD4, TSPAN9, LOC100128253, LINC02417, PRMT8, THCAT155, CRACR2A, PARP11, PARP11-AS1, CCND2-AS1, CCND2, TIGAR, FGF23, FGF6, C12orf4, RAD51AP1, DYRK4, AKAP3, NDUFA9, GAU1, GALNT8, KCNA6, KCNA1, KCNA5, LINC02443, NTF3, ANO2, VWF, SNORA120, CD9, PLEKHG6, TNFRSF1A, SCNN1A, LTBR, CD27-AS1, CD27, TAPBPL, VAMP1, MRPL51, NCAPD2, SCARNA10, GAPDH, IFFO1, NOP2, CHD4, SCARNA11, LPAR5, ACRBP, ING4, ZNF384, PIANP, COPS7A, MLF2, PTMS, LAG3, CD4, GPR162, P3H3, GNB3, CDCA3, USP5, TPI1, SPSB2, LOC105369632, RPL13P5, DSTNP2, LRRC23, ENO2, ATN1, C12orf57, RNU7-1, PTPN6, MIR200CHG, MIR200C, MIR141, PHB2, SCARNA12, EMG1, LPCAT3, C1S, C1R, C1RL, C1RL-AS1, RBP5, CLSTN3, PEX5, ACSM4, CD163L1, CD163, APOBEC1, GDF3, DPPA3, CLEC4C, NANOGNB, NANOG, SLC2A14, SLC2A3, FOXJ2, C3AR1, NECAP1, CLEC4A, POU5F1P3, ZNF705A, FAM66C, FAM90A1, FAM86FP, LINC02449, LINC00937, CLEC6A, CLEC4D, CLEC4E, AICDA, MFAP5, RIMKLB, A2ML1, PHC1, M6PR, KLRG1, LINC00612, A2M-AS1, A2M, PZP, A2MP1, MIR1244-3, LINC00987, LOC642846, LINC02367, LOC101928030, LOC728715, DDX12P, LOC408186, KLRB1, LOC374443, LOC105369728, CLEC2D, CLECL1P, CD69, KLRF1, CLEC2B, KLRF2, CLEC2A, LINC02470, CLEC12A-AS1, CLEC12A, CLEC1B, CLEC12B, LOC102724020, CLEC9A, CLEC1A, CLEC7A, OLR1, TMEM52B, GABARAPL1, GABARAPL1-AS1, KLRD1, LINC02617, LINC02598, KLRK1-AS1, KLRK1, KLRC4-KLRK1, KLRC4, KLRC3, KLRC2, KLRC1, EIF2S3B, LINC02446, KLRA1P, MAGOHB, STYK1, YBX3, LINC02366, TAS2R7, TAS2R8, TAS2R9, TAS2R10, PRR4, PRH1-PRR4, PRH1, TAS2R13, PRH2, TAS2R14, PRH1-TAS2R14, TAS2R50, TAS2R20, TAS2R19, TAS2R31, TAS2R46, TAS2R43, TAS2R30, SMIM10L1, TAS2R42, PRB3, PRB4, PRB1, PRB2, LOC440084, LINC01252, ETV6, BCL2L14, MIR1244-4, LRP6, MANSC1, LOH12CR2, BORCS5, DUSP16, CREBL2, GPR19, CDKN1B, APOLD1, MIR613, DDX47, RPL13AP20, GPRC5A, MIR614, GPRC5D-AS1, GPRC5D, HEBP1, HTR7P1, FAM234B, GSG1, EMP1, LINC01559, GRIN2B, ATF7IP, PLBD1, PLBD1-AS1, GUCY2C, H4C16, H2AJ, WBP11, C12orf60, SMCO3, ART4, MGP, ERP27, ARHGDIB, PDE6H, LINC01489, RERG, RERG-AS1, PTPRO, EPS8, STRAP, DERA, SLC15A5, MGST1, LMO3, SKP1P2, LINC02378, MIR3974, RERGL, PIK3C2G, PLCZ1, CAPZA3, PLEKHA5, AEBP2, LINC02398, LINC02468, PDE3A, SLCO1C1, SLCO1B3, SLCO1B3-SLCO1B7, SLCO1B1, SLCO1A2, IAPP, PYROXD1, RECQL, GOLT1B, SPX, GYS2, LDHB, KCNJ8, ABCC9, CMAS, ST8SIA1, C2CD5, ETNK1-DT, ETNK1, LOC101928441, SOX5, MIR920, SOX5-AS1, LINC00477, BCAT1, LINC02909, IRAG2, DNAI7, ETFRF1, KRAS, LMNTD1, MIR4302, RASSF8-AS1, RASSF8, BHLHE41, SSPN, ITPR2, INTS13, FGFR1OP2, TM7SF3, MED21, C12orf71, STK38L, BMAL2, BMAL2-AS1, SMCO2, PPFIBP1, REP15, MRPS35, MANSC4, KLHL42, PTHLH, LOC729291, CCDC91, FAR2, LOC100506606, ERGIC2, OVCH1-AS1, OVCH1, TMTC1, LINC02386, IPO8, CAPRIN2, LOC645485, LINC00941, TSPAN11, DDX11-AS1, DDX11, OVOS2, SINHCAF, FLJ13224, LINC02387, DENND5B, DENND5B-AS1, ETFBKMT, AMN1, H3-5, LINC02422, RESF1, BICD1, FGD4, DNM1L, YARS2, PKP2, SYT10, ALG10 | arr[GRCh38] 12p13.33p11.1(64,621_34,544,509)x3 |
| 1  Brain | Gain | 12 | q12 | q12 | 5,720 | ALG10B, CPNE8, LINC02406, KIF21A, ABCD2, C12orf40, SLC2A13, LINC02471, LRRK2, MUC19, CNTN1, PDZRN4, LINC02400, GXYLT1, YAF2, ZCRB1, MIR7851, PPHLN1, PRICKLE1, LINC02402, LINC02451, LINC02450, LINC02461, ADAMTS20 | arr[GRCh38] 12q12(37,983,642_43,703,708)x3 |
| 1  Brain | Gain | 12 | q12 | q14.2 | 18,290 | NELL2, DBX2, RACGAP1P1, PLEKHA8P1, ANO6, LINC00938, ARID2, SCAF11, SLC38A1, SLC38A2, SLC38A4-AS1, SLC38A4, AMIGO2, PCED1B, MIR4698, PCED1B-AS1, LOC105369747, LINC02416, MIR4494, LINC02156, RPAP3, RPAP3-DT, ENDOU, RAPGEF3, SLC48A1, HDAC7, VDR, TMEM106C, COL2A1, SENP1, PFKM, MIR6505, ASB8, CCDC184, OR10AD1, H1-7, ZNF641, ANP32D, C12orf54, OR8S1, OR5BS1P, LALBA, KANSL2, SNORA2C, MIR1291, SNORA2A, SNORA2B, CCNT1, TEX49, ADCY6, MIR4701, ADCY6-DT, CACNB3, DDX23, RND1, CCDC65, FKBP11, ARF3, WNT10B, WNT1, DDN, DDN-AS1, PRKAG1, KMT2D, RHEBL1, DHH, LMBR1L, TUBA1B, TUBA1B-AS1, TUBA1A, TUBA1C, TROAP-AS1, PRPH, TROAP, C1QL4, DNAJC22, SPATS2, LOC100335030, KCNH3, MCRS1, FAM186B, PRPF40B, FMNL3, TMBIM6, NCKAP5L, BCDIN3D-AS1, BCDIN3D, FAIM2, LINC02396, LINC02395, AQP2, AQP5-AS1, AQP5, AQP6, RACGAP1, ASIC1, SMARCD1, GPD1, COX14, CERS5, LIMA1, MIR1293, FAM186A, LARP4, SNORD133, DIP2B, ATF1, TMPRSS12, TMT1A, HIGD1C, SLC11A2, LETMD1, CSRNP2, TFCP2, POU6F1, DAZAP2, SMAGP, BIN2, CELA1, GALNT6, SLC4A8, SCN8A, TMDD1, FIGNL2, FIGNL2-DT, ANKRD33, ACVRL1, ACVR1B, TAMALIN-AS1, TAMALIN, NR4A1, NR4A1AS, ATG101, SMIM41, OR7E47P, KRT80, LINC02874, LINC00592, KRT7, KRT7-AS, KRT87P, KRT86, KRT81, KRT83, KRT85, KRT84, KRT82, KRT75, KRT6B, KRT6C, KRT6A, KRT5, KRT71, KRT74, KRT72, KRT73, KRT73-AS1, KRT2, KRT1, KRT77, KRT76, KRT3, KRT4, KRT79, KRT78, KRT8, MIR9898, KRT18, EIF4B, TNS2-AS1, TNS2, MIR6757, SPRYD3, IGFBP6, SOAT2, CSAD, ZNF740, ITGB7, RARG, LOC122455340, MFSD5, ESPL1, PFDN5, MYG1, AAAS, SP7, SP1, AMHR2, PRR13, PCBP2, PCBP2-OT1, MAP3K12, TARBP2, NPFF, ATF7-NPFF, ATF7, LOC100652999, ATP5MC2, CALCOCO1, CISTR, HOXC13-AS, HOXC13, HOXC12, HOTAIR, HOXC11, HOXC-AS3, HOXC10, MIR196A2, HOXC-AS2, HOXC-AS1, HOXC9, HOXC8, HOXC6, HOXC5, HOXC4, MIR615, FLJ12825, LOC100240735, LOC100240734, FAM242C, LINC02381, SMUG1, CBX5, MIR3198-2, SCAT2, HNRNPA1, NFE2, COPZ1, MIR148B, GPR84-AS1, GPR84, ZNF385A, ITGA5, GTSF1, NCKAP1L, PDE1B, PPP1R1A, GLYCAM1, LACRT, DCD, MUCL1, TESPA1, NEUROD4, OR9K2, OR10A7, OR6C74, OR6C6, OR6C1, OR6C3, OR6C75, OR6C65, OR6C76, OR6C2, OR6C70, OR6C68, OR6C4, OR2AP1, OR10P1, TMT1B, ITGA7, BLOC1S1-RDH5, BLOC1S1, RDH5, CD63, CD63-AS1, GDF11, SARNP, ORMDL2, DNAJC14, TMEM198B, MMP19, PYM1, DGKA, PMEL, CDK2, RAB5B, SUOX, IKZF4, LOC105369781, RPS26, ERBB3, PA2G4, RPL41, ZC3H10, ESYT1, MYL6B, MYL6, SMARCC2, RNF41, NABP2, SLC39A5, ANKRD52, COQ10A, CS, CNPY2, PAN2, IL23A, STAT2, APOF, TIMELESS, MIP, SPRYD4, GLS2, SNORA105C, RBMS2, BAZ2A, ATP5F1B, SNORD59B, SNORD59A, PTGES3, NACA, PRIM1, HSD17B6, SDR9C7, RDH16, GPR182, ZBTB39, TAC3, MYO1A, NEMP1, NAB2, STAT6, LRP1, LRP1-AS, MIR1228, NXPH4, SHMT2, NDUFA4L2, STAC3, R3HDM2, INHBC, INHBE, GLI1, ARHGAP9, MARS1, MIR6758, DDIT3, LOC128125814, MIR616, MBD6, DCTN2, KIF5A, PIP4K2C, DTX3, ARHGEF25, LOC101927583, SLC26A10P, B4GALNT1, OS9, AGAP2, AGAP2-AS1, TSPAN31, CDK4, MIR6759, MARCHF9, CYP27B1, METTL1, EEF1AKMT3, TSFM, AVIL, CTDSP2, MIR26A2, LOC283387, GIHCG, ATP23, LINC02403, LINC02388, LOC100506869, LRIG3, LRIG3-DT, SLC16A7, TAFA2, MIR6125, USP15, MON2, LINC01465, MIRLET7I, PPM1H | arr[GRCh38] 12q12q14.2(44,844,831_63,135,022)x3 |
| 1  Brain | cn-LOH | 12 | q14.2 | q24.33 | 70,192 | AVPR1A, DPY19L2, RXYLT1, RXYLT1-AS1, MIR10527, SRGAP1, KICS2, C12orf56, XPOT, TBK1, RASSF3, MIR548C, MIR548Z, GNS, TBC1D30, LINC02389, LINC02231, WIF1, LEMD3, MSRB3, MSRB3-AS1, LOC105369187, LINC02454, RPSAP52, HMGA2, HMGA2-AS1, MIR6074, LINC02425, LLPH, LLPH-DT, TMBIM4, IRAK3, MIR6502, HELB, GRIP1, LOC102724421, CAND1, LINC02420, LINC02408, LINC02442, DYRK2, LINC02421, LINC01479, IFNG-AS1, IFNG, IL26, IL22, MDM1, LINC02384, RAP1B, SNORA70G, LOC100507250, NUP107, SLC35E3, SZRD1P1, MDM2, CPM, CPSF6, MIR1279, LYZ, YEATS4, FRS2, SNORA113, MIR3913-1, MIR3913-2, CCT2, LRRC10, BEST3, LOC101928002, RAB3IP, MYRFL, PRANCR, CNOT2, KCNMB4, PTPRB, PTPRR, TSPAN8, LGR5, ZFC3H1, THAP2, TMEM19, RAB21, TBC1D15, MRS2P2, TPH2, TRHDE-AS1, TRHDE, LINC02444, LINC02882, ATXN7L3B, KCNC2, LOC100130268, CAPS2, GLIPR1L1, GLIPR1L2, GLIPR1, KRR1, PHLDA1, NAP1L1, LNCOG, BBS10, OSBPL8, ZDHHC17, CSRP2, E2F7, NAV3, LINC02424, SYT1, MIR1252, PAWR, PPP1R12A, PPP1R12A-AS1, OTOGL, PTPRQ, MYF6, MYF5, LINC01490, LIN7A, MIR617, MIR618, ACSS3, MIR4699, PPFIA2, PPFIA2-AS1, LINC02426, CCDC59, METTL25, TMTC2, SLC6A15, TSPAN19, LRRIQ1, ALX1, LINC02820, RASSF9, NTS, MGAT4C, MIR3059, LOC105369879, LINC02258, MKRN9P, C12orf50, RLIG1, CEP290, TMTC3, KITLG, LINC02458, DUSP6, POC1B, GALNT4, POC1B-GALNT4, POC1B-AS1, ATP2B1, ATP2B1-AS1, LINC02399, LINC02392, LINC00615, CCER1, EPYC, KERA, LUM, DCN, LINC02823, LINC01619, BTG1, BTG1-DT, CLLU1-AS1, CLLU1, LINC02397, PLEKHG7, EEA1, LOC643339, LINC02412, NUDT4, UBE2N, MRPL42, SOCS2-AS1, SOCS2, CRADD, CRADD-AS1, LOC105369911, PLXNC1, CEP83, CEP83-DT, MIR5700, TMCC3, MIR7844, MIR492, KRT19P2, NDUFA12, NR2C1, FGD6, VEZT, MIR331, MIR3685, METAP2, USP44, PGAM1P5, NTN4, SNRPF-DT, LINC02410, SNRPF, CCDC38, AMDHD1, HAL, LTA4H, ELK3, CDK17, CFAP54, NEDD1, LINC02409, RMST, MIR1251, MIR135A2, PAFAH1B2P2, MIR4495, MIR4303, SLC9A7P1, LINC02453, TMPO-AS1, TMPO, SLC25A3, SNORA53, IKBIP, APAF1, ANKS1B, LOC101928937, GARIN6, BLTP3B, GOLGA2P5, MIR1827, ACTR6, DEPDC4, SCYL2, SLC17A8, NR1H4, GAS2L3, ANO4, SLC5A8, UTP20, ARL1, SPIC, MYBPC1, CHPT1, SYCP3, GNPTAB, DRAM1, WASHC3, NUP37, PARPBP, PMCH, IGF1, LINC00485, PAH, ASCL1, C12orf42, C12orf42-AS1, LOC105369945, LINC02401, STAB2, NT5DC3, TTC41P, HSP90B1, MIR3652, UQCC6, TDG, GLT8D2, HCFC2, NFYB, TXNRD1, EID3, CHST11, MIR3922, SLC41A2, NOPCHAP1, ALDH1L2, LOC414300, WASHC4, APPL2, C12orf75-AS1, C12orf75, CASC18, NUAK1, CKAP4, TCP11L2, POLR3B, LOC100287944, RFX4, LOC100505978, RIC8B, TMEM263, MTERF2, CRY1, ABTB3, PWP1, PRDM4, PRDM4-AS1, ASCL4, LOC728739, WSCD2, CMKLR1, LINC01498, FICD, SART3, ISCU, TMEM119, SELPLG, MIR4496, CORO1C, SSH1, MIR619, DAO, SVOP, USP30, USP30-AS1, ALKBH2, UNG, ACACB, FOXN4, MYO1H, LINC01486, KCTD10, UBE3B, MMAB, MVK, FAM222A, FAM222A-AS1, TRPV4, MIR4497, GLTP, TCHP, GIT2, ANKRD13A, C12orf76, IFT81, ATP2A2, ANAPC7, ARPC3, GPN3, FAM216A, VPS29, RAD9B, PPTC7, TCTN1, HVCN1, PPP1CC, CCDC63, MYL2, LINC01405, LINC01404, CUX2, MIR6760, PHETA1, SH2B3, ATXN2, ATXN2-AS, BRAP, ACAD10, ALDH2, MIR6761, MAPKAPK5-AS1, MAPKAPK5, TMEM116, ERP29, NAA25, MIR3657, TRAFD1, HECTD4, MIR6861, RPL6, PTPN11, RPH3A, MIR1302-1, OAS1, OAS3, OAS2, DTX1, RASAL1, CFAP73, DDX54, MIR7106, RITA1, IQCD, TPCN1, MIR6762, SLC8B1, PLBD2, SDS, SDSL, LHX5, LHX5-AS1, LINC01234, RBM19, LINC02459, TBX5, TBX5-AS1, TBX3, MED13L, MIR620, MIR4472-2, LINC00173, MAP1LC3B2, SPRING1, RNFT2, HRK, FBXW8, LOC100506551, TESC, TESC-AS1, FBXO21, NOS1, KSR2, RFC5, WSB2, VSIG10, PEBP1, TAOK3, SUDS3, LINC02423, LINC02440, LINC02439, SRRM4, LOC105370024, HSPB8, LINC00934, CCDC60, TMEM233, PRKAB1, CIT, MIR1178, BICDL1, RAB35, RAB35-AS1, GCN1, MIR4498, RPLP0, PXN-AS1, PXN, RNU4-2, SIRT4, RNU4-1, PLA2G1B, MSI1, COX6A1, TRIAP1, GATC, SRSF9, DYNLL1, NRAV, COQ5, RNF10, LOC128071547, POP5, CABP1-DT, CABP1, MLEC, UNC119B, MIR4700, ACADS, SPPL3, XLOC_009911, HNF1A-AS1, HNF1A, C12orf43, OASL, P2RX7, P2RX4, CAMKK2, ANAPC5, RNF34, KDM2B, MIR7107, KDM2B-DT, ORAI1, MORN3, TMEM120B, RHOF, LINC01089, SETD1B, HPD, PSMD9, CFAP251, BCL7A, LINC02985, MLXIP, LRRC43, IL31, B3GNT4, DIABLO, LOC128125816, LOC101593348, VPS33A, CLIP1, CLIP1-AS1, ZCCHC8, RSRC2, KNTC1, MIR9902-1, HCAR2, MIR9902-2, HCAR3, HCAR1, DENR, CCDC62, HIP1R, VPS37B, ABCB9, OGFOD2, ARL6IP4, PITPNM2, MIR4304, PITPNM2-AS1, MPHOSPH9, MTRFR, CDK2AP1, SBNO1, MIR8072, SBNO1-AS1, KMT5A, RILPL2, SNRNP35, RILPL1, MIR3908, TMED2-DT, TMED2, DDX55, SNORA9B, EIF2B1, GTF2H3, TCTN2, ATP6V0A2, DNAH10, CCDC92, ZNF664, ZNF664-RFLNA, RFLNA, NCOR2, MIR6880, SCARB1, UBC, MIR5188, DHX37, BRI3BP, THRIL, AACS, TMEM132B, LINC00939, LINC02826, LINC02359, LINC02825, LINC02350, LINC02347, LOC100996671, LINC02824, LINC00944, LINC00943, LINC02372, LINC02405, LINC02376, LINC02375, LINC02411, LOC105370068, LINC02393, LINC00508, LINC00507, LINC02441, LINC02369, LINC02368, TMEM132C, MIR3612, SLC15A4, LOC100128276, GLT1D1, TMEM132D, TMEM132D-AS1, TMEM132D-AS2, LINC02418, LINC02419, FZD10-AS1, FZD10, PIWIL1, RIMBP2, STX2, RAN, ADGRD1, ADGRD1-AS1, LINC01257, LINC02415, LINC02370, LINC02414, SFSWAP, MMP17, ULK1, PUS1, EP400, SNORA49, EP400P1, DDX51, NOC4L, LINC02361, GALNT9, GALNT9-AS1, LOC101928416, FBRSL1, MIR6763, LRCOL1, P2RX2, POLE, PXMP2, PGAM5, ANKLE2, GOLGA3, CHFR, CHFR-DT, ZNF605, ZNF26, ZNF84-DT, ZNF84, ZNF140, ZNF891, ZNF10, ZNF268 | arr[GRCh38] 12q14.2q24.33(63,009,396_133,201,580)x2 hmz |
| 1  Brain | Gain | 13 | q11 | q34 | 95,480 | ANKRD20A9P, LINC00408, LINC00442, LOC107984132, TUBA3C, LOC101928697, ANKRD26P3, LINC00421, TPTE2, TPTE2-AS1, MPHOSPH8, PSPC1, ZMYM5, ZMYM2, LINC01072, GJA3, GJB2, GJB6, CRYL1, MIR4499, IFT88, IL17D, EEF1AKMT1, XPO4, LINC00367, LATS2, SAP18, SKA3, MRPL57, LINC01046, LOC101928764, MIPEPP3, LINC00539, GRK6P1, ZDHHC20, MICU2, FGF9, LINC00424, LINC00540, LINC00621, BASP1P1, SGCG, SACS, SACS-AS1, LINC00327, TNFRSF19, MIPEP, PCOTH, C1QTNF9B, ANKRD20A19P, SPATA13, MIR2276, SPATA13-AS1, C1QTNF9, LINC00566, PARP4, LOC105370295, TPTE2P6, ATP12A, RNF17, CENPJ, TPTE2P1, PABPC3, AMER2, LINC00463, LINC01053, MTMR6, NUP58, ATP8A2, SHISA2, LINC00415, RNF6, CDK8, WASF3, GPR12, USP12, USP12-AS1, USP12-DT, LINC00412, RPL21, SNORD102, SNORA27, RASL11A, GTF3A, MTIF3, LNX2, POLR1D, GSX1, PLUT, PDX1, LINC00543, CDX2, URAD, FLT3, PAN3-AS1, PAN3, FLT1, POMP, SLC46A3, MTUS2, MTUS2-AS1, SLC7A1, LOC102723345, UBL3, LINC00297, LINC00572, LINC00544, LINC00365, LINC00384, KATNAL1, LINC00426, LINC01058, UBE2L5, HMGB1, USPL1, ALOX5AP, LINC00398, LINC00545, TEX26-AS1, MEDAG, TEX26, HSPH1, B3GLCT, RXFP2, EEF1DP3, FRY-AS1, FRY, ZAR1L, BRCA2, N4BP2L1, N4BP2L2, MICOS10P1, N4BP2L2-IT2, PDS5B, LINC00423, KL, STARD13, STARD13-AS, LINC02344, RFC3, LINC02343, LINC00457, NBEA, MAB21L1, LINC00445, DCLK1, SOHLH2, CCDC169-SOHLH2, CCDC169, SPART, SPART-AS1, CCNA1, SERTM1, RFXAP, SMAD9, ALG5, EXOSC8, SUPT20H, CSNK1A1L, LINC01048, LINC00547, POSTN, TRPC4, LINC00571, UFM1, LINC00437, LINC00366, FREM2, FREM2-AS1, STOML3, PROSER1, NHLRC3, LHFPL6, COG6, MIR4305, LINC00332, LINC00548, LINC00598, FOXO1, MIR320D1, MRPS31, SLC25A15, TPTE2P5, MIR621, SUGT1P3, ELF1, WBP4, MIR3168, KBTBD6, LOC101929140, KBTBD7, MTRF1, NAA16, OR7E37P, RGCC, VWA8, MIR5006, VWA8-AS1, DGKH, AKAP11, LINC02341, TNFSF11, FAM216B, LINC01050, LINC00428, EPSTI1, DNAJC15, LINC00400, ENOX1, ENOX1-AS2, CCDC122, LACC1, NRAD1, LINC00390, SMIM2-AS1, SMIM2, SMIM2-IT1, MIR8079, SERP2, TUSC8, TSC22D1, TSC22D1-AS1, LINC00407, LINC00330, NUFIP1, GPALPP1, LOC101929259, GTF2F2, KCTD4, TPT1, SNORA31B, SNORA31, TPT1-AS1, SLC25A30, SLC25A30-AS1, COG3, ERICH6B, LINC01055, CBY2, SIAH3, ZC3H13, CPB2-AS1, CPB2, LCP1, LRRC63, LINC00563, RUBCNL, LINC01198, LOC112268117, LRCH1, ESD, HTR2A, HTR2A-AS1, LINC00562, SUCLA2, NUDT15, MED4, MED4-AS1, ITM2B, RB1-DT, RB1, LPAR6, RCBTB2, LINC01077, LINC00462, CYSLTR2, FNDC3A, MLNR, LOC105370203, CDADC1, CAB39L, SETDB2, SETDB2-PHF11, PHF11, RCBTB1, ARL11, EBPL, KPNA3, CTAGE10P, SPRYD7, DLEU2, MIR3613, TRIM13, KCNRG, MIR16-1, MIR15A, DLEU1, ST13P4, DLEU1-AS1, DLEU7, DLEU7-AS1, RNASEH2B-AS1, RNASEH2B, GUCY1B2, C13orf42, FAM124A, SERPINE3, MIR5693, INTS6, INTS6-AS1, MIR4703, WDFY2, DHRS12, TMEM272, CCDC70, ATP7B, ALG11, UTP14C, NEK5, LOC101929657, NEK3, MRPS31P5, LINC02333, THSD1, VPS36, CKAP2, LINC00345, TPTE2P3, HNRNPA1L2, SUGT1-DT, SUGT1, CNMD, MIR759, PCDH8, OLFM4, LINC01065, LINC00558, LINC00458, MIR1297, MIR5007, PRR20A, PRR20B, PRR20C, PRR20D, PRR20E, PCDH17, LINC02338, LINC00374, DIAPH3, DIAPH3-AS1, DIAPH3-AS2, LINC00434, TDRD3, LINC00378, LINC01442, MIR3169, PCDH20, LINC02339, LINC00358, LINC01075, LINC01074, LINC00459, LINC00448, LINC00376, LINC00395, OR7E156P, LOC112267897, LOC102723968, LOC647264, LINC00355, LINC01052, MIR548X2, MIR4704, PCDH9, PCDH9-AS2, PCDH9-AS3, PCDH9-AS4, LINC00364, LINC00550, LINC00383, KLHL1, ATXN8OS, LINC00348, DACH1, MZT1, BORA, DIS3, PIBF1, KLF5, LINC00393, LINC00392, KLF12, LINC00402, LOC100288208, LINC00381, LINC00347, CTAGE11P, LINC01078, TBC1D4, COMMD6, UCHL3, LMO7-AS1, LMO7, LMO7DN, LMO7DN-IT1, LINC00561, KCTD12, BTF3P11, ACOD1, CLN5, FBXL3, MYCBP2, MYCBP2-AS1, MYCBP2-AS2, LOC127898562, SCEL, SCEL-AS1, LOC100129307, SLAIN1, MIR3665, EDNRB-AS1, EDNRB, LINC01069, LINC00446, OBI1-AS1, LOC780529, POU4F1, OBI1, LINC00331, RBM26, RBM26-AS1, NDFIP2-AS1, NDFIP2, LINC01068, LINC01038, LINC00382, LINC01080, SPRY2, LINC00377, LINC00564, SLITRK1, LINC00333, SNORA107, LINC00375, LINC00351, SLITRK6, LINC00430, MIR4500HG, MIR4500, SLITRK5, LINC00397, LINC00373, LINC00433, LINC00560, LINC01047, LINC00440, LINC01040, LINC00353, LINC00559, MIR622, LINC01049, LINC00410, LINC00380, LINC00379, MIR17HG, MIR17, MIR18A, MIR19A, MIR20A, MIR19B1, MIR92A1, GPC5, GPC5-AS2, MIR548AS, GPC5-AS1, LINC00363, GPC6, GPC6-AS2, GPC6-AS1, DCT, TGDS, GPR180, LINC00391, SOX21, SOX21-AS1, LOC112268114, LOC101927284, LINC00557, ABCC4, SNORD13G, CLDN10, CLDN10-AS1, DZIP1, DNAJC3-DT, DNAJC3, UGGT2, HS6ST3, MIR4501, LINC00359, OXGR1, MBNL2, LINC00456, RAP2A, IPO5, FARP1, RNF113B, MIR3170, STK24, STK24-AS1, SLC15A1, DOCK9, DOCK9-AS1, DOCK9-DT, UBAC2-AS1, UBAC2, GPR18, GPR183, FKSG29, MIR623, LINC01232, LINC00449, TM9SF2, LINC01039, CLYBL, MIR4306, CLYBL-AS2, CLYBL-AS1, CLYBL-AS3, ZIC5, ZIC2, LINC00554, PCCA-DT, PCCA, PCCA-AS1, GGACT, TMTC4, NALCN-AS1, LINC00411, NALCN, ITGBL1, FGF14, MIR2681, MIR4705, FGF14-IT1, FGF14-AS1, FGF14-AS2, TPP2, METTL21C, CCDC168, TEX30, POGLUT2, BIVM, BIVM-ERCC5, ERCC5, METTL21EP, SLC10A2, LINC01309, DAOA-AS1, DAOA, LINC00343, LINC00460, EFNB2, ARGLU1, LINC00551, LINC00443, NALF1, SNORD31B, MIR1267, NALF1-IT1, LIG4, ABHD13, TNFSF13B, MYO16, MYO16-AS2, MYO16-AS1, LINC00370, LINC00399, LINC00676, IRS2, LINC00396, LINC03032, COL4A1, COL4A2, MIR8073, COL4A2-AS2, COL4A2-AS1, RAB20, NAXD-AS1, NAXD, CARS2, ING1, LINC00567, LOC105370362, PRECSIT, ANKRD10, LINC00431, LINC00368, ARHGEF7-AS2, ARHGEF7, ARHGEF7-AS1, LOC101060553, TEX29, LINC02337, LINC00354, SOX1-OT, SOX1, LOC100506016, LINC01070, LOC101928730, LINC01043, LINC01044, SPACA7, TUBGCP3, ATP11AUN, ATP11A, ATP11A-AS1, MCF2L-AS1, MCF2L, F7, F10, F10-AS1, PROZ, PCID2, CUL4A, MIR8075, LAMP1, GRTP1, GRTP1-AS1, ADPRHL1, DCUN1D2, TMCO3, TFDP1, ATP4B, GRK1, LINC00552, TMEM255B, GAS6-AS1, GAS6, GAS6-DT, LINC00454, LINC00452, SWINGN, C13orf46, RASA3, RASA3-IT1, CFAP97D2, CDC16, MIR548AR, MIR4502, UPF3A, CHAMP1, LINC01054 | arr[GRCh38] 13q11q34(18,862,147_114,342,258)x4 |
| 1  Brain | Gain | 15 | q15.3 | q26.3 | 57,956 | FRMD5, GOLM2, CTDSPL2, EIF3J-DT, EIF3J, SPG11, PATL2, B2M, MIR10393, LOC100419583, TRIM69, SORD2P, TERB2, SORD, DUOX2, DUOXA2, DUOXA1, DUOX1, SHF, SLC28A2-AS1, SLC28A2, GATM, SPATA5L1, C15orf48, MIR147B, SLC30A4, HMGN2P46, SNORA41B, BLOC1S6, SQOR, LOC105370802, SEMA6D, LINC01491, SLC24A5, MYEF2, CTXN2, CTXN2-AS1, SLC12A1, DUT, FBN1, FBN1-DT, CEP152, SHC4, EID1, SECISBP2L, COPS2, GALK2, NDUFAF4P1, MIR4716, FAM227B, FGF7, DTWD1, ATP8B4, SLC27A2, HDC, GABPB1, GABPB1-IT1, GABPB1-AS1, MIR4712, USP8, USP50, TRPM7, LOC128092252, SPPL2A, AP4E1, DCAF13P3, MIR4713HG, TNFAIP8L3, CYP19A1, MIR4713, MIR7973-2, MIR7973-1, GLDN, DMXL2, SCG3, LYSMD2, TMOD2, TMOD3, LOC100422556, LEO1, MAPK6-DT, MAPK6, BCL2L10, GNB5, CERNA1, MYO5C, MIR1266, MYO5A, ARPP19, ATOSA, ONECUT1, LINC02490, WDR72, UNC13C, LOC105370829, RSL24D1, RAB27A, PIGBOS1, PIGB, DNAAF4-CCPG1, CCPG1, MIR628, PIERCE2, DNAAF4, PYGO1, PRTG, NEDD4, RFX7, TEX9, MNS1, ZNF280D, TCF12-DT, LINC03065, TCF12, SNORD13D, LINC00926, LINC01413, CGNL1, MYZAP, GCOM1, POLR2M, ALDH1A2, ALDH1A2-AS1, AQP9, LIPC, LIPC-AS1, ADAM10, HSP90AB4P, MINDY2-DT, MINDY2, SLTM, RNF111, CCNB2, MYO1E, MIR2116, LDHAL6B, FAM81A, GCNT3, GTF2A2, BNIP2, FOXB1, ANXA2, ICE2, RORA-AS1, RORA, RORA-AS2, LINC02349, VPS13C, VPS13C-DT, C2CD4A, C2CD4B, LOC107984784, GOLGA2P11, MIR8067, MIR6085, TLN2, MGC15885, MIR190A, TPM1, TPM1-AS, LACTB, RPS27L, RAB8B, APH1B, CA12, LINC02568, USP3, USP3-AS1, FBXL22, HERC1, MIR422A, DAPK2, LOC101928988, CIAO2A, SNX1, SNX22, PPIB, CSNK1G1, PCLAF, TRIP4, ZNF609, OAZ2, RBPMS2, MIR1272, PIF1, PLEKHO2, ANKDD1A, SPG21, MTFMT, SLC51B, RASL12, KBTBD13, UBAP1L, PDCD7, CLPX, CILP, PARP16, SNORA24B, RNU5A-1, RNU5B-1, IGDCC3, IGDCC4, DPP8, HACD3, INTS14, SLC24A1, DENND4A, SNORD13E, MIR4511, RAB11A, MEGF11, MIR4311, DIS3L-AS1, DIS3L, TIPIN, SCARNA14, MAP2K1, SNAPC5, MIR4512, RPL4, SNORD18C, SNORD18B, SNORD16, SNORD18A, ZWILCH, LCTL, LINC01169, SMAD6, SMASR, SMAD3-DT, SMAD3, AAGAB, IQCH, IQCH-AS1, C15orf61, MAP2K5, SKOR1, SKOR1-AS1, RNU6-1, PIAS1, CALML4, CLN6, FEM1B, ITGA11, CORO2B, ANP32A, MIR4312, ANP32A-IT1, SPESP1, NOX5, EWSAT1, GLCE, PAQR5-DT, PAQR5, KIF23-AS1, KIF23, RPLP1, DRAIC, PCAT29, LINC00593, TLE3, MIR629, LINC02205, LINC02204, SALRNA3, SALRNA2, UACA, LARP6, LRRC49, THAP10, THSD4, CT62, THSD4-AS1, THSD4-AS2, NR2E3, MYO9A, SENP8, GRAMD2A, PKM, PARP6, CELF6, HEXA, HEXA-AS1, TMEM202, TMEM202-AS1, ARIH1, MIR630, LINC02259, GOLGA6B, HIGD2B, BBS4, ADPGK, ADPGK-AS1, NEO1, MIR12135, HCN4, REC114, NPTN, NPTN-IT1, CD276, INSYN1, INSYN1-AS1, TBC1D21, LOXL1-AS1, LOXL1, STOML1, PML, GOLGA6A, COMMD4P2, LOC283731, ISLR2, ISLR, STRA6, CCDC33, CYP11A1, PPIAP46, LINC02255, SEMA7A, MIR6881, UBL7, UBL7-DT, ARID3B, CLK3, EDC3, CYP1A1, CYP1A2, CSK, MIR4513, LMAN1L, CPLX3, ULK3, MIR6882, SCAMP2, MPI, FAM219B, COX5A, RPP25, SCAMP5, PPCDC, C15orf39, LOC105376731, GOLGA6C, GOLGA6D, COMMD4, NEIL1, MIR631, MAN2C1, SIN3A, PTPN9, SNUPN, IMP3, SNX33, CSPG4, ODF3L1, DNM1P35, MIR4313, UBE2Q2, FBXO22, NRG4, TMEM266, LOC101929439, ETFA, TYRO3P, ISL2, SCAPER, MIR3713, RCN2, PSTPIP1, TSPAN3, PEAK1, LINC00597, HMG20A, LOC101929457, LINGO1, LINGO1-AS1, LINGO1-AS2, GOLGA6FP, COMMD4P1, LOC91450, TBC1D2B, SH2D7, CIB2, IDH3A, ACSBG1, DNAJA4, SKIC8, CRABP1, IREB2, HYKK, PSMA4, CHRNA5, CHRNA3, CHRNB4, LOC646938, ADAMTS7, MORF4L1, CTSH, RASGRF1, LOC100129540, ANKRD34C-AS1, MIR184, ANKRD34C, TMED3, MINAR1, MTHFS, ST20-MTHFS, ST20, ST20-AS1, BCL2A1, ZFAND6, FAH, CTXND1, LINC00927, ARNT2-DT, ARNT2, LOC101929586, MIR5572, ABHD17C, CEMIP, MIR549A, MESD, MIR4514, TLNRD1, LOC128071545, CFAP161, IL16, STARD5, TMC3-AS1, TMC3, MEX3B, LINC01583, EFL1, SAXO2, ADAMTS7P1, GOLGA6L10, UBE2Q2P2, GOLGA6L9, LOC102724034, GOLGA2P10, GOLGA6L17P, RPS17, CPEB1, CPEB1-AS1, AP3B2, LOC338963, ACTG1P17, SNHG21, FSD2, SCARNA15, WHAMM, HOMER2, RAMAC, C15orf40, BTBD1, MIR4515, TM6SF1, HDGFL3, BNC1, SH3GL3, ADAMTSL3, EFL1P1, DNM1P41, UBE2Q2P16, LOC440300, GOLGA2P7, GOLGA6L4, LOC103171574, LOC102724135, GOLGA6L5P, UBE2Q2P1, LINC00933, ZSCAN2, SCAND2P, WDR73, NMB, SEC11A, ZNF592, ALPK3, SLC28A1, PDE8A, LOC727751, LOC101929479, GOLGA6L3P, MIR7706, AKAP13, LINC02883, KLHL25, MIR1276, MIR548AP, AGBL1, LINC01584, AGBL1-AS1, LOC105370954, LOC102724452, LINC00052, NTRK3, MIR11181, NTRK3-AS1, MRPL46, MRPS11, DET1, LINC01586, MIR1179, MIR7-2, MIR3529, AEN, ISG20, ACAN, HAPLN3, MFGE8, CARMAL, ABHD2, RLBP1, FANCI, POLG, POLGARF, MIR6766, MIR9-3HG, MIR9-3, LOC105371031, RHCG, LINC00928, TICRR, KIF7, PLIN1, PEX11A, WDR93, MESP1, MESP2, ANPEP, AP3S2, ARPIN-AP3S2, MIR5094, MIR5009, ARPIN, ZNF710, MIR3174, ZNF710-AS1, IDH2, IDH2-DT, SEMA4B, CIB1, GDPGP1, TTLL13, NGRN, GABARAPL3, ZNF774, IQGAP1, CRTC3, CRTC3-AS1, LINC01585, BLM, FURIN, FES, MAN2A2, HDDC3, UNC45A, RCCD1-AS1, RCCD1, PRC1, PRC1-AS1, VPS33B, VPS33B-DT, SV2B, CRAT37, SLCO3A1, ST8SIA2, LOC104613533, C15orf32, LINC00930, FAM174B, ASB9P1, CHASERR, CHD2, MIR3175, RGMA, LOC105370980, LINC02207, LINC01579, LINC01580, LINC01581, MCTP2, LOC440311, LETR1, LINC00924, LOC105369212, NR2F2-AS1, NR2F2, MIR1469, SPATA8-AS1, SPATA8, LINC02253, LINC02254, LOC101927310, LINC00923, ARRDC4, LINC02251, LINC01582, LINC02351, FAM169BP, IRAIN, IGF1R, MIR4714, PGPEP1L, LUNAR1, SYNM, TTC23, LRRC28, HSP90B2P, MEF2A, LYSMD4, DNM1P46, LOC400464, ADAMTS17, SPATA41, CERS3-AS1, CERS3, PRKXP1, LOC102723335, LINS1, ASB7, GCAWKR, ALDH1A3, ALDH1A3-AS1, LRRK1, CHSY1, SELENOS, SNRPA1, PCSK6, LOC100507472, PCSK6-AS1, LINC02348, TM2D3, TARS3, UBE2Q2P13, OR4F6, OR4F15, OR4F13P | arr[GRCh38] 15q15.3q26.3(43,898,705_101,854,764)x3 |
| 1  Brain | Gain | 16 | p13.3 | p11.2 | 32,524 | POLR3K, SNRNP25, RHBDF1, MPG, NPRL3, HBZ, HBM, HBA2, HBA1, HBQ1, LUC7L, FAM234A, RGS11, ARHGDIG, PDIA2, AXIN1, MRPL28, PGAP6, LOC100134368, NME4, DECR2, RAB11FIP3, LINC00235, CAPN15, MIR5587, MIR3176, PRR35, NHLRC4, PIGQ, RAB40C, WFIKKN1, METTL26, LOC100287175, MCRIP2, LOC105371038, WDR90, RHOT2, RHBDL1, STUB1-DT, STUB1, JMJD8, WDR24, FBXL16, METRN, ANTKMT, CCDC78, HAGHL, CIAO3, MSLN, MIR662, RPUSD1, CHTF18, GNG13, LMF1, LMF1-AS1, CEROX1, SOX8, SSTR5-AS1, SSTR5, C1QTNF8, CACNA1H, TPSG1, TPSB2, TPSAB1, TPSD1, UBE2I, BAIAP3, TSR3, GNPTG, UNKL, UQCC4, PERCC1, CCDC154, CLCN7, PTX4, TELO2, IFT140, TMEM204, LOC105371046, CRAMP1, JPT2, MAPK8IP3, MAPK8IP3-AS1, MIR3177, NME3, MRPS34, EME2, SPSB3, NUBP2, IGFALS, HAGH, FAHD1, MEIOB, LINC00254, LINC02124, HS3ST6, MSRB1, RPL3L, NDUFB10, RPS2, SNORA10, SNORA64, SNHG9, SNORA78, RNF151, TBL3, NOXO1, GFER, SYNGR3, ZNF598, NPW, NHERF2, NTHL1, TSC2, PKD1, MIR1225, PKD1-AS1, MIR6511B1, MIR4516, MIR3180-5, RAB26, SNHG19, SNORD60, TRAF7, CASKIN1, MLST8, BRICD5, PGP, E4F1, DNASE1L2, ECI1, RNPS1, MIR3677HG, MIR3677, MIR940, MIR4717, ABCA3, ABCA17P, CCNF, MIR6767, TEDC2, MIR6768, TEDC2-AS1, NTN3, TBC1D24, ATP6V0C, AMDHD2, CEMP1, MIR3178, PDPK1, LOC652276, FLJ42627, ERVK13-1, KCTD5, PRSS27, SRRM2-AS1, SRRM2, ELOB, PRSS33, SNORA3C, PRSS41, PRSS21, ZG16B, PRSS30P, PRSS22, FLYWCH2, FLYWCH1, KREMEN2, PAQR4, PKMYT1, GREP1, CLDN9, CLDN6, TNFRSF12A, HCFC1R1, THOC6, BICDL2, LOC100128770, MMP25, MMP25-AS1, IL32, ZSCAN10, ZNF205-AS1, ZNF205, ZNF213-AS1, ZNF213, CASP16P, OR1F1, OR1F2P, ZNF200, MEFV, LINC00921, ZNF263, TIGD7, ZNF75A, OR2C1, ZSCAN32, ZNF174, ZNF597, NAA60, MIR6126, C16orf90, CLUAP1, NLRC3, SLX4, DNASE1, TRAP1, CREBBP, LINC02861, ADCY9, SRL, LINC01569, TFAP4, GLIS2, GLIS2-AS1, PAM16, CORO7-PAM16, CORO7, VASN, DNAJA3, NMRAL1, HMOX2, CDIP1, C16orf96, UBALD1, MGRN1, MIR6769A, NUDT16L1, ANKS3, DNAAF8, ZNF500, SEPTIN12, SMIM22, ROGDI, GLYR1, UBN1, PPL, SEC14L5, NAGPA, NAGPA-AS1, C16orf89, ALG1, EEF2KMT, RBFOX1, LINC01570, MIR8065, LINC02152, TMEM114, METTL22, ABAT, TMEM186, PMM2, LOC100130283, CARHSP1, LITAFD, USP7, USP7-AS1, HAPSTR1, LINC02177, LINC01177, LINC01195, GRIN2A, ATF7IP2, LINC01290, EMP2, TEKT5, NUBP1, TVP23A, CIITA, DEXI, CLEC16A, SOCS1, TNP2, PRM3, PRM2, PRM1, MIR548H2, LOC105371083, RMI2, LOC400499, LOC101927131, LITAF, SNN, TXNDC11, ZC3H7A, BCAR4, RSL1D1, GSPT1, NPIPB2, TNFRSF17, SNX29, CPPED1, MIR4718, SHISA9, ERCC4, LINC02185, LINC02186, MRTFB, MIR193BHG, MIR193B, MIR365A, LINC02130, PARN, BFAR, PLA2G10, NPIPA3, NPIPA2, ABCC6P2, NOMO1, MIR3179-1, MIR3670-1, MIR3180-1, PKD1P3-NPIPA1, LOC100288162, MIR6511A1, MIR6770-1, NPIPA1, PDXDC1, MIR1972-1, NTAN1, RRN3, LOC100505915, PKD1P6-NPIPP1, MIR6511B2, MIR3180-4, NPIPA5, MPV17L, MPV17L-BMERB1, BMERB1, MARF1, MIR6506, MIR484, NDE1, MYH11, CEP20, ABCC1, ABCC6, NOMO3, MIR3179-2, MIR3670-2, MIR3180-2, PKD1P1, MIR6511A2, MIR6770-2, MIR6511A3, NPIPA7, XYLT1, LOC102723692, NPIPA8, PKD1P4-NPIPA8, MIR6511A4, NPIPA9, PKD1P5-LOC105376752, MIR6770-3, MIR3180-3, MIR3670-3, MIR3179-3, MIR3670-4, MIR3179-4, NOMO2, ABCC6P1, RPS15A, ARL6IP1, SMG1, TMC7, COQ7-DT, COQ7, ITPRIPL2, SYT17, CLEC19A, TMC5, GDE1, CCP110, VPS35L, KNOP1, IQCK, GPRC5B, GPR139, GP2, UMOD, PDILT, ACSM5, ACSM2A, ACSM2B, ACSM1, THUMPD1, ACSM3, ERI2, REXO5, DCUN1D3, LYRM1, DNAH3, LDAF1, ZP2, ANKS4B, CRYM, CRYM-AS1, SNX29P1, NPIPB3, LOC100190986, SMG1P3, MIR3680-1, SLC7A5P2, LOC101927814, METTL9, IGSF6, OTOA, RRN3P1, NPIPB4, UQCRC2, PDZD9, MOSMO, VWA3A, SDR42E2, EEF2K, POLR3E, CDR2, CDR2-DT, MFSD13B, RRN3P3, SMG1P1, NPIPB5, OTOAP1, HS3ST2, USP31, SCNN1G, SCNN1B, COG7, GGA2, EARS2, UBFD1, NDUFAB1, PALB2, DCTN5, PLK1, ERN2, CHP2, PRKCB, MIR1273H, LINC02194, CACNG3, RBBP6, TNRC6A, LINC01567, SLC5A11, ARHGAP17, LOC554206, LINC02175, LCMT1-AS1, LCMT1, LCMT1-AS2, AQP8, ZKSCAN2, ZKSCAN2-DT, LINC02191, HS3ST4, MIR548W, C16orf82, LINC02129, KDM8, NSMCE1, NSMCE1-DT, IL4R, IL21R, IL21R-AS1, GTF3C1, KATNIP, LOC100128079, GSG1L, XPO6, SBK1, NPIPB6, EIF3CL, MIR6862-1, NPIPB7, CLN3, APOBR, IL27, NUPR1, SGF29, SULT1A2, SULT1A1, NPIPB8, EIF3C, MIR6862-2, NPIPB9, ATXN2L, TUFM, MIR4721, SH2B1, ATP2A1, ATP2A1-AS1, RABEP2, CD19, NFATC2IP, MIR4517, SPNS1, LAT, RRN3P2, SNX29P2, NPIPB11, SMG1P6, BOLA2-SMG1P6, LOC606724, BOLA2, SLX1B, SLX1B-SULT1A4, SULT1A4, LOC388242, NPIPB12, SMG1P2, MIR3680-2, SPN, QPRT, C16orf54, ZG16, KIF22, MAZ, PRRT2, PAGR1, MVP, CDIPT, CDIPTOSP, SEZ6L2, ASPHD1, KCTD13, TMEM219, TAOK2, HIRIP3, INO80E, DOC2A, C16orf92, TLCD3B, LOC112694756, ALDOA, PPP4C, TBX6, YPEL3, YPEL3-DT, GDPD3, MAPK3, CORO1A, BOLA2B, SLX1A, SLX1A-SULT1A3, SULT1A3, LOC613038, NPIPB13, SMG1P5, CD2BP2, CD2BP2-DT, TBC1D10B, MYL11, SEPTIN1, ZNF48, ZNF771, SNORA80C, DCTPP1, SEPHS2, ITGAL, MIR4518, ZNF768, ZNF747, ZNF747-DT, ZNF764, ZNF688, ZNF785, ZNF689, PRR14, FBRS, LOC730183, SRCAP, SNORA30, TMEM265, PHKG2, CFAP119, RNF40, ZNF629, BCL7C, MIR4519, MIR762HG, MIR762, CTF1, FBXL19-AS1, FBXL19, ORAI3, SETD1A, HSD3B7, STX1B, STX4, ZNF668, ZNF646, PRSS53, VKORC1, BCKDK, KAT8, PRSS8, PRSS36, FUS, PYCARD, PYCARD-AS1, TRIM72, PYDC1, ITGAM, ITGAX, ITGAD, COX6A2, ZNF843, ARMC5, TGFB1I1, SLC5A2, RUSF1, AHSP, LINC02190, FRG2KP, YBX3P1, CLUHP3, KRBOX5, LOC107983990, VN1R3, ZNF267, HERC2P4, TP53TG3D, LOC390705 | arr[GRCh38] 16p13.3p11.2(35,881_32,559,611)x3~4 |
| 1  Brain | Gain | 16 | q12.1 | q24.3 | 39,872 | TENT4B, ADCY7, MIR6771, BRD7, LINC02178, NKD1, SNX20, LOC101927272, NOD2, CYLD-AS1, CYLD, MIR3181, LINC02168, LINC02128, LINC02127, SALL1, HNRNPA1L3, LINC01571, LINC00919, LINC02180, CASC22, TOX3, CASC16, LINC03064, CHD9NB, CHD9, MPHOSPH10P1, LOC102723373, RBL2, AKTIP, RPGRIP1L, FTO, FTO-IT1, LINC02169, IRX3, LINC02140, LOC101927480, CRNDE, IRX5, IRX6, MMP2-AS1, MMP2, LPCAT2, CAPNS2, SLC6A2, CES1P2, CES1P1, CES1, CES5A, GNAO1-DT, GNAO1, GNAO1-AS1, MIR3935, AMFR, NUDT21, OGFOD1, BBS2, MT4, MT3, MT2A, MT1L, MT1E, MT1M, MT1JP, MT1A, MT1DP, MT1B, MT1F, MT1G, MT1H, MT1IP, MT1X, NUP93-DT, NUP93, MIR138-2, SLC12A3, MIR6863, HERPUD1, CETP, NLRC5, CPNE2, PSME3IP1, RSPRY1, ARL2BP, PLLP, CCL22, CX3CL1, CCL17, CIAPIN1, COQ9, POLR2C, DOK4, CCDC102A, ADGRG5, ADGRG1, ADGRG3, DRC7, KATNB1, KIFC3, MIR6772, LOC388282, CNGB1, TEPP, ZNF319, USB1, MMP15, CFAP20, CSNK2A2, CCDC113, PRSS54, GINS3, NDRG4, SETD6, CNOT1, SNORA46, SNORA50A, SLC38A7, GOT2, APOOP5, LINC02141, CDH8, CDH11, LINC02126, LINC00922, CDH5, LINC00920, BEAN1, BEAN1-AS1, TK2, CKLF, CKLF-CMTM1, CMTM1, CMTM2, CMTM3, CMTM4, DYNC1LI2, DYNC1LI2-DT, TERB1, NAE1, CA7, PDP2, CDH16, RRAD, CIAO2B, CES2, CES3, CES4A, CBFB, PHAF1, B3GNT9, TRADD, FBXL8, HSF4, NOL3, MATCAP1, EXOC3L1, E2F4, ELMO3, MIR328, FBXL9P, TMEM208, FHOD1, SLC9A5, PLEKHG4, KCTD19, LRRC36, TPPP3, ZDHHC1, HSD11B2, ATP6V0D1, ATP6V0D1-DT, AGRP, LOC100505942, RIPOR1, CTCF-DT, CTCF, CARMIL2, ACD, PARD6A, ENKD1, C16orf86, GFOD2, RANBP10, TSNAXIP1, CENPT, THAP11, NUTF2, EDC4, NRN1L, PSKH1, CTRL, PSMB10, LCAT, SLC12A4, DPEP3, DPEP2, DPEP2NB, DDX28, DUS2, NFATC3, ESRP2, MIR6773, PLA2G15, SLC7A6, SLC7A6OS, PRMT7, SMPD3, ZFP90, CDH3, CDH1, TANGO6, HAS3, CHTF8, DERPC, UTP4, SNTB2, VPS4A, COG8, PDF, NIP7, TMED6, TERF2, CYB5B, MIR1538, NFAT5, SNORD13H, NQO1, NOB1, WWP2, MIR140, CLEC18A, PDXDC2P-NPIPB14P, MIR1972-2, PDPR, LOC400541, CLEC18C, LOC105371328, SMG1P7, EXOSC6, AARS1, DDX19B, DDX19A-DT, DDX19A, ST3GAL2, FCSK, COG4, SF3B3, SNORD111B, SNORD111, IL34, MTSS2, VAC14, VAC14-AS1, HYDIN, CMTR2, CALB2, LINC02136, TLE7, ZNF23, ZNF19, LOC105371335, CHST4, TAT-AS1, TAT, MARVELD3, PHLPP2, SNORA70D, AP1G1, SNORD71, ATXN1L, ZNF821, IST1, PKD1L3, DHODH, HP, HPR, TXNL4B, DHX38, PMFBP1, LINC01572, ZFHX3-AS1, ZFHX3, HCCAT5, LINC01568, PSMD7-DT, PSMD7, PDPR2P, NPIPB15, LOC105376772, CLEC18B, GLG1, RFWD3, MLKL, FA2H, WDR59, ZNRF1, LDHD, ZFP1, CTRB2, CTRB1, LOC100506281, BCAR1, CFDP1, TMEM170A, CHST6, CHST5, TMEM231, GABARAPL2, ADAT1, KARS1, TERF2IP, CPHXL2, DUXB, CPHXL, CNTNAP4, LINC02125, MIR4719, MON1B, SYCE1L, ADAMTS18, LINC02131, NUDT7, VAT1L, CLEC3A, WWOX, WWOX-AS1, MAF, MAFTRR, LINC01229, LINC01228, DYNLRB2-AS1, DYNLRB2, LINC01227, CDYL2, ARLNC1, CMC2, CENPN, ATMIN, C16orf46, GCSH, PKD1L2, BCO1, GAN, MIR4720, CMIP, MIR7854, MIR6504, LOC100129617, PLCG2, SDR42E1, HSD17B2, MPHOSPH6, CDH13, MIR8058, LOC101928446, LOC101928417, MIR3182, CDH13-AS2, HSBP1, MLYCD, OSGIN1, NECAB2, SLC38A8, MBTPS1, HSDL1, DNAAF1, TAF1C, ADAD2, LOC654780, KCNG4, WFDC1, ATP2C2, ATP2C2-AS1, MEAK7, COTL1, KLHL36, USP10, CRISPLD2, ZDHHC7, KIAA0513, CIBAR2, LINC02139, MIR12128, LINC00311, MIR5093, GSE1, GINS2, C16orf74, MIR1910, EMC8, LOC101928557, COX4I1, IRF8, MIR6774, LINC01082, LINC01081, LINC02135, LINC00917, FENDRR, FOXF1, MTHFSD, FLJ30679, FOXC2-AS1, FOXC2, FOXL1, LINC02189, LINC02188, LINC02181, LOC101928708, LOC101928682, C16orf95, C16orf95-DT, FBXO31, MAP1LC3B, ZCCHC14, ZCCHC14-DT, JPH3, KLHDC4, LOC102724467, SLC7A5, MIR6775, MIR11401, CA5A, BANP, LOC400553, LINC02182, LOC107984862, ZNF469, ZFPM1, MIR5189, ZFPM1-AS1, ZC3H18-AS1, ZC3H18, IL17C, CYBA, MVD, SNAI3-AS1, SNAI3, RNF166, CTU2, PIEZO1, MIR4722, LOC100289580, LOC339059, CDT1, APRT, GALNS, TRAPPC2L, PABPN1L, CBFA2T3, LOC101927793, LOC100129697, ACSF3, LINC00304, LINC02138, CDH15, SLC22A31, ZNF778, ANKRD11, LOC105371414, LOC128462377, LOC100287036, LOC101927817, SPG7, RPL13, SNORD68, CPNE7, DPEP1, CHMP1A, SPATA33, LINC02166, CDK10, SPATA2L, VPS9D1, VPS9D1-AS1, ZNF276, FANCA, SPIRE2, TCF25, MC1R, TUBB3, DEF8, SNORA119, CENPBD1P, AFG3L1P, DBNDD1, GAS8, GAS8-AS1 | arr[GRCh38] 16q12.1q24.3(50,166,030_90,037,827)x3~4 |
| 1  Brain | Loss | 17 | p13.3 | p11.2 | 18,777 | DOC2B, LINC02091, RPH3AL, RPH3AL-AS1, LOC105371430, LIAT1, RFLNB, VPS53, TLCD3A, GEMIN4, DBIL5P, GLOD4, MRM3, NXN, LOC101927727, TIMM22, ABR, MIR3183, BHLHA9, TRARG1, YWHAE, CRK, MYO1C, INPP5K, PITPNA-AS1, PITPNA, SLC43A2, SCARF1, RILP, PRPF8, TLCD2, MIR22HG, MIR22, WDR81, SERPINF2, SERPINF1, SMYD4, RPA1, RTN4RL1, LOC105371485, DPH1, OVCA2, MIR132, MIR212, HIC1, SMG6, LOC101927839, SRR, TSR1, SNORD91B, SNORD91A, SGSM2, MNT, LOC284009, METTL16, PAFAH1B1, CLUH, MIR6776, LOC105371592, CCDC92B, MIR1253, RAP1GAP2, LOC101927911, OR1D5, OR1D2, OR1G1, LOC100288728, OR1A2, OR1A1, OR1D4, OR3A2, OR3A1, OR3A4P, OR1R1P, OR1E1, OR3A3, OR1E2, SPATA22, ASPA, TRPV3, TRPV1, SHPK, CTNS, P2RX5-TAX1BP3, TAX1BP3, EMC6, P2RX5, ITGAE, HASPIN, NCBP3, CAMKK1, P2RX1, ATP2A3, LINC01975, ZZEF1, CYB5D2, ANKFY1, UBE2G1, SPNS3, SPNS2, MYBBP1A, GGT6, SMTNL2, LINC01996, ALOX15, PELP1, PELP1-DT, ARRB2, MED11, CXCL16, ZMYND15, TM4SF5, VMO1, GLTPD2, PSMB6, C17orf114, PLD2, MINK1, CHRNE, C17orf107, GP1BA, SLC25A11, RNF167, PFN1, ENO3, SPAG7, CAMTA2, MIR6864, MIR6865, INCA1, KIF1C, KIF1C-AS1, SLC52A1, ZFP3, ZNF232, ZNF232-AS1, USP6, ZNF594, ZNF594-DT, SCIMP, RABEP1, NUP88, RPAIN, C1QBP, DHX33, DHX33-DT, DERL2, MIS12, LOC728392, NLRP1, LOC339166, WSCD1, AIPL1, PIMREG, PITPNM3, KIAA0753, LOC122526780, TXNDC17, MED31, C17orf100, MIR4520-1, MIR4520-2, ALOX15P1, SLC13A5, XAF1, FBXO39, TEKT1, ALOX12P2, ALOX12-AS1, ALOX12, RNASEK-C17orf49, RNASEK, C17orf49, MIR497HG, MIR195, MIR497, BCL6B, SLC16A13, SLC16A11, CLEC10A, ASGR2, ASGR1, DLG4, ACADVL, MIR324, DVL2, PHF23, GABARAP, CTDNEP1, ELP5, CLDN7, SLC2A4, YBX2, EIF5A, GPS2, NEURL4, ACAP1, KCTD11, TMEM95, TNK1, PLSCR3, TMEM256-PLSCR3, TMEM256, NLGN2, SPEM1, SPEM2, SPEM3, TMEM102, FGF11, CHRNB1, ZBTB4, SLC35G6, POLR2A, TNFSF12, TNFSF12-TNFSF13, TNFSF13, SENP3, SENP3-EIF4A1, EIF4A1, SNORA48, SNORD10, SNORA67, CD68, MPDU1-AS1, MPDU1, SOX15, FXR2, SHBG, SAT2, ATP1B2, TP53, WRAP53, EFNB3, DNAH2, RPL29P2, KDM6B, TMEM88, NAA38, CYB5D1, CHD3, SCARNA21, RNF227, KCNAB3, TRAPPC1, CNTROB, GUCY2D, ALOX15B, ALOX12B, MIR4314, ALOXE3, HES7, PER1, MIR6883, VAMP2, TMEM107, SNORD118, MIR4521, BORCS6, AURKB, LINC00324, CTC1, PFAS, SLC25A35, RANGRF, ARHGEF15, ODF4, LOC100128288, KRBA2, RPL26, RNF222, NDEL1, MYH10, CCDC42, SPDYE4, MFSD6L, PIK3R6, PIK3R5, PIK3R5-DT, NTN1, LOC101928266, STX8, CFAP52, USP43, DHRS7C, GSG1L2, GLP2R, RCVRN, GAS7, MYH13, MYHAS, MYH8, MYH4, MYH1, MYH2, MYH3, SCO1, ADPRM, TMEM220, MAGOH2P, TMEM220-AS1, TMEM238L, PIRT, SHISA6, DNAH9, ZNF18, MAP2K4, MIR744, LINC00670, MYOCD, MYOCD-AS1, ARHGAP44-AS1, ARHGAP44, MIR1269B, ELAC2, LINC02093, HS3ST3A1, MIR548H3, CDRT15P1, COX10-DT, COX10, CDRT15, HS3ST3B1, MGC12916, CDRT7, LOC101928475, CDRT8, PMP22, MIR4731, TEKT3, CDRT4, TVP23C-CDRT4, CDRT3, TVP23C, FBXW10B, TRIM16, ZNF286A, ZNF286A-TBC1D26, TBC1D26, CDRT15P2, MEIS3P1, LINC02087, ADORA2B, ZSWIM7, TTC19, NCOR1, SNORD163, PIGL, MIR1288, CENPV, UBB, TRPV2, SNHG29, SNORD49B, SNORD49A, SNORD65, LRRC75A, ZNF287, ZNF624, CCDC144A, USP32P1, FAM106C, KRT16P2, KRT17P1, TBC1D27P, TNFRSF13B, LOC284191, LINC02090, MPRIP, PLD6, FLCN, COPS3, NT5M, MED9, RASD1, PEMT, SMCR2, RAI1, RAI1-AS1, SMCR5, SREBF1, MIR6777, MIR33B, TOM1L2, DRC3, ATPAF2, GID4, DRG2, LOC105371566, MYO15A, ALKBH5, LLGL1, FLII, MIEF2, TOP3A, SMCR8, SHMT1, MIR6778, EVPLL, LINC02076, KRT17P5, KRT17P2, KRT16P1, LGALS9C, USP32P2, FAM106A, CCDC144BP, TBC1D28, ZNF286B, FOXO3B, TRIM16L, FBXW10, TVP23B, LOC101929141, PRPSAP2, SLC5A10 | arr[GRCh38] 17p13.3p11.2(179,827_18,957,284)x1 |
| 1  Brain | Gain | 17 | q11.2 | q12 | 11,223 | NLK, PYY2, PPY2P, KRT18P55, TMEM97, IFT20, TNFAIP1, POLDIP2, TMEM199, MIR4723, SEBOX, VTN, SARM1, SLC46A1, SLC13A2, FOXN1, UNC119, PIGS, ALDOC, SPAG5, SPAG5-AS1, RSKR, BLTP2, SDF2, SUPT6H, PROCA1, RAB34, RPL23A, SNORD42B, SNORD4A, SNORD42A, SNORD4B, TLCD1, NEK8, TRAF4, FAM222B, ERAL1, MIR451A, MIR451B, MIR144, MIR4732, FLOT2, DHRS13, PHF12, LOC101927018, SEZ6, PIPOX, MYO18A, CRYBA1, NUFIP2, TAOK1, MIR4523, ABHD15, TP53I13, GIT1, ANKRD13B, CORO6, SSH2, EFCAB5, NSRP1, MIR423, MIR3184, SLC6A4, BLMH, TMIGD1, CPD, GOSR1, TBC1D29P, SMURF2P1-LRRC37BP1, SH3GL1P2, SUZ12P1, CRLF3, ATAD5, TEFM, ADAP2, RNF135, DPRXP4, LOC107984974, LOC646030, MIR4733, NF1, OMG, EVI2B, EVI2A, RAB11FIP4, MIR4724, MIR193A, MIR4725, MIR365B, COPRS, UTP6, SUZ12, LRRC37B, SH3GL1P1, LOC105371730, RHOT1, ARGFXP2, RHBDL3, C17orf75, MIR632, ZNF207, PSMD11, CDK5R1, MYO1D, MYO1D-DT, H2BN1, TMEM98, SPACA3, ASIC2, AA06, LINC01989, CCL2, CCL7, CCL11, CCL8, CCL13, CCL1, TMEM132E-DT, TMEM132E, CCT6B, ZNF830, LIG3, RFFL, RAD51L3-RFFL, RAD51D, FNDC8, NLE1, UNC45B, SLC35G3, SLFN5, SLFN11, LOC105371933, SLFN12, SLFN13, SLFN12L, SLFN14, LOC107985033, SNHG30, SNORD7, PEX12, AP2B1, RASL10B, GAS2L2, MMP28, C17orf50, TAF15, HEATR9, CCL5, LRRC37A8P, RDM1, LYZL6, CCL16, CCL14, CCL15-CCL14, CCL15, CCL23, CCL18, CCL3, CCL4, TBC1D3B, CCL3L3, CCL4L2, TBC1D3I, TBC1D3G, TBC1D3H, TBC1D3F, ZNHIT3, MYO19, PIGW, GGNBP2, DHRS11, MRM1, LHX1-DT, LHX1, AATF, MIR2909, ACACA, SNORA90, C17orf78, TADA2A, DUSP14, SYNRG, DDX52, MIR378J, HNF1B, YWHAEP7, TBC1D3K, TBC1D3L, TBC1D3D, TBC1D3C, LOC101929950, TBC1D3E, TBC1D3, NPEPPSP1, MRPL45, GPR179, SOCS7, ARHGAP23, SRCIN1, EPOP, LOC105371763, MIR4734, MLLT6, MIR4726, CISD3, PCGF2, LOC100287808, PSMB3, PIP4K2B, CWC25, MIR4727, C17orf98, RPL23, SNORA21B, SNORA21, LASP1, MIR6779, LASP1NB, FBXO47, LINC02079, LRRC37A11P, RDM1P5, PLXDC1, ARL5C, CACNB1, RPL19, STAC2, LOC101929578 | arr[GRCh38] 17q11.2q12(28,019,772_39,243,083)x3~4 |
| 1  Brain | Gain | 17 | q21.2 | q21.31 | 4,520 | GAST, HAP1, JUP, P3H4, FKBP10, NT5C3B, KLHL10, KLHL11, ACLY, ODAD4, CNP, DNAJC7, NKIRAS2, ZNF385C, C17orf113, DHX58, KAT2A, HSPB9, RAB5C, KCNH4, HCRT, GHDC, STAT5B, STAT5A, STAT3, CAVIN1, ATP6V0A1, MIR548AT, MIR5010, NAGLU, HSD17B1-AS1, HSD17B1, COASY, MLX, PSMC3IP, RETREG3, TUBG1, TUBG2, PLEKHH3, CCR10, CNTNAP1, EZH1, MIR6780A, RAMP2-AS1, RAMP2, VPS25, WNK4, COA3, CNTD1, BECN1, MIR6781, PSME3, AOC2, AOC3, AOC4P, LINC00671, G6PC1, AARSD1, PTGES3L-AARSD1, PTGES3L, RUNDC1, RPL27, IFI35, VAT1, RND2, BRCA1, NBR2, LOC101929767, NBR1, TMEM106A, CCDC200, RNU2-1, LINC00910, ARL4D, MIR2117HG, MIR2117, DHX8, ETV4, MEOX1, LINC02594, SOST, DUSP3, CFAP97D1, MPP3, CD300LG, MPP2, FAM215A, LINC01976, PPY, PYY, NAGS, TMEM101, LSM12, G6PC3, HDAC5, LOC105371789, HROB, ASB16, ASB16-AS1, TMUB2, ATXN7L3, ATXN7L3-AS1, UBTF, MIR6782, SLC4A1, RUNDC3A-AS1, RUNDC3A, SLC25A39, GRN, FAM171A2, ITGA2B, GPATCH8, FZD2, LINC01180, MEIOC, CCDC43, DBF4B, ADAM11, GJC1, HIGD1B, EFTUD2, CCDC103, GFAP, FAM187A, KIF18B, MIR6783, C1QL1, DCAKD, NMT1, PLCD3, MIR6784, ACBD4, HEXIM1, HEXIM2, LOC105371795, FMNL1-DT, FMNL1, MAP3K14-AS1, SPATA32, MAP3K14, ARHGAP27, PLEKHM1, MIR4315-1, LRRC37A4P, MAPK8IP1P2, LINC02210, LINC02210-CRHR1, CRHR1, MAPT-AS1, SPPL2C, MAPT, MAPT-IT1, STH, KANSL1, KANSL1-AS1 | arr[GRCh38] 17q21.2q21.31(41,695,163_46,215,376)x2~3 |
| 1  Brain | Gain | 17 | q21.33 | q23.2 | 11,505 | SPAG9, NME1, NME1-NME2, NME2, MBTD1, UTP18, LINC02071, LINC02072, LINC02073, CA10, LINC01982, LINC02089, LINC02876, KIF2B, TOM1L1, COX11, STXBP4, HLF, MMD, SMIM36, TMEM100, PCTP, ANKFN1, NOG, C17orf67, DGKE, MTVR2, TRIM25, MIR3614, COIL, SCPEP1, RNF126P1, AKAP1, MSI2, LOC101927557, LOC101927539, CCDC182, MRPS23, CUEDC1, VEZF1, SRSF1, DYNLL2-DT, DYNLL2, OR4D1, MSX2P1, OR4D2, EPX, MKS1, LPO, MPO, TSPOAP1, TSPOAP1-AS1, MIR142, MIR4736, SUPT4H1, RNF43, HSF5, MTMR4, SEPTIN4-AS1, SEPTIN4, TEX14, IGBP1C, RAD51C, PPM1E, TRIM37, SKA2, MIR454, MIR301A, PRR11, SMG8, GDPD1, YPEL2, MIR4729, LINC01476, DHX40, CLTC, PTRH2, VMP1, MIR21, TUBD1, RPS6KB1, RNFT1, TBC1D3P1-DHX40P1, RNFT1-DT, HEATR6, MIR4737, HEATR6-DT, WFDC21P, LOC653653, CA4, USP32, SCARNA20, CHCT1, APPBP2, LINC01999, PPM1D, BCAS3, LOC101927855, TBX2-AS1, TBX2, LINC02875, TBX4, NACA2, BRIP1, INTS2, MED13, TBC1D3P2, EFCAB3, METTL2A, TLK2 | arr[GRCh38] 17q21.33q23.2(51,035,681_62,540,692)x3~4 |
| 1  Brain | Gain | 17 | q25.1 | q25.3 | 9,507 | SDK2, LOC100134391, LINC00469, LINC02092, LINC02074, RPL38, MGC16275, TTYH2, DNAI2, KIF19, BTBD17, GPR142, GPRC5C, CD300A, CD300LB, CD300C, CD300H, CD300LD, CD300LD-AS1, LOC101928343, CD300E, RAB37, CD300LF, MIR3615, NHERF1, NAT9, TMEM104, GRIN2C, FDXR, FADS6, USH1G, OTOP2, OTOP3, HID1, HID1-AS1, CDR2L, MRPL58, KCTD2, ATP5PD, SLC16A5, ARMC7, NT5C, JPT1, SUMO2, NUP85, GGA3, MRPS7, MIF4GD, MIF4GD-DT, SLC25A19, GRB2, MIR3678, TMEM94, MIR6785, CASKIN2, TSEN54, LLGL2, MYO15B, RECQL5, SMIM5, SMIM6, SAP30BP, ITGB4, GALK1, H3-3B, MIR4738, UNK, UNC13D, WBP2, TRIM47, TRIM65, MRPL38, FBF1, ACOX1, TEN1-CDK3, TEN1, CDK3, EVPL, SRP68, GALR2, ZACN, EXOC7, MIR6868, FOXJ1, RNF157-AS1, RNF157, UBALD2, QRICH2, PRPSAP1, SPHK1, UBE2O, AANAT, RHBDF2, CYGB, PRCD, SNHG16, SNORD1C, SNORD1B, SNORD1A, ST6GALNAC2, LOC122455342, ST6GALNAC1, LOC105274304, MXRA7, JMJD6, METTL23, SRSF2, MIR636, MFSD11, LINC02080, LINC00868, MGAT5B, LOC105371899, SNHG20, SEC14L1, SCARNA16, MIR6516, SEPTIN9-DT, SEPTIN9, MIR4316, LOC400622, LOC100507351, LINC01987, LINC01973, TNRC6C, TMC6, TMC8, C17orf99, SYNGR2, TK1, AFMID, BIRC5, TMEM235, LOC105371910, LINC01993, SOCS3, SOCS3-DT, PGS1, DNAH17, DNAH17-AS1, SCAT1, CYTH1, USP36, TIMP2, CEP295NL, LGALS3BP, CANT1, C1QTNF1-AS1, C1QTNF1, ENGASE, RBFOX3, MIR4739, LINC02078, ENPP7, CBX2, CBX8, LINC01977, CBX4, LINC01979, LINC01978, TBC1D16, CCDC40, MIR1268B, GAA, EIF4A3, CARD14, SGSH, SLC26A11, RNF213, RNF213-AS1, ENDOV, MIR4730, NPTX1, RPTOR, LOC101928855, LOC400627, CHMP6, BAIAP2-DT, BAIAP2, AATK, MIR657, MIR3065, MIR338, MIR1250, PVALEF, CEP131, TEPSIN, LOC105371925, NDUFAF8, SLC38A10, LINC00482, TMEM105, LINC03048, BAHCC1, MIR4740, MIR3186, LINC01971, ACTG1, FSCN2, FAAP100, NPLOC4, TSPAN10, PDE6G, OXLD1, CCDC137, ARL16, HGS, MIR6786, MRPL12, SLC25A10, GCGR, MCRIP1, PPP1R27, P4HB, ARHGDIA, ALYREF, ANAPC11, PCYT2, NPB, SIRT7, MAFG, MILIP, PYCR1, MYADML2, NOTUM, ASPSCR1, CENPX, LRRC45, RAC3, DCXR, DCXR-DT, RFNG, GPS1, DUS1L, FASN, SNORD134, CCDC57, SLC16A3, MIR6787, CSNK1D, LINC01970, CD7, SECTM1, TEX19, UTS2R, OGFOD3, HEXD, HEXD-IT1, CYBC1, NARF-AS2, NARF, FOXK2, WDR45B, RAB40B, MIR4525, LOC101929552, FN3KRP, FN3K, TBCD, ZNF750, B3GNTL1, METRNL | arr[GRCh38] 17q25.1q25.3(73,576,881_83,083,947)x3~4 |
| 1  Brain | Loss | 19 | p13.3 | p11 | 24,063 | PLPP2, MIER2, THEG, C2CD4C, SHC2, ODF3L2, MADCAM1, TPGS1, CDC34, GZMM, BSG-AS1, BSG, HCN2, POLRMT, FGF22, RNF126, FSTL3, PRSS57, PALM, MISP, PTBP1, MIR4745, PLPPR3, MIR3187, AZU1, PRTN3, ELANE, CFD, MED16, RNU6-9, R3HDM4, KISS1R, ARID3A, WDR18, GRIN3B, TMEM259, RNU6-2, CNN2, ABCA7, ARHGAP45, POLR2E, GPX4, SBNO2, STK11, CBARP, ATP5F1D, MIDN, CIRBP-AS1, CIRBP, FAM174C, EFNA2, PWWP3A, NDUFS7, GAMT, DAZAP1, RPS15, APC2, C19orf25, PCSK4, REEP6, ADAMTSL5, PLK5, MEX3D, MBD3, UQCR11, TCF3, ONECUT3, ATP8B3, REXO1, MIR1909, LOC100288123, KLF16, ABHD17A, ADAT3, SCAMP4, CSNK1G2, CSNK1G2-AS1, BTBD2, MKNK2, MOB3A, IZUMO4, AP3D1, DOT1L, PLEKHJ1, MIR1227, MIR6789, SF3A2, AMH, MIR4321, JSRP1, OAZ1, PEAK3, LINGO3, LSM7, SPPL2B, TMPRSS9, TIMM13, LMNB2, MIR7108, LINC01775, GADD45B, GNG7, MIR7850, DIRAS1, SLC39A3, SGTA, THOP1, ZNF554, ZNF555, ZNF556, ZNF57, ZNF77, TLE6, TLE2, TLE5, GNA11, GNA15, GNA15-DT, S1PR4, NCLN, CELF5, NFIC, SMIM24, SMIM44, DOHH, FZR1, TEKTIP1, MFSD12, HMG20B, GIPC3, TBXA2R, CACTIN-AS1, CACTIN, PIP5K1C, TJP3, APBA3, MRPL54, RAX2, MATK, ZFR2, ATCAY, NMRK2, DAPK3, MIR637, EEF2, SNORD37, PIAS4, ZBTB7A, MAP2K2, CREB3L3, SIRT6, ANKRD24, EBI3, YJU2, SHD, TMIGD2, FSD1, STAP2, MPND, SH3GL1, CHAF1A, UBXN6, MIR4746, HDGFL2, PLIN4, PLIN5, LRG1, SEMA6B, TNFAIP8L1, MYDGF, DPP9, DPP9-AS1, MIR7-3HG, MIR7-3, FEM1A, TICAM1, PLIN3, ARRDC5, UHRF1, MIR4747, KDM4B, PTPRS, ZNRF4, TINCR, SAFB2, SAFB, MICOS13, HSD11B1L, RPL36, LONP1, CATSPERD, PRR22, DUS3L, NRTN, FUT6, FUT3, LOC101928844, FUT5, NDUFA11, VMAC, CAPS, RANBP3, RANBP3-DT, RFX2, ACSBG2, MLLT1, ACER1, CLPP, ALKBH7, PSPN, GTF2F1, MIR6885, MIR6790, LOC390877, KHSRP, MIR3940, SLC25A41, SLC25A23, CRB3, DENND1C, TUBB4A, TNFSF9, CD70, TNFSF14, C3, GPR108, MIR6791, TRIP10, SH2D3A, VAV1, ADGRE1, ADGRE4P, FLJ25758, MBD3L2B, MBD3L5, MBD3L4, MBD3L2, MBD3L3, ZNF557, INSR, ARHGEF18, PEX11G, TEX45, ZNF358, MCOLN1, PNPLA6, CAMSAP3, MIR6792, XAB2, PET100, STXBP2, PCP2, RETN, MCEMP1, TRAPPC5, FCER2, CLEC4G, CD209, CLEC4M, CLEC4GP1, EVI5L, PRR36, LYPLA2P2, LRRC8E, MAP2K7, TGFBR3L, SNAPC2, CTXN1, TIMM44, ELAVL1, CCL25, FBN3, CERS4, CD320, NDUFA7, RPS28, KANK3, ANGPTL4, RAB11B-AS1, MIR4999, RAB11B, MARCHF2, HNRNPM, PRAM1, ZNF414, MYO1F, ADAMTS10, NFILZ, ACTL9, OR2Z1, ZNF558, MBD3L1, MUC16, OR1M1, OR7G2, OR7G1, OR7G3, ZNF317, OR7D2, OR7D4, OR7E24, ZNF699, ZNF559, ZNF559-ZNF177, ZNF177, ZNF266, ZNF560, ZNF426, ZNF426-DT, ZNF121, ZNF561, ZNF561-AS1, ZNF562, ZNF812P, ZNF846, LOC100505555, FBXL12, UBL5, PIN1-DT, PIN1, OLFM2, COL5A3, RDH8, MIR5589, C3P1, SHFL, ANGPTL6, PPAN, PPAN-P2RY11, SNORD105, SNORD105B, P2RY11, EIF3G, DNMT1, S1PR2, MIR4322, MRPL4, ICAM1, ICAM4, ICAM5, ZGLP1, FDX2-ZGLP1, FDX2, RAVER1, ICAM3, TYK2, CDC37, MIR1181, PDE4A, KEAP1, S1PR5, ATG4D, MIR1238, KRI1, CDKN2D, AP1M2, SLC44A2, ILF3-DT, ILF3, QTRT1, DNM2, MIR638, MIR4748, MIR199A1, MIR6793, TMED1, HIKESHIP2, C19orf38, CARM1, YIPF2, TIMM29, SMARCA4, LDLR-AS1, LDLR, MIR6886, SPC24, KANK2, DOCK6, LOC105372273, ANGPTL8, TSPAN16, RAB3D, TMEM205, CCDC159, PLPPR2, SWSAP1, EPOR, RGL3, ODAD3, PRKCSH, ELAVL3, ZNF653, MIR7974, ECSIT, CNN1, ELOF1, ACP5, ZNF627, HNRNPA1P10, ZNF833P, ZNF823, ZNF441, ZNF491, ZNF440, ZNF439, ZNF69, ZNF700, ZNF763, ZNF433-AS1, ZNF433, ZNF878, ZNF844, ZNF788P, ZNF20, ZNF625-ZNF20, ZNF625, ZNF136, LOC100289333, ZNF44, ZNF563, ZNF442, ZNF799, ZNF443, ZNF709, ZNF564, ZNF490, ZNF791, MAN2B1, WDR83, WDR83OS, DHPS, GNG14, FBXW9, TNPO2, SNORD135, SNORD41, TRIR, GET3, BEST2, HOOK2, MIR5684, JUNB, PRDX2, THSD8, RNASEH2A, RTBDN, MAST1, MIR6794, DNASE2, KLF1, GCDH, SYCE2, MIR5695, FARSA, FARSA-AS1, CALR, MIR6515, RAD23A, GADD45GIP1, DAND5, NFIX, LYL1, TRMT1, NACC1, STX10, IER2, CACNA1A, YJU2B, MRI1, C19orf53, ZSWIM4, MIR23AHG, MIR24-2, MIR27A, MIR23A, NANOS3, MIR181C, MIR181D, BRME1, CC2D1A, PODNL1, DCAF15, RFX1, RLN3, IL27RA, PALM3, MISP3, MIR1199, C19orf67, SAMD1, PRKACA, SMIM46, ASF1B, ADGRL1-AS1, ADGRL1, LINC01841, LINC01842, ADGRE5, DDX39A, PKN1, PTGER1, GIPC1, DNAJB1, MIR639, TECR, NDUFB7, CLEC17A, ADGRE3, SNORA104, ZNF333, ADGRE2, OR7C1, OR7A5, OR7A10, OR7A17, OR7C2, SLC1A6, CCDC105, CASP14, OR1I1, SYDE1, ILVBL, NOTCH3, MIR6795, EPHX3, BRD4, AKAP8, AKAP8L, WIZ, MIR1470, RASAL3, PGLYRP2, CYP4F22, CYP4F8, CYP4F3, CYP4F12, OR10H2, OR10H3, CYP4F24P, OR10H5, OR10H1, UCA1, CLEC4OP, CYP4F2, CYP4F11, OR10H4, LINC00661, LINC00905, LINC01855, TPM4, RAB8A, HSH2D, CIB3, FAM32A, AP1M1, KLF2, EPS15L1, CALR3, C19orf44, CHERP, SLC35E1, MED26, SMIM7, TMEM38A, NWD1, SIN3B, F2RL3, CPAMD8, HAUS8, MYO9B, SNORA118, USE1, OCEL1, NR2F6, USHBP1, BABAM1, ANKLE1, ABHD8, MRPL34, DDA1, ANO8, GTPBP3, PLVAP, CCDC194, BST2, BISPR, MVB12A, TMEM221, NXNL1, SLC27A1, PGLS-DT, PGLS, NIBAN3, COLGALT1, UNC13A, MAP1S, FCHO1, B3GNT3, INSL3, JAK3, RPL18A, SNORA68, SLC5A5, CCDC124, KCNN1, ARRDC2, IL12RB1, MAST3, PIK3R2, IFI30, MPV17L2, RAB3A, LOC102725254, PDE4C, LOC729966, IQCN, JUND, MIR3188, LSM4, PGPEP1, GDF15, MIR3189, LRRC25, SSBP4, ISYNA1, ELL, FKBP8, KXD1, UBA52, REX1BD, CRLF1, TMEM59L, KLHL26, CRTC1, COMP, UPF1, CERS1, GDF1, COPE, DDX49, HOMER3, HOMER3-AS1, SUGP2, ARMC6, SLC25A42, TMEM161A, MEF2B, BORCS8-MEF2B, BORCS8, RFXANK, NR2C2AP, NCAN, HAPLN4, TM6SF2, SUGP1, MAU2, GATAD2A, MIR640, TSSK6, NDUFA13, YJEFN3, CILP2, PBX4, LPAR2, GMIP, ATP13A1, ZNF101, ZNF14, LINC00663, ZNF56P, ZNF506, ZNF253, ZNF93, ZNF682, ZNF90, ZNF486, ZNF826P, MIR1270, ZNF737, ZNF626, LOC105372319, ZNF66, ZNF85, ZNF430, ZNF714, ZNF431, ZNF708, ZNF738, ZNF493, LINC00664, ZNF429, LOC400682, ZNF100, CCNYL6, ZNF43, ZNF208, ZNF257, ZNF676, ZNF729, ZNF98, LOC105376917, LOC101929124, LINC01233, GOLGA2P9, LOC100996349, LINC01785, ZNF492, ZNF99, ZNF723, ZNF728, LINC01859, LINC01858, ZNF730, ZNF724, IPO5P1, ZNF91, LINC01224, ZNF675, ZNF681, RPSA2, ZNF726, LOC100505851, ZNF254, HAVCR1P1 | arr[GRCh38] 19p13.3p11(260,912_24,324,339)x1 |
| 1  Brain | cn-LOH | 19 | q11 | q13.43 | 30,786 | LINC00662, LINC02987, LOC100420587, LOC102724908, LINC00906, LOC102724958, LINC01532, UQCRFS1, UQCRFS1-DT, VSTM2B-DT, VSTM2B, POP4, PLEKHF1, C19orf12, CCNE1, URI1, ZNF536, LINC01834, TSHZ3, LINC01791, LINC02841, THEG5, LINC01533, LINC01782, ZNF507, DPY19L3-DT, DPY19L3, PDCD5, ANKRD27, SNORA68B, RGS9BP, NUDT19, TDRD12, SLC7A9, CEP89, FAAP24, RHPN2, GPATCH1, WDR88, LRP3, SLC7A10, CEBPA, CEBPA-DT, CEBPG, PEPD, CHST8, KCTD15, LSM14A, GARRE1, GPI, PDCD2L, UBA2, WTIP, ZNF807P, SCGB1B2P, SCGB2B2, SCGB2B3P, ZNF302, ZNF181, ZNF599, LINC01801, LINC03049, LINC00904, LINC01838, ZNF30-AS1, ZNF30, ZNF792, GRAMD1A, SCN1B, HPN, HPN-AS1, FXYD3, MIR6887, LGI4, FXYD1, FXYD7, FXYD5, FAM187B, LSR, USF2, HAMP, MAG, CD22, MIR5196, FFAR1, FFAR3, GPR42, LINC01531, FFAR2, KRTDAP, DMKN, SBSN, GAPDHS, TMEM147-AS1, TMEM147, ATP4A, PMIS2, LINC01766, HAUS5, RBM42, ETV2, COX6B1, UPK1A, UPK1A-AS1, ZBTB32, KMT2B, IGFLR1, U2AF1L4, PSENEN, LIN37, HSPB6, PROSER3, ARHGAP33, LINC01529, PRODH2, NPHS1, KIRREL2, APLP1, NFKBID, HCST, TYROBP, LRFN3, LOC105372383, SDHAF1, SYNE4, ALKBH6, LOC101927572, CLIP3, THAP8, WDR62, OVOL3, POLR2I, TBCB, CAPNS1, COX7A1, ZNF565, ZNF146, LOC100134317, LINC00665, ZFP14, ZFP82, LOC644189, ZNF566, ZNF566-AS1, ZNF260, ZNF529, ZNF529-AS1, ZNF382, ZNF461, ZNF567, ZNF567-DT, ZNF850, LOC728485, ZNF790-AS1, ZNF790, ZNF345, ZNF829, ZNF568, ZNF420, ZNF585A, ZNF585B, ZNF383, LINC01535, LOC284412, ZNF875, ZNF527, ZNF569, ZNF570, ZNF793-AS1, ZNF793, ZNF571-AS1, ZNF540, ZNF571, ZFP30, ZNF781, ZNF607, ZNF573, LOC644554, WDR87BP, WDR87, SIPA1L3, SNORD152, DPF1, PPP1R14A, SPINT2, YIF1B, C19orf33, KCNK6, CATSPERG, PSMD8, GGN, SPRED3, FAM98C, RASGRP4, RYR1, MAP4K1, MAP4K1-AS1, EIF3K, ACTN4, CAPN12, LGALS7, LGALS7B, LGALS4, ECH1, HNRNPL, RINL, SIRT2, NFKBIB, CCER2, SARS2, MRPS12, FBXO17, FBXO27, ACP7, PAK4, NCCRP1, SYCN, IFNL3, IFNL4, IFNL2, IFNL1, LRFN1, GMFG, SAMD4B, PAF1, MED29, ZFP36, MIR4530, PLEKHG2, RPS16, SUPT5H, SNORD175, TIMM50, DLL3, SELENOV, EID2B, EID2, LGALS13, LOC100129935, LGALS16, LGALS17A, LGALS14, CLC, LEUTX, DYRK1B, MIR6719, FBL, FCGBP, PSMC4, ZNF546, ZNF780B, ZNF780A, MAP3K10, TTC9B, CCNP, AKT2, MIR641, C19orf47, PLD3, MIR6796, HIPK4, PRX, SERTAD1, SERTAD3, BLVRB, SPTBN4, SHKBP1, LTBP4, NUMBL, COQ8B, ITPKC, ACTMAP, SNRPA, MIA, MIA-RAB4B, RAB4B-EGLN2, RAB4B, EGLN2, CYP2T1P, CYP2A6, CYP2A7, CYP2G1P, CYP2B7P, CYP2B6, CYP2A13, CYP2F1, CYP2S1, AXL, HNRNPUL1, CCDC97, TGFB1, B9D2, TMEM91, EXOSC5, BCKDHA, B3GNT8, DMAC2, ERICH4, PCAT19, LINC01480, CEACAM21, CEACAM4, CEACAM7, CEACAM5, CEACAM6, CEACAM3, LYPD4, DMRTC2, RPS19, MIR6797, CD79A, ARHGEF1, ERFL, RABAC1, ATP1A3, GRIK5, ZNF574, POU2F2, LNROP, MIR4323, DEDD2, ZNF526, GSK3A, ERF, CIC, PAFAH1B3, PRR19, TMEM145, MEGF8, MIR8077, CNFN, LOC101930071, LIPE-AS1, LIPE, CXCL17, CEACAM1, CEACAM8, PSG3, PSG8, PSG8-AS1, PSG10P, PSG1, PSG6, PSG7, PSG11, PSG2, PSG5, PSG4, LOC284344, PSG9, PRG1, CD177, TEX101, LYPD3, PHLDB3, ETHE1, ZNF575, XRCC1, PINLYP, IRGQ, ZNF576, ZNF428, SRRM5, CADM4, PLAUR, LOC105372412, IRGC, SMG9, KCNN4, LYPD5, ZNF283, ZNF404, LOC100505715, ZNF45-AS1, ZNF45, ZNF221, ZNF155, ZNF230-DT, ZNF230, ZNF222-DT, ZNF222, ZNF223, ZNF284, ZNF224, ZNF225-AS1, ZNF225, ZNF234, ZNF226, ZNF227, ZNF233, ZNF235, ZNF112, ZNF285, ZNF229, ZNF180, CEACAM20, CEACAM22P, IGSF23, PVR, MIR4531, CEACAM19, CEACAM16, BCL3, MIR8085, CBLC, BCAM, NECTIN2, TOMM40, APOE, APOC1, APOC1P1, APOC4, APOC4-APOC2, APOC2, CLPTM1, RELB, CLASRP, ZNF296, GEMIN7, GEMIN7-AS1, PPP1R37, NKPD1, TRAPPC6A, BLOC1S3, EXOC3L2, MARK4, CKM, KLC3, ERCC2, PPP1R13L, POLR1G, ERCC1, MIR6088, FOSB, RTN2, PPM1N, VASP, OPA3, GPR4, EML2, MIR330, EML2-AS1, GIPR, MIR642A, MIR642B, SNRPD2, QPCTL, FBXO46, MEIOSIN, SIX5, DM1-AS, DMPK, DMWD, RSPH6A, SYMPK, FOXA3, IRF2BP1, MYPOP, NANOS2, NOVA2, CCDC61, MIR769, PGLYRP1, IGFL4, IGFL3, IGFL2, IGFL2-AS1, LOC93429, IGFL1, HIF3A, PPP5C, CCDC8, PNMA8C, PNMA8A, PNMA8B, PPP5D1P, CALM3, PTGIR, GNG8, DACT3, DACT3-AS1, PRKD2, MIR320E, STRN4, FKRP, SLC1A5, SNAR-E, AP2S1, ARHGAP35, NPAS1, TMEM160, ZC3H4, SAE1, BBC3, MIR3190, MIR3191, CCDC9, INAFM1, C5AR1, C5AR2, DHX34, MEIS3, SLC8A2, KPTN, NAPA-AS1, NAPA, ZNF541, BICRA, EHD2, NOP53, SNORD23, NOP53-AS1, SELENOW, TPRX1, CRX, TPRX2, SULT2A1, SNAR-A12, SNAR-C5, SNAR-A1, SNAR-A3, SNAR-C2, SNAR-A2, SNAR-C4, SNAR-A13, SNAR-C3, SNAR-C1, BSPH1, ELSPBP1, CABP5, PLA2G4C, PLA2G4C-AS1, LIG1, ZSWIM9, CARD8, CARD8-AS1, ZNF114, ODAD1, EMP3, TMEM143, SYNGR4, KDELR1, GRIN2D, GRWD1, KCNJ14, CYTH2, LMTK3, SULT2B1, FAM83E, SPACA4, RPL18, SPHK2, DBP, CA11, SEC1P, NTN5, FUT2, LOC105447645, MAMSTR, RASIP1, IZUMO1, FUT1, FGF21, BCAT2, HSD17B14, PLEKHA4, PPP1R15A, TULP2, NUCB1, NUCB1-AS1, DHDH, BAX, FTL, GYS1, RUVBL2, MIR6798, LHB, LOC101059948, CGB3, SNAR-G2, CGB2, CGB1, SNAR-G1, CGB5, CGB8, CGB7, NTF4, KCNA7, SNRNP70, LIN7B, C19orf73, PPFIA3, HRC, TRPM4, SLC6A16, MIR4324, CD37, TEAD2, DKKL1, LOC101928295, KASH5, PTH2, GFY, SLC17A7, PIH1D1, ALDH16A1, FLT3LG, RPL13A, SNORD32A, SNORD33, SNORD34, SNORD35A, RPS11, SNORD35B, MIR150, FCGRT, RCN3, NOSIP, PRRG2, PRR12, RRAS, SCAF1, IRF3, BCL2L12, PRMT1, MIR5088, ADM5, CPT1C, TSKS, AP2A1, MIR6799, FUZ, MED25, MIR6800, PTOV1-AS1, PTOV1, MIR4749, PTOV1-AS2, PNKP, AKT1S1, TBC1D17, MIR4750, IL4I1, NUP62, ATF5, MIR4751, SIGLEC11, SIGLEC16, VRK3, ZNF473, ZNF473CR, SNAR-A4, SNAR-A14, SNAR-A5, SNAR-A6, SNAR-A7, SNAR-A8, SNAR-A9, SNAR-A10, SNAR-A11, SNAR-B1, SNAR-B2, SNAR-D, IZUMO2, MYH14, KCNC3, NAPSB, NAPSA, NR1H2, POLD1, SPIB, MYBPC2, GARIN5A, EMC10, JOSD2, ASPDH, LRRC4B, SNAR-F, SYT3, C19orf81, SHANK1, CLEC11A, GPR32, SMIM47, ACP4, C19orf48P, SNORD88B, SNORD88A, SNORD88C, LINC01869, KLK1, KLK15, LOC105372441, KLK3, KLK2, KLKP1, KLK4, KLK5, KLK6, KLK7, KLK8, KLK9, KLK10, KLK11, KLK12, KLK13, KLK14, CTU1, SIGLEC9, SIGLEC7, LOC101928517, SIGLEC17P, MIR8074, CD33, SIGLECL1, LINC01872, IGLON5, VSIG10L, ETFB, CLDND2, NKG7, LIM2, C19orf84, SIGLEC10, SIGLEC10-AS1, SIGLEC8, CEACAM18, SIGLEC12, SIGLEC6, ZNF175, LINC01530, SIGLEC5, SIGLEC14, SPACA6-AS1, SPACA6, MIR99B, MIRLET7E, MIR125A, HAS1, FPR1, FPR2, FPR3, ZNF577, ZNF649-AS1, ZNF649, ZNF613, ZNF350-AS1, ZNF350, ZNF615, ZNF614, ZNF432, ZNF841, ZNF616, ZNF836, PPP2R1A, MIR6801, ZNF766, MIR643, ZNF480, ZNF610, ZNF880, ZNF528-AS1, ZNF528, ZNF534, ZNF578, ZNF808, ZNF701, ZNF137P, ZNF83, LOC122539214, ZNF611, ZNF600, ZNF28, ZNF468, ZNF320, ZNF888, ZNF321P, ZNF816-ZNF321P, ZNF816, ZNF702P, ERVV-1, ERVV-2, ZNF160, ZNF415, ZNF347, ZNF665, ZNF818P, ZNF677, VN1R2, VN1R4, FAM90A27P, BIRC8, ZNF845, ZNF525, ZNF765, ZNF765-ZNF761, TPM3P9, ZNF761, ZNF813, ZNF331, LOC284379, DPRX, MIR512-1, MIR512-2, MIR1323, MIR498, MIR520E, MIR515-1, MIR519E, MIR520F, MIR515-2, MIR519C, MIR1283-1, MIR520A, MIR526B, MIR519B, MIR525, MIR523, MIR518F, MIR520B, MIR518B, MIR526A1, MIR520C, MIR518C, MIR524, MIR517A, MIR519D, MIR521-2, MIR520D, MIR517B, MIR520G, MIR516B2, MIR526A2, MIR518E, MIR518A1, MIR518D, MIR516B1, MIR518A2, MIR517C, MIR520H, MIR521-1, MIR522, MIR519A1, MIR527, MIR516A1, MIR1283-2, MIR516A2, MIR519A2, MIR371A, MIR371B, MIR372, MIR373, NLRP12, MYADM-AS1, MYADM, PRKCG, CACNG7, CACNG8, MIR935, CACNG6, VSTM1, TARM1, OSCAR, NDUFA3, TFPT, PRPF31, CNOT3, LENG1, TMC4, MBOAT7, TSEN34, RPS9, LILRB3, LILRA6, LILRB5, LILRB2, MIR4752, LILRA5, LILRA4, LAIR1, TTYH1, LENG8-AS1, LENG8, LENG9, CDC42EP5, LAIR2, KIR3DX1, LILRA2, LILRA1, LILRB1, MIR8061, LILRB4, LILRP2, KIR3DL3, KIR2DL3, LOC101928804, KIR2DL1, KIR2DL4, KIR3DL1, KIR2DS4, KIR3DL2, FCAR, NCR1, NLRP7, NLRP2, GP6, RDH13, EPS8L1, PPP1R12C, MIR7975, TNNT1, TNNI3, DNAAF3, SYT5, PTPRH, TMEM86B, PPP6R1, MIR6804, MIR6802, MIR6803, HSPBP1, BRSK1, TMEM150B, KMT5C, COX6B2, GARIN5B, IL11, TMEM190, TMEM238, RPL28, MIR6805, UBE2S, SNORD157, SHISA7, ISOC2, C19orf85, ZNF628, NAT14, SSC5D, SBK2, SBK3, ZNF579, FIZ1, ZNF524, ZNF865, ZNF784, LOC107983998, ZNF580, ZNF581, CCDC106, U2AF2, EPN1, NLRP9, RFPL4A, RFPL4AL1, NLRP11, NLRP4, NLRP13, NLRP8, NLRP5, LINC01864, ZNF787, ZNF444, GALP, ZSCAN5B, ZSCAN5C, ZSCAN5A, EDDM13, ZNF542P, ZNF582, ZNF582-DT, ZNF583, ZNF667, ZNF667-AS1, ZNF471, ZFP28-DT, ZFP28, ZNF470, ZNF71, ZNF71-SMIM17, SMIM17, ZNF835, ZIM2-AS1, ZIM2, PEG3, PEG3-AS1, MIMT1, USP29, ZIM3, DUXA, ZNF264, AURKC, ZNF805, ZNF460-AS1, ZNF460, ZNF543, ZNF304, TRAPPC2B, ZNF547, ZNF548, ZNF17, ZNF749, VN1R1, ZNF772, ZNF419, ZNF773, ZNF549, ZNF550, ZNF416, ZIK1, ZNF530, ZNF134, ZNF211, ZSCAN4, ZNF551, ZNF154, ZNF671, ZNF776, ZNF586, ZNF552, FKBP1AP1, ZNF587B, ZNF587, UBE2CP5, ZNF814, ZNF417, ZNF418, ZNF256, C19orf18, ZNF606, ZNF606-AS1, VN2R19P, ZSCAN1, ZNF135, ZSCAN18, ZNF329, ZNF274, ZNF544, ZNF8, ZNF8-ERVK3-1, ERVK3-1, LOC105372480, ZSCAN22, MIR6806, A1BG, A1BG-AS1, ZNF497, ZNF497-AS1, ZNF837, MIR4754, RPS5, MIR10394, RNF225, LINC02560, ZNF584, ZNF132, ZNF324B, ZNF324, ZNF446, SLC27A5, ZBTB45, TRIM28, MIR6807, CHMP2A, UBE2M, MZF1-AS1, MZF1 | arr[GRCh38] 19q11q13.43(27,782,420_58,568,566)x2 hmz |
| 1  Brain | Gain | 20 | p13 | p12.1 | 13,889 | DEFB125, DEFB126, DEFB127, DEFB128, DEFB129, DEFB132, C20orf96, ZCCHC3, NRSN2-AS1, SOX12, NRSN2, TRIB3, RBCK1, TBC1D20, CSNK2A1, TCF15, SRXN1, SCRT2, SLC52A3, FAM110A, ANGPT4, RSPO4, PSMF1, LOC105372493, TMEM74B, C20orf202, RAD21L1, SNPH, SDCBP2, FKBP1A-SDCBP2, SDCBP2-AS1, FKBP1A, MIR6869, NSFL1C, SIRPB2, SIRPD, SIRPB1, SIRPG, SIRPG-AS1, SIRPB3P, LOC100289473, SIRPA, PDYN-AS1, PDYN, STK35, LINC03086, TGM3, TGM6, SNRPB, SNORD119, ZNF343, TMC2, NOP56, MIR1292, SNORD110, SNORA51, SNORD86, SNORD56, SNORD57, IDH3B, EBF4, CPXM1, C20orf141, TMEM239, PCED1A, VPS16, PTPRA, GNRH2, MRPS26, OXT, AVP, UBOX5-AS1, UBOX5, FASTKD5, LZTS3, DDRGK1, ITPA, SLC4A11, DNAAF9, ATRN, GFRA4, ADAM33, SIGLEC1, HSPA12B, ADISSP, SPEF1, CENPB, CDC25B, LINC01730, AP5S1, MAVS, PANK2, MIR103A2, MIR103B2, RNF24, SMOX, LINC01433, ADRA1D, PRNP, PRND, PRNT, RASSF2, SLC23A2, TMEM230, PCNA, PCNA-AS1, CDS2, PROKR2, LINC00658, LOC643406, LINC00654, LINC01729, GPCPD1, SHLD1, CHGB, TRMT6, MCM8, MCM8-AS1, CRLS1, LRRN4, FERMT1, CASC20, LINC01713, BMP2, LINC01428, LINC01751, LINC01706, MIR8062, HAO1, TMX4, TMX4-AS1, PLCB1, PLCB1-IT1, RNU105B, PLCB4, LAMP5-AS1, LAMP5, PAK5, PARAL1, SNAP25-AS1, ANKEF1, SNAP25, MKKS, LOC128706665, LOC128706666, SLX4IP, JAG1, MIR6870, LINC01752, LOC101929413, LINC02871, LOC339593, LINC00687, BTBD3, LINC01722, LOC102606466, LINC01723, SPTLC3, ISM1, ISM1-AS1, TASP1, ESF1, NDUFAF5, SEL1L2 | arr[GRCh38] 20p13p12.1(80,928_13,969,786)x3 |
| 1  Brain | Gain | 20 | p12.1 | p11.1 | 11,635 | MACROD2, MACROD2-AS1, LOC613266, KIF16B, SNRPB2, OTOR, PCSK2, BFSP1, DSTN, RRBP1, BANF2, SNX5, SNORD17, MGME1, OVOL2, KAT14, PET117, ZNF133, LINC00851, DZANK1, POLR3F, MIR3192, RBBP9, SEC23B, SMIM26, DTD1, DTD1-AS1, LINC00652, LCDR, SCP2D1-AS1, SCP2D1, SLC24A3, SLC24A3-AS1, RIN2, NAA20, CRNKL1, CFAP61, CFAP61-AS1, INSM1, RALGAPA2, LINC00237, KIZ, KIZ-AS1, XRN2, NKX2-4, LOC112268271, NKX2-2, LINC01727, LINC01726, PAX1, LINC01432, LINC01427, LOC284788, LINC00261, FOXA2, LNCNEF, LINC01747, SSTR4, THBD, CD93, LINC00656, NXT1, LINC01431, GZF1, NAPB, CSTL1, CST11, CST8, CST13P, CST9L, CST9, CST3, CST4, CST1, CST2, CST5, GGTLC1, LINC01721, SYNDIG1, CST7, APMAP, ACSS1, VSX1, LOC284798, LOC101926889, ENTPD6, PYGB, ABHD12, GINS1, NINL, NANP, ZNF337-AS1, ZNF337, LOC105372582, FAM182B, LOC101926935, LINC01733, LOC100134868, FAM182A, NCOR1P1, MIR663AHG, MIR663A | arr[GRCh38] 20p12.1p11.1(14,690,429_26,324,930)x4 |
| 1  Brain | Gain | 20 | q11.21 | q13.33 | 33,037 | DEFB115, DEFB116, DEFB118, DEFB119, DEFB121, DEFB122, DEFB123, DEFB124, REM1, LINC00028, HM13, MCTS2, HM13-AS1, ID1, MIR3193, COX4I2, BCL2L1, ABALON, TPX2, MYLK2, FOXS1, DUSP15, TTLL9, PDRG1, XKR7, CCM2L, HCK, TM9SF4, TSPY26P, PLAGL2, POFUT1, MIR1825, KIF3B, ASXL1, NOL4L, LOC101929698, NOL4L-DT, C20orf203, COMMD7, DNMT3B, MAPRE1, LOC119746555, EFCAB8, SUN5, BPIFB2, BPIFB6, BPIFB3, BPIFB4, BPIFA2, BPIFA4P, BPIFA3, BPIFA1, BPIFB1, CDK5RAP1, SNTA1, CBFA2T2, NECAB3, C20orf144, ACTL10, E2F1, PXMP4, ZNF341, ZNF341-AS1, CHMP4B, RALY-AS1, RALY, MIR4755, EIF2S2, ASIP, AHCY, ITCH, MIR644A, DYNLRB1, MAP1LC3A, PIGU, TP53INP2, NCOA6, HMGB3P1, GGT7, ACSS2, GSS, MYH7B, MIR499A, MIR499B, TRPC4AP, EDEM2, MMP24-AS1-EDEM2, PROCR, MMP24, MMP24OS, EIF6, FAM83C-AS1, FAM83C, UQCC1, GDF5-AS1, GDF5, MIR1289-1, CEP250, CEP250-AS1, C20orf173, ERGIC3, FER1L4, SPAG4, CPNE1, RBM12, NFS1, ROMO1, RBM39, PHF20, SCAND1, CNBD2, NORAD, EPB41L1, EPB41L1-AS1, AAR2, DLGAP4, DLGAP4-AS1, MYL9, TGIF2, TGIF2-RAB5IF, RAB5IF, SLA2, NDRG3, DSN1, SOGA1, TLDC2, SAMHD1, RBL1, MROH8, RPN2, GHRH, MANBAL, SRC, BLCAP, NNAT, LINC01746, LINC00489, LOC100287792, CTNNBL1, VSTM2L, TTI1, RPRD1B, TGM2, KIAA1755, LOC149684, BPI, LBP, SNHG17, SNORA71B, SNORA71A, SNORA71C, SNORA71D, SNHG11, SNORA71E, SNORA60, RALGAPB, MIR548O2, ADIG, ARHGAP40, SLC32A1, ACTR5, PPP1R16B, FAM83D, DHX35, LINC01734, LINC01370, MAFB, SNORD154, LOC100128988, TOP1, PLCG1-AS1, PLCG1, MIR6871, ZHX3, LPIN3, EMILIN3, CHD6, PTPRT, LOC101927159, PTPRT-AS1, SRSF6, L3MBTL1, SGK2, IFT52, MYBL2, GTSF1L, LINC01728, TOX2, JPH2, OSER1, OSER1-DT, GDAP1L1, FITM2, R3HDML, R3HDML-AS1, HNF4A, HNF4A-AS1, MIR3646, LINC01430, LINC01620, TTPAL, SERINC3, PKIG, ADA, LINC01260, KCNK15-AS1, CCN5, KCNK15, RIMS4, YWHAB, PABPC1L, TOMM34, STK4-DT, STK4, KCNS1, WFDC5, WFDC12, PI3, SEMG1, SEMG2, SLPI, MATN4, RBPJL, SDC4, SYS1, SYS1-DBNDD2, TP53TG5, DBNDD2, PIGT, MIR6812, LOC105372631, WFDC2, SPINT3, WFDC6, EPPIN-WFDC6, EPPIN, WFDC8, WFDC9, WFDC10A, WFDC11, WFDC10B, WFDC13, MIR3617, SPINT4, WFDC3, DNTTIP1, UBE2C, TNNC2, SNX21, ACOT8, ZSWIM3, ZSWIM1, SPATA25, NEURL2, CTSA, PLTP, LOC107985388, PCIF1, ZNF335, MMP9, SLC12A5-AS1, SLC12A5, NCOA5, CD40, CDH22, SLC35C2, ELMO2, LOC105372633, ZNF663P, MKRN7P, ZNF334, OCSTAMP, SLC13A3, TP53RK, SLC2A10, EYA2, EYA2-AS1, MIR3616, ZMYND8, LOC100131496, LOC101927377, LINC01754, NCOA3, SULF2, LINC01522, LINC01523, LINC00494, PREX1, ARFGEF2, CSE1L-DT, CSE1L, STAU1, DDX27, ZNFX1, ZFAS1, SNORD12C, SNORD12B, SNORD12, KCNB1, PTGIS, B4GALT5, SLC9A8, MIR12122, SPATA2, LOC105372653, RNF114, SNAI1, TRERNA1, UBE2V1, PEDS1-UBE2V1, PEDS1, LINC01275, LINC01273, CEBPB-AS1, CEBPB, PELATON, LINC01270, LINC01271, PTPN1, MIR645, RIPOR3, MIR1302-5, RIPOR3-AS1, PARD6B, BCAS4, ADNP, ADNP-AS1, DPM1, MOCS3, KCNG1, NFATC2, MIR3194, ATP9A, SALL4, LINC01429, ZFP64, LINC01524, TSHZ2, LOC101927770, ZNF217, LOC105372672, SUMO1P1, BCAS1, MIR4756, CYP24A1, PFDN4, DOK5, LINC01441, LINC01440, CBLN4, MC3R, FAM210B, AURKA, CSTF1, CASS4, RTF2, GCNT7, FAM209A, FAM209B, LINC01716, TFAP2C, BMP7, BMP7-AS1, LOC112268270, MIR4325, SPO11, RAE1, RBM38-AS1, RBM38, CTCFL, PCK1, ZBP1, PMEPA1, NKILA, LINC01742, C20orf85, ANKRD60, PPP4R1L, RAB22A, VAPB, APCDD1L, APCDD1L-DT, LINC01711, STX16-NPEPL1, STX16, NPEPL1, LOC105372695, MIR296, MIR298, GNAS-AS1, GNAS, LOC101927932, NELFCD, CTSZ, TUBB1, ATP5F1E, SLMO2-ATP5E, PRELID3B, ZNF831, EDN3, PHACTR3, PHACTR3-AS1, SYCP2, FAM217B, PPP1R3D, CDH26, LINC02910, LOC729296, MIR646HG, LOC105372698, MIR646, LOC101928048, MIR4533, MIR548AG2, LINC01718, CDH4, LOC100128310, MIR1257, TAF4, MIR3195, LSM14B, PSMA7, SS18L1, MTG2, HRH3, LOC105369209, OSBPL2, ADRM1, LAMA5, MIR4758, LAMA5-AS1, RPS21, CABLES2, RBBP8NL, GATA5, CRMA, MIR1-1HG, MIR1-1, MIR133A2, LINC02970, SLCO4A1, SLCO4A1-AS2, SLCO4A1-AS1, NTSR1, LINC00659, MRGBP, OGFR-AS1, OGFR, COL9A3, TCFL5, DIDO1, SNORA117, GID8, SLC17A9, BHLHE23, LINC01749, LINC00029, LINC01056, HAR1B, HAR1A, MIR124-3, YTHDF1, BIRC7, MIR3196, NKAIN4, FLJ16779, ARFGAP1, MIR4326, COL20A1, CHRNA4, LOC100130587, KCNQ2, EEF1A2, PPDPF, PTK6, SRMS, FNDC11, HELZ2, GMEB2, MHENCR, STMN3, RTEL1, RTEL1-TNFRSF6B, TNFRSF6B, ARFRP1, ZGPAT, LIME1, SLC2A4RG, ZBTB46, ZBTB46-AS1, LOC112268269, ABHD16B, TPD52L2, DNAJC5, MIR941-1, MIR941-2, MIR941-3, MIR941-4, MIR941-5, UCKL1, MIR1914, MIR647, UCKL1-AS1, ZNF512B, SAMD10, PRPF6, C20orf204, SOX18, TCEA2, RGS19, MIR6813, OPRL1, LKAAEAR1, NPBWR2, MYT1, PCMTD2 | arr[GRCh38] 20q11.21q13.33(31,247,164_64,284,202)x3 |
| 1  Brain | Gain | 21 | q22.2 | q22.3 | 8,081 | ERG, LINC00114, ETS2, LOC101928398, ETS2-AS1, LINC01700, PSMG1, BRWD1, BRWD1-AS2, BRWD1-AS1, HMGN1, GET1, GET1-SH3BGR, LCA5L, SH3BGR, MIR6508, B3GALT5-AS1, B3GALT5, IGSF5, PCP4, DSCAM, MIR4760, DSCAM-AS1, DSCAM-IT1, LINC00323, MIR3197, BACE2, PLAC4, FAM3B, MX2, MX1, TMPRSS2, PCSEAT, LINC00111, LINC00479, LINC00112, RIPK4, MIR6814, PRDM15, C2CD2, SNORA91, ZBTB21, ZNF295-AS1, UMODL1, UMODL1-AS1, ABCG1, TFF3, TFF2, TFF1, TMPRSS3, UBASH3A, RSPH1, RSPH1-DT, SLC37A1, LOC101928212, LINC01671, PDE9A, PDE9A-AS1, LINC01668, WDR4, NDUFV3, ERVH48-1, MIR5692B, PKNOX1, CBS, U2AF1, FRGCA, CRYAA, LINC00322, LINC01679, LNCSIK1, SIK1, LINC00319, LINC00313, HSF2BP, H2BC12L, MIR6070, RRP1B, PDXK, CSTB, RRP1, AATBC, AGPAT3, TRAPPC10, PWP2, GATD3, LINC01678, ICOSLG, DNMT3L, DNMT3L-AS1, AIRE, PFKL, CFAP410, TRPM2, TRPM2-AS, LRRC3-DT, LRRC3, LINC02575, TSPEAR, TSPEAR-AS1, TSPEAR-AS2, KRTAP10-1, KRTAP10-2, KRTAP10-3, KRTAP10-4, KRTAP10-5, KRTAP10-6, KRTAP10-7, KRTAP10-8, KRTAP10-9, KRTAP10-10, KRTAP10-11, KRTAP12-4, KRTAP12-3, KRTAP12-2, KRTAP12-1, KRTAP10-12, UBE2G2, LINC01424, SUMO3, PTTG1IP, ITGB2, ITGB2-AS1, LINC01547, SLX9, LINC00163, LINC00165, PICSAR, SSR4P1, ADARB1, LINC00334, POFUT2, LINC00205, LINC00316, BNAT1, COL18A1, COL18A1-AS2, COL18A1-AS1, MIR6815, SLC19A1, LINC01694, PCBP3, PCBP3-AS1, LOC101928796, COL6A1, COL6A2, FTCD, FTCD-AS1, SPATC1L, LSS, SNORD159, MCM3AP-AS1, MCM3AP, YBEY, C21orf58, PCNT, LOC128092249, DIP2A, DIP2A-IT1, S100B, PRMT2 | arr[GRCh38] 21q22.2q22.3(38,596,411_46,677,460)x3~4 |
| 1  Brain | Loss | X | p22.33 | p11.21 | 56,337 | PLCXD1, GTPBP6, LINC00685, PPP2R3B, SHOX, CRLF2, CSF2RA, MIR3690, IL3RA, SLC25A6, LINC00106, ASMTL-AS1, ASMTL, P2RY8, AKAP17A, ASMT, DHRSX, ZBED1, MIR6089, CD99P1, LINC00102, CD99, XG, GYG2, ARSD, ARSD-AS1, ARSL, ARSH, ARSF, LINC01546, MXRA5, SNORA48B, PRKX, PRKX-AS1, LOC389906, FAM239A, FAM239B, LOC101928201, NLGN4X, LOC105373156, MIR4770, VCX3A, PUDP, STS, MIR4767, VCX, PNPLA4, MIR651, VCX2, VCX3B, ANOS1, FAM9A, FAM9B, TBL1X, GPR143, SHROOM2, CLDN34, WWC3, CLCN4, MID1, HCCS, ARHGAP6, AMELX, MIR548AX, MSL3, FRMPD4, FRMPD4-AS1, PRPS2, TLR7, TLR8-AS1, TLR8, TMSB4X, FAM9C, LOC105373133, LINC02154, GS1-600G8.3, ATXN3L, LINC01203, EGFL6, MIR6086, LOC107985657, TCEANC, RAB9A, TRAPPC2, OFD1, GPM6B, GEMIN8, UBE2E4P, GLRA2, FANCB, MOSPD2, ASB9, ASB11, PIGA, PIR-FIGF, VEGFD, PIR, BMX, ACE2, ACE2-DT, CLTRN, CA5BP1, CA5BP1-CA5B, CA5B, INE2, ZRSR2, AP1S2, GRPR, MAGEB17, CTPS2, MIR548AM, S100G, SYAP1, TXLNG, RBBP7, REPS2, NHS, MIR4768, LOC101928389, NHS-AS1, SCML1, RAI2, LINC01456, BEND2, SCML2, CDKL5, RS1, PPEF1, PPEF1-AS1, PHKA2-AS1, PHKA2, ADGRG2, PDHA1, MAP3K15, SH3KBP1, BCLAF3, LOC729609, MAP7D2, MIR23C, EIF1AX, SCARNA9L, EIF1AX-AS1, RPS6KA3, CNKSR2, KLHL34, SMPX, MBTPS2, YY2, SMS, PHEX, PHEX-AS1, PTCHD1-AS, CBLL2, DDX53, PTCHD1, PRDX4, ACOT9, SAT1-DT, SAT1, LOC127933115, APOO, CXorf58, KLHL15, EIF2S3, ZFX-AS1, ZFX, SUPT20HL2, SUPT20HL1, PDK3, PCYT1B, PCYT1B-AS1, POLA1, SCARNA23, ARX, MAGEB18, MAGEB6B, MAGEB6, MAGEB5, VENTXP1, PPP4R3C, DCAF8L2, MAGEB10, DCAF8L1, MIR6134, IL1RAPL1, MIR4666B, MAGEB2, MAGEB3, MAGEB4, MAGEB1, NR0B1, TASL, GK, GK-AS1, TAB3, TAB3-AS1, FTHL17, DMD, MIR3915, MIR548F5, FAM47A, TMEM47, FAM47B, MAGEB16, CFAP47, LOC101928627, FAM47C, FTHL18P, PRRG1, LANCL3, XK, CYBB, DYNLT3, H2AP, H2AL3, SYTL5, MIR548AJ2, SRPX, RPGR, OTC, TSPAN7, MID1IP1-AS1, MID1IP1, LINC01281, LINC01282, MIR3937, MIR1587, BCOR, LOC107985687, LINC03099, ATP6AP2, MPC1L, CXorf38, MED14, MED14OS, TNIP2P1, USP9X, LINC02601, DDX3X, NYX, CASK, GPR34, GPR82, PPP1R2C, PINCR, MAOA, MAOB, NDP, NDP-AS1, EFHC2, FUNDC1, DUSP21, KDM6A, DIPK2B, LINC01204, MFFP3, MIR222HG, MIR221, MIR222, LINC02595, LINC01186, KRBOX4, ZNF674, ZNF674-AS1, CHST7, SLC9A7, RP2, LINC01545, JADE3, RGN, NDUFB11, RBM10, UBA1, INE1, CDK16, USP11, ZNF157, SNORA11C, ZNF41, LINC01560, ARAF, SYN1, TIMP1, MIR4769, CFP, ELK1, UXT, UXT-AS1, CXXC1P1, ZNF81, ZNF182, SPACA5, ZNF630-AS1, ZNF630, SSX6P, SPACA5B, SSX5, SSX1, SSX9P, SSX3, SSX4, SSX4B, SLC38A5, FTSJ1, PORCN-DT, PORCN, EBP, TBC1D25, RBM3, WDR13, WAS, SUV39H1, GLOD5, GATA1, HDAC6, ERAS, PCSK1N, TIMM17B, PQBP1, SLC35A2, PIM2, OTUD5, KCND1, GRIPAP1, TFE3, CCDC120, PRAF2, WDR45, GPKOW, MAGIX, PLP2, PRICKLE3, SYP, SYP-AS1, CACNA1F, CCDC22, FOXP3, FLICR, PPP1R3F, GAGE10, GAGE12J, GAGE13, GAGE12B, GAGE12C, GAGE12D, GAGE12E, GAGE12F, GAGE12G, GAGE12H, GAGE2A, GAGE1, PAGE1, PAGE4, USP27X-DT, USP27X, CLCN5, MIR532, MIR188, MIR500A, MIR362, MIR501, MIR500B, MIR660, MIR502, AKAP4, CCNB3, DGKK, SHROOM4, BMP15, LINC01284, NUDT10, EZHIP, NUDT11, LINC01496, CENPVL3, CENPVL2, CENPVL1, GSPT2, MAGED1, MAGED4B, SNORA11E, MAGED4, SNORA11D, MIR8088, XAGE2, XAGE1A, XAGE1B, SSX8P, SSX7, SSX2, SSX2B, SPANXN5, XAGE5, XAGE3, FAM156B, FAM156A, GPR173, TSPYL2, KANTR, KDM5C, MIR6895, MIR6894, IQSEC2, SMC1A, MIR6857, RIBC1, HSD17B10, HUWE1, MIR98, MIRLET7F2, PHF8, FAM120C, WNK3, TSR2, FGD1, GNL3L, ITIH6, MAGED2, SNORA11, TRO, SNORA11G, PFKFB1, APEX2, ALAS2, PAGE2B, PAGE2, FAM104B, SNORA109, PAGE5, PAGE3, LOC100421746, MIR4536-1, MIR4536-2, MAGEH1, USP51, FOXR2, RRAGB, KLF8, UBQLN2 | arr[GRCh38] Xp22.33p11.21(251,880_56,589,037)x1 |
| 2  Lung | Gain | 8 | q24.3 | q24.3 | 4,006 | DENND3-AS1, DENND3, SLC45A4, SLC45A4-AS1, LINC01300, GPR20, PTP4A3, MROH5, C8orf90, MIR1302-7, MIR4472-1, LINC00051, TSNARE1, ADGRB1, ARC, LOC101928087, JRK, PSCA, LY6K, LNCOC1, THEM6, SLURP1, LYPD2, SLURP2, LYNX1-SLURP2, LYNX1, LY6D, GML, CYP11B1, CYP11B2, LY6E-DT, CDC42P3, LY6E, LINC02904, LY6S, LY6L, LY6H, GPIHBP1, ZFP41, GLI4, MINCR, ZNF696, TOP1MT, RHPN1-AS1, RHPN1, MAFA-AS1, MAFA, ZC3H3, SNORD149, GSDMD, LOC100310756, MROH6, NAPRT, EEF1D, TIGD5, PYCR3, GFUS, ZNF623, ZNF707, LINC02878, CCDC166, LOC101928160, MAPK15, FAM83H, MIR4664, IQANK1, LOC105375800, SCRIB, MIR937, PUF60, NRBP2, MIR6845, EPPK1, PLEC, MIR661, PARP10, GRINA, SPATC1, SMPD5, OPLAH, MIR6846, EXOSC4, MIR6847, GPAA1, CYC1, SHARPIN, MAF1, WDR97, HGH1, MROH1, BOP1, MIR7112, SCX, HSF1, DGAT1, MIR6848, SCRT1, TMEM249, FBXL6, SLC52A2, LOC101928902, ADCK5, CPSF1, MIR939, MIR1234, MIR6849, SLC39A4, VPS28, TONSL, TONSL-AS1, MIR6893, ZFTRAF1, TMEM276-ZFTRAF1, MIR10400, TMEM276, KIFC2, FOXH1, PPP1R16A, GPT, MFSD3, RECQL4, LRRC14, LRRC24, C8orf82, ARHGAP39, ZNF251, ZNF34, RPL8, MIR6850, ZNF517, LOC100130027, ZNF7, COMMD5, ZNF250, ZNF16, ZNF252P, TMED10P1, ZNF252P-AS1, C8orf33 | arr[GRCh38] 8q24.3(141,064,057_145,070,385)x3~4 |
| 2  Lung | Gain | 12 | p13.33 | p13.32 | 3,241 | FBXL14, WNT5B, MIR3649, ADIPOR2, CACNA2D4, LRTM2, LINC00940, DCP1B, CACNA1C-IT2, CACNA1C, CACNA1C-AS4, CACNA1C-IT3, CACNA1C-AS2, CACNA1C-AS1, ITFG2-AS1, FKBP4, ITFG2, NRIP2, TEX52, FOXM1, RHNO1, TULP3, TEAD4, TSPAN9, LOC100128253, LINC02417, PRMT8, THCAT155, CRACR2A, PARP11, PARP11-AS1, CCND2-AS1, CCND2, TIGAR, FGF23, FGF6, C12orf4, RAD51AP1, DYRK4, AKAP3, NDUFA9, GAU1, GALNT8 | arr[GRCh38] 12p13.33p13.32(1,537,246_4,778,644)x3~4 |
| 2  Lung | Gain | 20 | q12 | q13.12 | 6,901 | LINC01734, LINC01370, MAFB, SNORD154, LOC100128988, TOP1, PLCG1-AS1, PLCG1, MIR6871, ZHX3, LPIN3, EMILIN3, CHD6, PTPRT, LOC101927159, PTPRT-AS1, SRSF6, L3MBTL1, SGK2, IFT52, MYBL2, GTSF1L, LINC01728, TOX2, JPH2, OSER1, OSER1-DT, GDAP1L1, FITM2, R3HDML, R3HDML-AS1, HNF4A, HNF4A-AS1, MIR3646, LINC01430, LINC01620, TTPAL, SERINC3, PKIG, ADA, LINC01260, KCNK15-AS1, CCN5, KCNK15, RIMS4, YWHAB, PABPC1L, TOMM34, STK4-DT, STK4, KCNS1, WFDC5, WFDC12, PI3, SEMG1, SEMG2, SLPI, MATN4, RBPJL, SDC4, SYS1, SYS1-DBNDD2, TP53TG5, DBNDD2, PIGT, MIR6812, LOC105372631, WFDC2, SPINT3, WFDC6, EPPIN-WFDC6, EPPIN, WFDC8, WFDC9, WFDC10A, WFDC11, WFDC10B, WFDC13, MIR3617, SPINT4, WFDC3, DNTTIP1, UBE2C, TNNC2, SNX21, ACOT8, ZSWIM3, ZSWIM1, SPATA25, NEURL2, CTSA, PLTP, LOC107985388, PCIF1, ZNF335 | arr[GRCh38] 20q12q13.12(39,067,571_45,968,108)x3~4 |
| 2  Lung | Gain | 20 | q13.2 | q13.33 | 8,387 | CBLN4, MC3R, FAM210B, AURKA, CSTF1, CASS4, RTF2, GCNT7, FAM209A, FAM209B, LINC01716, TFAP2C, BMP7, BMP7-AS1, LOC112268270, MIR4325, SPO11, RAE1, RBM38-AS1, RBM38, CTCFL, PCK1, ZBP1, PMEPA1, NKILA, LINC01742, C20orf85, ANKRD60, PPP4R1L, RAB22A, VAPB, APCDD1L, APCDD1L-DT, LINC01711, STX16-NPEPL1, STX16, NPEPL1, LOC105372695, MIR296, MIR298, GNAS-AS1, GNAS, LOC101927932, NELFCD, CTSZ, TUBB1, ATP5F1E, SLMO2-ATP5E, PRELID3B, ZNF831, EDN3, PHACTR3, PHACTR3-AS1, SYCP2, FAM217B, PPP1R3D, CDH26, LINC02910, LOC729296, MIR646HG, LOC105372698, MIR646, LOC101928048, MIR4533, MIR548AG2, LINC01718, CDH4, LOC100128310, MIR1257, TAF4, MIR3195, LSM14B, PSMA7, SS18L1, MTG2, HRH3, LOC105369209, OSBPL2, ADRM1, LAMA5, MIR4758, LAMA5-AS1, RPS21, CABLES2, RBBP8NL, GATA5, CRMA, MIR1-1HG, MIR1-1, MIR133A2, LINC02970, SLCO4A1, SLCO4A1-AS2, SLCO4A1-AS1, NTSR1, LINC00659, MRGBP, OGFR-AS1, OGFR, COL9A3, TCFL5, DIDO1, SNORA117, GID8, SLC17A9, BHLHE23, LINC01749, LINC00029, LINC01056, HAR1B, HAR1A, MIR124-3, YTHDF1, BIRC7, MIR3196, NKAIN4, FLJ16779, ARFGAP1, MIR4326, COL20A1, CHRNA4, LOC100130587, KCNQ2, EEF1A2, PPDPF, PTK6, SRMS, FNDC11, HELZ2, GMEB2, MHENCR, STMN3, RTEL1, RTEL1-TNFRSF6B, TNFRSF6B, ARFRP1, ZGPAT, LIME1, SLC2A4RG, ZBTB46, ZBTB46-AS1, LOC112268269, ABHD16B, TPD52L2, DNAJC5, MIR941-1, MIR941-2, MIR941-3, MIR941-4, MIR941-5, UCKL1, MIR1914, MIR647, UCKL1-AS1, ZNF512B, SAMD10, PRPF6, C20orf204, SOX18, TCEA2, RGS19, MIR6813, OPRL1, LKAAEAR1, NPBWR2, MYT1, PCMTD2 | arr[GRCh38] 20q13.2q13.33(55,897,220_64,284,202)x3~4 |
| 2  Lung | Loss | X | p22.33 | p22.32 | 3,199 | ARSD, ARSD-AS1, ARSL, ARSH, ARSF, LINC01546, MXRA5, SNORA48B, PRKX, PRKX-AS1, LOC389906, FAM239A, FAM239B, LOC101928201, NLGN4X | arr[GRCh38] Xp22.33p22.32(2,883,424_6,082,234)x1~2 |
| 2  Brain | Gain | 1 | p36.33 | p36.21 | 14,323 | LINC02593, LOC107985728, SAMD11, NOC2L, KLHL17, PLEKHN1, PERM1, HES4, ISG15, AGRN, LOC100288175, LOC105378948, RNF223, C1orf159, LINC01342, MIR200B, MIR200A, MIR429, TTLL10-AS1, TTLL10, TNFRSF18, TNFRSF4, SDF4, B3GALT6, C1QTNF12, UBE2J2, SCNN1D, ACAP3, MIR6726, SNORD167, PUSL1, INTS11, MIR6727, CPTP, TAS1R3, DVL1, MIR6808, MXRA8, AURKAIP1, CCNL2, MRPL20-AS1, MRPL20, MRPL20-DT, ANKRD65, TMEM88B, LINC01770, VWA1, ATAD3C, ATAD3B, ATAD3A, TMEM240, SSU72, FNDC10, LOC105378586, MIB2, MMP23B, CDK11B, SLC35E2B, MMP23A, CDK11A, SLC35E2A, NADK, GNB1, GNB1-DT, CALML6, TMEM52, CFAP74, GABRD, PRKCZ-DT, PRKCZ, PRKCZ-AS1, FAAP20, LOC112268219, SKI, MORN1, LOC100129534, RER1, PEX10, PLCH2, PANK4, HES5, TNFRSF14-AS1, TNFRSF14, LOC100996583, PRXL2B, MMEL1, MMEL1-AS1, TTC34, ACTRT2, PRDM16-DT, PRDM16, MIR4251, ARHGEF16, MEGF6, MIR551A, TPRG1L, WRAP73, TP73, TP73-AS1, CCDC27, SMIM1, LRRC47, CEP104, DFFB, C1orf174, LINC01134, LINC01346, LINC01345, LINC01777, LINC01646, AJAP1, LINC02782, MIR4689, NPHP4, KCNAB2, CHD5, RPL22, RNF207-AS1, RNF207, ICMT, ICMT-DT, HES3, GPR153, ACOT7, HES2, ESPN, MIR4252, TNFRSF25, PLEKHG5, NOL9, TAS1R1, ZBTB48, KLHL21, PHF13, THAP3, DNAJC11, LINC01672, CAMTA1-DT, CAMTA1, CAMTA1-AS3, CAMTA1-AS2, VAMP3, PER3, UTS2, TNFRSF9, PARK7, ERRFI1, LINC01714, SLC45A1, RERE, RERE-AS1, SNORD128, ENO1, MIR6728, ENO1-AS1, CA6, SLC2A7, SLC2A5, GPR157, MIR34AHG, MIR34A, LNCTAM34A, H6PD, SPSB1, LINC02606, SLC25A33, TMEM201, PIK3CD, PIK3CD-AS1, PIK3CD-AS2, CLSTN1, CTNNBIP1, LZIC, NMNAT1, TMEM274P, MIR5697, RBP7, UBE4B, KIF1B, PGD, CENPS, CENPS-CORT, CORT, DFFA, PEX14, CASZ1, C1orf127, TARDBP, MASP2, SRM, EXOSC10, EXOSC10-AS1, MTOR, MTOR-AS1, ANGPTL7, UBIAD1, DISP3, LINC01647, FBXO2, FBXO44, FBXO6, MAD2L2, DRAXIN, AGTRAP, C1orf167, C1orf167-AS1, MTHFR, CLCN6, NPPA-AS1, NPPA, NPPB, RNU5E-1, KIAA2013, PLOD1, MFN2, MIIP, MIR6729, TNFRSF8, MIR7846, TNFRSF1B, MIR4632, VPS13D, SNORA59A, DHRS3, MIR6730, AADACL4, AADACL3, CFAP107, PRAMEF12, PRAMEF1, LINC01784, PRAMEF11, HNRNPCL1, PRAMEF2, PRAMEF4, PRAMEF10, PRAMEF7, PRAMEF6, PRAMEF27, HNRNPCL3, PRAMEF25, PRAMEF34P, HNRNPCL2, PRAMEF36P, PRAMEF26, HNRNPCL4, PRAMEF9, PRAMEF13, PRAMEF18, PRAMEF5, PRAMEF8, PRAMEF33, PRAMEF15, PRAMEF14, PRAMEF19, PRAMEF17, PRAMEF20, LRRC38, PDPN, PRDM2, KAZN-AS1, KAZN, TMEM51-AS1, TMEM51, TMEM51-AS2 | arr[GRCh38] 1p36.33p36.21(914,087_15,236,739)x3~4 |
| 2  Brain | cn-LOH | 1 | p36.21 | p34.3 | 23,024 | FHAD1, EFHD2-AS1, EFHD2, CTRC, CELA2A, CELA2B, CASP9, DNAJC16, SCARNA21B, AGMAT, DDI2, RSC1A1, PLEKHM2, SLC25A34, SLC25A34-AS1, TMEM82, FBLIM1, UQCRHL, SPEN-AS1, SPEN, ZBTB17, SRARP, HSPB7, CLCNKA, CLCNKB, FAM131C, EPHA2, ARHGEF19, CPLANE2, FBXO42, SZRD1, SPATA21, NECAP2, LINC01772, CROCCP3, RNU1-1, LINC01783, NBPF1, MST1P2, RNU1-3, ESPNP, RNU1-4, MST1L, MIR3675, LOC112267871, LOC105376805, RNU1-2, CROCC, MFAP2, ATP13A2, SDHB, PADI2, LINC02783, PADI1, PADI3, MIR3972, PADI4, PADI6, RCC2, RCC2-AS1, ARHGEF10L, ACTL8, LINC01654, IGSF21, KLHDC7A, PAX7, TAS1R2, ALDH4A1, MIR4695, MIR1290, IFFO2, UBR4, EMC1-AS1, EMC1, MRTO4, AKR7L, AKR7A3, LOC100506730, AKR7A2, SLC66A1, CAPZB, MICOS10-DT, MICOS10, MICOS10-NBL1, RPS14P3, NBL1, HTR6, TMCO4, RNF186, OTUD3, PLA2G2E, PLA2G2A, PLA2G5, PLA2G2D, PLA2G2F, LOC117779438, PLA2G2C, UBXN10, LINC01757, VWA5B1, LINC01141, CAMK2N1, MUL1, FAM43B, CDA, PINK1, MIR6084, PINK1-AS, DDOST, KIF17, SH2D5, HP1BP3, EIF4G3, MIR1256, ECE1, ECE1-AS1, NBPF3, ALPL, RAP1GAP, USP48, LDLRAD2, HSPG2, CELA3B, CELA3A, LINC01635, LINC00339, CDC42, WNT4, MIR4418, ZBTB40, EPHA8, MIR6127, C1QA, C1QC, C1QB, EPHB2, MIR4684, MIR4253, LACTBL1, TEX46, KDM1A, MIR3115, LUZP1, HTR1D, LINC01355, HNRNPR, ZNF436, ZNF436-AS1, TCEA3, ASAP3, E2F2, LOC101928163, ID3, MDS2, RPL11, ELOA, ELOA-AS1, PITHD1, LYPLA2, GALE, HMGCL, FUCA1, CNR2, MIR378F, PNRC2, SRSF10, MYOM3, IL22RA1, IFNLR1, LINC02800, GRHL3-AS1, GRHL3, STPG1, NIPAL3, RCAN3AS, RCAN3, NCMAP-DT, NCMAP, SRRM1, CLIC4, RUNX3, MIR6731, RUNX3-AS1, MIR4425, SYF2, RSRP1, RHD, TMEM50A, RHCE, MACO1, LDLRAP1, MAN1C1, SELENON, LOC646471, MTFR1L, AUNIP, PAQR7, STMN1, MIR3917, PAFAH2, EXTL1, SLC30A2, TRIM63, PDIK1L, FAM110D, C1orf232, ZNF593OS, ZNF593, CNKSR1, CATSPER4, ZPLD2P, CEP85, SH3BGRL3, UBXN11, CD52, CRYBG2, ZNF683, LIN28A, DHDDS, DHDDS-AS1, HMGN2, RPS6KA1, MIR1976, LOC101928728, ARID1A, PIGV, ZDHHC18, SFN, GPN2, GPATCH3, NR0B2, NUDC, KDF1, TRNP1, TENT5B, SLC9A1, WDTC1, TMEM222, ACTG1P20, SYTL1, MAP3K6, FCN3, CD164L2, GPR3, WASF2, AHDC1, FGR, LINC02574, IFI6, FAM76A, STX12, PPP1R8, SCARNA1, THEMIS2, RPA2, SMPDL3B, XKR8, EYA3, PTAFR, DNAJC8, ATP5IF1, SESN2, MED18, PHACTR4, SNHG3, RCC1, SNORA73A, SNORA73B, TRNAU1AP, SNHG12, SNORD99, SNORA61, SNORA44, SNORA16A, RAB42, TAF12, TAF12-DT, RNU11, GMEB1, YTHDF2, OPRD1, EPB41, TMEM200B, SRSF4, MECR, PTPRU, LINC01756, LINC01648, MATN1, MATN1-AS1, LAPTM5, MIR4420, LINC01778, SDC3, PUM1, SNORD103A, SNORD103B, SNORD103C, NKAIN1, SNRNP40, ZCCHC17, FABP3, SERINC2, LDC1P, LINC01226, TINAGL1, HCRTR1, PEF1, PEF1-AS1, COL16A1, ADGRB2, MIR4254, SPOCD1, PTP4A2, LOC128031832, KHDRBS1, TMEM39B, MIR5585, KPNA6, TXLNA, CCDC28B, IQCC, DCDC2B, TMEM234, EIF3I, MTMR9LP, FAM167B, LCK, HDAC1, MARCKSL1, FAM229A, TSSK3, BSDC1, ZBTB8B, ZBTB8A, ZBTB8OS, RBBP4, SYNC, KIAA1522, YARS1, S100PBP, FNDC5, HPCA, TMEM54, RNF19B, AK2, AZIN2, TRIM62, ZNF362, A3GALT2, PHC2, MIR3605, PHC2-AS1, ZSCAN20, CSMD2, HMGB4, CSMD2-AS1, C1orf94, MIR552, GJB5, GJB4, GJB3, GJA4, SMIM12, DLGAP3, GPR199P, TMEM35B, ZMYM6, ZMYM1, SFPQ, ZMYM4, ZMYM4-AS1, KIAA0319L, NCDN, TFAP2E-AS1, TFAP2E, PSMB2, C1orf216, CLSPN, AGO4, AGO1, AGO3, TEKT2, ADPRS, COL8A2, TRAPPC3, MAP7D1, THRAP3, SH3D21, EVA1B, STK40, LSM10, OSCP1, SNORA63C, MRPS15, CSF3R, GRIK3, MIR4255, ZC3H12A-DT, ZC3H12A, MIR6732, MEAF6, MIR5581, SNIP1, DNALI1, GNL2, RSPO1, C1orf109, CDCA8, EPHA10, MANEAL, YRDC, C1orf122, MTF1, INPP5B, SF3A3, FHL3, UTP11, POU3F1, MIR3659HG, MIR3659, LINC01343 | arr[GRCh38] 1p36.21p34.3(15,372,831_38,396,459)x2 hmz |
| 2  Brain | Gain | 1 | p34.3 | p12 | 81,564 | LINC01685, RRAGC, MYCBP, GJA9-MYCBP, LOC105378663, GJA9, RHBDL2, AKIRIN1, NDUFS5, MACF1, BMP8A, OXCT2P1, PPIEL, PABPC4, PABPC4-AS1, SNORA55, HEYL, NT5C1A, HPCAL4, PPIE, BMP8B, OXCT2, LOC101929536, TRIT1, MYCL-AS1, MYCL, MFSD2A, CAP1, PPT1, RLF, TMCO2, ZMPSTE24, COL9A2, SMAP2, ZFP69B, ZFP69, EXO5, ZNF684, RIMS3, NFYC-AS1, NFYC, MIR30E, MIR30C1, KCNQ4, CITED4, CTPS1, SLFNL1-AS1, SLFNL1, SCMH1, FOXO6, FOXO6-AS1, EDN2, HIVEP3, LOC128125817, GUCA2B, GUCA2A, FOXJ3, RIMKLA, ZMYND12, CCDC30, PPCS, PPIH, YBX1, CLDN19, P3H1, C1orf50, TMEM269-DT, TMEM269, SVBP, ERMAP, ZNF691, LOC339539, SLC2A1, SLC2A1-DT, CFAP144, EBNA1BP2, MIR6733, CFAP57, TMEM125, C1orf210, TIE1, MPL, CDC20-DT, CDC20, ELOVL1, MIR6734, MED8, SZT2, SZT2-AS1, MIR6735, HYI, HYI-AS1, PTPRF, KDM4A, KDM4A-AS1, ST3GAL3, ST3GAL3-AS1, MIR6079, ARTN, IPO13, DPH2, ATP6V0B, B4GALT2, CCDC24, SLC6A9, KLF17, KLF18, DMAP1, ERI3, ERI3-IT1, SNORA110, RNF220, MIR5584, TMEM53, SNORD145, ARMH1, RNU5F-1, RNU5D-1, KIF2C, SNORD160, RPS8, SNORD55, SNORD46, SNORD38A, SNORD38B, BEST4, PLK3, DYNLT4, BTBD19, PTCH2, EIF2B3, HECTD3, UROD, ZSWIM5, LINC01144, HPDL, MUTYH, TOE1, TESK2, CCDC163, MMACHC, PRDX1, AKR1A1, NASP, CCDC17, GPBP1L1, RPS15AP10, TMEM69, IPP, MAST2, PIK3R3, P3R3URF-PIK3R3, LOC101929626, TSPAN1, P3R3URF, POMGNT1, LURAP1, RAD54L, LRRC41, UQCRH, NSUN4, FAAH, FAAHP1, LINC01398, DMBX1, TMEM275, MKNK1-AS1, KNCN, MKNK1, MOB3C, ATPAF1, TEX38, EFCAB14-AS1, EFCAB14, CYP4B1, CYP4Z2P, CYP4A11, CYP4X1, CYP4Z1, CYP4A22, LINC00853, PDZK1IP1, TAL1, STIL, CMPK1, LINC01389, FOXE3, FOXD2-AS1, FOXD2, TRABD2B, SKINT1L, SLC5A9, SPATA6, AGBL4, BEND5, AGBL4-AS1, AGBL4-IT1, ELAVL4, DMRTA2, FAF1, CDKN2C, MIR4421, MIR6500, C1orf185, LINC01562, RNF11, TTC39A, TTC39A-AS1, EPS15, EPS15-AS1, OSBPL9, CALR4P, NRDC, MIR761, RAB3B, TXNDC12, KTI12, TXNDC12-AS1, BTF3L4, ZFYVE9, CC2D1B, ORC1, PRPF38A, TUT4, GPX7, SHISAL2A, COA7, ZYG11B, ZYG11A, ECHDC2, SCP2, PODN, SLC1A7, CPT2, CZIB, MAGOH, MAGOH-DT, LRP8, LRP8-DT, SLC25A3P1, DMRTB1, GLIS1, NDC1, YIPF1, DIO1, IFT25, LRRC42, LDLRAD1, TMEM59, TCEANC2, MIR4781, CDCP2, CYB5RL, MRPL37, SSBP3, SSBP3-AS1, LINC02784, ACOT11, FAM151A, MROH7-TTC4, MROH7, TTC4, PARS2, TTC22, LEXM, DHCR24, TMEM61, BSND, PCSK9, USP24, LOC100507634, MIR4422HG, MIR4422, LINC01753, LINC01755, LINC01767, PLPP3, LOC101929935, PRKAA2, FYB2, C8A, C8B, DAB1, DAB1-AS1, OMA1, TACSTD2, MYSM1, JUN, LINC01135, LINC02777, LINC01358, HSD52, FGGY, MIR4711, LOC101926944, HOOK1, CYP2J2, C1orf87, LINC01748, LOC101926964, NFIA-AS2, NFIA, NFIA-AS1, MGC34796, TM2D1, PATJ, MIR12132, MIR3116-1, MIR3116-2, L1TD1, KANK4, USP1, DOCK7, ANGPTL3, ATG4C, LINC01739, LINC00466, FOXD3-AS1, FOXD3, MIR6068, ALG6, ITGB3BP, EFCAB7, DLEU2L, PGM1, ROR1, ROR1-AS1, UBE2U, CACHD1, MIR4794, RAVER2, JAK1, LINC01359, MIR3671, MIR101-1, AK4, DNAJC6, LEPR, LEPROT, PDE4B, PDE4B-AS1, SGIP1, MIR3117, DYNLT5, INSL5, DNAI4, MIER1, SLC35D1, C1orf141, IL23R, IL12RB2, SERBP1, GADD45A, GNG12, GNG12-AS1, DIRAS3, WLS, MIR1262, RPE65, DEPDC1, DEPDC1-AS1, LINC01707, LINC02791, LINC01758, LRRC7, PIN1P1, LRRC40, SRSF11, ANKRD13C, HHLA3, HHLA3-AS1, CTH, LINC01788, PTGER3, ZRANB2-AS1, ZRANB2, MIR186, ZRANB2-DT, NEGR1, NEGR1-IT1, LINC01360, LINC02238, LRRIQ3, FPGT, FPGT-TNNI3K, TNNI3K, LRRC53, ERICH3, ERICH3-AS1, CRYZ, TYW3, LHX8, SLC44A5, ACADM, DLSTP1, RABGGTB, SNORD45C, SNORD45A, SNORD45B, MSH4, ASB17, LOC101927342, ST6GALNAC3, LINC02567, ST6GALNAC5, MIR7156, PIGK, AK5, ZZZ3, USP33, MIGA1, NEXN-AS1, NEXN, FUBP1, DNAJB4, GIPC2, MGC27382, PTGFR, IFI44L, IFI44, ADGRL4, LINC01781, ADGRL2, LOC101927434, LINC01362, LINC01361, LINC01712, LINC01725, LOC101927560, TTLL7, TTLL7-IT1, PRKACB, SAMD13, UOX, DNASE2B, RPF1, GNG5, SPATA1, CTBS, LINC01461, LINC01555, SSX2IP, LPAR3, MCOLN2, MCOLN3, DNAI3, MIR4423, SYDE2, C1orf52, BCL10, BCL10-AS1, DDAH1, CCN1, ZNHIT6, COL24A1, ODF2L, MIR7856, CLCA2, CLCA1, CLCA4, CLCA4-AS1, CLCA3P, SH3GLB1, SELENOF, HS2ST1, LINC01140, LINC02801, LMO4, LINC01364, PKN2-AS1, PKN2, GTF2B, KYAT3, RBMXL1, GBP3, GBP1, GBP2, GBP7, GBP4, GBP5, LOC729930, GBP6, GBP1P1, LRRC8B, LOC128092251, LRRC8C-DT, LRRC8C, LRRC8D, GEMIN8P4, ZNF326, SNORD3G, BARHL2, LINC02609, LINC01763, ZNF644, HFM1, CDC7, TGFBR3, BRDT, EPHX4, SETSIP, BTBD8, C1orf146, GLMN, RPAP2, GFI1, EVI5, RPL5, DIPK1A, SNORD21, SNORA66, MTF2, TMED5, CCDC18, CCDC18-AS1, DR1, FNBP1L, BCAR3, BCAR3-AS1, MIR760, DNTTIP2, GCLM, ABCA4, ARHGAP29, ABCD3, F3, MIR12133, SLC44A3-AS1, MIR378G, SLC44A3, CNN3, CNN3-DT, ALG14, ALG14-AS1, TLCD4, TLCD4-RWDD3, RWDD3-DT, RWDD3, LINC01760, LINC01650, LINC01761, LINC02607, LINC02790, LINC01787, PTBP2, DPYD, DPYD-AS1, DPYD-AS2, LINC01930, MIR137HG, MIR2682, MIR137, LINC01776, SNX7, PLPPR5, PLPPR5-AS1, PLPPR4, LINC01708, PALMD, FRRS1, AGL, SLC35A3, MFSD14A, SASS6, TRMT13, LRRC39, DBT, RTCA-AS1, RTCA, MIR553, CDC14A, GPR88, LINC01349, VCAM1, EXTL2, SLC30A7, DPH5, DPH5-DT, S1PR1-DT, S1PR1, LINC01307, LINC01709, OLFM3, DNAJA1P5, COL11A1, RNPC3-DT, RNPC3, AMY2B, ACTG1P4, AMY2A, AMY1A, AMY1B, AMY1C, LOC100129138, LINC01676, LINC01677, LINC01661, PRMT6, NTNG1, VAV3, MIR7852, VAV3-AS1, SLC25A24, NBPF4, NBPF6, EEIG2, HENMT1, PRPF38B, FNDC7, STXBP3, AKNAD1, SPATA42, GPSM2, CLCC1, WDR47, TAF13, TMEM167B, SCARNA2, CFAP276, ELAPOR1, SARS1, CELSR2, PSRC1, MYBPHL, SORT1, PSMA5, SYPL2, ATXN7L2, CYB561D1, AMIGO1, GPR61, GNAI3, MIR197, GNAT2, AMPD2, GSTM4, GSTM2, GSTM1, GSTM5, GSTM3, EPS8L3, CSF1, AHCYL1, STRIP1, ALX3, LINC01397, UBL4B, SLC6A17, SLC6A17-AS1, KCNC4-DT, KCNC4, RBM15-AS1, RBM15, SLC16A4, LAMTOR5, LAMTOR5-AS1, PROK1, CYMP, CYMP-AS1, KCNA10, KCNA2, KCNA3, CD53, LRIF1, DRAM2, CEPT1, DENND2D, CHI3L2, CHIAP2, CHIA, PIFO, PGBP, OVGP1, WDR77, ATP5PB, C1orf162, TMIGD3, ADORA3, RAP1A, LINC01160, LOC107985184, INKA2, INKA2-AS1, LOC101928718, DDX20, KCND3, KCND3-IT1, KCND3-AS1, LINC01750, LINC02884, CTTNBP2NL, MIR4256, WNT2B, ST7L, CAPZA1, MOV10, RHOC, PPM1J, TAFA3, LINC01356, MIR11399, SLC16A1, AKR7A2P1, SLC16A1-AS1, LRIG2-DT, LRIG2, LOC643441, MAGI3, PHTF1, RSBN1, AP4B1-AS1, PTPN22, BCL2L15, AP4B1, DCLRE1B, HIPK1-AS1, HIPK1, OLFML3, SYT6, TRIM33, BCAS2, DENND2C, AMPD1, NRAS, CSDE1, SIKE1, SYCP1, TSHB, TSPAN2, NGF-AS1, NGF, VANGL1, CASQ2, NHLH2, LINC01649, LOC101928977, SLC22A15, MAB21L3, ATP1A1, ATP1A1-AS1, LINC01762, CD58, MIR548AC, IGSF3, MIR320B1, LINC02868, CD2, PTGFRN, CD101, CD101-AS1, TTF2, MIR942, TRIM45, VTCN1, LINC01525, MAN1A2, TENT5C-DT, TENT5C, GDAP2, WDR3, SPAG17, TBX15, LOC105378933, WARS2, WARS2-IT1, WARS2-AS1, LINC01780, HAO2, HAO2-IT1, HSD3B2, HSD3B1, HSD3BP4, LINC00622, ZNF697, PHGDH, HMGCS2, REG4, NBPF7P, ADAM30, NOTCH2 | arr[GRCh38] 1p34.3p12(38,415,043_119,979,439)x3 |
| 2  Brain | Gain | 1 | q21.1 | q44 | 103,631 | LINC01145, LOC100996740, PPIAL4D, RNVU1-14, NBPF20, NBPF25P, GPR89A, PDZK1, CD160, RNF115, POLR3C, NUDT17, PIAS3, MIR6736, ANKRD35, ITGA10, PEX11B, GNRHR2, RBM8A, LIX1L-AS1, LIX1L, ANKRD34A, POLR3GL, TXNIP, HJV, RNVU1-6, LINC01719, NBPF10, NOTCH2NLA, NUDT4P2, PPIAL4H, HYDIN2, NBPF12, LOC728989, RNVU1-8, NBPF13P, PRKAB2, CHD1L, PDIA3P1, FMO5, LINC00624, BCL9, LOC128071544, ACP6, GJA5, GJA8, GPR89B, PDZK1P1, RNVU1-7, PDE4DIPP1, NBPF11, LINC02805, LOC101927468, LINC01731, MIR5087, RNVU1-1, MIR6077, PDE4DIPP6, RNVU1-3, LINC01138, PPIAL4G, NBPF14, NOTCH2NLB, NUDT4B, SEC22B3P, PDE4DIP, NBPF9, LOC653513, SEC22B2P, NOTCH2NLC, NBPF19, PPIAL4C, LINC00869, LOC644634, H2BC18, FCGR1A, H3C13, H4C14, H3C14, H2AC18, H2BC20P, H2AC19, H3C15, H4C15, H2BC21, H2AC20, H2AC21, BOLA1, SV2A, SF3B4, MTMR11, OTUD7B, VPS45, PLEKHO1, LINC02988, ANP32E, CA14, SNORD13C, APH1A, C1orf54, CIART, MRPS21, PRPF3, RPRD2, TARS2, MIR6878, ECM1, FALEC, ADAMTSL4, MIR4257, ADAMTSL4-AS1, MCL1, ENSA, GOLPH3L, HORMAD1, CTSS, CTSK, ARNT, CTXND2, SETDB1, CERS2, ANXA9, MINDY1, PRUNE1, BNIPL, C1orf56, CDC42SE1, MLLT11, GABPB2, SEMA6C, TNFAIP8L2, TNFAIP8L2-SCNM1, LYSMD1, SCNM1, TMOD4, VPS72, PIP5K1A, PSMD4, ZNF687-AS1, ZNF687, PI4KB, RFX5, RFX5-AS1, SELENBP1, PSMB4, POGZ, CGN, TUFT1, MIR554, SNX27, CELF3, RIIAD1, MRPL9, OAZ3, TDRKH, TDRKH-AS1, LINGO4, RORC, C2CD4D, C2CD4D-AS1, THEM5, THEM4, S100A10, NBPF18P, S100A11, LOC100131107, TCHHL1, TCHH, RPTN, HRNR, FLG, FLG-AS1, FLG2, CRNN, LCE5A, CRCT1, LCE3E, LCE3D, LCE3C, LCE3B, LCE3A, LINC00302, LCE2D, LCE2C, LCE2B, LCE2A, LCE4A, KPLCE, KPRP, LCE1F, LCE1E, LCE1D, LCE1C, LCE1B, LCE1A, LCE6A, LCE7A, SMCP, IVL, LINC01527, SPRR5, SPRR4, SPRR1A, SPRR3, SPRR1B, SPRR2D, SPRR2A, SPRR2B, SPRR2E, SPRR2F, SPRR2C, SPRR2G, LOC101928009, LELP1, PRR9, LORICRIN, PGLYRP3, PGLYRP4, S100A9, S100A12, S100A8, S100A7A, S100A7, S100A6, S100A5, S100A4, LOC101928034, S100A3, S100A2, S100A16, S100A14, S100A13, S100A1, CHTOP, SNAPIN, ILF2, NPR1, MIR8083, INTS3, SLC27A3, LOC343052, GATAD2B, DENND4B, CRTC2, SLC39A1, MIR6737, CREB3L4, JTB, RAB13, RPS27, NUP210L, MIR5698, TPM3, MIR190B, CFAP141, C1orf43, UBAP2L, SNORA58B, HAX1, AQP10, ATP8B2, IL6R-AS1, IL6R, SHE, TDRD10, UBE2Q1, UBE2Q1-AS1, CHRNB2, ADAR, KCNN3, PMVK, PBXIP1, PYGO2, LOC101928120, SHC1, CKS1B, MIR4258, FLAD1, LENEP, ZBTB7B, DCST2, DCST1, DCST1-AS1, ADAM15, ADAM15-EFNA4, EFNA4, EFNA4-EFNA3, EFNA3, EFNA1, SLC50A1, DPM3, KRTCAP2, TRIM46, MUC1, THBS3-AS1, MIR92B, THBS3, MTX1, GBAP1, GBA1, ENTREP3, SCAMP3, CLK2, HCN3, PKLR, FDPS, RUSC1-AS1, RUSC1, ASH1L, MIR555, POU5F1P4, ASH1L-AS1, MSTO1, YY1AP1, SCARNA26A, DAP3, MSTO2P, GON4L, SCARNA26B, SYT11, RIT1, KHDC4, SNORA80E, SCARNA4, RXFP4, ARHGEF2, MIR6738, ARHGEF2-AS2, SSR2, UBQLN4, LAMTOR2, RAB25, MEX3A, LMNA, SEMA4A, SLC25A44, PMF1-BGLAP, PMF1, BGLAP, PAQR6, SMG5, TMEM79, GLMP, VHLL, CCT3, TSACC, RHBG, MIR9-1HG, MIR9-1, MEF2D, IQGAP3, TTC24, NAXE, GPATCH4, LOC101928177, HAPLN2, BCAN, BCAN-AS2, NES, CRABP2, ISG20L2, METTL25B, MRPL24, HDGF, PRCC, SH2D2A, NTRK1, INSRR, PEAR1, LRRC71, ARHGEF11, MIR765, ETV3L, ETV3, CYCSP52, LINC02772, FCRL5, FCRL4, FCRL3, FCRL2, FCRL1, CD5L, LOC105371458, KIRREL1, KIRREL1-IT1, SMIM42, LINC01704, CD1D, CD1A, CD1C, CD1B, CD1E, OR10T2, OR10K2, OR10K1, OR10R2, OR6Y1, OR6P1, OR10X1, OR10Z1, SPTA1, OR6K2, OR6K3, OR6K6, OR6N1, OR6N2, MNDA, PYHIN1, PYDC5, IFI16, AIM2, CADM3, CADM3-AS1, ACKR1, FCER1A, OR10J3, OR10J1, OR10J4, OR10J5, APCS, CRP, DUSP23, FCRL6, SLAMF8, SNHG28, VSIG8, CFAP45, MIR4259, TAGLN2, IGSF9, SLAMF9, LINC01133, PIGM, KCNJ10, KCNJ9, IGSF8, ATP1A2, ATP1A4, CASQ1, LOC729867, PEA15, DCAF8, DCAF8-DT, PEX19, COPA, SUMO1P3, NCSTN, NHLH1, VANGL2, SLAMF6, CD84, SLAMF1, CD48, SLAMF7, LY9, CD244, ITLN1, LOC101928372, ITLN2, F11R, TSTD1, USF1, ARHGAP30, NECTIN4, NECTIN4-AS1, KLHDC9, PFDN2, NIT1, DEDD, LOC112543491, UFC1, USP21, PPOX, B4GALT3, ADAMTS4, NDUFS2, FCER1G, APOA2, TOMM40L, MIR5187, NR1I3, PCP4L1, MPZ, SDHC, CFAP126, FCGR2A, HSPA6, FCGR3A, FCGR2C, HSPA7, FCGR3B, FCGR2B, RPL31P11, FCRLA, FCRLB, DUSP12, ATF6, OLFML2B, NOS1AP, MIR4654, MIR556, SPATA46, C1orf226, SH2D1B, UHMK1, UAP1, DDR2, HSD17B7, CCDC190, RGS4, RGS5, RGS5-AS1, LOC127814295, NUF2, LOC100422212, PBX1, PBX1-AS1, LMX1A, LMX1A-AS1, RXRG, LRRC52-AS1, LRRC52, MGST3, ALDH9A1, LOC440700, TMCO1, TMCO1-AS1, UCK2, MIR3658, FAM78B, FAM78B-AS1, MIR921, LINC01675, FMO9P, POGK, TADA1, ILDR2, MAEL, GPA33, STYXL2, LINC01363, POU2F1, CD247, CREG1, RCSD1, MPZL1, ADCY10, MPC2, DCAF6, MIR1255B2, GPR161, TIPRL, SFT2D2, ANKRD36BP1, TBX19, MIR557, LOC100505918, LOC125312414, LOC101928565, XCL2, XCL1, DPT, LINC00626, LINC00970, LOC101928596, ATP1B1, NME7, BLZF1, CCDC181, SLC19A2, F5, SELP, SELL, SELE, METTL18, FIRRM, SCYL3, KIFAP3, NTMT2, MIR3119-1, MIR3119-2, LINC01681, LINC01142, GORAB-AS1, GORAB, PRRX1, MROH9, FMO3, MIR1295A, MIR1295B, FMO6P, FMO2, FMO1, FMO4, TOP1P1, PRRC2C, MYOCOS, MYOC, VAMP4, METTL13, DNM3, DNM3-IT1, DNM3OS, MIR214, MIR3120, MIR199A2, C1orf105, PIGC, SUCO, FASLG, TNFSF18, TNFSF4, LOC100506023, PRDX6-AS1, PRDX6, SLC9C2, ANKRD45, TEX50, KLHL20, CENPL, DARS2, GAS5-AS1, GAS5, SNORD81, SNORD47, SNORD80, SNORD79, SNORD78, SNORD44, SNORA103, SNORD77, SNORD76, SNORD75, SNORD74, ZBTB37, SERPINC1, RC3H1, RABGAP1L-DT, RABGAP1L, GPR52, RABGAP1L-AS1, CACYBP, MRPS14, TNN, KIAA0040, TNR, LINC01657, COP1, MIR1843, SCARNA3, PAPPA2, ASTN1, MIR488, BRINP2, LINC01645, LINC01741, SEC16B, CRYZL2P-SEC16B, CRYZL2P, RASAL2-AS1, RASAL2, CLEC20A, TEX35, C1orf220, MIR4424, RALGPS2, ANGPTL1, FAM20B, TOR3A, ABL2, SOAT1, AXDND1, NPHS2, TDRD5, FAM163A, LOC128071543, MIR12116, TOR1AIP2, TOR1AIP1, CEP350, QSOX1, LHX4, LHX4-AS1, ACBD6, MIR3121, OVAAL, XPR1, KIAA1614, KIAA1614-AS1, STX6, MR1, IER5, LINC01732, LINC01699, CACNA1E, ZNF648, LINC01344, GLUL, TEDDM1, LINC00272, RGSL1, RNASEL, RGS16, LINC01686, RGS8, LINC01688, NPL, DHX9-AS1, DHX9, SHCBP1L, LAMC1, LAMC1-AS1, LAMC2, NMNAT2, SMG7-AS1, SMG7, NCF2, ARPC5, RGL1, APOBEC4, COLGALT2, TSEN15, C1orf21-DT, C1orf21, EDEM3, NIBAN1, LINC01633, RNF2, TRMT1L, SWT1, IVNS1ABP, GS1-279B7.1, LINC01350, HMCN1, PRG4, TPR, ODR4, PDC-AS1, PDC, PTGS2, PACERR, PLA2G4A, LINC01036, LINC01037, LINC01035, BRINP3, BRINP3-DT, LINC01720, LINC01680, RGS18, RGS21, RGS1, RGS13, MIR4426, RGS2, RGS2-AS1, UCHL5, SCARNA18B, RO60, GLRX2, CDC73, MIR1278, B3GALT2, LINC01031, LINC01724, KCNT2, MIR4735, CFH, CFHR3, CFHR1, CFHR4, CFHR2, CFHR5, F13B, ASPM, ZBTB41, CRB1, DENND1B, C1orf53, LHX9, NEK7, ATP6V1G3, PTPRC, MIR181A1HG, MIR181B1, MIR181A1, LINC01222, LINC01221, LINC02789, NR5A2, LINC00862, ZNF281, KIF14, DDX59, DDX59-AS1, CAMSAP2, GPR25, INAVA, MROH3P, KIF21B, CACNA1S, ASCL5, TMEM9, IGFN1, PKP1, TNNT2, LAD1, TNNI1, PHLDA3, CSRP1, CSRP1-AS1, RPS10P7, NAV1, IPO9-AS1, MIR5191, MIR1231, IPO9, MIR6739, SHISA4, LMOD1, TIMM17A, SNORA70H, RNPEP, ELF3-AS1, MIR6740, ELF3, GPR37L1, ARL8A, PTPN7, PTPRVP, LGR6, UBE2T, PPP1R12B, SYT2, KDM5B, PCAT6, MGAT4EP, ACTG1P25, RABIF, KLHL12, ADIPOR1, CYB5R1, MGAT4FP, TMEM183A, PPFIA4, MYOG, MYOPARR, ADORA1, MYBPH, CHI3L1, CHIT1, LINC01353, BTG2-DT, BTG2, FMOD, PRELP, OPTC, ATP2B4, SNORA77, LINC00260, LAX1, ZBED6, ZC3H11A, LOC128031836, SNRPE, LINC00303, SOX13, ETNK2, ERLNC1, REN, KISS1, GOLT1A, PLEKHA6, LINC00628, PPP1R15B, PIK3C2B, MDM4, LRRN2, NFASC, CNTN2, TMEM81, RBBP5, DSTYK, TMCC2, TMCC2-AS1, NUAK2, KLHDC8A, LEMD1-AS1, LEMD1, BLACAT1, MIR135B, LEMD1-DT, CDK18, MFSD4A-AS1, MFSD4A, ELK4, SLC45A3, NUCKS1, RAB29, SLC41A1, PM20D1, PM20D1-AS1, SLC26A9, SLC26A9-AS1, LOC103021295, RAB7B, CTSE, RHEX, AVPR1B, FAM72A, SRGAP2, IKBKE, MIR6769B, IKBKE-AS1, RASSF5, EIF2D, DYRK3-AS1, DYRK3, MAPKAPK2, IL10, IL19, IL20, IL24, FCMR, PIGR, FCAMR, C1orf116, YOD1, PFKFB2, C4BPB, C4BPA, CD55, CR2, CR1, CR1L, CD46, MIR29B2CHG, MIR29C, MIR29B2, LOC148696, CD34, LINC02767, PLXNA2, LINC01717, LINC01774, LINC01696, LINC01698, MIR205HG, MIR205, CAMK1G, LAMB3, MIR4260, HSD11B1-AS1, G0S2, HSD11B1, TRAF3IP3, C1orf74, IRF6, UTP25, SYT14, SERTAD4-AS1, SERTAD4, LINC02602, HHAT, KCNH1, KCNH1-IT1, RCOR3, TRAF5, LINC00467, RD3, SLC30A1, NTRAS, NEK2, NEK2-DT, LPGAT1, LPGAT1-AS1, INTS7, DTL, RPL21P28, MIR3122, LINC02608, PPP2R5A, SNORA16B, PACC1, NENF, LINC02771, LINC01740, ATF3, GARIN4, LINC02773, BATF3, NSL1, TATDN3, SPATA45, FLVCR1-DT, FLVCR1, VASH2, ANGEL2, RPS6KC1, PROX1-AS1, LINC00538, PROX1, SMYD2, PTPN14, CENPF, KCNK2, KCTD3, USH2A, USH2A-AS2, ESRRG, GPATCH2, SPATA17, SPATA17-AS1, LINC00210, LINC01653, RRP15, TGFB2-AS1, TGFB2, TGFB2-OT1, LINC02869, LINC01710, LYPLAL1-DT, LYPLAL1, LYPLAL1-AS1, ZC3H11B, SLC30A10, EPRS1, BPNT1, IARS2, MIR215, MIR194-1, RAB3GAP2, MIR664A, SNORA36B, AURKAP1, MARK1, C1orf115, MTARC2, MTARC1, RNU6ATAC35P, LINC01352, HLX-AS1, HLX, LINC02817, DUSP10, LINC01655, LINC02257, LINC02474, HHIPL2, TAF1A, TAF1A-AS1, MIA3, AIDA, BROX, FAM177B, DISP1, TLR5, SUSD4, CCDC185, CAPN8, CAPN2, TP53BP2, GTF2IP20, SEPTIN7P13, FBXO28, DEGS1, LOC101927143, LOC101927164, NVL, MIR320B2, CNIH4, WDR26, MIR4742, CNIH3, CNIH3-AS2, DNAH14, LBR, ENAH, SRP9, EPHX1, TMEM63A, LEFTY1, PYCR2, MIR6741, LEFTY2, SDE2, H3-3A, LINC01703, ACBD3, ACBD3-AS1, MIXL1, LIN9, PARP1, STUM, ITPKB, ITPKB-IT1, PSEN2, COQ8A, CDC42BPA, ZNF678, ZNF847P, SNAP47, JMJD4, LOC105373289, PRSS38, WNT9A, MIR5008, LOC107985355, WNT3A, LINC02809, ARF1, MIR3620, C1orf35, MRPL55, GUK1, GJC2, IBA57-DT, IBA57, OBSCN-AS1, OBSCN, TRIM11, MIR6742, TRIM17, H3-4, H2AC25, H2BC26, MIR4666A, RNF187, BTNL10P, RNA5S1, RNA5S2, RNA5S3, RNA5S4, RNA5S5, RNA5S6, RNA5S7, RNA5S8, RNA5S9, RNA5S10, RNA5S11, RNA5S12, RNA5S13, RNA5S14, RNA5S15, RNA5S16, RHOU, DUSP5P1, RNA5S17, TMEM78, RAB4A-AS1, RAB4A, CCSAP, ACTA1, NUP133, NUP133-DT, ABCB10, TAF5L, URB2, LINC01682, LINC01736, GALNT2, PGBD5, LINC01737, COG2, AGT, CAPN9, C1orf198, LOC101927604, TTC13, ARV1, FAM89A, MIR1182, TRIM67, TRIM67-AS1, C1orf131, GNPAT, EXOC8, SPRTN, EGLN1, SNRPD2P2, LOC122526782, TSNAX-DISC1, TSNAX, LINC00582, DISC1, DISC2, DISC1-IT1, SIPA1L2, LINC01745, LINC01744, MAP10, NTPCR, PCNX2, MAP3K21, KCNK1, MIR4427, SLC35F3, SLC35F3-AS1, MIR4671, COA6-AS1, COA6, TARBP1, LINC01354, IRF2BP2, LINC00184, LOC101927787, LINC01132, LINC02961, LNCATV, LINC01348, TOMM20, SNORA14B, RBM34, ARID4B, MIR4753, GGPS1, TBCE, B3GALNT2, GNG4, LYST, MIR1537, LINC02768, NID1, GPR137B, ERO1B, EDARADD, LGALS8, LGALS8-AS1, HEATR1, ACTN2, MTR, MT1HL1, RYR2, MIR4428, LOC100130331, ZP4, LINC01139, CHRM3, CHRM3-AS2, CHRM3-AS1, RPS7P5, FMN2, GREM2, RGS7, MIR3123, FH, KMO, OPN3, CHML, WDR64, EXO1, BECN2, MAP1LC3C, PLD5, LINC01347, CEP170, SDCCAG8, MIR4677, AKT3, AKT3-IT1, LINC02774, ZBTB18, C1orf100, ADSS2, CATSPERE, DESI2, C1orf202, COX20, HNRNPU, SNORA100, LOC101928068, EFCAB2, KIF26B, KIF26B-AS1, SMYD3, SMYD3-AS1, LINC01743, TFB2M, CNST, SCCPDH, LINC01341, AHCTF1, ZNF695, ZNF670-ZNF695, ZNF670, ZNF669, FLJ39095, LINC02897, ZNF124, MIR3916, VN1R5, ZNF496, ZNF496-DT, NLRP3, OR2B11, OR2W5P, GCSAML, OR2C3, GCSAML-AS1, OR2G2, OR2G3, OR14L1, OR13G1, OR6F1, OR14A2, OR14K1, OR1C1, OR9H1P, OR14A16, OR11L1, TRIM58, OR2W3, OR2T8, OR2AJ1, OR2L13, OR2L8, OR2AK2, OR2L1P, OR2L5, OR2L2, OR2L3, OR2M1P, OR2M5, OR2M2, OR2M3, OR2M4, OR2T33, OR2T12, OR2M7, OR14C36, OR2T4, OR2T6, OR2T1, OR2T7, OR2T2, OR2T3, OR2T5, OR2G6, OR2T29, OR2T34, OR2T10, OR2T11, OR2T35, OR2T27, OR14I1, LYPD9P, LYPD8, SH3BP5L, MIR3124 | arr[GRCh38] 1q21.1q44(145,196,802_248,827,731)x4 |
| 2  Brain | Gain | 2 | p25.3 | p16.3 | 50,908 | FAM110C, SH3YL1, ACP1, ALKAL2, LINC01865, LINC01874, LOC100996637, LINC01875, LOC105373352, TMEM18, TMEM18-DT, LINC01115, LINC01939, SNTG2-AS1, SNTG2, TPO, LOC102723730, PXDN, MYT1L, MYT1L-AS1, LINC01250, EIPR1, TRAPPC12, TRAPPC12-AS1, ADI1, RNASEH1, RNASEH1-DT, RPS7, COLEC11, ALLC, DCDC2C, LINC01304, LOC105373394, LINC01249, LINC01248, SOX11, LINC01810, SILC1, MIR7158, LOC400940, LINC01247, LINC01246, MIR7515HG, MIR7515, LINC00487, NRIR, CMPK2, RSAD2, GRASLND, RNF144A, LOC101929452, LOC100506274, LINC01871, LOC101929551, LINC00298, LINC00299, LINC01814, ID2-AS1, ID2, KIDINS220, MBOAT2, ASAP2, ITGB1BP1, CPSF3, IAH1, ADAM17, YWHAQ, TAF1B, GRHL1, KLF11-DT, KLF11, CYS1, RRM2, MIR4261, HPCAL1, ODC1, SNORA80B, ODC1-DT, NOL10, RN7SL832P, ATP6V1C2, PDIA6, LINC01954, KCNF1, FLJ33534, C2orf50, SLC66A3, ROCK2, LINC00570, E2F6, GREB1, MIR4429, NTSR2, LPIN1, MIR548S, MIR4262, MIR3681HG, MIR3681, TRIB2, MIR3125, LOC100506474, LINC00276, LRATD1, NBAS, DDX1, LINC01804, MYCNUT, MYCNOS, MYCN, GACAT3, CYRIA, RAD51AP2, VSNL1, SMC6, GEN1, MSGN1, KCNS3, RDH14, NT5C1B-RDH14, NT5C1B, LINC01376, MIR4757, OSR1, LINC01808, LINC00954, TTC32, WDR35, WDR35-DT, MATN3, LAPTM4A, SDC1, PUM2, RHOB, LOC107985856, HS1BP3-IT1, HS1BP3, GDF7, LDAH, APOB, TDRD15, LINC01822, LINC01884, LOC107985792, KLHL29, ATAD2B, UBXN2A, MFSD2B, WDCP, FKBP1B, SF3B6, FAM228B, TP53I3, PFN4, FAM228A, ITSN2, NCOA1, PTRHD1, CENPO, ADCY3, DNAJC27, DNAJC27-AS1, EFR3B, POMC, LINC01381, DNMT3A, MIR1301, DTNB, DTNB-AS1, ASXL2, KIF3C, RAB10, GAREM2, HADHA, HADHB, ADGRF3, SELENOI, DRC1, OTOF, FAM166C, CIB4, KCNK3, SLC35F6, CENPA, DPYSL5, MAPRE3, MAPRE3-AS1, TMEM214, AGBL5-AS1, AGBL5, OST4, EMILIN1, KHK, CGREF1, ABHD1, PREB, PRR30, TCF23, SLC5A6, ATRAID, CAD, SLC30A3, DNAJC5G, TRIM54, UCN, MPV17, GTF3C2, GTF3C2-AS1, GTF3C2-AS2, EIF2B4, SNX17, ZNF513, PPM1G, FTH1P3, NRBP1, KRTCAP3, IFT172, FNDC4, GCKR, C2orf16, ZNF512, CCDC121, GPN1, SUPT7L, SLC4A1AP, LINC01460, LOC105374378, MRPL33, RBKS, BABAM2, BABAM2-AS1, MIR4263, LOC100505736, LOC100505716, FOSL2-AS1, FOSL2, PLB1, LOC100505774, PPP1CB, SPDYA, TRMT61B, WDR43, SNORD92, SNORD53, SNORD53B, TOGARAM2, PCARE, CLIP4, ALK, LOC105374389, YPEL5, SNORA10B, LBH, LINC01936, LCLAT1, CAPN13, GALNT14, CAPN14, EHD3, XDH, SRD5A2, LINC01946, MEMO1, DPY30, SPAST, SLC30A6, NLRC4, YIPF4, BIRC6, MIR558, BIRC6-AS2, TTC27, MIR4765, LINC00486, LOC100271832, LTBP1, MIR4430, RASGRP3, RASGRP3-AS1, FAM98A, LINC01317, MYADML, LINC01318, LINC01320, MIR548AD, CRIM1-DT, CRIM1, FEZ2, VIT, STRN, HEATR5B, GPATCH11, EIF2AK2, SULT6B1, CEBPZOS, CEBPZ, NDUFAF7, PRKD3, PRKD3-DT, QPCT, CDC42EP3, PIRAT1, RMDN2, RMDN2-AS1, CYP1B1, CYP1B1-AS1, ATL2, LINC01883, LINC02613, HNRNPLL, GALM, SRSF7, GEMIN6, DHX57, MORN2, ARHGEF33, LOC375196, SOS1, CDKL4, MAP4K3, MAP4K3-DT, TMEM178A, THUMPD2, SLC8A1-AS1, SLC8A1, LINC01794, LINC01913, LINC01914, LINC02898, PKDCC, EML4-AS1, EML4, COX7A2L, KCNG3, MTA3, OXER1, HAAO, LINC01819, LINC02580, ZFP36L2, LINC01126, THADA, PLEKHH2, C1GALT1C1L, DYNC2LI1, ABCG5, ABCG8, LRPPRC, PPM1B, SLC3A1, PREPL, CAMKMT, LINC01833, SIX3-AS1, SIX3, SIX2, LINC01121, SRBD1, PRKCE, EPAS1, LINC01820, LINC02583, TMEM247, ATP6V1E2, RHOQ, RHOQ-AS1, PIGF, CRIPT, SOCS5, LINC01118, LINC01119, MCFD2, TTC7A, STPG4, CALM2, EPCAM-DT, BCYRN1, EPCAM, MIR559, MSH2, KCNK12, MSH2-OT1, MSH6, FBXO11, FOXN2, PPP1R21, STON1-GTF2A1L, STON1, GTF2A1L, LHCGR, FSHR, MIR548BA, NRXN1, MIR8485 | arr[GRCh38] 2p25.3p16.3(12,771_50,920,883)x3~4 |
| 2  Brain | Gain | 2 | p16.3 | p16.1 | 4,500 | LOC730100, LINC01867, MIR4431, ASB3, GPR75-ASB3, CHAC2, ERLEC1, MIR3682, GPR75, PSME4, ACYP2, TSPYL6, C2orf73, SPTBN1, RPL23AP32, EML6, RTN4, CLHC1, RPS27A, MTIF2, PRORSD1P, CCDC88A, CFAP36, PPP4R3B, PNPT1, EFEMP1, MIR217HG, MIR217, MIR216A, MIR216B, LINC01813 | arr[GRCh38] 2p16.3p16.1(51,660,303_56,160,013)x3 |
| 2  Brain | Gain | 2 | p16.1 | p11.2 | 32,551 | CCDC85A, VRK2, LOC107984043, FANCL, LINC01795, LINC01122, LINC01793, MIR4432HG, MIR4432, BCL11A, PAPOLG, REL-DT, REL, PUS10, PEX13, SANBR, C2orf74-DT, C2orf74, C2orf74-AS1, AHSA2P, USP34, SNORA70B, XPO1, FAM161A, CCT4, COMMD1, B3GNT2, MIR5192, TMEM17, EHBP1, EHBP1-AS1, OTX1, DBIL5P2, WDPCP, MDH1, UGP2, VPS54, PELI1, LINC00309, LOC100507006, MIR4433B, MIR4433A, LGALSL, LINC01805, AFTPH-DT, AFTPH, MIR4434, LINC02579, SERTAD2, LINC01800, LINC02245, SLC1A4, LINC02576, CEP68, RAB1A, ACTR2, SPRED2, LINC03050, MIR4778, LINC01873, MEIS1-AS3, MEIS1, MEIS1-AS2, LINC01798, LINC01797, LINC01799, LINC01628, LINC01828, LINC01829, LOC101927661, ETAA1, LINC02831, LINC01812, C1D, DNAAF10, PNO1, PPP3R1, CNRIP1, PLEK, FBXO48, APLF, PROKR1, ARHGAP25, LINC01890, LINC01888, BMP10, GKN2, GKN1, ANTXR1, MIR3126, GFPT1, NFU1, AAK1, SNORA36C, ANXA4, SMANTIS, GMCL1, SNRNP27, MXD1, ASPRV1, PCBP1-AS1, PCBP1, LINC01816, C2orf42, TIA1, MIR1285-2, PCYOX1, SNRPG, FAM136A, TGFA, TGFA-IT1, ADD2, FIGLA, CLEC4F, CD207, LINC01143, VAX2, ATP6V1B1, ATP6V1B1-AS1, ANKRD53, TEX261, OR7E91P, NAGK, MCEE, MPHOSPH10, PAIP2B, ZNF638, DYSF, CYP26B1, EXOC6B, SPR, EMX1, SFXN5, RAB11FIP5, NOTO, SMYD5, PRADC1, CCT7, FBXO41, EGR4, ALMS1, ALMS1-IT1, NAT8, ALMS1P1, NAT8B, TPRKB, DUSP11, C2orf78, STAMBP, ACTG2, DGUOK, DGUOK-AS1, TET3, BOLA3, BOLA3-DT, MOB1A, MTHFD2, SLC4A5, DCTN1, DCTN1-AS1, C2orf81, WDR54, RTKN, INO80B-WBP1, INO80B, WBP1, MOGS, MRPL53, CCDC142, TTC31, LBX2, LBX2-AS1, PCGF1, TLX2, DQX1, AUP1, HTRA2, LOXL3, DOK1, M1AP, SEMA4F, HK2, LINC01291, POLE4, TACR1, MIR5000, LOC105374811, EVA1A, EVA1A-AS, MRPL19, GCFC2, LRRTM4, LRRTM4-AS1, LOC101927967, LINC01851, SNAR-H, LOC101927948, LOC105374820, REG3G, REG1B, REG1A, REG1CP, REG3A, CTNNA2, CTNNA2-AS1, MIR4264, MIR8080, LRRTM1, LINC01815, DHFRP3, LINC01809, FUNDC2P2, SUCLG1, DNAH6, TRABD2A, TMSB10, KCMF1, LINC01964, TCF7L1, TCF7L1-IT1, LOC102724579, TGOLN2, RETSAT, ELMOD3, CAPG, SH2D6, PARTICL, MAT2A, GGCX, VAMP8, VAMP5, RNF181, TMEM150A, USP39, C2orf68, SFTPB, GNLY, ATOH8, MIR6071, LOC284950, ST3GAL5, ST3GAL5-AS1, POLR1A, PTCD3, SNORD94, IMMT, MIR4779, MRPL35, REEP1, KDM3A, CHMP3, RNF103-CHMP3, CHMP3-AS1, RNF103, RMND5A, CD8A, CD8B, ANAPC1P1, RGPD1, PLGLB1, ANAPC1P2, LOC102724642, MIR4771-1, CYTOR, MIR4435-1, ANAPC1P4, PLGLB2, RGPD2, KRCC1, SMYD1, MIR4780, FABP1, THNSL2, FOXI3, TEX37, LOC101928371, EIF2AK3, EIF2AK3-DT, RPIA, ANKRD36BP2, MIR4436A | arr[GRCh38] 2p16.1p11.2(56,278,044_88,828,593)x3~4 |
| 2  Brain | Gain | 2 | q11.2 | q12.1 | 5,305 | ANKRD36B, COX5B, ACTR1B, C2orf92, ZAP70, TMEM131, VWA3B, CNGA3, INPP4A, COA5, UNC50, MGAT4A, LINC02611, CRACDL, TSGA10, C2orf15, LIPT1, MITD1, MRPL30, LYG2, LYG1, TXNDC9, EIF5B, REV1, AFF3, LINC01104, LONRF2, CHST10, NMS, PDCL3, LINC01849, NPAS2, NPAS2-AS1, RPL31, TBC1D8, TBC1D8-AS1, CNOT11, SNORD89, RNF149, MIR5696, CREG2, RFX8, MAP4K4, LINC01127, IL1R2, IL1R1, IL1R1-AS1, IL1RL2, IL1RL1, IL18R1, IL18RAP, MIR4772, SLC9A4, SLC9A2, MFSD9, TMEM182 | arr[GRCh38] 2q11.2q12.1(97,521,942_102,826,864)x3~4 |
| 2  Brain | Gain | 2 | q12.1 | q14.1 | 9,838 | LINC01114, PANTR1, POU3F3, LINC01159, MRPS9-AS2, MRPS9, MRPS9-AS1, LINC01918, GPR45, TGFBRAP1, C2orf49, FHL2, LINC02946, NCK2, ECRG4, UXS1, ANAPC1P6, PLGLA, RGPD3, LOC107985931, CD8B2, ST6GAL2, LINC01789, LINC01885, LINC01886, GACAT1, RGPD4-AS1, RGPD4, SLC5A7, LINC01593, LINC01594, SULT1C3, SULT1C2, SULT1C5P, SULT1C4, GCC2, GCC2-AS1, LIMS1, LIMS1-AS1, RANBP2, CCDC138, EDAR, SH3RF3-AS1, SH3RF3, MIR4265, MIR4266, SEPTIN10, SOWAHC, RGPD5, LIMS3-LOC440895, LIMS3, LOC440895, LINC01123, MIR4267, MALL, MIR4436B1, NPHP1, MTLN, LOC100507334, MIR4436B2, LINC01106, LOC100288570, LIMS4, RGPD6, LOC105373553, BUB1, SNORD132, ACOXL, ACOXL-AS1, BCL2L11, MIR4435-2HG, MIR4435-2, ANAPC1, MIR4771-2, MERTK, TMEM87B, FBLN7, ZC3H8, ZC3H6, RGPD8, TTL, LOC105373562, POLR1B, CHCHD5, SLC20A1-DT, SLC20A1, NT5DC4, CKAP2L, IL1A, IL1B, IL37, IL36G, IL36A, IL36B, IL36RN, IL1F10, IL1RN, PSD4, PAX8, PAX8-AS1, LINC02966, ZNG1B, FOXD4L1, PGM5P4-AS1, PGM5P4, FAM138B, MIR1302-3, WASH2P, DDX11L2, RPL23AP7, RABL2A, SLC35F5, MIR4782, ACTR3-AS1, ACTR3, LINC01191, LINC02992, DPP10 | arr[GRCh38] 2q12.1q14.1(104,647,867_114,486,029)x3~4 |
| 2  Brain | Gain | 2 | q14.2 | q14.2 | 3,294 | THORLNC, LOC101927709, EN1, MARCO, C1QL2, STEAP3, STEAP3-AS1, C2orf76, DBI, TMEM37, SCTR, SCTR-AS1, CFAP221, TMEM177, PTPN4, EPB41L5, TMEM185B, RALB, INHBB, LINC01101, GLI2, TFCP2L1, CLASP1 | arr[GRCh38] 2q14.2(118,134,420_121,428,593)x3 |
| 2  Brain | Gain | 2 | q14.2 | q32.2 | 67,889 | CLASP1, RNU4ATAC, NIFK-AS1, NIFK, TSN, LINC01823, LINC01826, CNTNAP5-DT, CNTNAP5, LINC01941, GYPC, TEX51, BIN1, CYP27C1, ERCC3, MAP3K2, PROC, MIR4783, IWS1, MYO7B, LOC105373609, LIMS2, GPR17, WDR33, SFT2D3, POLR2D, AMMECR1L, SAP130, UGGT1, HS6ST1, LOC101927881, LINC01854, LINC02572, PLAC9P1, LINC01856, RAB6C-AS1, RAB6C, FAR2P1, POTEF, MED15P9, CCDC74B, SMPD4, MZT2B, TUBA3E, CCDC115, IMP4, PTPN18, FAR2P2, CYP4F62P, POTEI, CFC1B, PRSS40B, PRSS40A, CFC1, POTEJ, CYP4F30P, GPR148, AMER3, ARHGEF4, SMIM39, FAM168B, PLEKHB2, POTEE, LOC440910, RAB6D, LINC01120, NOC2LP2, TUBA3D, MZT2A, MIR4784, SMPD4BP, CCDC74A, POTEKP, LINC01087, C2orf27A, CDRT15P3, LINC01945, ANKRD30BL, MIR663B, ZNF285CP, FAM201B, GPR39, MIR9986, LYPD1, NCKAP5, NCKAP5-AS1, NCKAP5-AS2, MGAT5, MIR3679, TMEM163, ACMSD, MIR5590, CCNT2-AS1, CCNT2, MAP3K19, RAB3GAP1, SNORA40B, ZRANB3, R3HDM1, MIR128-1, LOC107985946, UBXN4, LCT, LCT-AS1, MCM6, DARS1, DARS1-AS1, CXCR4, THSD7B, LOC101928273, HNMT, LINC01832, SPOPL, LINC02631, NXPH2, YY1P2, LINC01853, LRP1B, MIR7157, KYNU, ARHGAP15, LOC101928386, GTDC1, ZEB2, ZEB2-AS1, LINC01412, LINC02993, TEX41, PABPC1P2, ACVR2A, ORC4, MBD5, EPC2, KIF5C, LYPD6B, LYPD6, MIR9899, MMADHC, MMADHC-DT, LINC01931, LINC01817, RND3, LINC01920, LINC02612, RBM43, NMI, LOC101929319, TNFAIP6, MIR4773-1, MIR4773-2, RIF1, NEB, ARL5A, CACNB4, STAM2, FMNL2, PRPF40A, ARL6IP6, RPRM, GALNT13, GALNT13-AS1, KCNJ3, LINC01876, NR4A2, FLJ46875, GPD2, GALNT5, ERMN, FAM133DP, CYTIP, ACVR1C, ACVR1, UPP2, CCDC148-AS1, CCDC148, PKP4, PKP4-AS1, DAPL1, TANC1, MIR6888, WDSUB1, BAZ2B, BAZ2B-AS1, MARCHF7, CD302, LY75-CD302, LY75, PLA2R1, ITGB6, LINC02478, RBMS1, MIR4785, TANK, PSMD14-DT, LINC01806, PSMD14, TBR1, AHCTF1P1, SLC4A10, DPP4, DPP4-DT, LOC101929532, GCG, FAP, IFIH1, GCA, KCNH7, KCNH7-AS1, FIGN, GRB14, COBLL1, SNORA70F, LOC101929633, SLC38A11, SCN3A, SCN2A, CSRNP3, GALNT3, LOC100506124, TTC21B, TTC21B-AS1, LOC102724058, SCN1A, SCN1A-AS1, SCN9A, SCN7A, XIRP2, XIRP2-AS1, B3GALT1, B3GALT1-AS1, STK39, CERS6, MIR4774, CERS6-AS1, NOSTRIN, SPC25, G6PC2, ABCB11, DHRS9, LRP2, BBS5, KLHL41, FASTKD1, PPIG, CFAP210, PHOSPHO2, PHOSPHO2-KLHL23, KLHL23, SSB, METTL5, SNORD3K, UBR3, MYO3B, MYO3B-AS1, ERICH2-DT, LINC01124, SP5, ERICH2, GAD1, GORASP2, TLK1, METTL8, DCAF17, CYBRD1, DYNC1I2, SLC25A12, HAT1, METAP1D, DLX1, DLX2, DLX2-DT, ITGA6, ITGA6-AS1, PDK1, RAPGEF4-AS1, RAPGEF4, MAP3K20, MAP3K20-AS1, CDCA7, SP3, LINC01960, OLA1, LINC01305, SP9, CIR1, SCRN3, GPR155, GPR155-DT, WIPF1, H3P6, CHRNA1, CHN1, ATF2, MIR933, ATP5MC3, LNPK, EVX2, HOXD13, HOXD12, HOXD11, HOXD10, HOXD9, HOXD8, HOXD-AS2, LOC401021, MIR10B, HOXD4, HOXD3, HAGLR, HAGLROS, HOXD1, MIR7704, MTX2, MIR1246, LINC01116, LINC01117, LOC105373759, MIR4444-1, HNRNPA3, NFE2L2, MIR3128, LOC100130691, MIR6512, AGPS, IFT70B, IFT70A, PDE11A, PDE11A-AS1, RBM45, OSBPL6, CHROMR, PRKRA, PJVK, FKBP7, PLEKHA3, TTN-AS1, TTN, LOC101927055, CCDC141, SESTD1, ZNF385B, MIR1258, CWC22, SCHLAP1, UBE2E3, LINC01934, MIR4437, ITGA4, CERKL, NEUROD1, ITPRID2, PPP1R1C, PDE1A, DNAJC10, FRZB, NCKAP1, DUSP19, NUP35, MIR548AE1, LOC105373780, ZNF804A, LOC105373782, FSIP2-AS2, FSIP2, FSIP2-AS1, LINC01473, ZC3H15, ITGAV, FAM171B, ZSWIM2, CALCRL, TFPI, LINC01090, GULP1, MIR561, DIRC1, COL3A1, MIR1245A, MIR1245B, MIR3606, COL5A2, MIR3129 | arr[GRCh38] 2q14.2q32.2(121,455,098_189,343,653)x3~4 |
| 2  Brain | Gain | 2 | q32.2 | q32.3 | 3,272 | C2orf88, HIBCH, INPP1, MFSD6, NEMP2, NEMP2-DT, NAB1, GLS, STAT1, STAT4-AS1, STAT4, MYO1B, NABP1, LOC729254, CAVIN2, TMEFF2, PCGEM1 | arr[GRCh38] 2q32.2q32.3(190,082,313_193,353,955)x2~3 |
| 2  Brain | Gain | 2 | q33.1 | q37.3 | 42,274 | LINC01877, FTCDNL1, C2orf69, TYW5, MAIP1, SPATS2L, KCTD18, SGO2, AOX1, AOX3P-AOX2P, LINC01792, BZW1-AS1, BZW1, CLK1, PPIL3, NIF3L1, ORC2, HYCC2, NDUFB3, CFLAR, CFLAR-AS1, CASP10, CASP8, FLACC1, TRAK2, STRADB, C2CD6, TMEM237, MPP4, ALS2, CDK15, FZD7, KIAA2012, KIAA2012-AS1, SUMO1, NOP58, SNORD70, SNORD70B, SNORD11B, SNORD11, BMPR2, FAM117B, ICA1L, WDR12, CARF, NBEAL1, CYP20A1, ABI2, RAPH1, CD28, CTLA4, ICOS, PARD3B, NRP2, INO80D, NDUFS1, GCSHP3, EEF1B2, SNORD51, SNORA41, CMKLR2, CMKLR2-AS, ZDBF2, ADAM23, FAM237A, DYTN, MDH1B, FASTKD2, MIR3130-1, MIR3130-2, CPO, KLF7, MIR2355, MIR7845, MYOSLID-AS1, MYOSLID, LINC01802, MIR1302-4, CREB1, METTL21A, LINC01857, CCNYL1, MIR4775, FZD5, PLEKHM3, LOC100507443, CRYGD, CRYGC, CRYGB, CRYGA, C2orf80, IDH1, IDH1-AS1, PIKFYVE, PTH2R, LOC101927960, MAP2, UNC80, RPE, KANSL1L, KANSL1L-AS1, ACADL, MYL1, LANCL1-AS1, LANCL1, CPS1, CPS1-IT1, ERBB4, MIR548F2, LINC01878, MIR4776-1, MIR4776-2, IKZF2, LINC01953, SPAG16-DT, SPAG16, MIR4438, VWC2L, VWC2L-IT1, BARD1, SNHG31, SNORA70I, ABCA12, LINC02862, ATIC, FN1, LOC102724849, LINC00607, LINC01614, MREG, PECR, TMEM169, XRCC5, LINC01963, MARCHF4, SMARCAL1, RPL37A-DT, RPL37A, LINC01280, IGFBP2, IGFBP5, TNP1, LOC105373876, LINC01921, DIRC3-AS1, DIRC3, TNS1, SNORA115, MIR6809, TNS1-AS1, RUFY4, CXCR2P1, CXCR2, CXCR1, ARPC2, GPBAR1, AAMP, PNKD, TMBIM1, MIR6513, CATIP-AS2, MIR6810, CATIP, CATIP-AS1, SLC11A1, CTDSP1, MIR26B, VIL1, USP37, CNOT9, PLCD4, ZNF142, BCS1L, RNF25, STK36, TTLL4, CYP27A1, PRKAG3, MIR9500, WNT6, WNT10A, LINC01494, CDK5R2, LINC00608, FEV, CRYBA2, MIR375, LOC100129175, CFAP65, IHH, MIR3131, NHEJ1, SLC23A3, CNPPD1, RETREG2, ZFAND2B, ABCB6, ATG9A, ANKZF1, GLB1L, STK16, TUBA4A, TUBA4B, DNAJB2, PTPRN, MIR153-1, RESP18, DNPEP, DNPEP-AS1, DES, SPEG, SPEGNB, GMPPA, ASIC4, CHPF, TMEM198, MIR3132, OBSL1, INHA, STK11IP, SLC4A3, LINC01803, MIR4268, EPHA4, PAX3, CCDC140, LOC122319436, CT75, SGPP2, FARSB, MOGAT1, ACSL3, KCNE4, SCG2, AP1S3, WDFY1, MRPL44, SERPINE2, FAM124B, CUL3, CCDC195, DOCK10, MIR4439, NYAP2, LOC646736, MIR5702, IRS1, RHBDD1, COL4A4, COL4A3, MFF-DT, MFF, TM4SF20, SCYGR1, MIR5703, AGFG1, SCYGR2, SCYGR10, C2orf83, SCYGR3, LOC729968, SCYGR4, SCYGR5, SLC19A3, SCYGR6, SCYGR7, SCYGR8, CCL20, DAW1, SPHKAP, LINC01807, PID1, DNER, TRIP12, FBXO36, SLC16A14, SP110, SP140, SP140L, SP100, LINC01907, CAB39, ITM2C, GCSIR, GPR55, SPATA3-AS1, SPATA3, C2orf72, PSMD1, HTR2B, ARMC9, MIR4777, B3GNT7, NCL, SNORA75, SNORD20, SNORD82, LINC00471, NMUR1, TEX44, PTMA, MIR1244-1, PDE6D, COPS7B, MIR1471, NPPC, DIS3L2, MIR562, ALPP, ECEL1P2, ALPG, ALPI, ECEL1, PRSS56, CHRND, CHRNG, TIGD1, MIR5001, EIF4E2, EFHD1, GIGYF2, KCNJ13, SNORC, NGEF, LOC101928881, NEU2, INPP5D, ATG16L1, SCARNA5, SCARNA6, SAG, DGKD, USP40, UGT1A8, UGT1A10, UGT1A9, UGT1A7, UGT1A6, UGT1A5, UGT1A4, UGT1A3, DNAJB3, LOC100286922, UGT1A1, MROH2A, HJURP, MSL3P1, TRPM8, SPP2, LINC01891, ARL4C, LINC01173, SH3BP4, AGAP1, AGAP1-IT1, TNRC17, GBX2, ASB18, IQCA1, IQCA1-AS1, ACKR3, LOC93463, COPS8, COL6A3, MLPH, MIR6811, PRLH, RAB17, LRRFIP1, RBM44, RAMP1, UBE2F-SCLY, UBE2F, SCLY, ESPNL, KLHL30, ERFE, ILKAP, LINC02610, TARDBPP3, HES6, PER2, TRAF3IP1, ASB1, LINC01107, LOC100287387, TWIST2, LINC01940, HDAC4, MIR4440, MIR4441, MGC16025, MIR4269, MIR2467, HDAC4-AS1, LINC02991, LOC150935, NDUFA10, MIR4786, OR6B2, OR6B3, COPS9, OTOS, GPC1, GPC1-AS1, MIR149, ANKMY1, DUSP28, RNPEPL1, CAPN10-DT, CAPN10, GPR35, AQP12B, LOC285191, AQP12A, KIF1A, AGXT, MAB21L4, CROCC2, UICLM, SNED1, MTERF4, PASK, PPP1R7, ANO7, HDLBP, HDLBP-AS1, SEPTIN2, FARP2, MIR3133, STK25, BOK-AS1, BOK, THAP4, ATG4B, DTYMK, ING5, D2HGDH, GAL3ST2, NEU4 | arr[GRCh38] 2q33.1q37.3(199,567,661_241,841,232)x3~4 |
| 2  Brain | Gain | 3 | p26.3 | q11.2 | 98,199 | LINC01986, CHL1-AS2, CHL1, CHL1-AS1, LINC01266, CNTN6, CNTN4, CNTN4-AS2, CNTN4-AS1, IL5RA, TRNT1, CRBN, LOC100130207, LRRN1, SETMAR, SUMF1, ITPR1-DT, ITPR1, EGOT, BHLHE40-AS1, BHLHE40, ARL8B, EDEM1, MIR4790, GRM7-AS3, GRM7, GRM7-AS2, GRM7-AS1, LOC101927394, LMCD1-AS1, LMCD1, LINC00312, SSUH2, CAV3, OXTR, RAD18, SRGAP3, SRGAP3-AS2, SRGAP3-AS3, SRGAP3-AS4, THUMPD3, THUMPD3-AS1, SETD5, LHFPL4, MTMR14, CPNE9, BRPF1, OGG1, CAMK1, TADA3, ARPC4, ARPC4-TTLL3, TTLL3, RPUSD3, CIDEC, JAGN1, IL17RE, IL17RC, CRELD1, PRRT3, PRRT3-AS1, EMC3, EMC3-AS1, CIDECP1, FANCD2, FANCD2OS, BRK1, VHL, IRAK2, TATDN2, MIR12127, GHRLOS, LINC00852, GHRL, SEC13, ATP2B2, MIR378B, MIR885, ATP2B2-IT2, LINC00606, SLC6A11, SLC6A1, SLC6A1-AS1, HRH1, ATG7, VGLL4, TAMM41, SYN2, TIMP4, PPARG, TSEN2, MKRN2OS, MKRN2, RAF1, TMEM40, CAND2, RPL32, SNORA7A, LINC02022, IQSEC1, NUP210, HDAC11-AS1, HDAC11, FBLN2, SNORA93, LINC00620, WNT7A, FGD5P1, TPRXL, LOC112268445, CHCHD4, TMEM43, XPC, LSM3, LINC01267, SLC6A6, GRIP2, CCDC174, C3orf20, LINC02011, FGD5, FGD5-AS1, NR2C2, MRPS25, RBSN, COL6A4P1, CAPN7, SH3BP5-AS1, SH3BP5, METTL6, EAF1, COLQ, MIR4270, HACL1, BTD, ANKRD28, MIR3134, MIR563, GALNT15, DPH3, OXNAD1, RFTN1, LINC00690, DAZL, PLCL2, MIR3714, PLCL2-AS1, TBC1D5, LOC105376975, BALR6, SATB1, SATB1-AS1, KCNH8, MIR4791, EFHB, RAB5A, PP2D1, KAT2B, MIR3135A, SGO1, SGO1-AS1, LOC101927829, VENTXP7, ZNF385D, ZNF385D-AS1, ZNF385D-AS2, UBE2E2-DT, UBE2E2, UBE2E1-AS1, UBE2E1, NKIRAS1, RPL15, NR1D2, LINC00691, THRB, THRB-AS2, THRB-AS1, RARB, RARB-AS1, TOP2B, MIR4442, NGLY1, OXSM, LINC00692, LRRC3B-AS1, LRRC3B, NEK10, SLC4A7, EOMES, LINC01980, LINC01981, CMC1, AZI2, ZCWPW2, LINC00693, RBMS3-AS3, RBMS3, RBMS3-AS2, RBMS3-AS1, LINC01985, TGFBR2, GADL1, MIR466, STT3B, OSBPL10, OSBPL10-AS1, ZNF860, GPD1L, CMTM8, CMTM7, CMTM6, MIR548AY, DYNC1LI1, CNOT10, TRIM71, CCR4, GLB1, TMPPE, CRTAP, SUSD5, FBXL2, UBP1, CLASP2, PDCD6IP-DT, PDCD6IP, LINC01811, LOC101928135, ARPP21, MIR128-2, STAC, DCLK3, LINC02033, TRANK1, EPM2AIP1, MLH1, LRRFIP2, UBE2FP1, GOLGA4-AS1, GOLGA4, APRG1, ITGA9, ITGA9-AS1, CTDSPL, MIR26A1, VILL, PLCD1, DLEC1, ACAA1, MYD88, OXSR1, SLC22A13, SLC22A14, XYLB, ACVR2B-AS1, ACVR2B, EXOG, SCN5A, SCN10A, SCN11A, WDR48, GORASP1, TTC21A, MIR6822, CSRNP1, XIRP1, CX3CR1, CCR8, SLC25A38, RPSA, SNORA6, SNORA62, MOBP, MYRIP, EIF1B-AS1, EIF1B, ENTPD3, ENTPD3-AS1, RPL14, ZNF619, ZNF620, ZNF621, CTNNB1, ULK4, TRAK1, CCK, LYZL4, VIPR1, VIPR1-AS1, SEC22C, SS18L2, NKTR, ZBTB47-AS1, ZBTB47, KLHL40, HHATL, HHATL-AS1, CCDC13, CCDC13-AS1, CCDC13-AS2, HIGD1A, ACKR2, CYP8B1, ZNF662, KRBOX1-AS1, KRBOX1, GASK1A, POMGNT2, SNRK, SNRK-AS1, ANO10, ABHD5, MIR138-1, TOPAZ1, TCAIM, C3orf86P, LINC01988, ZNF445, ZNF852, ZKSCAN7, ZKSCAN7-AS1, ZNF660, ZNF660-ZNF197, ZNF197-AS1, ZNF197, ZNF35, ZNF502, ZNF501, KIAA1143, KIF15, MIR564, TMEM42, TGM4, ZDHHC3, EXOSC7, CLEC3B, CDCP1, TMEM158, LARS2, LARS2-AS1, LIMD1, LIMD1-AS1, SACM1L, SLC6A20, LZTFL1, CCR9, FYCO1, CXCR6, XCR1, CCR1, CCR3, CCR2, CCR5AS, CCR5, CCRL2, LINC02009, LTF, RTP3, LRRC2, LRRC2-AS1, TDGF1, FAM240A, ALS2CL, TMIE, PRSS50, PRSS46P, PRSS45P, PRSS43P, PRSS44P, PRSS42P, MYL3, PTH1R, CCDC12, NBEAL2, NRADDP, SETD2, KIF9-AS1, KIF9, SNORD13J, KLHL18, PTPN23, SCAP, ELP6, CSPG5, SMARCC1, SNORD146, DHX30, MIR1226, MAP4, CDC25A, MIR4443, CAMP, ZNF589, FCF1P2, NME6, SPINK8, MIR2115, FBXW12, PLXNB1, CCDC51, TMA7, ATRIP, ATRIP-TREX1, TREX1, SHISA5, PFKFB4, MIR6823, UCN2, COL7A1, MIR711, UQCRC1, SNORA94, TMEM89, SLC26A6, MIR6824, CELSR3, MIR4793, LINC02585, NCKIPSD, IP6K2, PRKAR2A, PRKAR2A-AS1, SLC25A20, ARIH2OS, ARIH2, P4HTM, WDR6, DALRD3, MIR425, NDUFAF3, MIR191, IMPDH2, QRICH1, QARS1, MIR6890, USP19, LAMB2, LAMB2P1, CCDC71, KLHDC8B, C3orf84, IHO1, C3orf62, MIR4271, USP4, GPX1, RHOA, TCTA, AMT, NICN1, DAG1, BSN-DT, BSN, APEH, MST1, RNF123, AMIGO3, GMPPB, IP6K1, CDHR4, INKA1, UBA7, MIR5193, TRAIP, CAMKV, MST1R, MON1A, RBM6, RBM5, RBM5-AS1, SEMA3F-AS1, SEMA3F, GNAT1, SLC38A3, GNAI2, MIR5787, SEMA3B-AS1, SEMA3B, MIR6872, LSMEM2, IFRD2, HYAL3, NAA80, HYAL1, HYAL2, TUSC2, RASSF1, RASSF1-AS1, ZMYND10, NPRL2, CYB561D2, LOC127898564, TMEM115, CACNA2D2, LOC101928965, C3orf18, HEMK1, CISH, MAPKAPK3, LINC02019, DOCK3, MIR4787, MANF, RBM15B, DCAF1, RAD54L2, TEX264, GRM2, IQCF6, IQCF4P, IQCF3, IQCF2, IQCF5-AS1, IQCF5, IQCF1, RRP9, PARP3, GPR62, PCBP4, ABHD14B, ABHD14A, ABHD14A-ACY1, ACY1, RPL29, DUSP7, LINC00696, POC1A, ALAS1, TLR9, TWF2, TWF2-DT, PPM1M, WDR82, MIRLET7G, GLYCTK, GLYCTK-AS1, MIR135A1, DNAH1, BAP1, PHF7, SEMA3G, TNNC1, NISCH, STAB1, NT5DC2, UQCC5, PBRM1, GNL3, SNORD136, SNORD19, SNORD19B, SNORD19C, SNORD69, GLT8D1, SPCS1, NEK4, ITIH1, ITIH3, ITIH4, ITIH4-AS1, MUSTN1, STIMATE-MUSTN1, STIMATE, MIR8064, SFMBT1, RFT1, PRKCD, TKT, DCP1A, SNORD38C, CACNA1D, CHDH, IL17RB, ACTR8, SELENOK, CACNA2D3, ESRG, CACNA2D3-AS1, LRTM1, LINC02017, LINC02030, WNT5A, ERC2, ERC2-IT1, MIR3938, CCDC66, TASOR, ARHGEF3, ARHGEF3-AS1, SPATA12, IL17RD, HESX1, APPL1, ASB14, LOC105377102, DNAH12, PDE12, ARF4, ARF4-AS1, DENND6A, DENND6A-DT, SLMAP, FLNB, FLNB-AS1, DNASE1L3, ABHD6, HTD2, RPP14, PXK, PDHB, KCTD6, ACOX2, FAM107A, FAM3D-AS1, FAM3D, CFAP20DC, CFAP20DC-AS1, LOC339902, FHIT, MIR548BB, PTPRG, PTPRG-AS1, C3orf14, FEZF2, CADPS, LINC00698, SYNPR, SYNPR-AS1, SNTN, CDHR18P, C3orf49, THOC7, THOC7-AS1, ATXN7, SCAANT1, PSMD6-AS2, PSMD6, PRICKLE2-AS1, LINC00994, PRICKLE2, PRICKLE2-AS2, PRICKLE2-AS3, PRICKLE2-DT, ADAMTS9, ADAMTS9-AS1, ADAMTS9-AS2, LINC02040, MAGI1, MAGI1-IT1, MAGI1-AS1, SLC25A26, LRIG1, LOC105377143, KBTBD8, MIR4272, SUCLG2, SUCLG2-DT, TAFA1, LOC105377146, TAFA4, EOGT, TMF1, MIR3136, UBA3, ARL6IP5, LMOD3, FRMD4B, MITF, SAMMSON, MDFIC2, FOXP1, FOXP1-AS1, MIR1284, FOXP1-DT, EIF4E3, GPR27, PROK2, LINC00877, LINC00870, RYBP, LOC105377162, SHQ1, GXYLT2, PPP4R2, EBLN2, PDZRN3, LOC101927296, PDZRN3-AS1, LINC02005, LINC02047, CNTN3, MIR4444-2, FAM86DP, LINC02018, MIR1324, FRG2C, LINC00960, ZNF717, MIR4273, ROBO2, LINC02077, ROBO1, LOC101927374, MIR3923, LINC02050, LINC02027, GBE1, LINC02008, LINC00971, LINC02025, CADM2, SNORA95, MIR5688, CADM2-AS2, LINC02070, VGLL3, LINC00506, MIR4795, CHMP2B, POU1F1, HTR1F, CGGBP1, LOC128031834, ZNF654, C3orf38, CSNKA2IP, EPHA3, LOC101930420, PROS1, ARL13B, STX19, DHFR2, NSUN3, LINC00879, MTHFD2P1, MIR8060, EPHA6, ARL6, CRYBG3, RIOX2, GABRR3, OR5AC2, OR5H1, OR5H14, OR5H15 | arr[GRCh38] 3p26.3q11.2(20,214_98,219,206)x3~4 |
| 2  Brain | Gain | 3 | q12.2 | q21.3 | 28,017 | ADGRG7, TFG, ABI3BP, IMPG2, SENP7, FAM172BP, TRMT10C, PCNP, ZBTB11, ZBTB11-AS1, RPL24, PDCL3P4, CEP97, NXPE3, NFKBIZ, RDUR, LOC101929411, ZPLD1, MIR548AB, ALCAM, CBLB, LINC00882, DUBR, CCDC54, CCDC54-AS1, LINC01990, BBX, LINC00635, LINC00636, CD47, LINC01215, IFT57, HHLA2, MYH15, CIP2A, DZIP3, RETNLB, TRAT1, GUCA1C, MORC1, MORC1-AS1, C3orf85, LINC00488, DPPA2, DPPA4, LINC01205, MIR4445, NECTIN3-AS1, NECTIN3, CD96, ZBED2, PLCXD2, PLCXD2-AS1, PHLDB2, ABHD10, TAGLN3, TMPRSS7, C3orf52, MIR567, GCSAM, TBILA, SLC9C1, LOC105374042, CD200, BTLA, ATG3, SLC35A5, CCDC80, LINC02042, MIR9900, CD200R1L-AS1, CD200R1L, CD200R1, GTPBP8, NEPRO, LINC02044, BOC, CFAP44, LOC127898559, CFAP44-AS1, MIR8076, SPICE1, SIDT1, MIR4446, USF3, NAA50, ATP6V1A, GRAMD1C, ZDHHC23, CCDC191, QTRT2, DRD3, ZNF80, TIGIT, ZBTB20, MIR568, ZBTB20-AS1, ZBTB20-AS5, MIR4796, ZBTB20-AS3, ZBTB20-AS4, GAP43, LSAMP, SNORD155, LSAMP-AS1, LINC00903, TUSC7, MIR4447, LINC00901, LINC02024, LINC03051, LOC105374060, LOC101926968, IGSF11, IGSF11-AS1, TEX55, UPK1B, B4GALT4, B4GALT4-AS1, ARHGAP31, ARHGAP31-AS1, TMEM39A, POGLUT1, TIMMDC1, CD80, ADPRH, PLA1A, POPDC2, COX17, CFAP91, NR1I2, GSK3B, MIR6529, GPR156, LRRC58, FSTL1, MIR198, NDUFB4, HGD, RABL3, GTF2E1, LINC02049, STXBP5L, MIR5682, POLQ, ARGFX, FBXO40, HCLS1, GOLGB1, IQCB1, EAF2, SLC15A2, ILDR1, CD86, CASR, CSTA, MIX23, FAM162A, WDR5B, WDR5B-DT, KPNA1, PARP9, DTX3L, PARP15, PARP14, HSPBAP1, SLC49A4, LINC02035, SEMA5B, PDIA5, MIR7110, SEC22A, ADCY5, HACD2, MYLK-AS1, MYLK, MYLK-AS2, CCDC14, ROPN1, KALRN, MIR5002, MIR6083, UMPS, MIR544B, ITGB5, MUC13, HEG1, SLC12A8, MIR5092, ZNF148, SNX4, OSBPL11, LOC105374312, MIR548I1, LINC02614, FAM86JP, ALG1L1P, ROPN1B, SLC41A3, ALDH1L1, ALDH1L1-AS1, ALDH1L1-AS2, KLF15, CFAP100-DT, CFAP100, ZXDC, UROC1, CHST13, C3orf22, TXNRD3, NUP210P1, CHCHD6, PLXNA1, PRR23E, PRR20G, LINC02016, LINC01471, TPRA1, MIR6825, MIR7976, MCM2, PODXL2, ABTB1, MGLL, KBTBD12, SEC61A1, RUVBL1, RUVBL1-AS1, EEFSEC, DNAJB8, DNAJB8-AS1, GATA2, GATA2-AS1, LOC90246, LINC01565, RPN1 | arr[GRCh38] 3q12.2q21.3(100,632,337_128,648,875)x3~4 |
| 2  Brain | Gain | 3 | q22.1 | q22.3 | 4,303 | CPNE4, MIR5704, ACP3, DNAJC13, NPHP3-ACAD11, ACAD11, ACKR4, UBA5, NPHP3, NPHP3-AS1, TMEM108, TMEM108-AS1, BFSP2, BFSP2-AS1, CDV3, TOPBP1, TF, SRPRB, RAB6B, C3orf36, SLCO2A1, LINC02000, RYK, LINC02004, AMOTL2, MIR6827, MIR4788, ANAPC13, CEP63, KY, EPHB1, PPP2R3A | arr[GRCh38] 3q22.1q22.3(131,762,291_136,064,954)x3~4 |
| 2  Brain | Gain | 3 | q22.3 | q29 | 62,028 | PPP2R3A, MSL2, PCCB, STAG1, SLC35G2, NCK1-DT, NCK1, IL20RB-AS1, IL20RB, SOX14, LINC01210, CLDN18, DZIP1L, A4GNT, DBR1, ARMC8, NME9, MRAS, ESYT3, CEP70, FAIM, PIK3CB, LINC01391, FOXL2, FOXL2NB, PRR23A, PRR23B, PRR23C, BPESC1, PISRT1, MRPS22, COPB2, COPB2-DT, RBP2, RBP1, NMNAT3, CLSTN2, CLSTN2-AS1, TRIM42, SLC25A36, SPSB4, PXYLP1, ZBTB38, RASA2, LINC02618, RNF7, GRK7, ATP1B3, TFDP2, GK5, XRN1, ATR, PLS1, TRPC1, PCOLCE2, LOC100507389, PAQR9, PAQR9-AS1, LOC100289361, U2SURP, CHST2, SLC9A9, SLC9A9-AS1, DIPK2A, LNCSRLR, PLOD2, PLSCR4, PLSCR2, PLSCR1, PLSCR5, LINC02010, ZIC4, ZIC4-AS1, ZIC1, LOC440982, LINC02032, LINC02046, AGTR1, CPB1, CPA3, GYG1, HLTF, HLTF-AS1, HPS3, CP, TM4SF18, TM4SF1, TM4SF1-AS1, TM4SF4, WWTR1, WWTR1-IT1, WWTR1-AS1, COMMD2, ANKUB1, RNF13, PFN2, LOC646903, LINC01998, LOC105374313, LINC01213, LINC01214, TSC22D2, SERP1, EIF2A, SELENOT, ERICH6, ERICH6-AS1, LOC101928105, SIAH2, MINDY4B, CLRN1, CLRN1-AS1, MED12L, GPR171, P2RY14, GPR87, P2RY13, P2RY12, IGSF10, LINC02066, MIR5186, AADACL2, AADACL2-AS1, AADACP1, AADAC, SUCNR1, LOC101928166, MBNL1, MBNL1-AS1, TMEM14EP, P2RY1, RAP2B, LINC02006, LINC02877, ARHGEF26-AS1, ARHGEF26, DHX36, GPR149, MME, LINC01487, STRIT1, PLCH1-AS1, PLCH1, PLCH1-AS2, C3orf33, SLC33A1, GMPS, KCNAB1, KCNAB1-AS2, KCNAB1-AS1, SSR3, TIPARP-AS1, TIPARP, LINC00886, PA2G4P4, LEKR1, LINC00880, LINC02029, LINC00881, CCNL1, VEPH1, PTX3, SLC66A1L, SHOX2, RSRC1, UC.134, MLF1-DT, MLF1, GFM1, LXN, RARRES1, LOC100287290, MFSD1, IQCJ, IQCJ-SCHIP1, SCHIP1, MIR3919, IQCJ-SCHIP1-AS1, IL12A-AS1, IL12A, LINC01100, C3orf80, TRIM59-IFT80, IFT80, SMC4, MIR15B, MIR16-2, TRIM59, KPNA4, SCARNA7, ARL14, PPM1L, B3GALNT1, NMD3, SPTSSB, LINC02067, OTOL1, LINC01192, MIR1263, LINC01324, SI, SLITRK3, LINC01322, BCHE, LOC105374194, ZBBX, LINC01327, SERPINI2, WDR49, PDCD10, SERPINI1, LRRC77P, GOLIM4, EGFEM1P, MIR551B, LINC02082, MECOM, MECOM-AS1, TERC, ACTRT3, MYNN, LRRC34, LRRIQ4, LRRC31, SAMD7, FHL1P1, SEC62, GPR160, PHC3, PRKCI, SKIL, CLDN11, MIR6828, SLC7A14, SLC7A14-AS1, RPL22L1, EIF5A2, SLC2A2, TNIK, MIR569, PLD1, TMEM212, TMEM212-AS1, FNDC3B, GHSR, TNFSF10, LINC02068, NCEH1, ECT2, SPATA16, NLGN1, NLGN1-AS1, NAALADL2, NAALADL2-AS3, NAALADL2-AS2, MIR4789, NAALADL2-AS1, MIR7977, LINC01208, LINC01209, TBL1XR1, TBL1XR1-AS1, LINC00501, LINC00578, LINC02015, LINC01014, KCNMB2-AS1, KCNMB2, ZMAT3, PIK3CA-DT, PIK3CA, KCNMB3, ZNF639, MFN1, GNB4, ACTL6A, MRPL47, NDUFB5, USP13, PEX5L, PEX5L-AS2, LINC02053, TTC14-DT, TTC14, CCDC39, LOC101928882, FXR1, DNAJC19, SOX2-OT, LOC102724604, SOX2, LINC01206, LINC01994, LINC01995, ATP11B, DCUN1D1, MCCC1, LAMP3, MCF2L2, B3GNT5, SNHG33, SNORA63D, SNORA63E, KLHL6, KLHL6-AS1, KLHL24, YEATS2, YEATS2-AS1, MAP6D1, PARL, MIR4448, ABCC5, ABCC5-AS1, HTR3D, HTR3C, HTR3E-AS1, HTR3E, EIF2B5-DT, EIF2B5, DVL3, AP2M1, ABCF3, VWA5B2, MIR1224, ALG3, EEF1AKMT4, EEF1AKMT4-ECE2, CAMK2N2, ECE2, PSMD2, EIF4G1, SNORD66, FAM131A, CLCN2, POLR2H, THPO, CHRD, LINC02054, LINC01840, EPHB3, MAGEF1, LOC107986163, LINC02069, VPS8, C3orf70, EHHADH-AS1, EHHADH, MIR5588, MAP3K13, TMEM41A, LIPH, SENP2, IGF2BP2, IGF2BP2-AS1, MIR548AQ, TRA2B, NMRAL2P, ETV5, DGKG, LINC02020, LINC02052, CRYGS, TBCCD1, DNAJB11, AHSG, FETUB, HRG, KNG1, EIF4A2, SNORD2, SNORA63B, MIR1248, SNORA81, SNORA63, SNORA4, RFC4, LINC02043, ADIPOQ, ADIPOQ-AS1, ST6GAL1, RPL39L, LOC101929106, RTP1, MASP1, LOC101929130, RTP4, LINC02041, SST, RTP2, LOC100131635, BCL6, LOC122526776, LINC01991, LPP-AS2, LPP, FLJ42393, LPP-AS1, MIR28, TPRG1-AS1, TPRG1, TPRG1-AS2, TP63, MIR944, P3H2, P3H2-AS1, CLDN16, CLDN1, TMEM207, IL1RAP, LINC02013, GMNC, SNAR-I, OSTN, OSTN-AS1, UTS2B, CCDC50, PYDC2-AS1, PYDC2, FGF12, FGF12-AS1, FGF12-AS2, MB21D2, PLAAT1, MGC2889, ATP13A5, ATP13A5-AS1, ATP13A4, ATP13A4-AS1, OPA1, OPA1-AS1, LINC02038, LINC02026, DPPA2P3, LINC02028, HES1, LINC02036, LINC02037, LINC02048, LINC00887, CPN2, LRRC15, GP5, ATP13A3, ATP13A3-DT, TMEM44-AS1, TMEM44, LSG1, FAM43A, LINC01968, LINC01972, XXYLT1, XXYLT1-AS1, MIR3137, XXYLT1-AS2, ACAP2, PPP1R2, APOD, LOC105374297, SDHAP2, MIR570HG, MIR570, MUC20, MUC4, LINC01983, TNK2, MIR6829, TNK2-AS1, SDHAP1, TFRC, LINC00885, ZDHHC19, SLC51A, PCYT1A, DYNLT2B, TM4SF19-DYNLT2B, TM4SF19-AS1, TM4SF19, UBXN7, UBXN7-AS1, RNF168, SMCO1, WDR53, FBXO45, LINC01063, NRROS, CEP19, PIGX, PAK2, SENP5, NCBP2, NCBP2-AS1, NCBP2AS2, PIGZ, MELTF, MELTF-AS1, DLG1, MIR4797, DLG1-AS1, LINC02012, BDH1, SDHAP4, RUBCN, MIR922, FYTTD1, LRCH3, IQCG, RPL35A, LMLN, LMLN-AS1, ANKRD18DP | arr[GRCh38] 3q22.3q29(136,097,460_198,125,115)x3 |
| 2  Brain | cn-LOH | 4 | p16.3 | p11 | 49,018 | ZNF595, ZNF718, ZNF876P, ZNF732, ZNF141, MIR571, ABCA11P, ZNF721, PIGG, TMEM271, LOC105374338, PDE6B, PDE6B-AS1, ATP5ME, MYL5, SLC49A3, PCGF3, PCGF3-AS1, CPLX1, GAK, TMEM175, DGKQ, SLC26A1, IDUA, FGFRL1, RNF212, LOC105374344, TMED11P, SPON2, LOC100130872, CTBP1-AS, CTBP1, CTBP1-DT, MAEA, UVSSA, NKX1-1, FAM53A, SLBP, TMEM129, TACC3, FGFR3, LETM1, NSD2, SCARNA22, NELFA, MIR943, C4orf48, NAT8L, POLN, HAUS3, MXD4, MIR4800, ZFYVE28, CFAP99, RNF4, FAM193A, TNIP2, SH3BP2, ADD1, MFSD10, NOP14-AS1, NOP14, GRK4, HTT-AS, HTT, MSANTD1, RGS12, HGFAC, DOK7, LRPAP1, LINC00955, LINC02171, ADRA2C, FAM86EP, OTOP1, TMEM128, LYAR, ZBTB49, NSG1, STX18, STX18-IT1, STX18-AS1, SNORD162, LOC101928279, LINC01396, MSX1, LOC101928306, CYTL1, STK32B, LINC01587, EVC2, EVC, CRMP1, MIR378D1, C4orf50, JAKMIP1, LOC128125818, JAKMIP1-DT, WFS1, PPP2R2C, MAN2B2, MRFAP1, LINC02482, LOC93622, LINC02481, S100P, MRFAP1L1, BLOC1S4, KIAA0232, TBC1D14, LOC100129931, CCDC96, TADA2B, GRPEL1, LINC02447, SORCS2, MIR4798, PSAPL1, MIR4274, AFAP1-AS1, AFAP1, LOC389199, ABLIM2, MIR95, SH3TC1, HTRA3, LINC02517, ACOX3, TRMT44, GPR78, CPZ, HMX1, FAM90A26, USP17L10, USP17L11, USP17L12, USP17L13, USP17L15, USP17L17, USP17L18, USP17L19, USP17L20, USP17L21, USP17L22, USP17L24, USP17L25, USP17L26, USP17L5, USP17L27, USP17L28, USP17L29, USP17L9P, USP17L30, USP17L6P, DEFB131A, MIR548I2, DRD5, SLC2A9, SLC2A9-AS1, WDR1, MIR3138, ZNF518B, CLNK, MIR572, HS3ST1, LINC02360, MIR12113, LINC02270, RAB28, LINC01097, NKX3-2, LINC01096, BOD1L1, MIR5091, LINC01182, LINC01085, LINC00504, CPEB2-DT, CPEB2, C1QTNF7-AS1, C1QTNF7, CC2D2A, FBXL5, FAM200B, BST1, CD38, FGFBP1, FGFBP2, PROM1, TAPT1, TAPT1-AS1, LDB2, LINC02493, SNORA75B, QDPR, CLRN2, LAP3, MED28, FAM184B, DCAF16, NCAPG, LCORL, SLIT2, SLIT2-IT1, MIR218-1, PACRGL, KCNIP4, MIR7978, LOC105374516, KCNIP4-IT1, LOC100505912, ADGRA3, MIR12115, GBA3, PPARGC1A, MIR573, DHX15, LINC02473, SOD3, CCDC149, LGI2, SEPSECS, SEPSECS-AS1, PI4K2B, ZCCHC4, ANAPC4, LOC101929161, SLC34A2, SEL1L3, SMIM20, RBPJ, CCKAR, TBC1D19, STIM2-AS1, STIM2, LINC02261, MIR4275, LINC02364, LINC02472, PCDH7, LINC02497, LINC02501, LINC02506, LOC105377651, LINC02353, LOC101928622, LINC02484, ARAP2, LOC439933, DTHD1, MIR1255B1, LINC02505, LINC02616, MIR4801, NWD2, C4orf19, RELL1, PGM2, TBC1D1, PTTG2, LINC02513, LINC01258, LINC01259, LINC02278, KLF3-AS1, KLF3, TLR10, TLR1, TLR6, FAM114A1, MIR574, TMEM156, KLHL5, WDR19, RFC1, KLB, MIR5591, RPL9, LIAS, LOC401127, UGDH, UGDH-AS1, SMIM14, SMIM14-DT, UBE2K, PDS5A, LOC344967, N4BP2, RHOH, LINC02265, CHRNA9, RBM47, MIR4802, NSUN7, APBB2, UCHL1-DT, UCHL1, LIMCH1, PHOX2B, LINC00682, TMEM33, DCAF4L1, SLC30A9, BEND4, LOC105374428, SHISA3, ATP8A1, GRXCR1, LINC02383, LINC02475, KCTD8, YIPF7, GUF1, GNPDA2, GABRG1, GABRA2, COX7B2, GABRA4, GABRB1, COMMD8, ATP10D, CORIN, MIR8053, LOC101927179, NFXL1, LOC101927157, CNGA1, NIPAL1, TXK, TEC, SLAIN2, SLC10A4, ZAR1, FRYL, OCIAD1, OCIAD1-AS1, OCIAD2, CWH43 | arr[GRCh38] 4p16.3p11(69,019_49,087,164)x3 hmz |
| 2  Brain | cn-LOH | 4 | q12 | q35.2 | 138,161 | DCUN1D4, LRRC66, SGCB, LINC02480, SPATA18, USP46, USP46-DT, DANCR, MIR4449, SNORA26, ERVMER34-1, LINC01618, RASL11B, SCFD2, FIP1L1, LNX1, LNX1-AS1, LNX1-AS2, LOC100506444, RPL21P44, CHIC2, GSX2, PDGFRA, LINC02283, LINC02260, KIT, KDR, SRD5A3, SRD5A3-AS1, TMEM165, CLOCK, PDCL2, NMU, EXOC1L, EXOC1, CEP135, CRACD, AASDH, PPAT, PAICS, SRP72, ARL9, THEGL, HOPX, SPINK2, REST, NOA1, POLR2B, IGFBP7, IGFBP7-AS1, LINC02380, LINC02494, LINC02429, LINC02619, LINC02496, MIR548AG1, LINC02271, ADGRL3, ADGRL3-AS1, TECRL, LINC02232, EPHA5, EPHA5-AS1, MIR1269A, LOC101927237, CENPC, STAP1, UBA6, UBA6-DT, GNRHR, TMPRSS11D, TMPRSS11A, TMPRSS11GP, TMPRSS11F, LOC550113, SYT14P1, FTLP10, TMPRSS11BNL, TMPRSS11B, YTHDC1, TMPRSS11E, UGT2B17, UGT2B15, UGT2B10, UGT2A3, UGT2B7, LOC105377267, UGT2B11, UGT2B28, UGT2B4, UGT2A2, UGT2A1, SULT1B1, SULT1E1, CSN1S1, CSN2, STATH, HTN3, HTN1, CSN1S2AP, CSN1S2BP, PRR27, ODAM, FDCSP, CSN3, CABS1, SMR3A, SMR3B, OPRPN, MUC7, AMTN, AMBN, ENAM, JCHAIN, UTP3, RUFY3, GRSF1, MOB1B, DCK, SLC4A4, GC, NPFFR2, ADAMTS3, COX18, ANKRD17, ALB, AFP, AFM, LINC02499, RASSF6, CXCL8, CXCL6, PF4V1, CXCL1, PF4, PPBP, CXCL5, CXCL3, PPBPP2, CXCL2, MTHFD2L, EPGN, EREG, AREG, BTC, PARM1, PARM1-AS1, LINC02562, LINC02483, RCHY1, THAP6, ODAPH, CDKL2, G3BP2, USO1, PPEF2, NAAA, SDAD1, SDAD1-AS1, CXCL9, ART3, CXCL10, CXCL11, NUP54, SCARB2, FAM47E, FAM47E-STBD1, STBD1, CCDC158, SHROOM3, MIR4450, MIR548AH, SOWAHB, SEPTIN11, CCNI, CCNG2, CXCL13, CNOT6L, MRPL1, FRAS1, SNORD161, ANXA3, LINC01094, BMP2K, PAQR3, LINC01088, NAA11, GK2, LINC00989, PCAT4, ANTXR2, PRDM8-AS1, PRDM8, FGF5, CFAP299, BMP3, PRKG2, PRKG2-AS1, RASGEF1B, HNRNPD, HNRNPD-DT, HNRNPDL, ENOPH1, TMEM150C, LINC00575, SCD5, MIR575, SEC31A, THAP9-AS1, SNORD143, SNORD144, THAP9, LIN54, COPS4, PLAC8, COQ2, HPSE, HELQ, MRPS18C, ABRAXAS1, GPAT3, LINC02994, NKX6-1, CDS1, WDFY3, WDFY3-AS1, WDFY3-AS2, ARHGAP24, MIR4451, MAPK10, MAPK10-AS1, MIR4452, PTPN13, SLC10A6, C4orf36, AFF1-AS1, AFF1, KLHL8, MIR5705, HSD17B13, HSD17B11, NUDT9, SCPPPQ1, SPARCL1, DSPP, DMP1, IBSP, MEPE, SPP1, PKD2, ABCG2, PPM1K, PPM1K-DT, HERC6, HERC5, PIGY, PYURF, PIGY-DT, HERC3, NAP1L5, FAM13A-AS1, FAM13A, TIGD2, GPRIN3, SNCA, SNCA-AS1, MMRN1, CCSER1, LNCPRESS2, GRID2, ATOH1, SMARCAD1-DT, SMARCAD1, HPGDS, PDLIM5, BMPR1B-DT, BMPR1B, UNC5C, UNC5C-AS1, PDHA2, LINC02267, STPG2-AS1, STPG2, RAP1GDS1, TSPAN5, TSPAN5-DT, EIF4E, METAP1, MIR3684, ADH5, LOC100507053, ADH4, PCNAP1, ADH6, ADH1A, ADH1B, ADH1C, ADH7, C4orf17, TRMT10A, MTTP, C4orf54, DAPP1, LAMTOR3, DNAJB14, H2AZ1, H2AZ1-DT, DDIT4L, DDIT4L-AS1, SNORA101A, EMCN, LINC01216, LINC01217, PPP3CA, MIR8066, MIR1255A, FLJ20021, BANK1, SLC39A8, LOC105377621, NFKB1, MANBA, LOC102723704, UBE2D3, UBE2D3-AS1, CISD2, SLC9B1, SLC9B2, BDH2, CENPE, LINC02428, TACR3, CXXC4, CXXC4-AS1, TET2, TET2-AS1, PPA2, ARHGEF38, ARHGEF38-IT1, INTS12, GSTCD, GSTCD-AS1, NPNT, LOC101929577, TBCK, AIMP1, GIMD1, LINC02173, DKK2, PAPSS1, SGMS2, CYP2U1-AS1, CYP2U1, HADH, LEF1, LEF1-AS1, RPL34-DT, LOC101929621, RPL34, OSTC, ETNPPL, COL25A1, COL25A1-DT, SEC24B-AS1, SEC24B, MIR576, MCUB, CASP6, PLA2G12A, CFI, GAR1, RRH, LRIT3, EGF, ELOVL6, ENPEP, PANCR, PITX2, MIR297, FAM241A, AP1AR, TIFA, ALPK1, NEUROG2, NEUROG2-AS1, ZGRF1, LARP7, MIR302CHG, MIR367, MIR302D, MIR302A, MIR302C, MIR302B, ANK2, MIR1243, MIR8082, CAMK2D, ARSJ, UGT8, MIR577, NDST4, MIR1973, TRAM1L1, LINC02262, LINC02263, LINC01378, LINC02264, NDST3, SNHG8, SNORA24, PRSS12, CEP170P1, LOC729218, METTL14-DT, METTL14, SEC24D, SYNPO2, MYOZ2, LOC101929762, USP53, C4orf3, FABP2, LINC01061, GTF2IP12, SEPTIN7P14, PDE5A, LOC107986192, LINC01365, LINC02502, MAD2L1, PRDM5, NDNF, TNIP3, QRFPR, ANXA5, SMIM43, PP12613, EXOSC9, CCNA2, BBS7, TRPC3, BLTP1, ADAD1, IL2, IL21, IL21-AS1, CETN4P, BBS12, FGF2, NUDT6, SPATA5, SPRY1, LINC01091, LINC02516, ANKRD50, FAT4, MIR2054, LINC02379, INTU, SLC25A31, HSPA4L, PLK4, MFSD8, ABHD18, LARP1B, PGRMC2, LINC02615, JADE1, SCLT1, C4orf33, LINC02466, LINC02465, LINC02479, LINC02377, SNHG27, LINC01256, PCDH10-DT, PCDH10, PABPC4L, LINC02462, LINC02485, LINC00613, LINC02511, LINC02510, PCDH18, LINC02172, LINC00616, SLC7A11-AS1, SLC7A11, LINC00499, LOC105377448, NOCT, ELF2, MGARP, NDUFC1, NAA15, RAB33B-AS1, RAB33B, SETD7, LOC105377622, MGST2, MAML3, LOC101927516, SCOC, SCOC-AS1, CLGN, MGAT4D, ELMOD2, UCP1, TBC1D9, TNRC18P1, RNF150, ZNF330, LINC02432, IL15, INPP4B, LOC105377623, USP38, GAB1, MIR3139, SMARCA5-AS1, SMARCA5, GUSBP5, FREM3, LOC105377458, GYPE, LOC101927636, GYPB, GYPA, HHIP-AS1, HHIP, ANAPC10, ABCE1, OTUD4, LINC02266, SMAD1, SMAD1-AS2, SMAD1-AS1, LINC02491, MMAA, C4orf51, ZNF827, LINC01095, LSM6, REELD1, SLC10A7, MIR7849, POU4F2, TTC29, MIR548G, EDNRA, TMEM184C, PRMT9, ARHGAP10, MIR4799, NR3C2, LOC105377480, LINC02355, IQCM, DCLK2, LRBA, LOC729558, MAB21L2, RPS3A, SNORD73B, SNORD73A, SH3D19, PRSS48, FHIP1A-DT, FHIP1A, GATB, LOC105377488, LOC127898557, LOC127898556, LINC03074, LINC02273, FBXW7, FBXW7-AS1, MIR3140, MIR4453HG, MIR4453, TMEM154, TIGD4, ARFIP1, LOC729870, FHDC1, TRIM2, ANXA2P1, MND1, TMEM131L, LOC100419170, TLR2, RNF175, SFRP2, DCHS2, PLRG1, FGB, FGA, FGG, LRAT, RBM46, NPY2R, MAP9, MAP9-AS1, GUCY1A1, GUCY1B1, ASIC5, TDO2, CTSO, PDGFC, GLRB, GRIA2, LINC02433, GASK1B, GASK1B-AS1, TMEM144, RXFP1, C4orf46, ETFDH, PPID, FNIP2, C4orf45, RAPGEF2, MIR3688-1, MIR3688-2, LINC02233, FSTL5, LOC101928052, MIR4454, NAF1, NPY1R, NPY5R, TKTL2, TMA16, MARCHF1, SMIM31, APELA, TRIM61, FAM218A, TRIM60, TRIM75, TMEM192, KLHL2, GK3, MSMO1, CPE, MIR578, LINC01179, LOC101928131, TLL1, SPOCK3, ANXA10, DDX60, DDX60L, PALLD, CBR4, SH3RF1, NEK1, CLCN3, HPF1, LINC02275, MFAP3L, LOC101928198, AADAT, LINC01612, LINC02382, LINC02431, MIR6082, LINC02504, LINC02174, LOC441052, GALNTL6, GALNTL6-AS1, GALNT7-DT, GALNT7, MIR548T, HMGB2, SAP30-DT, SAP30, SCRG1, HAND2, HAND2-AS1, LINC02269, LINC02268, FBXO8, CEP44, MIR4276, HPGD, GLRA3, LOC101928551, ADAM29, GPM6A, GPM6A-DT, WDR17, SPATA4, ASB5, SPCS3, HAFML, VEGFC, LINC02509, NEIL3, AGA, AGA-DT, LINC01098, LINC01099, LINC00290, LINC02500, TEMN3-AS1, TENM3-AS1, TENM3, MIR1305, DCTD, CIBAR1P2, WWC2-AS2, WWC2, WWC2-AS1, CLDN22, CLDN24, CDKN2AIP, ING2-DT, ING2, RWDD4, TRAPPC11, STOX2, ENPP6, LINC02363, LINC02362, IRF2, LINC02427, LINC02365, CASP3, PRIMPOL, CENPU, ACSL1, SLED1, MIR3945HG, MIR3945, LINC01093, MIR4455, HELT, LINC02436, SLC25A4, CFAP97, SNX25, LRP2BP, ANKRD37, UFSP2, C4orf47, CCDC110, LOC105377590, PDLIM3, SORBS2, TLR3, FAM149A, FLJ38576, CYP4V2, KLKB1, F11, F11-AS1, MTNR1A, FAT1, LINC02374, LOC339975, LINC02514, LINC02515, LINC02492, ZFP42, TRIML2, TRIML1, LINC01060, LINC02508, LINC01262, FRG1-DT, LINC01596, FRG1 | arr[GRCh38] 4q12q35.2(51,833,594_189,994,495)x3 hmz |
| 2  Brain | Gain | 5 | p15.33 | p11 | 43,654 | IRX2, IRX2-DT, LOC105374620, LINC01377, LINC01019, LINC02162, LINC01017, IRX1, LINC02063, LINC02114, LINC01020, LINC02121, ADAMTS16-DT, ADAMTS16, ICE1, LINC02145, MED10, UBE2QL1, LINC01018, NSUN2, SRD5A1, LINC02102, TENT4A, LINC02236, MIR4278, LOC442132, LINC02123, LINC02142, ADCY2, CFAP90, FASTKD3, MTRR, LINC02226, MIR4458HG, MIR4458, LINC02199, SEMA5A, MIR4636, SEMA5A-AS1, SNHG18, SNORD123, TAS2R1, LINC02112, LINC02221, ATPSCKMT, CCT5, CMBL, MARCHF6-DT, MARCHF6, MIR10397, ROPN1L-AS1, ROPN1L, MIR6131, LINC02212, LINC02213, ANKRD33B, DAP, CTNND2, LINC01194, LINC02220, DNAH5, TRIO, SNORD170, OTULINL, SNORD141B, OTULIN-DT, OTULIN, ANKH, LOC100130744, MIR4637, LINC02149, FBXL7, CTD-2350J17.1, MIR887, MARCHF11, MARCHF11-DT, LINC02150, ZNF622, RETREG1, RETREG1-AS1, MYO10, BASP1-AS1, MIR10522, BASP1, LINC02111, LINC02217, LINC02218, H3Y2, TAF11L2, TAF11L3, TAF11L4, TAF11L5, TAF11L6, TAF11L7, TAF11L8, TAF11L9, TAF11L10, TAF11L11, TAF11L12, TAF11L13, TAF11L14, H3Y1, LINC02223, CDH18, CDH18-AS1, LINC02241, LINC02146, GUSBP1, CDH12, SNORA105A, PMCHL1, PRDM9, LINC02899, CDH10, LINC02239, LINC02228, LINC02211, CDH9, PURPL, LINC02103, LSP1P3, LOC101929645, LINC02109, LINC02064, LOC105374704, CDH6, DROSHA, C5orf22, PDZD2, MIR4279, GOLPH3, MTMR12, ZFR, MIR579, SUB1, NPR3, LINC02120, LINC02160, TARS1, ADAMTS12, RXFP3, SLC45A2, AMACR, C1QTNF3-AMACR, C1QTNF3, LOC646652, RAI14-DT, RAI14, TTC23L-AS1, TTC23L, RAD1, BRIX1, DNAJC21, AGXT2, PRLR, SPEF2, IL7R, CAPSL, CAPSL-DT, UGT3A1, UGT3A2, LMBRD2, MIR580, SKP2, NADK2, NADK2-AS1, RANBP3L, SLC1A3, NIPBL-DT, NIPBL, CPLANE1, CPLANE1-AS1, NUP155, WDR70, GDNF, GDNF-AS1, LINC02110, LINC02107, LINC02119, EGFLAM, EGFLAM-AS4, EGFLAM-AS2, EGFLAM-AS1, LIFR, LIFR-AS1, MIR3650, OSMR-DT, LINC01265, OSMR, RICTOR, FYB1, C9, DAB2, LINC02104, LINC00603, LINC00604, PTGER4, TTC33, PRKAA1, RPL37, SNORD72, CARD6, C7, MROH2B, C6, PLCXD3, OXCT1, OXCT1-AS1, RIMOC1, FBXO4, LINC02996, GHR, CCDC152, SELENOP, FLJ32255, ANXA2R-OT1, ANXA2R, ANXA2R-AS1, LOC100132356, LOC100506639, ZNF131, NIM1K, HMGCS1, CCL28, TMEM267, C5orf34, PAIP1, NNT-AS1, NNT, FGF10, FGF10-AS1, LINC02224, MRPS30-DT, MRPS30, HCN1 | arr[GRCh38] 5p15.33p11(2,735,007_46,389,237)x3~4 |
| 2  Brain | Gain | 5 | q11.1 | q13.2 | 19,158 | EMB, PARP8, LINC02106, ISL1-DT, ISL1, LINC02118, PELO, ITGA1, ITGA2, MOCS2, MOCS2-DT, FST, NDUFS4, LINC02105, ARL15, MIR581, LINC01033, HSPB3, SNX18, LINC02998, ESM1, LOC102467081, GZMK, GZMA, CDC20B, GPX8, MIR449A, MIR449B, MIR449C, MCIDAS, CCNO, DHX29, MTREX, PLPP1, MIR5687, RNF138P1, SLC38A9, DDX4, IL31RA, IL6ST, IL6ST-DT, ANKRD55, LINC01948, C5orf67, LOC105378979, MAP3K1, SETD9, MIER3, GPBP1, ACTBL2, LNCBRM, LOC101928505, LINC02225, LINC02101, PLK2, GAPT, MIR548AE2, LINC02108, RAB3C, PDE4D, MIR582, PART1, DEPDC1B, ELOVL7, ERCC8, ERCC8-AS1, NDUFAF2, SMIM15, SMIM15-AS1, LINC02057, ZSWIM6, C5orf64, LOC101928651, C5orf64-AS1, KIF2A, DIMT1, IPO11, IPO11-LRRC70, LRRC70, HTR1A, RNF180, RGS7BP, SHISAL2B, SREK1IP1, CWC27, ADAMTS6, CENPK, PPWD1, TRIM23, SHLD3, TRAPPC13, SGTB, NLN, ERBIN, LOC100303749, SREK1, LINC02065, LINC02229, MAST4, MAST4-AS1, CD180, LINC02242, LINC02997, LINC02219, PIK3R1, LINC02198, SLC30A5, SNORA50D, CCNB1, CENPH, MRPS36, CDK7, CCDC125, AK6, TAF9, RAD17, MARVELD2, LOC101928924, OCLN, SNORD13B-1 | arr[GRCh38] 5q11.1q13.2(50,393,189_69,551,239)x3~4 |
| 2  Brain | Gain | 5 | q13.2 | q21.3 | 36,922 | LINC02230, FOXD1, LINC01386, BTF3, ANKRA2, UTP15, ARHGEF28, LINC02122, LINC01335, LINC01333, LINC01331, ENC1, HEXB, GFM2, NSA2, FAM169A, FAM169A-AS1, GCNT4, ANKRD31, HMGCR, CERT1, POLK, ANKDD1B, POC5, LOC441087, SV2C-AS1, SV2C, IQGAP2, LOC101929109, F2RL2, NCRUPAR, F2R, F2RL1, S100Z, CRHBP, AGGF1, ZBED3, SNORA47, ZBED3-AS1, PDE8B, WDR41, OTP, TBCA, LOC101929154, AP3B1, SCAMP1-AS1, SCAMP1, LHFPL2, ARSB, DMGDH, BHMT2, BHMT, JMY, HOMER1, TENT2, CMYA5, LINC01455, MTX3, THBS4, THBS4-AS1, SERINC5, LOC644936, SPZ1, CRSP8P, ZFYVE16, FAM151B-DT, FAM151B, ANKRD34B, LINC01337, DHFR, MSH3, RASGRF2-AS1, RASGRF2, CKMT2, CKMT2-AS1, ZCCHC9, ACOT12, SSBP2, ATG10, RPS23, ATP6AP1L, MIR3977, LINC01338, TMEM167A, SCARNA18, XRCC4, VCAN, VCAN-AS1, HAPLN1, EDIL3, EDIL3-DT, NBPF22P, COX7C, SNORD138, LINC02059, MIR4280, LOC101929380, LINC01949, RASA1, CCNH, LOC644285, LINC02144, LINC02488, TMEM161B, TMEM161B-DT, LINC02060, LINC00461, MIR9-2, MEF2C-AS2, MEF2C, MEF2C-AS1, MIR3660, LINC01339, CETN3, LOC731157, MBLAC2, POLR3G, LYSMD3, ADGRV1, LUCAT1, ARRDC3, ARRDC3-AS1, NR2F1-AS1, NR2F1, FAM172A, MIR2277, POU5F2, KIAA0825, SLF1, MCTP1, FAM81B, SKIC3, ARSK, GPR150, RFESD, SPATA9, RHOBTB3, GLRX, LOC102724720, LINC01554, ELL2, LOC101929710, MIR583, PCSK1, CAST, ERAP1, ERAP2, LNPEP, LIX1, RIOK2, LINC01340, LINC02234, LINC01846, RGMB, RGMB-AS1, CHD1, CHD1-DT, LOC100289230, LINC02113, GUSBP19, FAM174A-DT, FAM174A, ST8SIA4, MIR548P, SLCO4C1, SLCO6A1, LINC00492, LINC00491, PAM, GIN1, PPIP5K2, MACIR, LINC02115, NUDT12, RAB9BP1, LINC01950, EFNA5, FBXL17, LINC01023, FER, LOC285638, PJA2, MAN2A1, LINC01848 | arr[GRCh38] 5q13.2q21.3(73,272,746_110,195,206)x3~4 |
| 2  Brain | Gain | 5 | q22.2 | q35.3 | 67,222 | MCC, TSSK1B, YTHDC2, KCNN2, LOC101927078, LINC01957, TRIM36, PGGT1B, CCDC112, FEM1C, TICAM2, TMED7-TICAM2, TICAM2-AS1, TMED7, LOC102467217, CDO1, ATG12, AP3S1, LINCADL, LVRN, ARL14EPL, COMMD10, MIR12130, LOC101927190, SEMA6A, SEMA6A-AS1, SEMA6A-AS2, LINC02214, LINC00992, LINC02147, LINC02148, LINC02208, LINC02216, LINC02215, DTWD2, MIR1244-2, DMXL1-DT, DMXL1, MIR5706, TNFAIP8, HSD17B4, FAM170A, PRR16, LOC102467226, FTMT, SRFBP1, LOX, ZNF474, LOC100505841, SNCAIP, MGC32805, LOC101927357, LINC02201, SNX2, SNX24, PPIC, PRDM6-AS1, PRDM6, CEP120, CSNK1G3, LINC01170, ZNF608, LOC101927421, LINC02240, LINC02039, LOC101927488, GRAMD2B, ALDH7A1, PHAX, TEX43, LMNB1-DT, LMNB1, MARCHF3, C5orf63, MEGF10, PRRC1, CTXN3, CCDC192, SLC12A2-DT, SLC12A2, FBN2, SLC27A6, ISOC1, MIR4633, MIR4460, ADAMTS19-AS1, ADAMTS19, MINAR2, CHSY3, HINT1, LYRM7, CDC42SE2, RAPGEF6, FNIP1, MEIKIN, ACSL6, ACSL6-AS1, IL3, CSF2, P4HA2-AS1, P4HA2, MIR6830, PDLIM4, SLC22A4, MIR3936HG, MIR3936, SLC22A5, IRF1-AS1, IRF1, IL5, RAD50, TH2LCRR, IL13, IL4, LOC105379176, KIF3A, CCNI2, SEPTIN8, SOWAHA, SHROOM1, GDF9, UQCRQ, LEAP2, AFF4, ZCCHC10, HSPA4, FSTL4, MIR1289-2, WSPAR, C5orf15, LOC105379183, VDAC1, TCF7, SKP1, PPP2CA, MIR3661, CDKL3, UBE2B, CDKN2AIPNL, LINC02999, LINC01843, JADE2, SAR1B, SEC24A, CAMLG, DDX46, C5orf24, TXNDC15, PCBD2, CATSPER3, PITX1, PITX1-AS1, EPIST, LINC02900, MACROH2A1, DCANP1, TIFAB, NEUROG1, CXCL14, SLC25A48, SLC25A48-AS1, MIR5692C1, LOC107986453, IL9, FBXL21P, LECT2, TGFBI, VTRNA2-1, SMAD5-AS1, SMAD5, SMIM32, TRPC7, TRPC7-AS1, TRPC7-AS2, SPOCK1, LOC105379192, KLHL3, MIR874, HNRNPA0, NPY6R, MYOT, PKD2L2, FAM13B, FAM13B-AS1, WNT8A, NME5, BRD8, KIF20A, CDC23, GFRA3, CDC25C, LOC100128966, FAM53C, KDM3B, REEP2, EGR1, ETF1, HSPA9, SNORD63B, SNORD63, CTNNA1-AS1, CTNNA1, LRRTM2, SIL1, SNHG4, MATR3, SNORA74D, SNORA74A, PAIP2, SLC23A1, MZB1, PROB1, SPATA24, DNAJC18, ECSCR, SMIM33, STING1, UBE2D2, CXXC5, CXXC5-AS1, PSD2-AS1, PSD2, NRG2, MALINC1, PURA, IGIP, LOC101929719, CYSTM1, PFDN1, HBEGF, SLC4A9, ANKHD1, ANKHD1-EIF4EBP3, EIF4EBP3, SRA1, APBB3, MIR6831, SLC35A4, CD14, TMCO6, NDUFA2, IK, MIR3655, WDR55, DND1, HARS1, HARS2, ZMAT2, VTRNA1-1, VTRNA1-2, VTRNA1-3, PCDHA1, PCDHA2, PCDHA3, PCDHA4, PCDHA5, PCDHA6, PCDHA7, PCDHA8, PCDHA9, PCDHA10, PCDHA11, LOC112267934, PCDHA12, PCDHA13, PCDHAC1, PCDHAC2, PCDHB1-AS1, PCDHB1, PCDHB2, PCDHB3, PCDHB4, PCDHB5, PCDHB6, PCDHB17P, PCDHB7, PCDHB8, PCDHB16, PCDHB9, PCDHB10, PCDHB11, PCDHB12, PCDHB13, PCDHB14, PCDHB18P, PCDHB19P, PCDHB15, SLC25A2, TAF7, PCDHGA1, PCDHGA2, PCDHGA3, PCDHGB1, PCDHGA4, PCDHGB2, PCDHGA5, PCDHGB3, PCDHGA6, PCDHGA7, PCDHGB4, PCDHGA8, PCDHGB5, PCDHGA9, PCDHGB6, PCDHGA10, PCDHGB7, PCDHGA11, PCDHGB8P, PCDHGA12, PCDHGC3, PCDHGC4, PCDHGC5, DIAPH1, DIAPH1-AS1, HDAC3, RELL2, FCHSD1, ARAP3, PCDH1, LOC729080, DELE1, PCDH12, RNF14, GNPDA1, NDFIP1, SPRY4, SPRY4-IT1, SPRY4-AS1, FGF1, LINC01844, ARHGAP26, ARHGAP26-AS1, ARHGAP26-IT1, NR3C1, MIR5197, HMHB1, YIPF5, KCTD16, PRELID2, GRXCR2, SH3RF2, PLAC8L1, LARS1, RBM27, LOC127814297, POU4F3, TCERG1, GPR151, PPP2R2B, PPP2R2B-IT1, STK32A-AS1, STK32A, DPYSL3, JAKMIP2-AS1, JAKMIP2, SPINK1, SCGB3A2, C5orf46, SPINK5, SPINK14, SPINK6, MARCOL, FBXO38-DT, SPINK13, SPINK7, SPINK9, FBXO38, HTR4, ADRB2, SH3TC2, MIR584, SH3TC2-DT, ABLIM3, AFAP1L1, GRPEL2, GRPEL2-AS1, PCYOX1L, IL17B, CARMN, MIR143, MIR145, CSNK1A1, ARHGEF37, PPARGC1B, MIR378A, PDE6A, MFFP2, SLC26A2, TIGD6, HMGXB3, CSF1R, PDGFRB, CDX1, SLC6A7, CAMK2A, ARSI, TCOF1, CD74, RPS14, NDST1-AS1, NDST1, SYNPO, MYOZ3, RBM22, DCTN4, SMIM3, IRGM, ZNF300, ZNF300P1, GPX3, TNIP1, ANXA6, CCDC69, LOC105378230, GM2A, SLC36A3, SLC36A2, SLC36A1, FAT2, MIR6499, SPARC, CLMAT3, ATOX1, LOC100652758, G3BP1, GLRA1, LINC01933, NMUR2, LINC01470, GRIA1, LINC01861, FAM114A2, MFAP3, GALNT10, MIR1294, SAP30L-AS1, SAP30L, HAND1, MIR3141, LARP1, MIR1303, FAXDC2, MIR378H, CNOT8, GEMIN5, MRPL22, KIF4B, SGCD, PPP1R2B, TIMD4, HAVCR1, HAVCR2, MED7, GARIN3, ITK, CYFIP2, FNDC9, NIPAL4-DT, NIPAL4, ADAM19, SOX30, C5orf52, THG1L, LSM11, CLINT1, LINC02227, EBF1, LINC02202, RNF145, LINC01932, UBLCP1, IL12B, LOC285626, LINC01845, LINC01847, ADRA1B, TTC1, PWWP2A, FABP6, CCNJL, C1QTNF2, FAM200C, SLU7, PTTG1, MIR3142HG, MIR3142, MIR146A, ATP10B, LINC02159, GABRB2, GABRA6, GABRA1, LINC01202, GABRG2, CCNG1, NUDCD2, HMMR, HMMR-AS1, MAT2B, LINC02143, LOC102546299, LINC01938, LINC01947, TENM2, LOC101927908, MIR12125, TENM2-AS1, WWC1, RARS1, FBLL1, PANK3, MIR103A1, MIR103B1, SLIT3, SLIT3-AS2, MIR218-2, SLIT3-AS1, MIR585, SPDL1, DOCK2, INSYN2B, MIR378E, FOXI1, LINC01187, C5orf58, LCP2, LOC100128059, LINC01366, KCNIP1, KCNMB1, KCNIP1-OT1, KCNIP1-AS1, GABRP, RANBP17, TLX3, SNORA70J, MIR3912, NPM1, FGF18, SMIM23, FBXW11, STK10, EFCAB9, UBTD2, LOC100288254, SH3PXD2B, LINC01944, NEURL1B, MIR5003, LOC101928093, DUSP1, ERGIC1, MIR10523, RPL26L1-AS1, RPL26L1, ATP6V0E1, SNORA74B, CREBRF, BNIP1, NKX2-5, MIR12118, STC2, MIR8056, LINC02995, BOD1, LINC01942, LINC01484, LINC01485, CPEB4, C5orf47, NSG2, LINC01411, MSX2, MIR4634, LINC01951, DRD1, SFXN1, HRH2, CPLX2, THOC3, THOC3-AS1, LOC100996385, FAM153B, LOC643201, SIMC1, KIAA1191, ARL10, MIR1271, NOP16, HIGD2A, CLTB, FAF2, RNF44, CDHR2, GPRIN1, SNCB, MIR4281, EIF4E1B, TSPAN17, LINC01574, UNC5A, HK3, UIMC1, ZNF346, FGFR4, NSD1, RAB24, PRELID1, MXD3, LMAN2, RGS14, SLC34A1, PFN3, F12, GRK6, PRR7-AS1, PRR7, DBN1, PDLIM7, DOK3, DDX41, FAM193B, TMED9, B4GALT7, LOC202181, FAM153A, LOC105377752, LOC728554, LOC100128340, PROP1, FAM153CP, N4BP3, RMND5B, NHP2, GMCL2, HNRNPAB, PHYKPL, COL23A1, CLK4, MSANTD5, ZNF354A, AACSP1, ZNF354B, ZFP2, ZNF454, GRM6, ZNF879, ZNF354C, ADAMTS2, LOC100289470, RUFY1, RUFY1-AS1, HNRNPH1, C5orf60, LOC100502572, LOC105377763, CBY3, CANX, MAML1, LTC4S, MGAT4B, MIR1229, SQSTM1, MRNIP, MRNIP-DT, TBC1D9B, RNF130, MIR340, RASGEF1C, MAPK9 | arr[GRCh38] 5q22.2q35.3(113,055,849_180,278,257)x3~4 |
| 2  Brain | Gain | 6 | p25.3 | p12.3 | 48,636 | LINC03066, DUSP22, IRF4, EXOC2, HUS1B, LOC101927691, LINC01622, FOXQ1, FOXF2, MIR6720, FOXCUT, FOXC1, GMDS, GMDS-DT, LINC01600, LINC02521, MYLK4, WRNIP1, SERPINB1, MIR4645, SERPINB9P1, SERPINB9-AS1, SERPINB9, SERPINB6, LINC01011, NQO2, HTATSF1P2, LOC101927759, RIPK1, BPHL, TUBB2A, LINC02525, TUBB2B, LOC100422781, PSMG4, SLC22A23, LOC643327, PXDC1, FAM50B, PRPF4B, FAM217A, TEX56P, ECI2, ECI2-DT, LOC102724096, LINC02533, KU-MEL-3, CDYL, CDYL-AS1, RPP40, LYRM4-AS1, LYRM4, PPP1R3G, MIR3691, FARS2, FARS2-AS1, LOC101927950, NRN1, F13A1, MIR7853, MIR5683, LY86-AS1, LY86, RREB1, SSR1, CAGE1, RIOK1, DSP-AS1, DSP, SNRNP48, BMP6, BLOC1S5-TXNDC5, TXNDC5, PIP5K1P1, BLOC1S5, EEF1E1-BLOC1S5, EEF1E1, SCARNA27, SLC35B3, LOC100506207, HULC, OFCC1, TFAP2A, TFAP2A-AS2, TFAP2A-AS1, LINC00518, MIR5689HG, MIR5689, LINC02522, GCNT2, C6orf52, PAK1IP1, TMEM14C, TMEM14B, MAK, GCM2, SYCP2L, LOC101928191, ELOVL2, ELOVL2-AS1, SMIM13, ERVFRD-1, NEDD9, TMEM170B, ADTRP, LOC101928253, HIVEP1, EDN1, LINC02530, PHACTR1, TBC1D7-LOC100130357, LOC100130357, TBC1D7, GFOD1, GFOD1-AS1, SIRT5, NOL7, RANBP9, MCUR1, RNF182, CD83, LINC01108, JARID2, JARID2-AS1, DTNBP1, LINC02543, MYLIP, MIR4639, GMPR, ATXN1, LOC127903862, ATXN1-AS1, STMND1, RBM24, CAP2, LOC101928491, FAM8A1, NUP153, NUP153-AS1, KIF13A, NHLRC1, TPMT, KDM1B, DEK, RNF144B, MIR548A1HG, MIR548A1, LOC101928519, LOC105374960, LNC-LBCS, ID4, MBOAT1, E2F3, E2F3-IT1, CDKAL1, LINC00581, SOX4, CASC15, NBAT1, PRL, HDGFL1, LINC03005, NRSN1, DCDC2, KAAG1, MRS2, GPLD1, ALDH5A1, KIAA0319, TDP2, ACOT13, C6orf62, LINC02828, GMNN, ARMH2, RIPOR2, CMAHP, LOC101928663, CARMIL1, SCGN, H2AC1, H2BC1, H2AC2P, SLC17A4, SLC17A1, SLC17A3, SLC17A2, TRIM38, H1-1, H3C1, H4C1, H4C2, H3C2, H2AC4, H2BC3, H3C3, H1-2, HFE-AS1, HFE, H4C3, H1-6, H2BC4, H2AC6, H1-4, H2BC5, H2BC6, H4C4, H3C4, H2AC7, H2BC7, H4C5, H2BC8, H2AC8, H3C6, H1-3, H4C6, H4C7, H3C7, H2BC9, H3C8, H2BC10, H4C8, BTN3A2, BTN2A2, BTN3A1, BTN2A3P, BTN3A3, BTN2A1, LOC285819, BTN1A1, HCG11, HMGN4, LOC105374988, ABT1, ZNF322, GUSBP2, LINC00240, LARRPM, H2BC11, H2AC11, H2BC12, H4C9, H2AC12, MIR3143, PRSS16, POM121L2, VN1R10P, ZNF204P, ZNF391, ZNF184, LINC01012, LOC100131289, H2BC13, H2AC13, H3C10, H2AC14, H2BC14, H4C11, H4C12, H2AC15, H2BC15, H2AC16, H1-5, H3C11, H4C13, H3C12, H2AC17, H2BC17, OR2B2, OR2B6, OR2B8P, ZNF165, ZSCAN12P1, ZSCAN16-AS1, ZSCAN16, ZKSCAN8, ZKSCAN8P1, TOB2P1, ZSCAN9, ZKSCAN4, NKAPL, ZSCAN26, PGBD1, ZSCAN31, ZKSCAN3, ZSCAN12, ZSCAN23, GPX6, GPX5, SCAND3, LINC00533, LINC01623, HCG14, TRIM27, LINC01556, HCG15, ZNF311, OR2W1-AS1, OR2W1, OR2B3, OR2J1, OR2J3, OR2J2, LINC03003, OR14J1, OR5V1, OR12D3, OR12D2, OR12D1, OR11A1, OR10C1, OR2H1, MAS1L, LINC02829, LINC01015, OR2I1P, UBD, SNORD32B, OR2H2, GABBR1, MOG, ZFP57, HLA-F, HLA-F-AS1, IFITM4P, HCG4, HLA-V, HLA-G, HCP5B, HLA-H, HCG4B, HLA-A, HCG9, POLR1HASP, HLA-J, POLR1H, PPP1R11, RNF39, TRIM31, TRIM31-AS1, TRIM40, TRIM10, TRIM15, TRIM26, HCG17, HLA-L, HCG18, TRIM39, TRIM39-RPP21, RPP21, HLA-E, LINC02569, GNL1, PRR3, ABCF1, MIR877, PPP1R10, MRPS18B, ATAT1, C6orf136, DHX16, PPP1R18, NRM, MDC1, MDC1-AS1, TUBB, FLOT1, IER3-AS1, IER3, HCG20, LINC00243, LINC02570, DDR1, MIR4640, GTF2H4, VARS2, SFTA2, MUCL3, HCG21, MUC21, MUC22, HCG22, C6orf15, PSORS1C1, CDSN, PSORS1C2, CCHCR1, TCF19, POU5F1, PSORS1C3, HCG27, HLA-C, LINC02571, HLA-B, MIR6891, MICA-AS1, MICA, LINC01149, HCP5, HCG26, MICB-DT, MICB, MCCD1, ATP6V1G2-DDX39B, DDX39B, SNORD117, SNORD84, DDX39B-AS1, ATP6V1G2, NFKBIL1, LOC100287329, LTA, TNF, LTB, LST1, NCR3, AIF1, PRRC2A, SNORA38, MIR6832, BAG6, APOM, C6orf47, GPANK1, CSNK2B, LY6G5B, LY6G5C, ABHD16A, MIR4646, LY6G6F, LY6G6F-LY6G6D, LY6G6E, LY6G6D, LY6G6C, MPIG6B, DDAH2, CLIC1, MSH5-SAPCD1, MSH5, SAPCD1, SAPCD1-AS1, VWA7, VARS1, LSM2, HSPA1L, HSPA1A, HSPA1B, SNHG32, SNORD48, SNORD52, NEU1, SLC44A4, EHMT2-AS1, EHMT2, C2, ZBTB12, C2-AS1, CFB, NELFE, MIR1236, SKIC2, DXO, STK19, C4A, CYP21A1P, TNXA, C4B, CYP21A2, TNXB, ATF6B, FKBPL, PRRT1, LOC100507547, PPT2, PPT2-EGFL8, EGFL8, AGPAT1, MIR6721, RNF5, MIR6833, AGER, PBX2, GPSM3, NOTCH4, TSBP1-AS1, TSBP1, HCG23, BTNL2, HLA-DRA, HLA-DRB5, HLA-DRB6, HLA-DRB1, HLA-DQA1, HLA-DQB1, HLA-DQB1-AS1, HLA-DQA2, MIR3135B, HLA-DQB2, HLA-DOB, TAP2, PSMB8, PSMB8-AS1, TAP1, PSMB9, LOC100294145, HLA-DMB, HLA-DMA, BRD2, HLA-DOA, HLA-DPA1, HLA-DPB1, HLA-DPB2, HCG24, COL11A2, RXRB, SLC39A7, HSD17B8, MIR219A1, RING1, HCG25, VPS52, RPS18, B3GALT4, WDR46, MIR6873, PFDN6, MIR6834, RGL2, TAPBP, ZBTB22, DAXX, SMIM40, KIFC1, PHF1, CUTA, SYNGAP1, SYNGAP1-AS1, MIR5004, ZBTB9, BAK1, GGNBP1, LINC00336, ITPR3, ITPR3-AS1, UQCC2, MIR3934, IP6K3, LEMD2, MLN, LINC01016, MIR7159, MIR1275, GRM4, HMGA1, MIR6835, SMIM29, NUDT3, RPS10-NUDT3, RPS10, PACSIN1, SPDEF, ILRUN, ILRUN-AS1, SNRPC, BLTP3A, TAF11, ANKS1A, TCP11, SCUBE3, ZNF76, DEF6, PPARD, FANCE, RPL10A, MIR7111, TEAD3, TULP1, FKBP5, MIR5690, LOC285847, ARMC12, CLPSL2, CLPSL1, CLPS, LHFPL5, SRPK1, SLC26A8, MAPK14, MAPK13, BRPF3, PNPLA1, BNIP5, ETV7, PXT1, KCTD20, STK38, SRSF3, MIR3925, PANDAR, CDKN1A, DINOL, RAB44, CPNE5, PPIL1, C6orf89, PI16, MTCH1, FGD2, PIM1, TMEM217, TMEM217B, TBC1D22B, RNF8, LOC107986531, CMTR1, CCDC167, LINC02520, MIR4462, MDGA1, ZFAND3, BTBD9, BTBD9-AS1, GLO1, DNAH8, DNAH8-AS1, GLP1R, MIR9983, SAYSD1, KCNK5, KCNK17, KCNK16, KIF6, DAAM2, DAAM2-AS1, MOCS1, LINC00951, TDRG1, LRFN2, LOC105379699, LOC101929555, UNC5CL, TSPO2, APOBEC2, OARD1, NFYA, ADCY10P1, TREML1, TREM2, TREML2, TREML3P, TREML4, TREML5P, TREM1, NCR2, LINC01276, FOXP4-AS1, FOXP4, MIR4641, MDFI, TFEB, MIR10398, PGC, FRS3, PRICKLE4, TOMM6, USP49, MED20, BYSL, CCND3, TAF8, C6orf132, GUCA1ANB, GUCA1ANB-GUCA1A, GUCA1A, GUCA1B, MRPS10, TRERF1, LOC107986596, UBR2, PRPH2, ATP6V0CP3, TBCC, BICRAL, LOC401261, RPL7L1, C6orf226, PTCRA, CNPY3, CNPY3-GNMT, GNMT, PEX6, PPP2R5D, MEA1, KLHDC3, RRP36, CUL7, MRPL2, KLC4, PTK7, SRF, CUL9, DNPH1, TTBK1, SLC22A7, CRIP3, ZNF318, ABCC10, MIR6780B, DLK2, TJAP1, LRRC73, YIPF3, POLR1C, XPO5, POLH, GTPBP2, MAD2L1BP, RSPH9, MRPS18A, VEGFA, LINC02537, LINC01512, SCIRT, LINC03040, MRPL14, TMEM63B, CAPN11, MYMX, SLC29A1, HSP90AB1, SLC35B2, MIR4647, NFKBIE, TMEM151B, TCTE1, AARS2, SPATS1, CDC5L, MIR4642, LOC105375075, SUPT3H, MIR586, RUNX2, CLIC5, ENPP4, ENPP5, RCAN2, LOC101926915, RCAN2-DT, CYP39A1, SLC25A27, TDRD6-AS1, TDRD6, PLA2G7, ANKRD66, MEP1A, ADGRF5, ADGRF5-AS1, ADGRF1, TNFRSF21, CD2AP, ADGRF2, ADGRF4, OPN5, PTCHD4 | arr[GRCh38] 6p25.3p12.3(156,975_48,793,406)x3 |
| 2  Brain | Gain | 6 | p12.2 | p12.1 | 4,642 | MIR206, LINCMD1, MIR133B, IL17A, IL17F, MCM3, PAQR8, EFHC1, TRAM2, TRAM2-AS1, LOC730101, TMEM14A, GSTA7P, GSTA2, GSTA1, GSTA5, GSTA3, GSTA4, RN7SK, CILK1, LOC128092246, FBXO9, LOC128031835, GCM1, ELOVL5, MIR5685, RPS16P5, GCLC-AS1, GCLC, KILH, LINC01564, KLHL31, LRRC1, LOC101927189, MLIP-IT1, MLIP, MLIP-AS1, TINAG, FAM83B, HCRTR2, GFRAL, HMGCLL1, BMP5, COL21A1, DST | arr[GRCh38] 6p12.2p12.1(52,090,402_56,732,340)x3 |
| 2  Brain | Gain | 6 | q12 | q13 | 5,581 | EYS, ZC3H11C, SLC25A51P1, LOC102723883, LINC02549, ADGRB3-DT, ADGRB3, LOC127898563, LMBRD1, COL19A1 | arr[GRCh38] 6q12q13(64,331,680_69,912,487)x3~4 |
| 2  Brain | Gain | 6 | q13 | q14.1 | 3,767 | KHDC3L, OOEP, OOEP-AS1, DDX43, CGAS, MTO1, EEF1A1, SLC17A5, CD109-AS1, CD109, LOC101928516, COL12A1, SNORD156, COX7A2, TMEM30A, TMEM30A-DT, FILIP1, LOC101928540, MIR4463, SENP6, MYO6, IMPG1, LINC02540 | arr[GRCh38] 6q13q14.1(73,356,349_77,122,984)x2~3 |
| 2  Brain | Gain | 7 | q31.32 | q31.33 | 3,677 | CADPS2, RNF133, RNF148, TAS2R16, SLC13A1, IQUB, NDUFA5, ASB15, ASB15-AS1, LMOD2, WASL, WASL-DT, HYAL6P, HYAL4, SPAM1, LOC105375483, TMEM229A, LOC101928211, SSU72L6, GPR37, LINC03043, POT1, POT1-AS1, LINC02830, LOC101928283, LOC101928254 | arr[GRCh38] 7q31.32q31.33(122,444,975_126,121,580)x3~4 |
| 2  Brain | Loss | 8 | p23.3 | p12 | 32,955 | FAM87A, FBXO25, TDRP, ERICH1, DLGAP2, LOC401442, LOC105377777, LOC286083, DLGAP2-AS1, CLN8-AS1, CLN8, MIR3674, MIR596, ARHGEF10, KBTBD11-AS1, KBTBD11-OT1, KBTBD11, MYOM2, MIR7160, LINC03021, LOC105377785, CSMD1, MCPH1-DT, MCPH1, ANGPT2, MCPH1-AS1, MIR8055, AGPAT5, MIR4659A, MIR4659B, XKR5, GS1-24F4.2, DEFB1, DEFA6, DEFA4, DEFA8P, DEFA9P, DEFA10P, DEFA1, DEFT1P, DEFA1B, DEFT1P2, DEFA3, DEFA11P, DEFA5, LOC101928095, LINC00965, FAM66B, DEFB109B, USP17L1, USP17L4, ZNF705G, DEFB4B, DEFB103B, SPAG11B, DEFB104B, DEFB106B, DEFB105B, DEFB107B, PRR23D1, FAM90A7, FAM90A22, FAM90A23, FAM90A14, FAM90A18, FAM90A16, FAM90A8, FAM90A17, FAM90A19, FAM90A9, FAM90A10, PRR23D2, DEFB107A, DEFB105A, DEFB106A, DEFB104A, SPAG11A, DEFB103A, DEFB4A, ZNF705B, FAM66E, USP17L8, USP17L3, MIR548I3, FAM85B, FAM86B3P, PRAG1, CLDN23, MFHAS1, ERI1, MIR4660, SNORD3I, PPP1R3B, PPP1R3B-DT, LOC101929128, LOC157273, TNKS, MIR597, MIR124-1HG, MIR124-1, MSRA, LINC03022, PRSS55, RP1L1, MIR4286, C8orf74, SOX7, LOC102723313, PINX1, MIR1322, PINX1-DT, XKR6, MIR598, LOC101929269, LINC00529, MTMR9, SLC35G5, TDH, TDH-AS1, FAM167A-AS1, FAM167A, BLK, LINC00208, GATA4, SNORA99, LINC02905, NEIL2, FDFT1, CTSB, DEFB136, DEFB135, DEFB134, DEFB130B, ZNF705D, FAM66D, LOC392196, USP17L7, USP17L2, FAM90A2P, FAM86B1, FAM85A, DEFB130A, FAM66A, LOC649352, DEFB109A, FAM90A25P, FAM86B2, FAM86B2-DT, LOC729732, MIR5692A2, LONRF1, MIR3926-1, MIR3926-2, LINC03019, LINC00681, TRMT9B, DLC1, C8orf48, LOC102725080, SGCZ, MIR383, TUSC3, MSR1, FGF20, MICU3, ZDHHC2, CNOT7, VPS37A, MTMR7, SLC7A2, PDGFRL, MTUS1, MIR548V, FGL1, PCM1, ASAH1, ASAH1-AS1, NAT1, NAT2, PSD3, LOC100128993, SH2D4A, CSGALNACT1, INTS10, LPL, SLC18A1, ATP6V1B2, LZTS1, LZTS1-AS1, SNORD3F, LINC03023, LINC02153, LINC03093, GFRA2, DOK2, XPO7, NPM2, FGF17, DMTN, FHIP2B, NUDT18, HR, HRURF, REEP4, LGI3, SFTPC, BMP1, PHYHIP, MIR320A, POLR3D, PIWIL2-DT, PIWIL2, SLC39A14, PPP3CC, SORBS3, PDLIM2, C8orf58, LOC107986876, CCAR2, BIN3, BIN3-IT1, EGR3, PEBP4, LOC101929237, RHOBTB2, TNFRSF10B, LOC286059, LOC254896, TNFRSF10C, TNFRSF10D, TNFRSF10A, TNFRSF10A-DT, CHMP7, R3HCC1, LOXL2, LOXL2-AS1, ENTPD4, SLC25A37, NKX3-1, NKX2-6, STC1, ADAM28, ADAM7-AS1, ADAMDEC1, ADAM7, ADAM7-AS2, NEFM, NEFL, MIR6841, DOCK5, MIR6876, LOC105379331, GNRH1, KCTD9, CDCA2, EBF2, PPP2R2A, SDAD1P1, BNIP3L, PNMA2, DPYSL2, ADRA1A, MIR548H4, STMN4, TRIM35, PTK2B, MIR6842, CHRNA2, EPHX2, CLU, MIR6843, SCARA3, MIR3622B, MIR3622A, CCDC25, ESCO2, PBK, SCARA5, MIR4287, NUGGC, ELP3, PNOC, ZNF395, FBXO16, FZD3, MIR4288, EXTL3-AS1, EXTL3, INTS9, HMBOX1, KIF13B, DUSP4, LINC00589, LINC02099, LOC101929470, LINC02209, MIR3148, SARAF, LEPROTL1, MBOAT4, DCTN6, RBPMS-AS1, RBPMS, GTF2E2, SMIM18, GSR, UBXN8, PPP2CB, TEX15, PURG, WRN, NRG1, NRG1-IT1, NRG1-IT3, LOC128092250 | arr[GRCh38] 8p23.3p12(260,291_33,215,131)x1 |
| 2  Brain | Gain | 8 | p12 | q13.3 | 36,860 | FUT10, MAK16, TTI2, SNORD13, RNF122, DUSP26, LINC01288, UNC5D, LOC101929550, KCNU1, LINC01605, ZNF703, LOC101929622, LOC102723701, ERLIN2, LOC728024, PLPBP, ADGRA2, BRF2, RAB11FIP1, GOT1L1, ADRB3, EIF4EBP1, ASH2L, STAR, LSM1, BAG4, DDHD2, PLPP5, NSD3, LETM2, FGFR1, LINC03042, RNF5P1, TACC1, PLEKHA2, HTRA4, TM2D2, ADAM9, SNORD38D, ADAM32, ADAM5, ADAM3A, LOC100130964, ADAM18, ADAM2, IDO1, IDO2, TCIM, SIRLNT, ZMAT4, SFRP1, MIR548AO, SNORD65B, GOLGA7, GINS4, GPAT4-AS1, GPAT4, NKX6-3, ANK1, MIR486-1, MIR486-2, KAT6A, KAT6A-AS1, AP3M2, PLAT, IKBKB-DT, IKBKB, POLB, DKK4, VDAC3, SLC20A2, SMIM19, CHRNB3, CHRNA6, THAP1, RNF170, MIR4469, HOOK3, FNTA, POMK, HGSNAT, POTEA, ASNSP1, LINC00293, LOC100287846, SPIDR, CEBPD, PRKDC, MCM4, UBE2V2, LINC02947, LOC101929268, LINC03054, CLXN, SNAI2, PPDPFL, LOC100507464, SNTG1, PXDNL, PCMTD1, ST18, LOC101929341, ALKAL1, RB1CC1, NPBWR1, OPRK1, LINC02984, ATP6V1H, RGS20, TCEA1, LYPLA1, MRPL15, RNU105C, SOX17, RP1, XKR4, SBF1P1, XKR4-AS1, TMEM68, TGS1, LYN, SNORA1B, RPS20, SNORD54, CERNA3, MOS, PLAG1, CHCHD7, SDR16C5, SDR16C6P, PENK, PENK-AS1, LINC00968, BPNT2, LINC01606, LOC286177, LINC00588, LOC101929488, LINC03018, LINC01602, FAM110B, LOC101929528, UBXN2B, CYP7A1, SDCBP, NSMAF, TOX, TOX-DT, CA8, LINC01301, RAB2A, CHD7, LOC105375938, LOC100130298, CLVS1, ASPH, MIR4470, LINC02155, NKAIN3, GGH, TTPA, YTHDF3-DT, YTHDF3, LOC102724612, LINC01289, LINC01414, MIR124-2HG, MIR124-2, BHLHE22-AS1, BHLHE22, CYP7B1, LINC00251, LINC01299, ARMC1, MTFR1, PDE7A, DNAJC5B, TRIM55, CRH, LINC00967, RRS1-DT, RRS1, ADHFE1, VXN, MYBL1, VCPIP1, C8orf44, C8orf44-SGK3, SGK3, PTTG3P, MCMDC2, SNHG6, SNORD87, TCF24, PPP1R42, COPS5, CSPP1, ARFGEF1, ARFGEF1-DT, CPA6, PREX2, C8orf34-AS1, C8orf34, LINC01592, LINC01603, SULF1, SLCO5A1, PRDM14 | arr[GRCh38] 8p12q13.3(33,216,925_70,077,064)x3 |
| 2  Brain | Gain | 8 | q13.3 | q21.3 | 19,572 | EYA1, MSC, MSC-AS1, TRPA1, LOC392232, KCNB2, LOC101926908, TERF1, SBSPON, C8orf89, RPL7, RDH10, RDH10-AS1, STAU2-AS1, STAU2, UBE2W, LINC01617, ELOC, TMEM70, LY96, JPH1, GDAP1, MIR5681A, MIR5681B, MIR2052HG, LINC03071, MIR2052, PI15, CRISPLD1, CASC9, HNF4G, LINC01109, LINC01111, ZFHX4-AS1, ZFHX4, MIR3149, PEX2, LOC102724874, PKIA, PKIA-AS1, ZC2HC1A, C4orf46P3, IL7, LINC02605, MIR12123, STMN2, HEY1, LINC01607, LOC101927040, MRPS28, TPD52-MRPS28, TPD52, MIR5708, ZBTB10, ZNF704, PAG1, FABP5, PMP2, FABP9, FABP4, FABP12, IMPA1P1, IMPA1, SLC10A5, ZFAND1, CHMP4C, SNX16, LINC02235, LINC02839, LOC101927141, LINC01419, RALYL, LRRCC1, E2F5-DT, E2F5, RBIS, CA13, CA1, CA3, CA3-AS1, CA2, REXO1L2P, PSKH2, ATP6V0D2, SLC7A13, WWP1, RMDN1, CPNE3, CNGB3, CNBD1, DCAF4L2, MMP16, RIPK2-DT, RIPK2, OSGIN2, NBN, DECR1, CALB1, LINC00534, LINC01030, TMEM64, NECAB1 | arr[GRCh38] 8q13.3q21.3(71,303,116_90,874,797)x3 |
| 2  Brain | Gain | 8 | q22.1 | q23.1 | 15,338 | CIBAR1-DT, CIBAR1, RBM12B, RBM12B-AS1, RBM12B-DT, TMEM67, MIR378D2HG, MIR378D2, PDP1, CDH17, GEM, RAD54B, FSBP, VIRMA, VIRMA-DT, LINC02894, ESRP1, DPY19L4, INTS8, CCNE2, NDUFAF6, TP53INP1, MIR3150BHG, MIR3150B, MIR3150A, PLEKHF2, LINC01298, CFAP418, CFAP418-AS1, SRSF3P2, GDF6, UQCRB, UQCRB-AS1, MTERF3, PTDSS1, LOC102724804, SDC2, CPQ, LOC101927066, TSPYL5, SNORD3H, MTDH, LAPTM4B, MATN2, RPL30, SNORA72, ERICH5, RIDA, POP1, NIPAL2, MIR9903, KCNS2, STK3, OSR2, VPS13B, MIR599, MIR875, COX6C, RGS22, SNORD77B, FBXO43, POLR2K, SPAG1, RNF19A, MIR4471, ANKRD46, SNX31, PABPC1, MIR7705, YWHAZ, FLJ42969, ZNNT1, ZNF706, LINC02844, NACA4P, GRHL2, NCALD, LOC104054148, MIR5680, RRM2B, UBR5-DT, UBR5, LOC105375683, ODF1, KLF10, LOC101927245, GASAL1, AZIN1, MAILR, ATP6V1C1, LINC01181, BAALC-AS2, BAALC, MIR3151, BAALC-AS1, LOC105369147, FZD6, SNORD173, CTHRC1, SLC25A32, DCAF13, LOC105375690, RIMS2, DCSTAMP, DPYS, MIR548A3, LRP12, ZFPM2, ZFPM2-AS1, OXR1, ABRA, ANGPT1, RSPO2, EIF3E, EMC2, TMEM74 | arr[GRCh38] 8q22.1q23.1(93,476,554_108,814,558)x3 |
| 2  Brain | Gain | 8 | q23.3 | q24.22 | 19,589 | TRPS1, LINC00536, EIF3H, LOC105375713, UTP23, RAD21, RAD21-AS1, MIR3610, AARD, SLC30A8, MED30, EXT1, SNORD168, SAMD12, SAMD12-AS1, TNFRSF11B, COLEC10, LOC101927513, MAL2, MAL2-AS1, MIR548AZ, CCN3, ENPP2, TAF2, DSCC1, DEPTOR, COL14A1, MRPL13, MTBP, SNTB1, LOC101927543, HAS2, HAS2-AS1, LINC02855, SMILR, LINC01151, ZHX2, DERL1, TBC1D31, FAM83A, FAM83A-AS1, MIR4663, C8orf76, ZHX1-C8orf76, ZHX1, ATAD2, MIR548AA1, MIR548D1, NTAQ1, FBXO32, KLHL38, ANXA13, FAM91A1, FER1L6, FER1L6-AS1, FER1L6-AS2, LOC101927588, TMEM65, TRMT12, RNF139-DT, RNF139, TATDN1, MIR6844, NDUFB9, MTSS1, MIR4662B, MIR4662A, LINC00964, ZNF572, SQLE-DT, SQLE, WASHC5, WASHC5-AS1, NSMCE2, TRIB1, LINC00861, LOC101927657, LRATD2, PCAT1, PCAT2, PRNCR1, CASC19, CCAT1, CASC21, CASC8, CCAT2, POU5F1B, CASC11, MYC, PVT1, MIR1204, LINC02912, MIR1205, MIR1206, MIR1207, MIR1208, LINC00824, LINC00976, LINC00977, CCDC26, MIR3686, GSDMC, CYRIB, MIR5194, ASAP1, ASAP1-IT2, ASAP1-IT1, ADCY8, EFR3A, OC90, HHLA1, KCNQ3, HPYR1, DNAAF11, TMEM71, PHF20L1, TG, SLA, MIR7848, PTCSC1 | arr[GRCh38] 8q23.3q24.22(113,584,776_133,173,279)x3 |
| 2  Brain | cn-LOH | 8 | q24.22 | q24.3 | 11,916 | CCN4, NDRG1, ST3GAL1, LINC03024, LOC101927798, LOC101927822, ZFAT, ZFAT-AS1, MIR30B, MIR30D, NCRNA00250, LOC101927845, LINC01591, KHDRBS3, LINC02055, LOC101927915, LOC401478, FAM135B, COL22A1, KCNK9, TRAPPC9, C8orf17, PEG13, CHRAC1, AGO2, ERICD, PTK2, MIR151A, DENND3-AS1, DENND3, SLC45A4, SLC45A4-AS1, LINC01300, GPR20, PTP4A3, MROH5, C8orf90, MIR1302-7, MIR4472-1, LINC00051, TSNARE1, ADGRB1, ARC, LOC101928087, JRK, PSCA, LY6K, LNCOC1, THEM6, SLURP1, LYPD2, SLURP2, LYNX1-SLURP2, LYNX1, LY6D, GML, CYP11B1, CYP11B2, LY6E-DT, CDC42P3, LY6E, LINC02904, LY6S, LY6L, LY6H, GPIHBP1, ZFP41, GLI4, MINCR, ZNF696, TOP1MT, RHPN1-AS1, RHPN1, MAFA-AS1, MAFA, ZC3H3, SNORD149, GSDMD, LOC100310756, MROH6, NAPRT, EEF1D, TIGD5, PYCR3, GFUS, ZNF623, ZNF707, LINC02878, CCDC166, LOC101928160, MAPK15, FAM83H, MIR4664, IQANK1, LOC105375800, SCRIB, MIR937, PUF60, NRBP2, MIR6845, EPPK1, PLEC, MIR661, PARP10, GRINA, SPATC1, SMPD5, OPLAH, MIR6846, EXOSC4, MIR6847, GPAA1, CYC1, SHARPIN, MAF1, WDR97, HGH1, MROH1, BOP1, MIR7112, SCX, HSF1, DGAT1, MIR6848, SCRT1, TMEM249, FBXL6, SLC52A2, LOC101928902, ADCK5, CPSF1, MIR939, MIR1234, MIR6849, SLC39A4, VPS28, TONSL, TONSL-AS1, MIR6893, ZFTRAF1, TMEM276-ZFTRAF1, MIR10400, TMEM276, KIFC2, FOXH1, PPP1R16A, GPT, MFSD3, RECQL4, LRRC14, LRRC24, C8orf82, ARHGAP39, ZNF251, ZNF34, RPL8, MIR6850, ZNF517, LOC100130027, ZNF7, COMMD5, ZNF250, ZNF16, ZNF252P, TMED10P1, ZNF252P-AS1, C8orf33 | arr[GRCh38] 8q24.22q24.3(133,151,196_145,067,348)x4 hmz |
| 2  Brain | Gain | 9 | p24.3 | p24.2 | 3,854 | KANK1, DMRT1, DMRT3, LINC01230, DMRT2, SMARCA2, VLDLR-AS1, VLDLR, KCNV2, PUM3, LINC01231, RFX3, RFX3-DT, GLIS3, GLIS3-AS1, SLC1A1 | arr[GRCh38] 9p24.3p24.2(676,042_4,529,860)x2~3 |
| 2  Brain | Gain | 9 | p24.1 | p22.3 | 7,540 | PTPRD, LOC105375972, PTPRD-DT, TYRP1, LURAP1L-AS1, LURAP1L, SNORD137, MPDZ, LINC01235, LINC00583, NFIB, ZDHHC21, CER1, FREM1, LOC389705, TTC39B, SNAPC3, PSIP1, CCDC171, LINC03041, BNC2 | arr[GRCh38] 9p24.1p22.3(8,943,485_16,483,225)x3~4 |
| 2  Brain | Gain | 9 | p22.2 | p21.1 | 13,052 | CNTLN, SH3GL2, ADAMTSL1, MIR3152, SAXO1, RRAGA, HAUS6, SCARNA8, PLIN2, DENND4C, RPS6, ACER2, SLC24A2, MLLT3, MIR4473, MIR4474, FOCAD, FOCAD-AS1, MIR491, SNORA30B, HACD4, IFNB1, IFNW1, IFNA21, IFNA4, IFNA7, IFNA10, IFNA16, IFNA17, IFNA14, IFNA22P, IFNA5, KLHL9, IFNA6, IFNA13, IFNA2, IFNA8, IFNA1, MIR31HG, IFNE, MIR31, MTAP, CDKN2A-DT, CDKN2A, CDKN2B-AS1, CDKN2B, DMRTA1, LINC01239, LOC101929563, ELAVL2, IZUMO3, TUSC1, LINC01241, LOC100506422, CAAP1, PLAA, IFT74, IFT74-AS1, LRRC19, TEK, LINC00032, EQTN, MOB3B, IFNK, C9orf72, LINGO2, MIR876, MIR873 | arr[GRCh38] 9p22.2p21.1(17,074,880_30,126,793)x3~4 |
| 2  Brain | cn-LOH | 9 | p21.1 | p13.2 | 7,267 | LINC01242, LINC01243, ACO1, RIGI, TOPORS, SMIM27, NDUFB6, TAF1L, TMEM215, APTX, DNAJA1, SMU1, B4GALT1, MIR12117, B4GALT1-AS1, SPINK4, BAG1, CHMP5, NFX1, AQP7, AQP3, NOL6, MIR6851, SUGT1P1, ANKRD18B, ANXA2P2, PTENP1, PTENP1-AS, UBE2R2-AS1, PRSS3, UBE2R2, UBAP2, SNORD121B, SNORD121A, DCAF12, UBAP1, KIF24, NUDT2, MYORG, C9orf24, FAM219A, DNAI1, ENHO, CNTFR, CNTFR-AS1, RPP25L, DCTN3, ARID3C, SIGMAR1, GALT, IL11RA, CCL27, LOC730098, CCL19, CCL21, SPATA31F1, SPATA31F2P, SPATA31F3, PHF24, DNAJB5-DT, DNAJB5, C9orf131, VCP, FANCG, PIGO, STOML2, ATOSB, UNC13B, ATP8B5P, RUSC2, FAM166B, TESK1, MIR4667, CD72, LOC101926948, SIT1, RMRP, CCDC107, ARHGEF39, CA9, TPM2, TLN1, MIR6852, CREB3, MIR6853, GBA2, RGP1, MSMP, NPR2, SPAG8, HINT2, FAM221B, TMEM8B, OR13J1, HRCT1, SPAAR, OR2S2, RECK, GLIPR2, CCIN, CLTA, GNE, RNF38, MELK, MIR4475, PAX5, MIR4540, MIR4476, EBLN3P, ZCCHC7 | arr[GRCh38] 9p21.1p13.2(30,097,790_37,364,678)x3 hmz |
| 2  Brain | Gain | 9 | q21.11 | q22.2 | 22,581 | PGM5, TMEM252, LINC01506, PIP5K1B, PABIR1, LOC101927069, PRKACG, FXN, TJP2, BANCR, ENTREP1, APBA1, PTAR1, CFAP95-DT, CFAP95, MAMDC2, MAMDC2-AS1, SMC5-DT, SMC5, KLF9, TRPM3, MIR204, CEMIP2, ABHD17B, C9orf85, C9orf57, GDA, LINC01504, ZFAND5, TMC1, LINC01474, ALDH1A1, ANXA1, LOC101927358, RORB-AS1, RORB, TRPM6, C9orf40, CARNMT1-AS1, CARNMT1, NMRK1, OSTF1, PCSK5, RFK, GCNT1, RPSAP9, PRUNE2, PCA3, FOXB2, VPS13A-AS1, VPS13A, GNA14, GNA14-AS1, GNAQ, CEP78, PSAT1, LOC101927450, LNCARSR, TLE4, LINC01507, TLE1, TLE1-DT, SPATA31D5P, SPATA31D4, SPATA31D3, SPATA31D1, RASEF, FRMD3-AS1, FRMD3, IDNK, UBQLN1, UBQLN1-AS1, GKAP1, KIF27, QNG1, HNRNPK, MIR7-1, RMI1, LOC101927575, SLC28A3, NTRK2, AGTPBP1, LOC389765, NAA35, GOLM1, LOC101927623, C9orf153, ISCA1, TUT7, GAS1, GAS1RR, LINC02893, NFYCP2, LINC02872, DAPK1, DAPK1-IT1, CTSL, CTSL3P, CTSLP8, NPAP1P9, LOC497256, SPATA31E1, SPATA31C1, LOC122394732, CDK20, LINC03026, SPATA31C2, SPIN1, NXNL2, LINC02843, MIR4289, S1PR3, SHC3, CKS2, MIR3153, SECISBP2, SEMA4D, GADD45G, LINC03062, LOC101927847, MIR4290HG, MIR4290, LINC01508, LINC01501, DIRAS2, SYK, LINC02957, LOC100129316, LINC02937 | arr[GRCh38] 9q21.11q22.2(68,506,370_91,087,748)x3~4 |
| 2  Brain | Gain | 9 | q22.31 | q31.1 | 10,027 | PHF2, MIR548AU, MIR4291, BARX1, BARX1-DT, PTPDC1, LOC107987099, MIRLET7A1HG, MIRLET7A1, MIRLET7F1, LINC02603, MIRLET7D, ZNF169, NUTM2F, LOC100132077, MFSD14B, PCAT7, FBP2, FBP1, AOPEP, MIR2278, LOC101928119, MIR6081, MIR23B, MIR27B, MIR3074, MIR24-1, FANCC, PTCH1, LOC100507346, ERCC6L2-AS1, ERCC6L2, LINC00092, LOC158435, LOC158434, HSD17B3, SLC35D2-HSD17B3, HSD17B3-AS1, SLC35D2, ZNF367, HABP4, CDC14B, PRXL2C, LOC441455, ZNF510, ZNF782, LOC100132781, PTMAP11, NUTM2G, MFSD14CP, CTSV, GAS2L1P2, ANKRD18CP, SUGT1P4-STRA6LP, SUGT1P4-STRA6LP-CCDC180, CCDC180, MIR1302-8, LOC286359, TDRD7, TMOD1, TSTD2, NCBP1, XPA, PTCSC2, FOXE1, TRMO, HEMGN, ANP32B, NANS, TRIM14, CORO2A, TBC1D2, MIR6854, GABBR2, ANKS6, GALNT12, ADIPINT, COL15A1, TGFBR1, ALG2, SEC61B, NAMA, LOC101928438, NR4A3, STX17-DT, STX17, ERP44, INVS, TEX10, MSANTD3, MSANTD3-TMEFF1, TMEFF1, CAVIN4, PLPPR1, BAAT, MRPL50, ZNF189, ALDOB, TMEM246-AS1, PGAP4, RNF20, GRIN3A, PPP3R2, LINC00587, CYLC2, LINC01492 | arr[GRCh38] 9q22.31q31.1(93,578,189_103,605,671)x3~4 |
| 2  Brain | Gain | 9 | q31.2 | q34.3 | 32,577 | FSD1L, FKTN-AS1, FKTN, TAL2, TMEM38B, MIR8081, LINC01505, ZNF462, LOC340512, RAD23B, LINC01509, KLF4, ACTL7B, ACTL7A, ELP1, ABITRAM, CTNNAL1, TMEM245, MIR32, FRRS1L, EPB41L4B, PTPN3, MIR3927, PALM2AKAP2, LOC107987013, C9orf152, TXN, TXNDC8, SVEP1, MUSK, LPAR1, MIR7702, OR2K2, ECPAS, ZNF483, PTGR1, LRRC37A5P, DNAJC25, DNAJC25-GNG10, GNG10, SHOC1, UGCG, MIR4668, SUSD1, PTBP3, HSDL2, HSDL2-AS1, KIAA1958, INIP, SNX30, SLC46A2, ZNF883, ZFP37, FAM225B, FAM225A, SLC31A2, FKBP15, SLC31A1, CDC26, PRPF4, RNF183, WDR31, BSPRY, HDHD3, ALAD, POLE3, C9orf43, RGS3, ZNF618, AMBP, KIF12, COL27A1, MIR455, ORM1, ORM2, AKNA, WHRN, ATP6V1G1, TMEM268, TEX53, TEX48, TNFSF15, TNFSF8, TNC, LOC101928748, DELEC1, LOC101928775, LINC00474, PAPPA, PAPPA-AS2, PAPPA-AS1, ASTN2, ASTN2-AS1, LOC107987014, TRIM32, SNORA70C, LOC101928797, TLR4, LINC02578, BRINP1, LINC01613, MIR147A, CDK5RAP2, MEGF9, FBXW2, B3GALT9, PSMD5, CUTALP, PHF19, TRAF1, C5-OT1, C5, CNTRL, RAB14, GSN, GSN-AS1, STOM, GGTA1, DAB2IP, TTLL11, MIR4478, NDUFA8, MORN5, LHX6, RBM18, MRRF, PTGS1, OR1J1, OR1J2, OR1J4, OR1N1, OR1N2, OR1L8, OR1Q1, OR1B1, OR1L1, OR1L3, OR1L4, OR1L6, OR5C1, OR1K1, PDCL, RC3H2, SNORD90, ZBTB6, ZBTB26, RABGAP1, GPR21, MIR600HG, MIR600, STRBP, CRB2, DENND1A, MIR601, MIR7150, LHX2-AS1, LHX2, NEK6, LOC613206, PSMB7, LOC100129034, ADGRD2, NR5A1, NR6A1, MIR181A2HG, MIR181A2, MIR181B2, OLFML2A, WDR38, RPL35, ARPC5L, GOLGA1, SCAI, PPP6C, LOC105376271, RABEPK, HSPA5, GAPVD1, MAPKAP1, PBX3-DT, PBX3, LOC101929116, MVB12B, NRON, LMX1B-DT, LMX1B, ZBTB43, ZBTB34, RALGPS1, ANGPTL2, GARNL3, SLC2A8, ZNF79, RPL12, SNORA65, LRSAM1, NIBAN2, STXBP1, MIR3911, CFAP157, PTRH1, TTC16, TOR2A, SH2D3C, MIR3960, MIR2861, CDK9, FPGS, ENG, LOC102723566, AK1, ST6GALNAC4-ST6GALNAC6-AK1, MIR4672, ST6GALNAC6, ST6GALNAC4, PIP5KL1, DPM2, EEIG1, NAIF1, SLC25A25, SLC25A25-AS1, PTGES2, PTGES2-AS1, LCN2, BBLN, CIZ1, DNM1, MIR199B, MIR3154, GOLGA2, SWI5, TRUB2, COQ4, SLC27A4, URM1, MIR219A2, MIR219B, CERCAM, ODF2, ODF2-AS1, GLE1, SPTAN1, DYNC2I2, SET, PKN3, ZDHHC12, ZDHHC12-DT, ZER1, TBC1D13, ENDOG, SPOUT1, KYAT1-SPOUT1, KYAT1, LRRC8A, PHYHD1, DOLK, NUP188, SH3GLB2, MIGA2, DOLPP1, CRAT, PTPA, IER5L, LINC02975, LINC02913, LINC01503, LINC00963, NTMT1, C9orf50, ASB6, PRRX2, PRRX2-AS1, PTGES, TOR1B, TOR1A, C9orf78, USP20, MIR6855, FNBP1, GPR107, GPRACR, NCS1, MIR12126, HMCN2, ASS1, LOC100272217, FUBP3, MIR6856, PRDM12, EXOSC2, ABL1, LOC128092248, QRFP, FIBCD1, LAMC3, AIF1L, NUP214, FAM78A, PLPP7, PRRC2B, SNORD62A, SNORD62B, POMT1, UCK1, PRRT1B, RAPGEF1, MED27, NTNG2, SETX, TTF1, CFAP77, BARHL1, DDX31, GTF3C4, AK8, SPACA9, TSC1, GFI1B, MIR548AW, SNORD141A, LOC105376306, GTF3C5, MIR6877, CEL, CELP, RALGDS, GBGT1, OBP2B, ABO, SURF6, MED22, RPL7A, SNORD24, SNORD36B, SNORD36A, SNORD36C, SURF1, SURF2, SURF4, STKLD1, REXO4, ADAMTS13, CACFD1, SLC2A6, MYMK, ADAMTSL2, FAM163B, DBH, DBH-AS1, SARDH, VAV2, BRD3OS, BRD3, LOC100130548, WDR5, RNU6ATAC, LINC02247, RXRA, MIR4669, COL5A1, COL5A1-AS1, LOC101448202, MIR3689C, MIR3689A, MIR3689D1, MIR3689B, MIR3689D2, MIR3689E, MIR3689F, FCN2, FCN1, OLFM1, LOC401557, LINC02907, PPP1R26-AS1, PPP1R26, PIERCE1, MRPS2, LOC101928525, LCN1, OBP2A, PAEP, LINC01502, LOC102723971, GLT6D1, LCN9, SOHLH1, KCNT1, CAMSAP1, UBAC1, NACC2, TMEM250, LOC107987142, LHX3, QSOX2, CCDC187, DKFZP434A062, GPSM1, DNLZ, CARD9, SNAPC4, ENTR1, PMPCA, INPP5E, SEC16A, C9orf163, NOTCH1, MIR4673, MIR4674, NALT1, LINC01451, HSPC324, EGFL7, MIR126, AGPAT2, DIPK1B, SNHG7, SNORA17B, SNORA17A, LCN10, LCN6, LOC100128593, MIR6722, LCN8, LCN15, TMEM141, CCDC183, CCDC183-AS1, RABL6, MIR4292, AJM1, PHPT1, MAMDC4, EDF1, TRAF2, MIR4479, FBXW5, C8G, LCN12, LINC02692, PTGDS, LCNL1, PAXX, CLIC3, ABCA2, LINC02908, FUT7, NPDC1, ENTPD2, SAPCD2, UAP1L1, MAN1B1-DT, MAN1B1, DPP7, GRIN1, LRRC26, MIR3621, TMEM210, ANAPC2, SSNA1, TPRN, TMEM203, NDOR1, LOC122513141, RNF208, CYSRT1, RNF224, SLC34A3, TUBB4B, FAM166A, STPG3-AS1, STPG3, NELFB, TOR4A, NRARP, EXD3, NOXA1, ENTPD8, NSMF, MIR7114, PNPLA7, MRPL41, DPH7, ZMYND19, ARRDC1, ARRDC1-AS1, EHMT1, LOC651337, MIR602, LOC100133077, CACNA1B, CACNA1B-AS1, LOC101928786 | arr[GRCh38] 9q31.2q34.3(105,548,469_138,125,937)x3~4 |
| 2  Brain | cn-LOH | 10 | q22.1 | q22.3 | 6,123 | ASCC1, ANAPC16, DDIT4, DNAJB12, MICU1, MCU, MIR4676, OIT3, PLA2G12B, P4HA1, NUDT13, SNORA11F, ECD, FAM149B1, DNAJC9, DNAJC9-AS1, MRPS16, CFAP70, ANXA7, MSS51, PPP3CB, PPP3CB-AS1, USP54, MYOZ1, SYNPO2L, BMS1P4-AGAP5, AGAP5, BMS1P4, GLUD1P3, SEC24C, FUT11, CHCHD1, ZSWIM8, ZSWIM8-AS1, NDST2-ZSWIM8-AS1, NDST2, CAMK2G, C10orf55, PLAU, VCL, AP3M1, ADK, LOC102723439, KAT6B, SNORD172, DUSP29, DUSP13, LOC128854680, SAMD8, VDAC2, COMTD1, ZNF503, ZNF503-AS1, ZNF503-AS2, LOC101929234, LRMDA, MIR606, LOC105378367, KCNMA1, KCNMA1-AS1, KCNMA1-AS2, KCNMA1-AS3, DLG5, DLG5-AS1, POLR3A, RPS24 | arr[GRCh38] 10q22.1q22.3(72,092,798_78,215,437)x3 hmz |
| 2  Brain | Gain | 12 | p13.33 | p13.2 | 11,024 | IQSEC3, IQSEC3-AS2, LOC574538, IQSEC3-AS1, SLC6A12, SLC6A12-AS1, SLC6A13, LOC102723544, KDM5A, CCDC77, B4GALNT3, NINJ2, LOC105369595, NINJ2-AS1, WNK1, RAD52, ERC1, LINC00942, LOC107984507, FBXL14, WNT5B, MIR3649, ADIPOR2, CACNA2D4, LRTM2, LINC00940, DCP1B, CACNA1C-IT2, CACNA1C, CACNA1C-AS4, CACNA1C-IT3, CACNA1C-AS2, CACNA1C-AS1, ITFG2-AS1, FKBP4, ITFG2, NRIP2, TEX52, FOXM1, RHNO1, TULP3, TEAD4, TSPAN9, LOC100128253, LINC02417, PRMT8, THCAT155, CRACR2A, PARP11, PARP11-AS1, CCND2-AS1, CCND2, TIGAR, FGF23, FGF6, C12orf4, RAD51AP1, DYRK4, AKAP3, NDUFA9, GAU1, GALNT8, KCNA6, KCNA1, KCNA5, LINC02443, NTF3, ANO2, VWF, SNORA120, CD9, PLEKHG6, TNFRSF1A, SCNN1A, LTBR, CD27-AS1, CD27, TAPBPL, VAMP1, MRPL51, NCAPD2, SCARNA10, GAPDH, IFFO1, NOP2, CHD4, SCARNA11, LPAR5, ACRBP, ING4, ZNF384, PIANP, COPS7A, MLF2, PTMS, LAG3, CD4, GPR162, P3H3, GNB3, CDCA3, USP5, TPI1, SPSB2, LOC105369632, RPL13P5, DSTNP2, LRRC23, ENO2, ATN1, C12orf57, RNU7-1, PTPN6, MIR200CHG, MIR200C, MIR141, PHB2, SCARNA12, EMG1, LPCAT3, C1S, C1R, C1RL, C1RL-AS1, RBP5, CLSTN3, PEX5, ACSM4, CD163L1, CD163, APOBEC1, GDF3, DPPA3, CLEC4C, NANOGNB, NANOG, SLC2A14, SLC2A3, FOXJ2, C3AR1, NECAP1, CLEC4A, POU5F1P3, ZNF705A, FAM66C, FAM90A1, FAM86FP, LINC02449, LINC00937, CLEC6A, CLEC4D, CLEC4E, AICDA, MFAP5, RIMKLB, A2ML1, PHC1, M6PR, KLRG1, LINC00612, A2M-AS1, A2M, PZP, A2MP1, MIR1244-3, LINC00987, LOC642846, LINC02367, LOC101928030, LOC728715, DDX12P, LOC408186, KLRB1, LOC374443, LOC105369728, CLEC2D, CLECL1P, CD69, KLRF1, CLEC2B, KLRF2, CLEC2A, LINC02470, CLEC12A-AS1, CLEC12A, CLEC1B, CLEC12B, LOC102724020, CLEC9A, CLEC1A, CLEC7A, OLR1, TMEM52B, GABARAPL1, GABARAPL1-AS1, KLRD1, LINC02617, LINC02598, KLRK1-AS1, KLRK1, KLRC4-KLRK1, KLRC4, KLRC3, KLRC2, KLRC1, EIF2S3B, LINC02446, KLRA1P, MAGOHB, STYK1, YBX3, LINC02366, TAS2R7, TAS2R8, TAS2R9, TAS2R10, PRR4, PRH1-PRR4, PRH1, TAS2R13, PRH2, TAS2R14, PRH1-TAS2R14, TAS2R50, TAS2R20, TAS2R19, TAS2R31, TAS2R46 | arr[GRCh38] 12p13.33p13.2(64,621_11,088,676)x4 |
| 2  Brain | Gain | 13 | q11 | q13.1 | 13,262 | ANKRD20A9P, LINC00408, LINC00442, LOC107984132, TUBA3C, LOC101928697, ANKRD26P3, LINC00421, TPTE2, TPTE2-AS1, MPHOSPH8, PSPC1, ZMYM5, ZMYM2, LINC01072, GJA3, GJB2, GJB6, CRYL1, MIR4499, IFT88, IL17D, EEF1AKMT1, XPO4, LINC00367, LATS2, SAP18, SKA3, MRPL57, LINC01046, LOC101928764, MIPEPP3, LINC00539, GRK6P1, ZDHHC20, MICU2, FGF9, LINC00424, LINC00540, LINC00621, BASP1P1, SGCG, SACS, SACS-AS1, LINC00327, TNFRSF19, MIPEP, PCOTH, C1QTNF9B, ANKRD20A19P, SPATA13, MIR2276, SPATA13-AS1, C1QTNF9, LINC00566, PARP4, LOC105370295, TPTE2P6, ATP12A, RNF17, CENPJ, TPTE2P1, PABPC3, AMER2, LINC00463, LINC01053, MTMR6, NUP58, ATP8A2, SHISA2, LINC00415, RNF6, CDK8, WASF3, GPR12, USP12, USP12-AS1, USP12-DT, LINC00412, RPL21, SNORD102, SNORA27, RASL11A, GTF3A, MTIF3, LNX2, POLR1D, GSX1, PLUT, PDX1, LINC00543, CDX2, URAD, FLT3, PAN3-AS1, PAN3, FLT1, POMP, SLC46A3, MTUS2, MTUS2-AS1, SLC7A1, LOC102723345, UBL3, LINC00297, LINC00572, LINC00544, LINC00365, LINC00384, KATNAL1, LINC00426, LINC01058, UBE2L5, HMGB1, USPL1, ALOX5AP, LINC00398, LINC00545, TEX26-AS1, MEDAG, TEX26, HSPH1, B3GLCT, RXFP2, EEF1DP3, FRY-AS1, FRY | arr[GRCh38] 13q11q13.1(18,862,147_32,123,927)x4 |
| 2  Brain | Gain | 13 | q14.3 | q21.1 | 4,274 | THSD1, VPS36, CKAP2, LINC00345, TPTE2P3, HNRNPA1L2, SUGT1-DT, SUGT1, CNMD, MIR759, PCDH8, OLFM4, LINC01065, LINC00558, LINC00458, MIR1297, MIR5007 | arr[GRCh38] 13q14.3q21.1(52,397,226_56,671,393)x4 |
| 2  Brain | Gain | 13 | q21.31 | q22.3 | 13,794 | LINC00395, OR7E156P, LOC112267897, LOC102723968, LOC647264, LINC00355, LINC01052, MIR548X2, MIR4704, PCDH9, PCDH9-AS2, PCDH9-AS3, PCDH9-AS4, LINC00364, LINC00550, LINC00383, KLHL1, ATXN8OS, LINC00348, DACH1, MZT1, BORA, DIS3, PIBF1, KLF5, LINC00393, LINC00392, KLF12, LINC00402, LOC100288208, LINC00381, LINC00347, CTAGE11P, LINC01078, TBC1D4, COMMD6, UCHL3, LMO7-AS1, LMO7, LMO7DN, LMO7DN-IT1, LINC00561, KCTD12, BTF3P11, ACOD1, CLN5, FBXL3, MYCBP2, MYCBP2-AS1, MYCBP2-AS2 | arr[GRCh38] 13q21.31q22.3(63,475,403_77,269,296)x4 |
| 2  Brain | Gain | 15 | q11.2 | q13.1 | 4,474 | MAGEL2, NDN, PWRN4, PWRN2, PWRN3, PWRN1, NPAP1, SNHG14, SNRPN, SNURF, SNORD107, PWARSN, PWAR5, SNORD64, SNORD108, PWAR6, SNORD109A, SNORD116-1, SNORD116-2, SNORD116-3, SNORD116-4, SNORD116-5, SNORD116-6, SNORD116-7, SNORD116-8, SNORD116-9, SNORD116-10, SNORD116-11, SNORD116-12, SNORD116-13, SNORD116-14, SNORD116-15, SNORD116-16, SNORD116-17, SNORD116-18, SNORD116-19, SNORD116-20, SNORD116-21, SNORD116-22, SNORD116-23, SNORD116-24, SNORD116-25, SNORD116-26, SNORD116-27, SNORD116-28, SNORD116-29, SNORD116-30, IPW, PWAR1, SNORD115-1, SNORD115-2, SNORD115-3, SNORD115-4, SNORD115-5, SNORD115-6, SNORD115-7, SNORD115-8, SNORD115-9, SNORD115-10, SNORD115-11, SNORD115-12, SNORD115-13, SNORD115-14, SNORD115-15, SNORD115-16, SNORD115-17, SNORD115-18, SNORD115-19, SNORD115-20, SNORD115-21, SNORD115-22, PWAR4, SNORD115-23, SNORD115-24, SNORD115-25, SNORD115-26, SNORD115-27, SNORD115-28, SNORD115-29, SNORD115-30, SNORD115-31, SNORD115-32, SNORD115-33, SNORD115-34, SNORD115-35, SNORD115-36, SNORD115-37, SNORD115-38, SNORD115-39, SNORD115-40, SNORD115-41, SNORD115-42, SNORD115-43, SNORD115-44, SNORD115-45, SNORD115-46, SNORD115-47, SNORD115-48, SNORD109B, UBE3A, ATP10A, MIR4715, ATP10A-DT, LINC02346, LINC00929, GABRB3, GABRA5, GABRG3, GABRG3-AS1, OCA2 | arr[GRCh38] 15q11.2q13.1(23,606,849_28,081,338)x3~4 |
| 2  Brain | Gain | 15 | q13.3 | q15.1 | 8,451 | ARHGAP11A, ARHGAP11A-SCG5, SCG5, SCG5-AS1, GREM1, GREM1-AS1, FMN1, TMCO5B, RYR3-DT, RYR3, AVEN, CHRM5, EMC7, PGBD4, KATNBL1, EMC4, SLC12A6, NOP10, NUTM1, LPCAT4, GOLGA8A, MIR1233-1, GOLGA8B, MIR1233-2, LINC02252, GJD2, GJD2-DT, ACTC1, AQR, ZNF770, NANOGP8, ANP32AP1, DPH6, MIR3942, DPH6-DT, MIR4510, LINC02853, CDIN1, CSNK1A1P1, LOC145845, MEIS2, MIR8063, TMCO5A, LINC02345, LINC01852, SPRED1, FAM98B, RASGRP1, LINC02694, LINC02915, THBS1, FSIP1, LOC105370941, GPR176, GPR176-DT, EIF2AK4, SRP14, SRP14-DT, BMF, BUB1B, BUB1B-PAK6, PAK6, PAK6-AS1, ANKRD63, PLCB2, INAFM2, CCDC9B, PHGR1, DISP2, KNSTRN, IVD, BAHD1, CHST14, CCDC32, MRPL42P5, RPUSD2, KNL1, RAD51-AS1, RAD51, RMDN3, GCHFR, DNAJC17, C15orf62, ZFYVE19, PPP1R14D, SPINT1-AS1, SPINT1, RHOV, VPS18, LOC105370943, DLL4, CHAC1, INO80, INO80-AS1 | arr[GRCh38] 15q13.3q15.1(32,632,994_41,084,308)x3~4 |
| 2  Brain | Gain | 15 | q21.1 | q21.3 | 11,358 | SEMA6D, LINC01491, SLC24A5, MYEF2, CTXN2, CTXN2-AS1, SLC12A1, DUT, FBN1, FBN1-DT, CEP152, SHC4, EID1, SECISBP2L, COPS2, GALK2, NDUFAF4P1, MIR4716, FAM227B, FGF7, DTWD1, ATP8B4, SLC27A2, HDC, GABPB1, GABPB1-IT1, GABPB1-AS1, MIR4712, USP8, USP50, TRPM7, LOC128092252, SPPL2A, AP4E1, DCAF13P3, MIR4713HG, TNFAIP8L3, CYP19A1, MIR4713, MIR7973-2, MIR7973-1, GLDN, DMXL2, SCG3, LYSMD2, TMOD2, TMOD3, LOC100422556, LEO1, MAPK6-DT, MAPK6, BCL2L10, GNB5, CERNA1, MYO5C, MIR1266, MYO5A, ARPP19, ATOSA, ONECUT1, LINC02490, WDR72, UNC13C, LOC105370829, RSL24D1, RAB27A, PIGBOS1, PIGB, DNAAF4-CCPG1, CCPG1, MIR628, PIERCE2, DNAAF4, PYGO1, PRTG, NEDD4, RFX7, TEX9, MNS1, ZNF280D, TCF12-DT, LINC03065, TCF12, SNORD13D, LINC00926, LINC01413, CGNL1, MYZAP, GCOM1, POLR2M, ALDH1A2, ALDH1A2-AS1, AQP9, LIPC, LIPC-AS1 | arr[GRCh38] 15q21.1q21.3(47,140,026_58,497,999)x3~4 |
| 2  Brain | Gain | 15 | q22.2 | q24.2 | 15,459 | ANXA2, ICE2, RORA-AS1, RORA, RORA-AS2, LINC02349, VPS13C, VPS13C-DT, C2CD4A, C2CD4B, LOC107984784, GOLGA2P11, MIR8067, MIR6085, TLN2, MGC15885, MIR190A, TPM1, TPM1-AS, LACTB, RPS27L, RAB8B, APH1B, CA12, LINC02568, USP3, USP3-AS1, FBXL22, HERC1, MIR422A, DAPK2, LOC101928988, CIAO2A, SNX1, SNX22, PPIB, CSNK1G1, PCLAF, TRIP4, ZNF609, OAZ2, RBPMS2, MIR1272, PIF1, PLEKHO2, ANKDD1A, SPG21, MTFMT, SLC51B, RASL12, KBTBD13, UBAP1L, PDCD7, CLPX, CILP, PARP16, SNORA24B, RNU5A-1, RNU5B-1, IGDCC3, IGDCC4, DPP8, HACD3, INTS14, SLC24A1, DENND4A, SNORD13E, MIR4511, RAB11A, MEGF11, MIR4311, DIS3L-AS1, DIS3L, TIPIN, SCARNA14, MAP2K1, SNAPC5, MIR4512, RPL4, SNORD18C, SNORD18B, SNORD16, SNORD18A, ZWILCH, LCTL, LINC01169, SMAD6, SMASR, SMAD3-DT, SMAD3, AAGAB, IQCH, IQCH-AS1, C15orf61, MAP2K5, SKOR1, SKOR1-AS1, RNU6-1, PIAS1, CALML4, CLN6, FEM1B, ITGA11, CORO2B, ANP32A, MIR4312, ANP32A-IT1, SPESP1, NOX5, EWSAT1, GLCE, PAQR5-DT, PAQR5, KIF23-AS1, KIF23, RPLP1, DRAIC, PCAT29, LINC00593, TLE3, MIR629, LINC02205, LINC02204, SALRNA3, SALRNA2, UACA, LARP6, LRRC49, THAP10, THSD4, CT62, THSD4-AS1, THSD4-AS2, NR2E3, MYO9A, SENP8, GRAMD2A, PKM, PARP6, CELF6, HEXA, HEXA-AS1, TMEM202, TMEM202-AS1, ARIH1, MIR630, LINC02259, GOLGA6B, HIGD2B, BBS4, ADPGK, ADPGK-AS1, NEO1, MIR12135, HCN4, REC114, NPTN, NPTN-IT1, CD276, INSYN1, INSYN1-AS1, TBC1D21, LOXL1-AS1, LOXL1, STOML1, PML, GOLGA6A, COMMD4P2, LOC283731, ISLR2, ISLR, STRA6, CCDC33, CYP11A1, PPIAP46, LINC02255, SEMA7A, MIR6881, UBL7, UBL7-DT, ARID3B, CLK3, EDC3, CYP1A1, CYP1A2, CSK, MIR4513, LMAN1L, CPLX3, ULK3, MIR6882, SCAMP2, MPI, FAM219B, COX5A, RPP25, SCAMP5, PPCDC, C15orf39, LOC105376731, GOLGA6C, GOLGA6D, COMMD4, NEIL1, MIR631, MAN2C1, SIN3A, PTPN9 | arr[GRCh38] 15q22.2q24.2(60,017,253_75,476,399)x3~4 |
| 2  Brain | Gain | 15 | q24.3 | q26.3 | 24,427 | HMG20A, LOC101929457, LINGO1, LINGO1-AS1, LINGO1-AS2, GOLGA6FP, COMMD4P1, LOC91450, TBC1D2B, SH2D7, CIB2, IDH3A, ACSBG1, DNAJA4, SKIC8, CRABP1, IREB2, HYKK, PSMA4, CHRNA5, CHRNA3, CHRNB4, LOC646938, ADAMTS7, MORF4L1, CTSH, RASGRF1, LOC100129540, ANKRD34C-AS1, MIR184, ANKRD34C, TMED3, MINAR1, MTHFS, ST20-MTHFS, ST20, ST20-AS1, BCL2A1, ZFAND6, FAH, CTXND1, LINC00927, ARNT2-DT, ARNT2, LOC101929586, MIR5572, ABHD17C, CEMIP, MIR549A, MESD, MIR4514, TLNRD1, LOC128071545, CFAP161, IL16, STARD5, TMC3-AS1, TMC3, MEX3B, LINC01583, EFL1, SAXO2, ADAMTS7P1, GOLGA6L10, UBE2Q2P2, GOLGA6L9, LOC102724034, GOLGA2P10, GOLGA6L17P, RPS17, CPEB1, CPEB1-AS1, AP3B2, LOC338963, ACTG1P17, SNHG21, FSD2, SCARNA15, WHAMM, HOMER2, RAMAC, C15orf40, BTBD1, MIR4515, TM6SF1, HDGFL3, BNC1, SH3GL3, ADAMTSL3, EFL1P1, DNM1P41, UBE2Q2P16, LOC440300, GOLGA2P7, GOLGA6L4, LOC103171574, LOC102724135, GOLGA6L5P, UBE2Q2P1, LINC00933, ZSCAN2, SCAND2P, WDR73, NMB, SEC11A, ZNF592, ALPK3, SLC28A1, PDE8A, LOC727751, LOC101929479, GOLGA6L3P, MIR7706, AKAP13, LINC02883, KLHL25, MIR1276, MIR548AP, AGBL1, LINC01584, AGBL1-AS1, LOC105370954, LOC102724452, LINC00052, NTRK3, MIR11181, NTRK3-AS1, MRPL46, MRPS11, DET1, LINC01586, MIR1179, MIR7-2, MIR3529, AEN, ISG20, ACAN, HAPLN3, MFGE8, CARMAL, ABHD2, RLBP1, FANCI, POLG, POLGARF, MIR6766, MIR9-3HG, MIR9-3, LOC105371031, RHCG, LINC00928, TICRR, KIF7, PLIN1, PEX11A, WDR93, MESP1, MESP2, ANPEP, AP3S2, ARPIN-AP3S2, MIR5094, MIR5009, ARPIN, ZNF710, MIR3174, ZNF710-AS1, IDH2, IDH2-DT, SEMA4B, CIB1, GDPGP1, TTLL13, NGRN, GABARAPL3, ZNF774, IQGAP1, CRTC3, CRTC3-AS1, LINC01585, BLM, FURIN, FES, MAN2A2, HDDC3, UNC45A, RCCD1-AS1, RCCD1, PRC1, PRC1-AS1, VPS33B, VPS33B-DT, SV2B, CRAT37, SLCO3A1, ST8SIA2, LOC104613533, C15orf32, LINC00930, FAM174B, ASB9P1, CHASERR, CHD2, MIR3175, RGMA, LOC105370980, LINC02207, LINC01579, LINC01580, LINC01581, MCTP2, LOC440311, LETR1, LINC00924, LOC105369212, NR2F2-AS1, NR2F2, MIR1469, SPATA8-AS1, SPATA8, LINC02253, LINC02254, LOC101927310, LINC00923, ARRDC4, LINC02251, LINC01582, LINC02351, FAM169BP, IRAIN, IGF1R, MIR4714, PGPEP1L, LUNAR1, SYNM, TTC23, LRRC28, HSP90B2P, MEF2A, LYSMD4, DNM1P46, LOC400464, ADAMTS17, SPATA41, CERS3-AS1, CERS3, PRKXP1, LOC102723335, LINS1, ASB7, GCAWKR, ALDH1A3, ALDH1A3-AS1, LRRK1, CHSY1, SELENOS, SNRPA1, PCSK6, LOC100507472, PCSK6-AS1, LINC02348, TM2D3, TARS3, UBE2Q2P13, OR4F6, OR4F15, OR4F13P | arr[GRCh38] 15q24.3q26.3(77,461,913_101,888,910)x3~4 |
| 2  Brain | Loss | 16 | p13.3 | p13.13 | 11,349 | POLR3K, SNRNP25, RHBDF1, MPG, NPRL3, HBZ, HBM, HBA2, HBA1, HBQ1, LUC7L, FAM234A, RGS11, ARHGDIG, PDIA2, AXIN1, MRPL28, PGAP6, LOC100134368, NME4, DECR2, RAB11FIP3, LINC00235, CAPN15, MIR5587, MIR3176, PRR35, NHLRC4, PIGQ, RAB40C, WFIKKN1, METTL26, LOC100287175, MCRIP2, LOC105371038, WDR90, RHOT2, RHBDL1, STUB1-DT, STUB1, JMJD8, WDR24, FBXL16, METRN, ANTKMT, CCDC78, HAGHL, CIAO3, MSLN, MIR662, RPUSD1, CHTF18, GNG13, LMF1, LMF1-AS1, CEROX1, SOX8, SSTR5-AS1, SSTR5, C1QTNF8, CACNA1H, TPSG1, TPSB2, TPSAB1, TPSD1, UBE2I, BAIAP3, TSR3, GNPTG, UNKL, UQCC4, PERCC1, CCDC154, CLCN7, PTX4, TELO2, IFT140, TMEM204, LOC105371046, CRAMP1, JPT2, MAPK8IP3, MAPK8IP3-AS1, MIR3177, NME3, MRPS34, EME2, SPSB3, NUBP2, IGFALS, HAGH, FAHD1, MEIOB, LINC00254, LINC02124, HS3ST6, MSRB1, RPL3L, NDUFB10, RPS2, SNORA10, SNORA64, SNHG9, SNORA78, RNF151, TBL3, NOXO1, GFER, SYNGR3, ZNF598, NPW, NHERF2, NTHL1, TSC2, PKD1, MIR1225, PKD1-AS1, MIR6511B1, MIR4516, MIR3180-5, RAB26, SNHG19, SNORD60, TRAF7, CASKIN1, MLST8, BRICD5, PGP, E4F1, DNASE1L2, ECI1, RNPS1, MIR3677HG, MIR3677, MIR940, MIR4717, ABCA3, ABCA17P, CCNF, MIR6767, TEDC2, MIR6768, TEDC2-AS1, NTN3, TBC1D24, ATP6V0C, AMDHD2, CEMP1, MIR3178, PDPK1, LOC652276, FLJ42627, ERVK13-1, KCTD5, PRSS27, SRRM2-AS1, SRRM2, ELOB, PRSS33, SNORA3C, PRSS41, PRSS21, ZG16B, PRSS30P, PRSS22, FLYWCH2, FLYWCH1, KREMEN2, PAQR4, PKMYT1, GREP1, CLDN9, CLDN6, TNFRSF12A, HCFC1R1, THOC6, BICDL2, LOC100128770, MMP25, MMP25-AS1, IL32, ZSCAN10, ZNF205-AS1, ZNF205, ZNF213-AS1, ZNF213, CASP16P, OR1F1, OR1F2P, ZNF200, MEFV, LINC00921, ZNF263, TIGD7, ZNF75A, OR2C1, ZSCAN32, ZNF174, ZNF597, NAA60, MIR6126, C16orf90, CLUAP1, NLRC3, SLX4, DNASE1, TRAP1, CREBBP, LINC02861, ADCY9, SRL, LINC01569, TFAP4, GLIS2, GLIS2-AS1, PAM16, CORO7-PAM16, CORO7, VASN, DNAJA3, NMRAL1, HMOX2, CDIP1, C16orf96, UBALD1, MGRN1, MIR6769A, NUDT16L1, ANKS3, DNAAF8, ZNF500, SEPTIN12, SMIM22, ROGDI, GLYR1, UBN1, PPL, SEC14L5, NAGPA, NAGPA-AS1, C16orf89, ALG1, EEF2KMT, RBFOX1, LINC01570, MIR8065, LINC02152, TMEM114, METTL22, ABAT, TMEM186, PMM2, LOC100130283, CARHSP1, LITAFD, USP7, USP7-AS1, HAPSTR1, LINC02177, LINC01177, LINC01195, GRIN2A, ATF7IP2, LINC01290, EMP2, TEKT5, NUBP1, TVP23A, CIITA, DEXI, CLEC16A, SOCS1, TNP2, PRM3, PRM2, PRM1, MIR548H2, LOC105371083, RMI2, LOC400499 | arr[GRCh38] 16p13.3p13.13(35,881_11,384,748)x1 |
| 2  Brain | cn-LOH | 17 | p13.3 | p11.2 | 17,945 | DOC2B, LINC02091, RPH3AL, RPH3AL-AS1, LOC105371430, LIAT1, RFLNB, VPS53, TLCD3A, GEMIN4, DBIL5P, GLOD4, MRM3, NXN, LOC101927727, TIMM22, ABR, MIR3183, BHLHA9, TRARG1, YWHAE, CRK, MYO1C, INPP5K, PITPNA-AS1, PITPNA, SLC43A2, SCARF1, RILP, PRPF8, TLCD2, MIR22HG, MIR22, WDR81, SERPINF2, SERPINF1, SMYD4, RPA1, RTN4RL1, LOC105371485, DPH1, OVCA2, MIR132, MIR212, HIC1, SMG6, LOC101927839, SRR, TSR1, SNORD91B, SNORD91A, SGSM2, MNT, LOC284009, METTL16, PAFAH1B1, CLUH, MIR6776, LOC105371592, CCDC92B, MIR1253, RAP1GAP2, LOC101927911, OR1D5, OR1D2, OR1G1, LOC100288728, OR1A2, OR1A1, OR1D4, OR3A2, OR3A1, OR3A4P, OR1R1P, OR1E1, OR3A3, OR1E2, SPATA22, ASPA, TRPV3, TRPV1, SHPK, CTNS, P2RX5-TAX1BP3, TAX1BP3, EMC6, P2RX5, ITGAE, HASPIN, NCBP3, CAMKK1, P2RX1, ATP2A3, LINC01975, ZZEF1, CYB5D2, ANKFY1, UBE2G1, SPNS3, SPNS2, MYBBP1A, GGT6, SMTNL2, LINC01996, ALOX15, PELP1, PELP1-DT, ARRB2, MED11, CXCL16, ZMYND15, TM4SF5, VMO1, GLTPD2, PSMB6, C17orf114, PLD2, MINK1, CHRNE, C17orf107, GP1BA, SLC25A11, RNF167, PFN1, ENO3, SPAG7, CAMTA2, MIR6864, MIR6865, INCA1, KIF1C, KIF1C-AS1, SLC52A1, ZFP3, ZNF232, ZNF232-AS1, USP6, ZNF594, ZNF594-DT, SCIMP, RABEP1, NUP88, RPAIN, C1QBP, DHX33, DHX33-DT, DERL2, MIS12, LOC728392, NLRP1, LOC339166, WSCD1, AIPL1, PIMREG, PITPNM3, KIAA0753, LOC122526780, TXNDC17, MED31, C17orf100, MIR4520-1, MIR4520-2, ALOX15P1, SLC13A5, XAF1, FBXO39, TEKT1, ALOX12P2, ALOX12-AS1, ALOX12, RNASEK-C17orf49, RNASEK, C17orf49, MIR497HG, MIR195, MIR497, BCL6B, SLC16A13, SLC16A11, CLEC10A, ASGR2, ASGR1, DLG4, ACADVL, MIR324, DVL2, PHF23, GABARAP, CTDNEP1, ELP5, CLDN7, SLC2A4, YBX2, EIF5A, GPS2, NEURL4, ACAP1, KCTD11, TMEM95, TNK1, PLSCR3, TMEM256-PLSCR3, TMEM256, NLGN2, SPEM1, SPEM2, SPEM3, TMEM102, FGF11, CHRNB1, ZBTB4, SLC35G6, POLR2A, TNFSF12, TNFSF12-TNFSF13, TNFSF13, SENP3, SENP3-EIF4A1, EIF4A1, SNORA48, SNORD10, SNORA67, CD68, MPDU1-AS1, MPDU1, SOX15, FXR2, SHBG, SAT2, ATP1B2, TP53, WRAP53, EFNB3, DNAH2, RPL29P2, KDM6B, TMEM88, NAA38, CYB5D1, CHD3, SCARNA21, RNF227, KCNAB3, TRAPPC1, CNTROB, GUCY2D, ALOX15B, ALOX12B, MIR4314, ALOXE3, HES7, PER1, MIR6883, VAMP2, TMEM107, SNORD118, MIR4521, BORCS6, AURKB, LINC00324, CTC1, PFAS, SLC25A35, RANGRF, ARHGEF15, ODF4, LOC100128288, KRBA2, RPL26, RNF222, NDEL1, MYH10, CCDC42, SPDYE4, MFSD6L, PIK3R6, PIK3R5, PIK3R5-DT, NTN1, LOC101928266, STX8, CFAP52, USP43, DHRS7C, GSG1L2, GLP2R, RCVRN, GAS7, MYH13, MYHAS, MYH8, MYH4, MYH1, MYH2, MYH3, SCO1, ADPRM, TMEM220, MAGOH2P, TMEM220-AS1, TMEM238L, PIRT, SHISA6, DNAH9, ZNF18, MAP2K4, MIR744, LINC00670, MYOCD, MYOCD-AS1, ARHGAP44-AS1, ARHGAP44, MIR1269B, ELAC2, LINC02093, HS3ST3A1, MIR548H3, CDRT15P1, COX10-DT, COX10, CDRT15, HS3ST3B1, MGC12916, CDRT7, LOC101928475, CDRT8, PMP22, MIR4731, TEKT3, CDRT4, TVP23C-CDRT4, CDRT3, TVP23C, FBXW10B, TRIM16, ZNF286A, ZNF286A-TBC1D26, TBC1D26, CDRT15P2, MEIS3P1, LINC02087, ADORA2B, ZSWIM7, TTC19, NCOR1, SNORD163, PIGL, MIR1288, CENPV, UBB, TRPV2, SNHG29, SNORD49B, SNORD49A, SNORD65, LRRC75A, ZNF287, ZNF624, CCDC144A, USP32P1, FAM106C, KRT16P2, KRT17P1, TBC1D27P, TNFRSF13B, LOC284191, LINC02090, MPRIP, PLD6, FLCN, COPS3, NT5M, MED9, RASD1, PEMT, SMCR2, RAI1, RAI1-AS1, SMCR5, SREBF1, MIR6777, MIR33B, TOM1L2, DRC3, ATPAF2, GID4, DRG2, LOC105371566, MYO15A | arr[GRCh38] 17p13.3p11.2(170,188_18,115,042)x3 hmz |
| 2  Brain | Gain | 18 | p11.32 | p11.31 | 5,263 | USP14, THOC1, COLEC12, LINC01925, CETN1, CLUL1, TYMSOS, TYMS, ENOSF1, YES1, ADCYAP1, LINC01904, LINC00470, METTL4, NDC80, CBX3P2, SMCHD1, EMILIN2, LPIN2, CHORDC1P4, MYOM1, MYL12A, MYL12-AS1, MYL12B, LINC01895, TGIF1, GAPLINC, DLGAP1, DLGAP1-AS1, DLGAP1-AS2, DLGAP1-AS3, MIR6718, DLGAP1-AS4, DLGAP1-AS5, LINC01892, AKAIN1, LINC00526, LINC00667, ZBTB14, EPB41L3 | arr[GRCh38] 18p11.32p11.31(136,227_5,399,711)x2~3 |
| 2  Brain | Gain | 18 | p11.23 | p11.21 | 5,807 | PTPRM, LOC100192426, RAB12, GACAT2, MTCL1, NDUFV2, NDUFV2-AS1, ANKRD12, TWSG1-DT, TWSG1, RALBP1, PPP4R1, PPP4R1-AS1, RAB31, TXNDC2, VAPA, LINC01254, APCDD1, NAPG, LINC01887, LOC101927410, PIEZO2, MIR6788, LINC01928, LINC01255, SLC35G4, MIR7153, GNAL, CHMP1B, MPPE1, IMPA2, ANKRD62, C18orf61, CIDEA, TUBB6, AFG3L2, PRELID3A, LOC105371998, SPIRE1, PSMG2, CEP76, LINC01882, PTPN2, SEH1L, CEP192, LDLRAD4, C18orf15, LDLRAD4-AS1, MIR5190, MIR4526, FAM210A, RNMT, MC5R, MC2R | arr[GRCh38] 18p11.23p11.21(8,250,937_14,058,311)x3~4 |
| 2  Brain | cn-LOH | 18 | q11.1 | q23 | 59,276 | ROCK1, GREB1L-DT, GREB1L, ESCO1, SNRPD1, ABHD3, MIR320C1, MIB1, MIR133A1HG, MIR133A1, MIR1-2, GATA6-AS1, GATA6, CTAGE1, LOC101927571, RBBP8, MIR4741, CABLES1, TMEM241, RIOK3, RMC1, NPC1, ANKRD29, LAMA3, TTC39C, TTC39C-AS1, CABYR, OSBPL1A, MIR320C2, IMPACT, HRH4, LINC01915, LOC105372028, LINC01894, ZNF521, SS18, PSMA8, TAF4B, LINC01543, KCTD1, MIR8057, PCAT18, AQP4, AQP4-AS1, CHST9, LOC105372038, CDH2, MIR302F, DSC3, DSC2, DSCAS, DSC1, DSG1, DSG1-AS1, DSG4, DSG3, DSG2, DSG2-AS1, TTR, B4GALT6, SLC25A52, TRAPPC8, RNF125, RNF138, MEP1B, GAREM1, WBP11P1, KLHL14, CCDC178, ASXL3, NOL4, DTNA, MAPRE2, ZNF397, ZSCAN30, ZNF271P, ZNF24, ZNF396, INO80C, GALNT1, MIR3975, MIR187, MIR3929, C18orf21, RPRD1A, SLC39A6, ELP2, COSMOC, MOCOS, FHOD3, LOC105372071, TPGS2, KIAA1328, LOC105372069, CELF4, LOC105372068, SNORA111, MIR4318, MIR924HG, MIR924, MIR5583-2, MIR5583-1, LINC01902, LINC01477, KC6, PIK3C3, LINC00907, RIT2, SYT4, LINC01478, LINC01601, SETBP1, MIR4319, SLC14A2, SLC14A1, EPG5, SIGLEC15, PSTPIP2, ATP5F1A, HAUS1, ARK2N, ARK2C, LOXHD1, ST8SIA5, PIAS2, KATNAL2, ELOA2, HDHD2, IER3IP1, SKOR2, MIR4527HG, MIR4527, SMAD2, ZBTB7C, ZBTB7C-AS2, CTIF, MIR4743, SMAD7, DYM-AS1, DYM, MIR4744, C18orf32, RPL17-C18orf32, MIR1539, RPL17, SNORD58C, SNORD58A, SNORD58B, LINC02837, LIPG, ACAA2, SCARNA17, SNHG22, MYO5B, MIR4320, CFAP53, MBD1, CXXC1, SKA1, MAPK4, MRO, ME2, ELAC1, SMAD4, MEX3C, LINC01630, DCC, MIR4528, LINC01919, LINC01917, MBD2, SNORA37, POLI, STARD6, C18orf54, DYNAP, RAB27B, CCDC68, LINC01929, TCF4, TCF4-AS1, MIR4529, LINC01415, LINC01416, LINC03069, LINC01905, LINC01539, TXNL1, WDR7, LINC-ROR, BOD1L2, LINC02565, ST8SIA3, ONECUT2, FECH, NARS1, ATP8B1-AS1, ATP8B1, NEDD4L, MIR122HG, MIR122, MIR3591, ALPK2, SNORA108, MALT1-AS1, MALT1, LINC01926, ZNF532, OACYLP, SEC11C, GRP, RAX, CPLX4, LMAN1, CCBE1, PMAIP1, MC4R, CDH20, LINC01544, RNF152, PIGN, RELCH, TNFRSF11A, ZCCHC2, PHLPP1, BCL2, KDSR, VPS4B, SERPINB5, SERPINB12, SERPINB13, SERPINB4, SERPINB3, SERPINB11, SERPINB7, SERPINB2, SERPINB10, HMSD, SERPINB8, LINC00305, LINC01924, LINC01538, CDH7, CDH19, MIR5011, DSEL, DSEL-AS1, LINC01903, LINC01912, TMX3, CCDC102B, DOK6, LOC105372179, CD226, RTTN, SOCS6, LINC01909, LIVAR, LINC01910, GTSCR1, LINC01541, LINC01899, CBLN2, NETO1, MIR548AV, NETO1-DT, LINC02864, LINC02582, FBXO15, TIMM21, CYB5A, C18orf63, LINC01922, DIPK1C, CNDP2, CNDP1, ZNF407-AS1, ZNF407, PTGR3, TSHZ1, SMIM21, LINC01898, LOC339298, LINC01893, ZNF516, ZNF516-AS1, ZNF516-DT, LINC00908, LINC00683, LINC01927, LINC01879, ZNF236-DT, ZNF236, MBP, GALR1, LINC01029, SALL3, ATP9B, NFATC1, LOC284240, CTDP1-DT, CTDP1, KCNG2, SLC66A2, HSBP1L1, TXNL4A, RBFA, RBFADN, ADNP2, PARD6G-AS1, PARD6G | arr[GRCh38] 18q11.1q23(20,980,393_80,256,699)x2 hmz |
| 2  Brain | Gain | 19 | p13.3 | p11 | 24,063 | PLPP2, MIER2, THEG, C2CD4C, SHC2, ODF3L2, MADCAM1, TPGS1, CDC34, GZMM, BSG-AS1, BSG, HCN2, POLRMT, FGF22, RNF126, FSTL3, PRSS57, PALM, MISP, PTBP1, MIR4745, PLPPR3, MIR3187, AZU1, PRTN3, ELANE, CFD, MED16, RNU6-9, R3HDM4, KISS1R, ARID3A, WDR18, GRIN3B, TMEM259, RNU6-2, CNN2, ABCA7, ARHGAP45, POLR2E, GPX4, SBNO2, STK11, CBARP, ATP5F1D, MIDN, CIRBP-AS1, CIRBP, FAM174C, EFNA2, PWWP3A, NDUFS7, GAMT, DAZAP1, RPS15, APC2, C19orf25, PCSK4, REEP6, ADAMTSL5, PLK5, MEX3D, MBD3, UQCR11, TCF3, ONECUT3, ATP8B3, REXO1, MIR1909, LOC100288123, KLF16, ABHD17A, ADAT3, SCAMP4, CSNK1G2, CSNK1G2-AS1, BTBD2, MKNK2, MOB3A, IZUMO4, AP3D1, DOT1L, PLEKHJ1, MIR1227, MIR6789, SF3A2, AMH, MIR4321, JSRP1, OAZ1, PEAK3, LINGO3, LSM7, SPPL2B, TMPRSS9, TIMM13, LMNB2, MIR7108, LINC01775, GADD45B, GNG7, MIR7850, DIRAS1, SLC39A3, SGTA, THOP1, ZNF554, ZNF555, ZNF556, ZNF57, ZNF77, TLE6, TLE2, TLE5, GNA11, GNA15, GNA15-DT, S1PR4, NCLN, CELF5, NFIC, SMIM24, SMIM44, DOHH, FZR1, TEKTIP1, MFSD12, HMG20B, GIPC3, TBXA2R, CACTIN-AS1, CACTIN, PIP5K1C, TJP3, APBA3, MRPL54, RAX2, MATK, ZFR2, ATCAY, NMRK2, DAPK3, MIR637, EEF2, SNORD37, PIAS4, ZBTB7A, MAP2K2, CREB3L3, SIRT6, ANKRD24, EBI3, YJU2, SHD, TMIGD2, FSD1, STAP2, MPND, SH3GL1, CHAF1A, UBXN6, MIR4746, HDGFL2, PLIN4, PLIN5, LRG1, SEMA6B, TNFAIP8L1, MYDGF, DPP9, DPP9-AS1, MIR7-3HG, MIR7-3, FEM1A, TICAM1, PLIN3, ARRDC5, UHRF1, MIR4747, KDM4B, PTPRS, ZNRF4, TINCR, SAFB2, SAFB, MICOS13, HSD11B1L, RPL36, LONP1, CATSPERD, PRR22, DUS3L, NRTN, FUT6, FUT3, LOC101928844, FUT5, NDUFA11, VMAC, CAPS, RANBP3, RANBP3-DT, RFX2, ACSBG2, MLLT1, ACER1, CLPP, ALKBH7, PSPN, GTF2F1, MIR6885, MIR6790, LOC390877, KHSRP, MIR3940, SLC25A41, SLC25A23, CRB3, DENND1C, TUBB4A, TNFSF9, CD70, TNFSF14, C3, GPR108, MIR6791, TRIP10, SH2D3A, VAV1, ADGRE1, ADGRE4P, FLJ25758, MBD3L2B, MBD3L5, MBD3L4, MBD3L2, MBD3L3, ZNF557, INSR, ARHGEF18, PEX11G, TEX45, ZNF358, MCOLN1, PNPLA6, CAMSAP3, MIR6792, XAB2, PET100, STXBP2, PCP2, RETN, MCEMP1, TRAPPC5, FCER2, CLEC4G, CD209, CLEC4M, CLEC4GP1, EVI5L, PRR36, LYPLA2P2, LRRC8E, MAP2K7, TGFBR3L, SNAPC2, CTXN1, TIMM44, ELAVL1, CCL25, FBN3, CERS4, CD320, NDUFA7, RPS28, KANK3, ANGPTL4, RAB11B-AS1, MIR4999, RAB11B, MARCHF2, HNRNPM, PRAM1, ZNF414, MYO1F, ADAMTS10, NFILZ, ACTL9, OR2Z1, ZNF558, MBD3L1, MUC16, OR1M1, OR7G2, OR7G1, OR7G3, ZNF317, OR7D2, OR7D4, OR7E24, ZNF699, ZNF559, ZNF559-ZNF177, ZNF177, ZNF266, ZNF560, ZNF426, ZNF426-DT, ZNF121, ZNF561, ZNF561-AS1, ZNF562, ZNF812P, ZNF846, LOC100505555, FBXL12, UBL5, PIN1-DT, PIN1, OLFM2, COL5A3, RDH8, MIR5589, C3P1, SHFL, ANGPTL6, PPAN, PPAN-P2RY11, SNORD105, SNORD105B, P2RY11, EIF3G, DNMT1, S1PR2, MIR4322, MRPL4, ICAM1, ICAM4, ICAM5, ZGLP1, FDX2-ZGLP1, FDX2, RAVER1, ICAM3, TYK2, CDC37, MIR1181, PDE4A, KEAP1, S1PR5, ATG4D, MIR1238, KRI1, CDKN2D, AP1M2, SLC44A2, ILF3-DT, ILF3, QTRT1, DNM2, MIR638, MIR4748, MIR199A1, MIR6793, TMED1, HIKESHIP2, C19orf38, CARM1, YIPF2, TIMM29, SMARCA4, LDLR-AS1, LDLR, MIR6886, SPC24, KANK2, DOCK6, LOC105372273, ANGPTL8, TSPAN16, RAB3D, TMEM205, CCDC159, PLPPR2, SWSAP1, EPOR, RGL3, ODAD3, PRKCSH, ELAVL3, ZNF653, MIR7974, ECSIT, CNN1, ELOF1, ACP5, ZNF627, HNRNPA1P10, ZNF833P, ZNF823, ZNF441, ZNF491, ZNF440, ZNF439, ZNF69, ZNF700, ZNF763, ZNF433-AS1, ZNF433, ZNF878, ZNF844, ZNF788P, ZNF20, ZNF625-ZNF20, ZNF625, ZNF136, LOC100289333, ZNF44, ZNF563, ZNF442, ZNF799, ZNF443, ZNF709, ZNF564, ZNF490, ZNF791, MAN2B1, WDR83, WDR83OS, DHPS, GNG14, FBXW9, TNPO2, SNORD135, SNORD41, TRIR, GET3, BEST2, HOOK2, MIR5684, JUNB, PRDX2, THSD8, RNASEH2A, RTBDN, MAST1, MIR6794, DNASE2, KLF1, GCDH, SYCE2, MIR5695, FARSA, FARSA-AS1, CALR, MIR6515, RAD23A, GADD45GIP1, DAND5, NFIX, LYL1, TRMT1, NACC1, STX10, IER2, CACNA1A, YJU2B, MRI1, C19orf53, ZSWIM4, MIR23AHG, MIR24-2, MIR27A, MIR23A, NANOS3, MIR181C, MIR181D, BRME1, CC2D1A, PODNL1, DCAF15, RFX1, RLN3, IL27RA, PALM3, MISP3, MIR1199, C19orf67, SAMD1, PRKACA, SMIM46, ASF1B, ADGRL1-AS1, ADGRL1, LINC01841, LINC01842, ADGRE5, DDX39A, PKN1, PTGER1, GIPC1, DNAJB1, MIR639, TECR, NDUFB7, CLEC17A, ADGRE3, SNORA104, ZNF333, ADGRE2, OR7C1, OR7A5, OR7A10, OR7A17, OR7C2, SLC1A6, CCDC105, CASP14, OR1I1, SYDE1, ILVBL, NOTCH3, MIR6795, EPHX3, BRD4, AKAP8, AKAP8L, WIZ, MIR1470, RASAL3, PGLYRP2, CYP4F22, CYP4F8, CYP4F3, CYP4F12, OR10H2, OR10H3, CYP4F24P, OR10H5, OR10H1, UCA1, CLEC4OP, CYP4F2, CYP4F11, OR10H4, LINC00661, LINC00905, LINC01855, TPM4, RAB8A, HSH2D, CIB3, FAM32A, AP1M1, KLF2, EPS15L1, CALR3, C19orf44, CHERP, SLC35E1, MED26, SMIM7, TMEM38A, NWD1, SIN3B, F2RL3, CPAMD8, HAUS8, MYO9B, SNORA118, USE1, OCEL1, NR2F6, USHBP1, BABAM1, ANKLE1, ABHD8, MRPL34, DDA1, ANO8, GTPBP3, PLVAP, CCDC194, BST2, BISPR, MVB12A, TMEM221, NXNL1, SLC27A1, PGLS-DT, PGLS, NIBAN3, COLGALT1, UNC13A, MAP1S, FCHO1, B3GNT3, INSL3, JAK3, RPL18A, SNORA68, SLC5A5, CCDC124, KCNN1, ARRDC2, IL12RB1, MAST3, PIK3R2, IFI30, MPV17L2, RAB3A, LOC102725254, PDE4C, LOC729966, IQCN, JUND, MIR3188, LSM4, PGPEP1, GDF15, MIR3189, LRRC25, SSBP4, ISYNA1, ELL, FKBP8, KXD1, UBA52, REX1BD, CRLF1, TMEM59L, KLHL26, CRTC1, COMP, UPF1, CERS1, GDF1, COPE, DDX49, HOMER3, HOMER3-AS1, SUGP2, ARMC6, SLC25A42, TMEM161A, MEF2B, BORCS8-MEF2B, BORCS8, RFXANK, NR2C2AP, NCAN, HAPLN4, TM6SF2, SUGP1, MAU2, GATAD2A, MIR640, TSSK6, NDUFA13, YJEFN3, CILP2, PBX4, LPAR2, GMIP, ATP13A1, ZNF101, ZNF14, LINC00663, ZNF56P, ZNF506, ZNF253, ZNF93, ZNF682, ZNF90, ZNF486, ZNF826P, MIR1270, ZNF737, ZNF626, LOC105372319, ZNF66, ZNF85, ZNF430, ZNF714, ZNF431, ZNF708, ZNF738, ZNF493, LINC00664, ZNF429, LOC400682, ZNF100, CCNYL6, ZNF43, ZNF208, ZNF257, ZNF676, ZNF729, ZNF98, LOC105376917, LOC101929124, LINC01233, GOLGA2P9, LOC100996349, LINC01785, ZNF492, ZNF99, ZNF723, ZNF728, LINC01859, LINC01858, ZNF730, ZNF724, IPO5P1, ZNF91, LINC01224, ZNF675, ZNF681, RPSA2, ZNF726, LOC100505851, ZNF254, HAVCR1P1 | arr[GRCh38] 19p13.3p11(260,912_24,324,339)x3 |
| 2  Brain | cn-LOH | 20 | p13 | p12.1 | 14,451 | DEFB125, DEFB126, DEFB127, DEFB128, DEFB129, DEFB132, C20orf96, ZCCHC3, NRSN2-AS1, SOX12, NRSN2, TRIB3, RBCK1, TBC1D20, CSNK2A1, TCF15, SRXN1, SCRT2, SLC52A3, FAM110A, ANGPT4, RSPO4, PSMF1, LOC105372493, TMEM74B, C20orf202, RAD21L1, SNPH, SDCBP2, FKBP1A-SDCBP2, SDCBP2-AS1, FKBP1A, MIR6869, NSFL1C, SIRPB2, SIRPD, SIRPB1, SIRPG, SIRPG-AS1, SIRPB3P, LOC100289473, SIRPA, PDYN-AS1, PDYN, STK35, LINC03086, TGM3, TGM6, SNRPB, SNORD119, ZNF343, TMC2, NOP56, MIR1292, SNORD110, SNORA51, SNORD86, SNORD56, SNORD57, IDH3B, EBF4, CPXM1, C20orf141, TMEM239, PCED1A, VPS16, PTPRA, GNRH2, MRPS26, OXT, AVP, UBOX5-AS1, UBOX5, FASTKD5, LZTS3, DDRGK1, ITPA, SLC4A11, DNAAF9, ATRN, GFRA4, ADAM33, SIGLEC1, HSPA12B, ADISSP, SPEF1, CENPB, CDC25B, LINC01730, AP5S1, MAVS, PANK2, MIR103A2, MIR103B2, RNF24, SMOX, LINC01433, ADRA1D, PRNP, PRND, PRNT, RASSF2, SLC23A2, TMEM230, PCNA, PCNA-AS1, CDS2, PROKR2, LINC00658, LOC643406, LINC00654, LINC01729, GPCPD1, SHLD1, CHGB, TRMT6, MCM8, MCM8-AS1, CRLS1, LRRN4, FERMT1, CASC20, LINC01713, BMP2, LINC01428, LINC01751, LINC01706, MIR8062, HAO1, TMX4, TMX4-AS1, PLCB1, PLCB1-IT1, RNU105B, PLCB4, LAMP5-AS1, LAMP5, PAK5, PARAL1, SNAP25-AS1, ANKEF1, SNAP25, MKKS, LOC128706665, LOC128706666, SLX4IP, JAG1, MIR6870, LINC01752, LOC101929413, LINC02871, LOC339593, LINC00687, BTBD3, LINC01722, LOC102606466, LINC01723, SPTLC3, ISM1, ISM1-AS1, TASP1, ESF1, NDUFAF5, SEL1L2, MACROD2, FLRT3 | arr[GRCh38] 20p13p12.1(94,613_14,546,046)x2 hmz |
| 2  Brain | cn-LOH | 20 | p12.1 | p11.21 | 7,859 | MACROD2, LOC613266, KIF16B, SNRPB2, OTOR, PCSK2, BFSP1, DSTN, RRBP1, BANF2, SNX5, SNORD17, MGME1, OVOL2, KAT14, PET117, ZNF133, LINC00851, DZANK1, POLR3F, MIR3192, RBBP9, SEC23B, SMIM26, DTD1, DTD1-AS1, LINC00652, LCDR, SCP2D1-AS1, SCP2D1, SLC24A3, SLC24A3-AS1, RIN2, NAA20, CRNKL1, CFAP61, CFAP61-AS1, INSM1, RALGAPA2, LINC00237, KIZ, KIZ-AS1, XRN2, NKX2-4, LOC112268271, NKX2-2, LINC01727, LINC01726, PAX1, LINC01432, LINC01427, LOC284788, LINC00261, FOXA2, LNCNEF, LINC01747, SSTR4, THBD, CD93, LINC00656, NXT1, LINC01431, GZF1 | arr[GRCh38] 20p12.1p11.21(15,510,115_23,369,112)x2 hmz |
| 2  Brain | cn-LOH | 20 | q11.21 | q12 | 10,836 | DEFB121, DEFB122, DEFB123, DEFB124, REM1, LINC00028, HM13, MCTS2, HM13-AS1, ID1, MIR3193, COX4I2, BCL2L1, ABALON, TPX2, MYLK2, FOXS1, DUSP15, TTLL9, PDRG1, XKR7, CCM2L, HCK, TM9SF4, TSPY26P, PLAGL2, POFUT1, MIR1825, KIF3B, ASXL1, NOL4L, LOC101929698, NOL4L-DT, C20orf203, COMMD7, DNMT3B, MAPRE1, LOC119746555, EFCAB8, SUN5, BPIFB2, BPIFB6, BPIFB3, BPIFB4, BPIFA2, BPIFA4P, BPIFA3, BPIFA1, BPIFB1, CDK5RAP1, SNTA1, CBFA2T2, NECAB3, C20orf144, ACTL10, E2F1, PXMP4, ZNF341, ZNF341-AS1, CHMP4B, RALY-AS1, RALY, MIR4755, EIF2S2, ASIP, AHCY, ITCH, MIR644A, DYNLRB1, MAP1LC3A, PIGU, TP53INP2, NCOA6, HMGB3P1, GGT7, ACSS2, GSS, MYH7B, MIR499A, MIR499B, TRPC4AP, EDEM2, MMP24-AS1-EDEM2, PROCR, MMP24, MMP24OS, EIF6, FAM83C-AS1, FAM83C, UQCC1, GDF5-AS1, GDF5, MIR1289-1, CEP250, CEP250-AS1, C20orf173, ERGIC3, FER1L4, SPAG4, CPNE1, RBM12, NFS1, ROMO1, RBM39, PHF20, SCAND1, CNBD2, NORAD, EPB41L1, EPB41L1-AS1, AAR2, DLGAP4, DLGAP4-AS1, MYL9, TGIF2, TGIF2-RAB5IF, RAB5IF, SLA2, NDRG3, DSN1, SOGA1, TLDC2, SAMHD1, RBL1, MROH8, RPN2, GHRH, MANBAL, SRC, BLCAP, NNAT, LINC01746, LINC00489, LOC100287792, CTNNBL1, VSTM2L, TTI1, RPRD1B, TGM2, KIAA1755, LOC149684, BPI, LBP, SNHG17, SNORA71B, SNORA71A, SNORA71C, SNORA71D, SNHG11, SNORA71E, SNORA60, RALGAPB, MIR548O2, ADIG, ARHGAP40, SLC32A1, ACTR5, PPP1R16B, FAM83D, DHX35, LINC01734, LINC01370, MAFB, SNORD154, LOC100128988, TOP1, PLCG1-AS1, PLCG1, MIR6871, ZHX3, LPIN3, EMILIN3, CHD6, PTPRT | arr[GRCh38] 20q11.21q12(31,394,130_42,230,410)x4 hmz |
| 2  Brain | Gain | 20 | q12 | q13.33 | 21,891 | PTPRT, LOC101927159, PTPRT-AS1, SRSF6, L3MBTL1, SGK2, IFT52, MYBL2, GTSF1L, LINC01728, TOX2, JPH2, OSER1, OSER1-DT, GDAP1L1, FITM2, R3HDML, R3HDML-AS1, HNF4A, HNF4A-AS1, MIR3646, LINC01430, LINC01620, TTPAL, SERINC3, PKIG, ADA, LINC01260, KCNK15-AS1, CCN5, KCNK15, RIMS4, YWHAB, PABPC1L, TOMM34, STK4-DT, STK4, KCNS1, WFDC5, WFDC12, PI3, SEMG1, SEMG2, SLPI, MATN4, RBPJL, SDC4, SYS1, SYS1-DBNDD2, TP53TG5, DBNDD2, PIGT, MIR6812, LOC105372631, WFDC2, SPINT3, WFDC6, EPPIN-WFDC6, EPPIN, WFDC8, WFDC9, WFDC10A, WFDC11, WFDC10B, WFDC13, MIR3617, SPINT4, WFDC3, DNTTIP1, UBE2C, TNNC2, SNX21, ACOT8, ZSWIM3, ZSWIM1, SPATA25, NEURL2, CTSA, PLTP, LOC107985388, PCIF1, ZNF335, MMP9, SLC12A5-AS1, SLC12A5, NCOA5, CD40, CDH22, SLC35C2, ELMO2, LOC105372633, ZNF663P, MKRN7P, ZNF334, OCSTAMP, SLC13A3, TP53RK, SLC2A10, EYA2, EYA2-AS1, MIR3616, ZMYND8, LOC100131496, LOC101927377, LINC01754, NCOA3, SULF2, LINC01522, LINC01523, LINC00494, PREX1, ARFGEF2, CSE1L-DT, CSE1L, STAU1, DDX27, ZNFX1, ZFAS1, SNORD12C, SNORD12B, SNORD12, KCNB1, PTGIS, B4GALT5, SLC9A8, MIR12122, SPATA2, LOC105372653, RNF114, SNAI1, TRERNA1, UBE2V1, PEDS1-UBE2V1, PEDS1, LINC01275, LINC01273, CEBPB-AS1, CEBPB, PELATON, LINC01270, LINC01271, PTPN1, MIR645, RIPOR3, MIR1302-5, RIPOR3-AS1, PARD6B, BCAS4, ADNP, ADNP-AS1, DPM1, MOCS3, KCNG1, NFATC2, MIR3194, ATP9A, SALL4, LINC01429, ZFP64, LINC01524, TSHZ2, LOC101927770, ZNF217, LOC105372672, SUMO1P1, BCAS1, MIR4756, CYP24A1, PFDN4, DOK5, LINC01441, LINC01440, CBLN4, MC3R, FAM210B, AURKA, CSTF1, CASS4, RTF2, GCNT7, FAM209A, FAM209B, LINC01716, TFAP2C, BMP7, BMP7-AS1, LOC112268270, MIR4325, SPO11, RAE1, RBM38-AS1, RBM38, CTCFL, PCK1, ZBP1, PMEPA1, NKILA, LINC01742, C20orf85, ANKRD60, PPP4R1L, RAB22A, VAPB, APCDD1L, APCDD1L-DT, LINC01711, STX16-NPEPL1, STX16, NPEPL1, LOC105372695, MIR296, MIR298, GNAS-AS1, GNAS, LOC101927932, NELFCD, CTSZ, TUBB1, ATP5F1E, SLMO2-ATP5E, PRELID3B, ZNF831, EDN3, PHACTR3, PHACTR3-AS1, SYCP2, FAM217B, PPP1R3D, CDH26, LINC02910, LOC729296, MIR646HG, LOC105372698, MIR646, LOC101928048, MIR4533, MIR548AG2, LINC01718, CDH4, LOC100128310, MIR1257, TAF4, MIR3195, LSM14B, PSMA7, SS18L1, MTG2, HRH3, LOC105369209, OSBPL2, ADRM1, LAMA5, MIR4758, LAMA5-AS1, RPS21, CABLES2, RBBP8NL, GATA5, CRMA, MIR1-1HG, MIR1-1, MIR133A2, LINC02970, SLCO4A1, SLCO4A1-AS2, SLCO4A1-AS1, NTSR1, LINC00659, MRGBP, OGFR-AS1, OGFR, COL9A3, TCFL5, DIDO1, SNORA117, GID8, SLC17A9, BHLHE23, LINC01749, LINC00029, LINC01056, HAR1B, HAR1A, MIR124-3, YTHDF1, BIRC7, MIR3196, NKAIN4, FLJ16779, ARFGAP1, MIR4326, COL20A1, CHRNA4, LOC100130587, KCNQ2, EEF1A2, PPDPF, PTK6, SRMS, FNDC11, HELZ2, GMEB2, MHENCR, STMN3, RTEL1, RTEL1-TNFRSF6B, TNFRSF6B, ARFRP1, ZGPAT, LIME1, SLC2A4RG, ZBTB46, ZBTB46-AS1, LOC112268269, ABHD16B, TPD52L2, DNAJC5, MIR941-1, MIR941-2, MIR941-3, MIR941-4, MIR941-5, UCKL1, MIR1914, MIR647, UCKL1-AS1, ZNF512B, SAMD10, PRPF6, C20orf204, SOX18, TCEA2, RGS19, MIR6813, OPRL1, LKAAEAR1, NPBWR2, MYT1, PCMTD2 | arr[GRCh38] 20q12q13.33(42,392,898_64,284,202)x3 |
| 2  Brain | cn-LOH | 21 | q11.2 | q21.2 | 10,189 | CYP4F29P, LOC110091776, ANKRD20A11P, LIPI, RBM11, ABCC13, HSPA13, SAMSN1, SAMSN1-AS1, ASMER1, NRIP1, USP25, MIR99AHG, SNORD74B, MIR99A, MIRLET7C, MIR125B2, LINC01549, CXADR, BTG3, BTG3-AS1, C21orf91-OT1, C21orf91, CHODL-AS1, CHODL, TMPRSS15, MIR548XHG, MIR548X, LINC01683, LINC02573, LINC00320, NCAM2, LINC00317, LINC01425, LINC01687, LINC00308, MIR6130, D21S2088E | arr[GRCh38] 21q11.2q21.2(13,757,057_23,946,545)x2 hmz |
| 2  Brain | cn-LOH | 21 | q21.2 | q22.3 | 21,618 | LINC01692, LINC00158, MIR155HG, MIR155, LINC00515, MRPL39, JAM2, ATP5PF, GABPA, APP, CYYR1-AS1, CYYR1, ADAMTS1, ADAMTS5, MIR4759, LINC01673, LINC00113, LINC00314, LINC01697, LINC01695, LINC00161, N6AMT1, LTN1, RWDD2B, USP16, CCT8, MAP3K7CL, LINC00189, BACH1, BACH1-IT2, BACH1-IT3, GRIK1, GRIK1-AS2, GRIK1-AS1, CLDN17, LINC00307, CLDN8, KRTAP24-1, KRTAP25-1, KRTAP26-1, KRTAP27-1, KRTAP23-1, KRTAP13-2, MIR4327, KRTAP13-1, KRTAP13-3, KRTAP13-4, KRTAP15-1, KRTAP19-1, KRTAP19-2, KRTAP19-3, KRTAP19-4, KRTAP19-5, KRTAP19-6, KRTAP19-7, KRTAP22-2, KRTAP6-3, KRTAP6-2, KRTAP22-1, KRTAP6-1, KRTAP20-1, KRTAP20-4, KRTAP20-2, KRTAP20-3, KRTAP21-3, KRTAP21-2, KRTAP21-1, KRTAP8-1, KRTAP7-1, KRTAP11-1, KRTAP19-8, TIAM1, TIAM1-AS1, SOD1, SCAF4, HUNK, LINC00159, MIS18A, MIS18A-AS1, MRAP, URB1, SNORA80A, URB1-AS1, EVA1C, CFAP298-TCP10L, TCP10L, CFAP298, SYNJ1, PAXBP1-AS1, PAXBP1, C21orf62-AS1, C21orf62, LINC01690, OLIG2, LINC00945, OLIG1, LOC101928107, LINC01548, IFNAR2, IFNAR2-IL10RB, IL10RB-DT, IL10RB, IFNAR1, IFNGR2, TMEM50B, DNAJC28, GART, SON, MIR6501, DONSON, CRYZL1, ITSN1, ATP5PO, LINC00649, LOC101928126, SLC5A3, MRPS6, LINC00310, KCNE2, SMIM11, C21orf140, SMIM34, KCNE1, RCAN1, CLIC6, LINC00160, LINC01426, RUNX1, RUNX1-IT1, LOC100506403, MIR802, PPP1R2P2, LOC101928269, LINC01436, SETD4, CBR1-AS1, CBR1, LOC105369306, CBR3-AS1, CBR3, DOP1B, MORC3, CHAF1B, CLDN14-AS1, CLDN14, SIM2, HLCS, RIPPLY3, PIGP, TTC3, TTC3-AS1, DSCR9, VPS26C, DYRK1A, KCNJ6-AS1, KCNJ6, DSCR4, DSCR8, DSCR10, KCNJ15, LINC01423, ERG, LINC00114, ETS2, LOC101928398, ETS2-AS1, LINC01700, PSMG1, BRWD1, BRWD1-AS2, BRWD1-AS1, HMGN1, GET1, GET1-SH3BGR, LCA5L, SH3BGR, MIR6508, B3GALT5-AS1, B3GALT5, IGSF5, PCP4, DSCAM, MIR4760, DSCAM-AS1, DSCAM-IT1, LINC00323, MIR3197, BACE2, PLAC4, FAM3B, MX2, MX1, TMPRSS2, PCSEAT, LINC00111, LINC00479, LINC00112, RIPK4, MIR6814, PRDM15, C2CD2, SNORA91, ZBTB21, ZNF295-AS1, UMODL1, UMODL1-AS1, ABCG1, TFF3, TFF2, TFF1, TMPRSS3, UBASH3A, RSPH1, RSPH1-DT, SLC37A1, LOC101928212, LINC01671, PDE9A, PDE9A-AS1, LINC01668, WDR4, NDUFV3, ERVH48-1, MIR5692B, PKNOX1, CBS, U2AF1, FRGCA, CRYAA, LINC00322, LINC01679, LNCSIK1, SIK1, LINC00319, LINC00313, HSF2BP, H2BC12L, MIR6070, RRP1B, PDXK, CSTB, RRP1, AATBC, AGPAT3, TRAPPC10, PWP2, GATD3, LINC01678, ICOSLG, DNMT3L, DNMT3L-AS1, AIRE, PFKL, CFAP410, TRPM2, TRPM2-AS, LRRC3-DT, LRRC3, LINC02575, TSPEAR, TSPEAR-AS1, TSPEAR-AS2, KRTAP10-1, KRTAP10-2, KRTAP10-3, KRTAP10-4, KRTAP10-5, KRTAP10-6, KRTAP10-7, KRTAP10-8, KRTAP10-9, KRTAP10-10, KRTAP10-11, KRTAP12-4, KRTAP12-3, KRTAP12-2, KRTAP12-1, KRTAP10-12, UBE2G2, LINC01424, SUMO3, PTTG1IP, ITGB2, ITGB2-AS1, LINC01547, SLX9, LINC00163, LINC00165, PICSAR, SSR4P1, ADARB1, LINC00334, POFUT2, LINC00205, LINC00316, BNAT1, COL18A1, COL18A1-AS2, COL18A1-AS1, MIR6815, SLC19A1, LINC01694, PCBP3, PCBP3-AS1, LOC101928796, COL6A1, COL6A2, FTCD, FTCD-AS1, SPATC1L, LSS, SNORD159, MCM3AP-AS1, MCM3AP, YBEY, C21orf58, PCNT, LOC128092249, DIP2A, DIP2A-IT1, S100B, PRMT2 | arr[GRCh38] 21q21.2q22.3(25,040,343_46,657,900)x2 hmz |
| 2  Brain | Gain | 22 | q11.21 | q12.2 | 12,930 | PEX26, TUBA8, USP18, FAM230D, FAM230J, FAM230A, GGTLC3, TMEM191B, PI4KAP1, RIMBP3, FAM246B, FAM230E, GGT3P, POM121L15P, LOC102724728, FAM230F, DGCR6, PRODH, LOC122455341, DGCR5, FAM246C, DGCR2, DGCR11, ESS2, TSSK2, GSC2, LINC01311, SLC25A1, CLTCL1, HIRA, MRPL40, C22orf39, UFD1, CDC45, CLDN5, LINC00895, SEPTIN5, SEPT5-GP1BB, GP1BB, TBX1, GNB1L, RTL10, TXNRD2, COMT, MIR4761, ARVCF, TANGO2, MIR185, DGCR8, MIR3618, MIR1306, TRMT2A, MIR6816, RANBP1, SNORA77B, ZDHHC8, CCDC188, LINC02891, LINC00896, RTN4R, MIR1286, DGCR6L, FAM230G, ZNF74, SCARF2, KLHL22, MED15, POM121L4P, TMEM191A, PI4KA, SERPIND1, SNAP29, CRKL, LINC01637, AIFM3, LZTR1, THAP7, THAP7-AS1, TUBA3FP, P2RX6, SLC7A4, MIR649, P2RX6P, LRRC74B, BCRP2, FAM230B, GGT2P, POM121L8P, FAM230H, LINC01651, FAM246A, RIMBP3B, HIC2, TMEM191C, PI4KAP2, RIMBP3C, UBE2L3, YDJC, CCDC116, SDF2L1, LOC107985532, MIR301B, MIR130B, PPIL2, YPEL1, MAPK1, PPM1F, PPM1F-AS1, TOP3B, PRAMENP, VPREB1, BMS1P20, ZNF280B, ZNF280A, PRAME, LL22NC03-63E9.3, POM121L1P, GGTLC2, MIR650, MIR5571, IGLL5, RSPH14, GNAZ, RAB36, BCR, FBXW4P1, LINC02556, CES5AP1, ZDHHC8BP, LINC01659, FAM230I, PCAT14, IGLL1, DRICH1, GUSBP11, RGL4, ZNF70, VPREB3, C22orf15, CHCHD10, MMP11, SMARCB1, DERL3, SLC2A11, MIF-AS1, MIF, LOC100652871, GSTT2B, DDTL, DDT, GSTT2, GSTT4, CABIN1, SUSD2, GGT5, POM121L9P, SPECC1L-ADORA2A, SPECC1L, ADORA2A, ADORA2A-AS1, UPB1, GUCD1, SNRPD3, GGT1, LRRC75B, BCRP3, POM121L10P, PIWIL3, TOP1P2, SGSM1, LHFPL7, KIAA1671, KIAA1671-AS1, CRYBB3, CRYBB2, IGLL3P, CRYBB2P1, MIR6817, GRK3-AS1, GRK3, MYO18B, SEZ6L, ASPHD2, HPS4, SRRD, TFIP11, TFIP11-DT, TPST2, MIR548J, CRYBB1, CRYBA4, MIAT, MIATNB, LOC110091768, LINC01422, LOC284898, LOC105372977, LINC01638, LINC02554, MN1, PITPNB, TTC28-AS1, MIR3199-1, MIR3199-2, TTC28, MIR5739, CHEK2, HSCB, CCDC117, XBP1, ZNRF3, ZNRF3-AS1, C22orf31, KREMEN1, EMID1, RHBDD3, EWSR1, GAS2L1, RASL10A, AP1B1, SNORD125, RFPL1, RFPL1S, NEFH, THOC5, NIPSNAP1, NF2, CABP7, ZMAT5, UQCR10, ASCC2, MTMR3, MIR6818, HORMAD2-AS1, HORMAD2, LIF-AS1, LIF, LIF-AS2, OSM, CASTOR1, TBC1D10A, SF3A1, CCDC157, KIAA1656, RNF215, SEC14L2, MTFP1, LOC105372990, SEC14L3, SDC4P, SEC14L4, SEC14L6, GAL3ST1, PES1, TCN2, SLC35E4, DUSP18, OSBP2, MIR3200, LOC107985544, MORC2-AS1, MORC2, TUG1 | arr[GRCh38] 22q11.21q12.2(18,071,042_31,000,625)x3 |
| 2  Brain | Gain | 22 | q12.3 | q13.33 | 17,716 | SYN3, LINC01640, LARGE1, MIR4764, SNORA50B, LARGE-AS1, LINC01643, LINC02885, ISX, LINC01399, HMGXB4, TOM1, MIR3909, MIR6069, HMOX1, MCM5, RASD2, MB, APOL6, APOL5, RBFOX2, APOL3, APOL4, APOL2, APOL1, MYH9, MIR6819, MYH9-DT, TXN2, FOXRED2, EIF3D, CACNG2, CACNG2-DT, IFT27, PVALB, NCF4-AS1, NCF4, CSF2RB, LL22NC01-81G9.3, TEX33, TST, MPST, KCTD17, TMPRSS6, IL2RB, C1QTNF6, SSTR3, RAC2, CYTH4, ELFN2, LOC100506271, MFNG, CARD10, CDC42EP1, LGALS2, GGA1, SH3BP1, PDXP-DT, PDXP, LGALS1, NOL12, TRIOBP, LOC102724378, H1-0, GCAT, GALR3, ANKRD54, MIR658, MIR659, EIF3L, MICALL1, C22orf23, POLR2F, MIR6820, SOX10, MIR4534, PICK1, SLC16A8, BAIAP2L2, PLA2G6, MAFF, TMEM184B, SNORA92, CSNK1E, TPTEP2-CSNK1E, TPTEP2, KCNJ4, KDELR3, DDX17, DMC1, LOC105373031, FAM227A, CBY1, TOMM22, JOSD1, GTPBP1, SUN2, DNAL4, NPTXR, CBX6, APOBEC3A, APOBEC3B, APOBEC3B-AS1, APOBEC3C, APOBEC3D, APOBEC3F, APOBEC3G, APOBEC3H, CBX7, PDGFB, RPL3, SNORD83B, SNORD83A, SNORD139, SNORD43, SYNGR1, TAB1, LOC100506472, MGAT3, MGAT3-AS1, MIEF1, MIURF, ATF4, RPS19BP1, CACNA1I, ENTHD1, GRAP2, TMA7B, FAM83F, TNRC6B-DT, TNRC6B, ADSL, SGSM3, MRTFA, MRTFA-AS1, MCHR1, SLC25A17, MIR4766, ST13, XPNPEP3, DNAJB7, RBX1, SNORD140, MIR1281, EP300, EP300-AS1, L3MBTL2, L3MBTL2-AS1, CHADL, RANGAP1, MIR6889, ZC3H7B, TEF, TOB2, PHF5A, ACO2, POLR3H, LOC105373044, CSDC2, PMM1, DESI1, XRCC6, SNU13, C22orf46P, MEI1, CCDC134, SREBF2-AS1, SREBF2, MIR33A, SHISA8, TNFRSF13C, MIR378I, CENPM, SMIM45, SEPTIN3, WBP2NL, NAGA, PHETA2, SMDT1, NDUFA6, NDUFA6-DT, CYP2D6, CYP2D7, TCF20, OGFRP1, LINC01315, NFAM1, SERHL, RRP7A, SERHL2, RRP7BP, POLDIP3, RNU12, CYB5R3, ATP5MGL, A4GALT, ARFGAP3, PACSIN2, TTLL1-AS1, TTLL1, BIK, MCAT, TSPO, TTLL12, SCUBE1, SCUBE1-AS2, SCUBE1-AS1, LINC01639, MPPED1, EFCAB6-AS1, EFCAB6, SULT4A1, PNPLA5, PNPLA3, SAMM50, PARVB, PARVG, SHISAL1, LINC01656, RTL6, LINC00207, LINC00229, PRR5, PRR5-ARHGAP8, ARHGAP8, PHF21B, LOC101927551, NUP50-DT, NUP50, LOC105373064, KIAA0930, MIR1249, UPK3A, FAM118A, SMC1B, RIBC2, FBLN1, LINC01589, ATXN10, MIR4762, LOC107985535, WNT7B, LOC730668, LINC00899, PRR34, PRR34-AS1, LINC02939, MIRLET7BHG, MIR3619, MIRLET7A3, MIR4763, MIRLET7B, PPARA, CDPF1, PKDREJ, TTC38, GTSE1-DT, GTSE1, TRMU, CELSR1, GRAMD4, CERK, TBC1D22A, TBC1D22A-AS1, LOC339685, LINC01644, LINC00898, EPIC1, MIR3201, TAFA5, LOC284933, MIR4535, LINC01310, NHIP, MIR3667HG, MIR3667, BRD1, ZBED4, ALG12, CRELD2, PIM3, MIR6821, IL17REL, TTLL8, MLC1, MOV10L1, PANX2, TRABD, SELENOO, TUBGCP6, HDAC10, MAPK12, MAPK11, PLXNB2, DENND6B, PPP6R2, MIR12114, SBF1, ADM2, MIOX, LMF2, NCAPH2, SCO2, TYMP, ODF3B, KLHDC7B, SYCE3, CPT1B, CHKB-CPT1B, CHKB, CHKB-DT, MAPK8IP2, ARSA | arr[GRCh38] 22q12.3q13.33(32,949,562_50,665,715)x3 |
| 2  Brain | Loss | X | p22.33 | p22.2 | 15,833 | PPP2R3B, SHOX, CRLF2, CSF2RA, MIR3690, IL3RA, SLC25A6, LINC00106, ASMTL-AS1, ASMTL, P2RY8, AKAP17A, ASMT, DHRSX, ZBED1, MIR6089, CD99P1, LINC00102, CD99, XG, GYG2, ARSD, ARSD-AS1, ARSL, ARSH, ARSF, LINC01546, MXRA5, SNORA48B, PRKX, PRKX-AS1, LOC389906, FAM239A, FAM239B, LOC101928201, NLGN4X, LOC105373156, MIR4770, VCX3A, PUDP, STS, MIR4767, VCX, PNPLA4, MIR651, VCX2, VCX3B, ANOS1, FAM9A, FAM9B, TBL1X, GPR143, SHROOM2, CLDN34, WWC3, CLCN4, MID1, HCCS, ARHGAP6, AMELX, MIR548AX, MSL3, FRMPD4, FRMPD4-AS1, PRPS2, TLR7, TLR8-AS1, TLR8, TMSB4X, FAM9C, LOC105373133, LINC02154, GS1-600G8.3, ATXN3L, LINC01203, EGFL6, MIR6086, LOC107985657, TCEANC, RAB9A, TRAPPC2, OFD1, GPM6B, GEMIN8, UBE2E4P, GLRA2, FANCB, MOSPD2, ASB9, ASB11, PIGA, PIR-FIGF, VEGFD, PIR, BMX, ACE2, ACE2-DT, CLTRN, CA5BP1, CA5BP1-CA5B, CA5B, INE2, ZRSR2, AP1S2, GRPR, MAGEB17 | arr[GRCh38] Xp22.33p22.2(352,513_16,185,955)x1 |
