# Supplementary material for: High resolution genome-wide SNP array analyses on matched colorectal-based lung and brain metastases
Source: J Cancer Res Clin Oncol. 2026 Jan 30;152(2):47. doi: 10.1007/s00432-026-06427-7 (PMC12858677; doi:10.1007/s00432-026-06427-7)
Supplement: Supplementary file 2 — Supplementary Material 2 [file 432_2026_6427_MOESM2_ESM.docx]

**Detected cancer genes (according to Network of Cancer Genes and Healthy Drivers), based on all detected cn-LOH aberrant regions (730 different cancer genes)**

| **Patient** | **Sample** | **Type** | **Chromosome** | **Cytoband Start** | **Cytoband End** | **Size (kbp)** | **Genes** |
| --- | --- | --- | --- | --- | --- | --- | --- |
| 2 | Brain | LOH | 1 | p36.21 | p34.3 | 23,024 | *EFHD2*, *CASP9*, *SPEN*, *EPHA2*, *NBPF1*, *SDHB*, *ARHGEF10L*, *NBL1*, *HSPG2*, *CDC42*, *KDM1A*, *LUZP1*, *E2F2*, *ID3*, *MYOM3*, *SYF2*, *MTFR1L*, *CNKSR1*, *ARID1A*, *WDTC1*, *SYTL1*, *MAP3K6*, *SESN2*, SNHG3, *RAB42*, *YTHDF2*, *OPRD1*, *EPB41*, *PTPRU*, *LCK*, *HDAC1*, *ZNF362*, *PHC2*, *CSMD2*, *SFPQ*, *AGO4*, *THRAP3*, *CSF3R*, *GRIK3*, *ZC3H12A* |
| 1 | Brain | LOH | 3 | p21.31 | p21.1 | 6,017 | *DHX30*, *CDC25A*, *COL7A1*, *P4HTM*, *C3orf62*, *GPX1*, *RHOA*, *MST1*, *CDHR4*, *TRAIP*, *MST1R*, *GNAT1*, *SLC38A3*, *GNAI2*, *ZMYND10*, *RBM15B*, *DCAF1*, *RPL29*, *DUSP7*, *TLR9*, *DNAH1*, *BAP1*, *STAB1*, *PBRM1*, *ITIH1*, *CACNA1D* |
| 2 | Brain | LOH | 4 | p16.3 | p11 | 49,018 | *ZNF595*, *FGFR3*, *LETM1*, *NSD2*, *NAT8L*, *RNF4*, *MFSD10*, *RGS12*, *OTOP1*, *TMEM128*, *EVC2*, *SORCS2*, *ABLIM2*, *TRMT44*, *DRD5*, *CPEB2*, *FGFBP1*, *PROM1*, *NCAPG*, *SLIT2*, *DHX15*, *SLC34A2*, *RBPJ*, *STIM2*, *DTHD1*, *TLR6*, *KLHL5*, *RFC1*, *PDS5A*, *N4BP2*, *RHOH*, *LIMCH1*, *PHOX2B*, *KCTD8*, *GABRG1*, *GABRA2*, *ATP10D*, *TEC*, *CWH43* |
| 2 | Brain | LOH | 4 | q12 | q35.2 | 138,161 | *LRRC66*, *SPATA18*, *PDGFRA*, *KIT*, *KDR*, *TMEM165*, *CRACD*, *ADGRL3*, *EPHA5*, *UBA6*, *TMPRSS11D*, *TMPRSS11A*, *UGT2B10*, *UGT2A3*, *UGT2A2*, *UGT2A1*, *SULT1B1*, *ODAM*, *GRSF1*, *MOB1B*, *DCK*, *ADAMTS3*, *ANKRD17*, *ALB*, *AFM*, *RASSF6*, *AREG*, *G3BP2*, *NUP54*, *SHROOM3*, *CCNI*, PCAT4, *FGF5*, *HNRNPD*, *HNRNPDL*, *WDFY3*, *PTPN13*, *KLHL8*, *DSPP*, *PKD2*, *ABCG2*, *PIGY*, *PDLIM5*, *UNC5C*, *RAP1GDS1*, *ADH1B*, *PPP3CA*, *CXXC4*, *TET2*, *INTS12*, *NPNT*, *LEF1*, *RPL34*, *COL25A1, FAM241A*, *ALPK1*, *NEUROG2*, *LARP7*, *ANK2*, *NDST4*, *METTL14*, *FGF2*, *ANKRD50*, *FAT4*, *PCDH10*, *PABPC4L*, *PCDH18*, *SETD7*, *MAML3*, *INPP4B*, *NR3C2*, *RPS3A*, *FBXW7*, *TLR2*, *PLRG1*, *FGB*, *FGA*, *PDGFC*, *GRIA2*, *FSTL5*, *TKTL2*, *TLL1*, *DDX60L*, *GALNTL6*, *GLRA3*, *ADAM29*, *DCTD*, *CDKN2AIP*, *IRF2*, *CASP3*, *SNX25*, *FAT1*, *FRG1* |
| 1 | Brain | LOH | 5 | p15.33 | p11 | 46,246 | *SDHA*, *PDCD6*, *TERT*, *SLC6A3*, *MRPL36*, *ADCY2*, CFAP90, *SEMA5A*, *CTNND2*, *TRIO*, *MARCHF11, CDH18*, *CDH12*, *PRDM9*, *CDH10*, *CDH9*, *CDH6*, *DROSHA*, *PDZD2*, *NPR3*, *ADAMTS12*, *RXFP3*, *PRLR*, *IL7R*, *SKP2*, *SLC1A3*, *NIPBL*, *LIFR*, *OSMR*, *RICTOR*, *CARD6*, *C7*, *ZNF131*, *PAIP1*, *HCN1* |
| 1 | Brain | LOH | 5 | q11.1 | q13.2 | 19,244 | *MTREX*, *IL6ST*, *MAP3K1*, *MIER3*, *ZSWIM6*, *ADAMTS6*, *TRIM23*, *NLN*, *ERBIN*, *MAST4*, *PIK3R1*, *RAD17* |
| 1 | Brain | LOH | 5 | q13.2 | q35.3 | 109,874 | *TNPO1*, *ENC1*, *POC5*, *IQGAP2*, *AGGF1*, *BHMT2*, *MSH3*, *RASGRF2*, *VCAN*, *HAPLN1*, *COX7C*, *RASA1*, *MEF2C*, *ADGRV1*, *CAST*, *RIOK2*, *CHD1*, *FBXL17*, *MAN2A1*, *APC*, *REEP5*, *KCNN2, SEMA6A*, *DTWD2*, *DMXL1*, *TNFAIP8*, *ZNF608*, *PHAX*, *FBN2*, *ADAMTS19*, *ACSL6*, *IRF1*, *AFF4*, *HSPA4*, *TCF7*, *TRPC7*, *BRD8*, *CDC23*, *CDC25C*, *EGR1*, *CTNNA1*, *CXXC5*, *NRG2, PCDHA10*, *PCDHA11*, *PCDHA13*, *PCDHAC2*, *PCDHB1*, *PCDHB6*, *PCDHB8*, *PCDHGB1*, *PCDHGA8*, *PCDHGC5*, *DIAPH1*, *ARHGAP26*, *NR3C1*, *RBM27*, *TCERG1*, *JAKMIP2*, *CSNK1A1*, *CSF1R*, *PDGFRB*, *CD74*, *MYOZ3*, *FAT2*, *G3BP1*, *NMUR2*, *LARP1*, *HAVCR1*, *ITK*, *EBF1, ADRA1B*, *ATP10B*, *GABRB2*, *GABRA6*, *GABRA1*, *TENM2*, *SLIT3*, *DOCK2*, *INSYN2B*, *TLX3*, *NPM1*, *ATP6V0E1*, *BOD1*, *CPEB4*, *MSX2*, *CDHR2*, *GPRIN1*, *HK3*, *FGFR4*, *NSD1*, *DDX41*, *CLK4*, *RUFY1*, *CANX*, *MAML1*, *RASGEF1C*, *FLT4*, *BTNL3* |
| 1 | Brain | LOH | 6 | q16.3 | q21 | 7,496 | *PRDM1*, *PDSS2*, *FOXO3*, *REV3L*, *FYN*, *CCN6*, *LAMA4* |
| 2 | Brain | LOH | 8 | q24.22 | q24.3 | 11,916 | *NDRG1*, *FAM135B*, *COL22A1*, *PTK2*, *LY6K*, *SLURP1*, *GML*, *MAFA*, *EEF1D*, *EPPK1*, *PLEC*, *MROH1*, *HSF1*, *RECQL4* |
| 2 | Brain | LOH | 9 | p21.1 | p13.2 | 7,267 | *ACO1*, RIGI, *TAF1L*, *SMU1*, *AQP7*, *PRSS3*, *FANCG*, *RUSC2*, *CCDC107*, *FAM221B*, *GLIPR2*, *PAX5* |
| 2 | Brain | LOH | 10 | q22.1 | q22.3 | 6,123 | *CFAP70*, *KAT6B*, *ZNF503* |
| 1 | Brain | LOH | 12 | q14.2 | q24.33 | 70,192 | *TBK1*, *WIF1*, *LEMD3*, *HMGA2*, *IL22*, *MDM2*, *YEATS4*, *PTPRB*, *TRHDE*, *GLIPR1L2*, *PHLDA1*, *NAP1L1*, *OSBPL8*, *NAV3*, *OTOGL*, *LIN7A*, *TMTC2*, *SLC6A15*, *LRRIQ1*, *TMTC3*, *CCER1*, *EPYC*, *LUM*, *BTG1*, *CRADD*, *CEP83*, *USP44*, *NTN4*, *CCDC38*, *LTA4H, CFAP54*, *DEPDC4*, *SCYL2*, *NR1H4*, *ANO4*, *UTP20*, *CHPT1*, *GNPTAB*, *TDG*, *GLT8D2*, *HCFC2*, *CHST11*, *NUAK1*, *TCP11L2*, *WSCD2*, *CMKLR1*, *SSH1*, *DAO*, *MYO1H*, *MMAB*, *ANKRD13A*, *ATP2A2*, *HVCN1*, *CCDC63*, *SH2B3*, *MAPKAPK5*, *TMEM116*, *PTPN11*, *DTX1*, *RASAL1*, *DDX54*, *TBX3*, *TESC, NOS1*, *SIRT4*, *SRSF9*, *DYNLL1*, *HNF1A*, *CAMKK2*, *KDM2B*, *SETD1B*, *HPD*, *BCL7A*, *CLIP1*, *ZCCHC8*, *PITPNM2*, *SNRNP35*, *DDX55*, *RFLNA*, *NCOR2*, *TMEM132B*, *TMEM132C*, *TMEM132D*, *RIMBP2*, *STX2*, *ADGRD1*, *EP400*, *DDX51*, *FBRSL1*, *POLE*, *ANKLE2*, *ZNF605* |
| 2 | Brain | LOH | 17 | p13.3 | p11.2 | 17,945 | *RFLNB*, *VPS53*, *YWHAE*, *SMG6*, *ITGAE*, *ZZEF1*, *USP6*, *RPAIN*, *NLRP1*, *MED31*, *ALOX12*, *CTDNEP1*, *EIF5A*, *GPS2*, *NEURL4, EIF4A1*, *TP53*, *DNAH2*, *CHD3*, *ALOX12B*, *PER1*, *PFAS*, *ODF4*, *NDEL1*, *PIK3R6*, *PIK3R5*, MYHAS, *MYH4*, *TMEM220*, *DNAH9*, *MAP2K4*, *MYOCD*, *ZSWIM7*, *NCOR1*, *UBB*, *ZNF624*, *FLCN* |
| 2 | Brain | LOH | 18 | q11.1 | q23 | 59,276 | *GATA6*, *CABLES1*, *LAMA3*, *HRH4*, *ZNF521*, *CDH2*, *DSC3*, *DSC1*, *DSG1*, *DSG4*, *CCDC178*, *ASXL3*, *DTNA*, *CELF4*, *PIK3C3*, *RIT2*, *SYT4*, *SETBP1*, *EPG5*, *LOXHD1*, *SMAD2*, *CTIF*, *MYO5B*, *MBD1*, *MAPK4*, *SMAD4*, *DCC*, *STARD6*, *TCF4*, *WDR7*, *ONECUT2*, *ATP8B1*, *ALPK2*, *MALT1*, *ZCCHC2*, *PHLPP1*, *BCL2*, *SERPINB13*, *SERPINB7*, *CDH19*, *DSEL*, *TMX3*, *CCDC102B*, *DOK6*, *RTTN*, *CNDP2*, *SALL3* |
| 1 | Brain | LOH | 19 | q11 | q13.43 | 30,786 | *CCNE1*, *ZNF536*, *TSHZ3*, *DPY19L3*, *RHPN2*, *CEBPA*, *PDCD2L*, *UBA2*, *WTIP*, *ZNF181*, *FXYD3*, *SBSN*, *TMEM147*, *ATP4A*, *KMT2B*, *ALKBH6*, *WDR62*, *ZFP14*, *PSMD8*, *RYR1*, *HNRNPL*, *RINL*, *AKT2*, *PRX*, *SPTBN4*, *SHKBP1*, *LTBP4*, *SNRPA*, *CYP2A7, CEACAM21*, *CD79A*, *GRIK5*, *POU2F2*, *GSK3A*, *ERF*, *CIC*, *ZNF226*, *ZNF227*, *ZNF180*, *BCL3*, *CBLC*, *RELB*, *ZNF296*, *ERCC2*, *POLR1G*, *SNRPD2*, *CCDC61*, *PRKD2*, *ARHGAP35*, *TMEM160*, *ZC3H4*, *CCDC9*, *BICRA*, *NOP53*, *KDELR1*, *GRIN2D, SULT2B1*, *MAMSTR*, *PPP1R15A*, *BAX*, *CGB2*, *CGB5*, *KASH5*, *RPL13A*, *RRAS*, *BCL2L12*, *AP2A1*, *POLD1*, *SPIB*, *SHANK1*, *GPR32*, *KLK1*, *KLK3*, *KLK6*, *KLK7*, *SIGLEC8*, *FPR1*, *PPP2R1A*, *ZNF578*, *ZNF28*, *ZNF677*, *VN1R2*, *VN1R4*, *ZNF765*, *ZNF331*, *PRKCG*, *CACNG6*, *CNOT3*, *LILRB2*, *LILRB1*, *KIR3DL1*, *EPS8L1*, *BRSK1*, *RPL28*, *ISOC2*, *FIZ1*, *U2AF2*, *NLRP5*, *ZNF471*, *ZIM2*, *PEG3*, *USP29*, *DUXA*, *ZSCAN4*, *ZNF814*, *ZNF497*, *ZNF324*, *TRIM28* |
| 2 | Brain | LOH | 20 | p13 | p12.1 | 14,451 | *DEFB129*, *CSNK2A1*, *PSMF1*, *SIRPB2*, *SIRPD*, *SIRPB1*, *SIRPG*, *SIRPA*, *PDYN*, *PROKR2*, *PLCB4*, *PAK5*, *JAG1*, *MACROD2* |
| 2 | Brain | LOH | 20 | p12.1 | p11.21 | 7,859 | *MACROD2*, *KIF16B*, *BANF2*, *ZNF133*, *CRNKL1*, *FOXA2* |
| 2 | Brain | LOH | 20 | q11.21 | q12 | 10,836 | *REM1*, *HM13*, *TPX2*, *TTLL9*, *ASXL1*, *DNMT3B*, *BPIFB1*, *PXMP4*, *RALY*, *EIF2S2*, *ITCH*, *MMP24*, *GDF5*, *ERGIC3*, *CPNE1*, *RBM12*, *RBM39*, *SAMHD1*, *SRC*, *BLCAP*, *VSTM2L*, *RPRD1B*, *LBP*, *ADIG*, *DHX35*, *MAFB*, *PLCG1*, *CHD6*, *PTPRT* |
| 2 | Brain | LOH | 21 | q11.2 | q21.2 | 10,189 | *NRIP1*, *USP25*, *CXADR*, *NCAM2* |
| 2 | Brain | LOH | 21 | q21.2 | q22.3 | 21,618 | *ADAMTS5*, *USP16*, *KRTAP13-3*, *KRTAP20-2*, *SCAF4*, *URB1*, *OLIG2*, *DNAJC28*, *SON*, *SLC5A3*, *RUNX1*, *CHAF1B*, *DYRK1A*, *ERG*, *TMPRSS2*, *U2AF1*, *TRPM2*, *TSPEAR*, *KRTAP12-1*, SLX9, *PCBP3*, *FTCD*, *SPATC1L* |
