# Supplementary material for: High resolution genome-wide SNP array analyses on matched colorectal-based lung and brain metastases
Source: J Cancer Res Clin Oncol. 2026 Jan 30;152(2):47. doi: 10.1007/s00432-026-06427-7 (PMC12858677; doi:10.1007/s00432-026-06427-7)
Supplement: Supplementary file 3 — Supplementary Material 3 [file 432_2026_6427_MOESM3_ESM.docx]

**
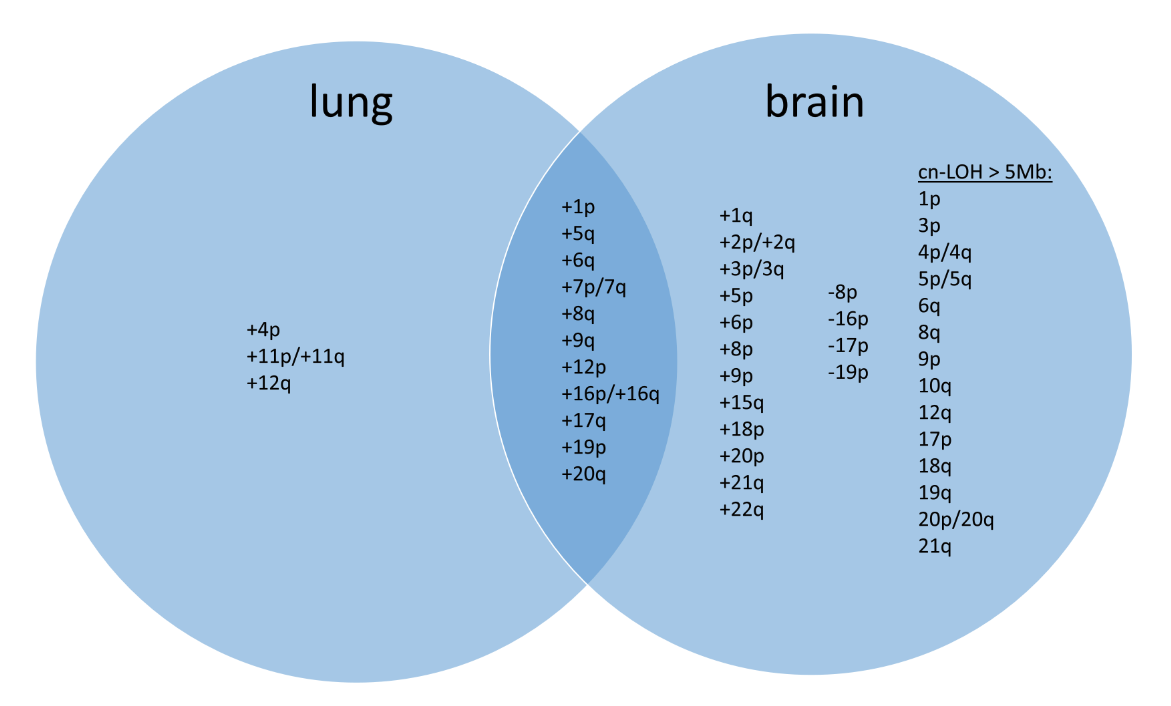
**

**Fig 5 Representation of the chromosomal aberrations in a Venn diagram.** The aberrant chromosomal regions (p or q) were assigned to lung metastases, brain metastases, or both metastatic sites.


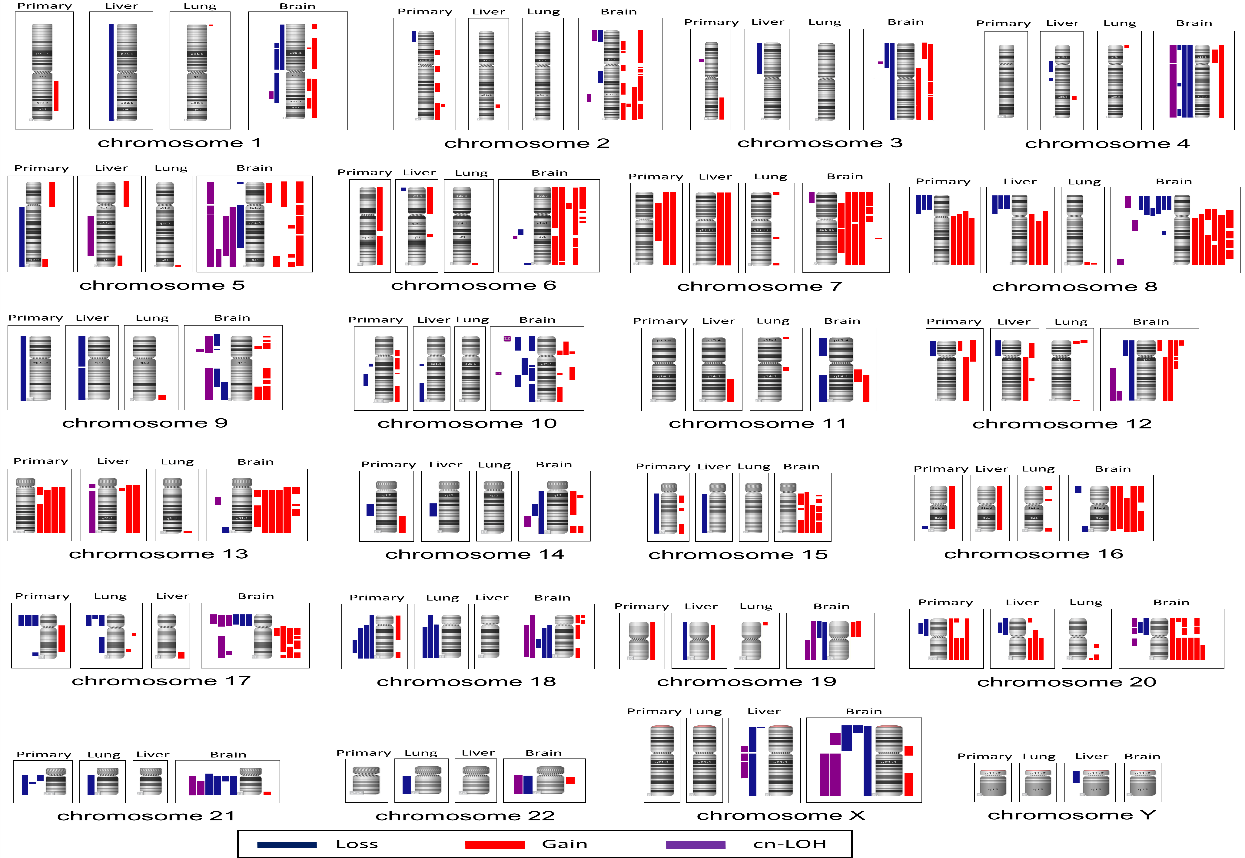


**Fig 6 Overview of all detected chromosomal aberrations sorted for primary CRC and each metastasis.** All detected aberrations identified in primary CRC, lung metastasis, liver metastasis and brain metastasis (from left to right) are shown. Gains = red; loss = blue; cn-LOH = violet.
